# Allosteric Guest Binding in Chiral Zirconium(IV) Double Decker Porphyrin Cages

**DOI:** 10.1002/ejoc.202001392

**Published:** 2021-01-05

**Authors:** Jeroen P. J. Bruekers, Matthijs A. Hellinghuizen, Nicolas Vanthuyne, Paul Tinnemans, Pieter J. Gilissen, Wybren Jan Buma, Jean‐Valère Naubron, Jeanne Crassous, Johannes A. A. W. Elemans, Roeland J. M. Nolte

**Affiliations:** ^1^ Radboud University Institute for Molecules and Materials Heyendaalseweg 135 6525 AJ Nijmegen The Netherlands; ^2^ Aix Marseille Univ CNRS Centrale Marseille iSm2 Marseille France; ^3^ University of Amsterdam Van ‘t Hoff Institute for Molecular Sciences Science Park 904 1098 XH Amsterdam The Netherlands; ^4^ Radboud University Institute for Molecules and Materials FELIX Laboratory Toernooiveld 7c 6525 ED Nijmegen The Netherlands; ^5^ Aix Marseille Univ CNRS Centrale Marseille Spectropole FR1739 Marseille France; ^6^ Univ Rennes CNRS Institut des Sciences Chimiques de Rennes ISCR-UMR 6226 35000 Rennes France

**Keywords:** Allostery, Cooperativity, Host-guest chemistry, Porphyrin, Zirconium

## Abstract

Chiral zirconium(IV) double cage sandwich complex **Zr(1)_2_** has been synthesized in one step from porphyrin cage **H_2_1. Zr(1)_2_** was obtained as a racemate, which was resolved by HPLC and the enantiomers were isolated in >99.5 % ee. Their absolute configurations were assigned on the basis of X‐ray crystallography and circular dichroism spectroscopy. Vibrational circular dichroism (VCD) experiments on the enantiomers of **Zr(1)_2_** revealed that the chirality around the zirconium center is propagated throughout the whole cage structure. The axial conformational chirality of the double cage complex displayed a VCD fingerprint similar to the one observed previously for a related chiral cage compound with planar and point chirality. **Zr(1)_2_** shows fluorescence, which is quenched when viologen guests bind in its cavities. The binding of viologen and dihydroxybenzene derivatives in the two cavities of **Zr(1)_2_** occurs with negative allostery, the cooperativity factors α (=4 K_2_/K_1_) being as low as 0.0076 for the binding of *N,N’*‐dimethylviologen. These allosteric effects are attributed to a pinching of the second cavity as a result of guest binding in the first cavity.

## Graphical abstract

Starting from achiral porphyrin cage compounds, chiral zirconium(IV) double decker porphyrin cages have been prepared and their enantiomers resolved. The binding of two viologen guests in the double decker porphyrin cages occurs via a strongly negative allosteric effect, resulting in an almost 3 orders of magnitude weaker binding of the second viologen guest compared to that of the first.

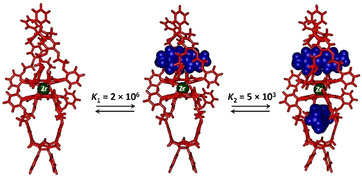

## Introduction

**Figure 1:**
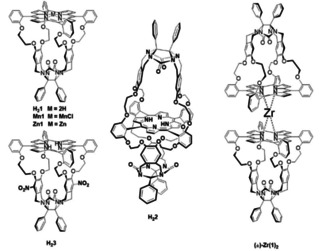
Structures of glycoluril‐based porphyrin cages.

To mimic the action of porphyrin‐containing enzymes, a wealth of synthetic model systems have been constructed in which (metallo)porphyrins are provided with bulky substituents, caps, and straps.[^1^, ^2^, ^3^, ^4^, ^5^] Many of these model compounds have been employed to study electron transfer processes and the binding of O_2_ and CO to their metal centers. For the additional binding and catalytic conversion of substrates, more sophisticated porphyrin architectures, in which specific substrate‐recognition functionalities[^6^, ^7^, ^8^, ^9^, ^10^, ^11^, ^12^] or macrocyclic receptor cavities such as cyclocholates,^[13]^ cyclodextrins,[^14^, ^15^, ^16^] cyclophanes,^[17]^ calixarenes,[^18^, ^19^, ^20^] cages,[^21^, ^22^, ^23^] tweezers,[^24^, ^25^] and boxes[^26^, ^27^, ^28^, ^29^] have been developed. In our group we have designed cage molecules derived from glycoluril and imparted them with a porphyrin roof, e. g. **H_2_1** (Figure 1).[^30^, ^31^] They feature very strong binding of viologen guests. When a metal center is inserted into the porphyrin, e. g. manganese(III), the cage compound (**Mn1**) acts as a biomimetic catalyst in the epoxidation of low molecular weight[^32^, ^33^] and polymeric alkenes, which thread through the cavity of the cage compound.[^34^, ^35^, ^36^] Another interesting aspect of the glycoluril‐based metalloporphyrin cages is that they display cooperative host‐guest binding properties. For instance, binding of a viologen guest in the cavity of **Zn1** is enhanced when a bulky pyridine ligand coordinates to the zinc center at the outside of the cage molecule.[^37^, ^38^] As part of a program aimed at the development of biomimetic catalytic systems that can write information on a polymeric chain in a controlled fashion,[^39^, ^40^] we intend to develop double porphyrin cage molecules that display allosteric host‐guest binding interactions. The idea is that a polyolefin substrate threads through one cavity and is catalytically converted into a poly‐epoxide, while the second cavity can bind a cofactor^[41]^ that controls the catalytic reaction via structural (allosteric) changes in the first cavity. This allosteric control may involve a change in shape, size, or chirality of the first cavity, thereby inducing shape‐ or stereo‐selectivity in the catalytic conversion of the polymer substrate, or changes in its threading speed^[42]^ and/or direction. Allosteric regulation is widely encountered in natural systems, and many artificial host‐guest systems displaying allosteric binding have been investigated in the literature in order to better understand these processes.[^22^, ^43^, ^44^, ^45^, ^46^, ^47^, ^48^, ^49^]

Along our efforts to develop porphyrin double cage compounds, we previously reported on **H_2_2** (Figure 1) in which a porphyrin molecule is featured to which two glycoluril cages are attached via 8 covalent bonds.[^50^, ^51^] This host was found to bind two viologen guests via negative allosteric cooperativity, i. e., the second viologen was bound more than 3 orders of magnitude weaker than the first. However, significant drawbacks for the application of this system in further research towards processive enzyme mimicry are its very cumbersome synthesis and the fact that the compound can only be obtained in extremely low yield (≈0.5 % in the final step, <1 mg per batch). We therefore explored alternative synthesis routes to obtain allosterically binding porphyrin cages more efficiently. In this paper we report the double porphyrin cage derivative **Zr(1)_2_** (Figure 1), in which two **H_2_1**‐cages are linked in a single step via coordination to a zirconium(IV) center. The resulting sandwich complex, which is readily obtainable in >100 mg quantities, has a twisted structure and is chiral. We were able to resolve the racemate and determine the absolute configuration of the enantiomers by X‐ray diffraction. **Zr(1)_2_** was found to bind viologens and resorcinol derivatives via negative allosteric cooperativity as a result of structural reorganizations throughout the whole double cage complex upon consecutive binding of the guest molecules.

## Results and Discussion

### Synthesis, resolution, and crystallographic characterization

**Figure 2:**
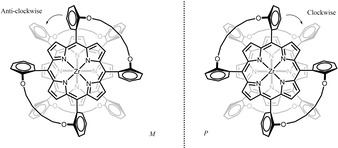
Schematic structure (top view) of the enantiomers of **(−)‐Zr(1)_2_** (left) and **(+)‐Zr(1)_2_** (right); the curved lines represent the side‐walls of the molecular cages attached to the porphyrin rings. The clockwise and anti‐clockwise enantiomers have the (*P*) and (*M*)‐configuration, respectively. The glycoluril units have been omitted for clarity.

Double cage compound **Zr(1)_2_** was synthesized in 33 % yield and in substantial quantities (up to 160 mg per batch) by stirring the free base porphyrin cage compound **H_2_1** with freshly prepared Zr(Et_2_N)_4_ in refluxing toluene. The same approach had previously been used by Kim et al. to prepare Zr(TPP)_2_ (TPP=tetrakis‐*meso*‐phenyl porphyrin), i. e. the double decker porphyrin complex without glycoluril‐based cages.^[52]^ Since the environment around the zirconium(IV) center becomes chiral upon complexation of the two C_2v_‐symmetric porphyrin cages, the product is obtained as a racemic mixture of (*M*)‐ and (*P*)‐enantiomers (Figure 2). With the help of chiral HPLC we were able to resolve these enantiomers, which were obtained with *ee*‐values of >99.5 %. In theory, the enantiomers can interconvert via a 90° rotation of one of the cage molecules relative to the other. However, **Zr(1)_2_** turned out to be very stable against thermal racemization, as refluxing a solution of the pure enantiomer **(+)‐Zr(1)_2_** in toluene for 18 h did not result in any changes in its CD spectrum (Figure S12). This result is in line with previous reports on other chiral zirconium porphyrin sandwich complexes, which were also inert to thermal racemization.^[53]^

**Figure 3:**
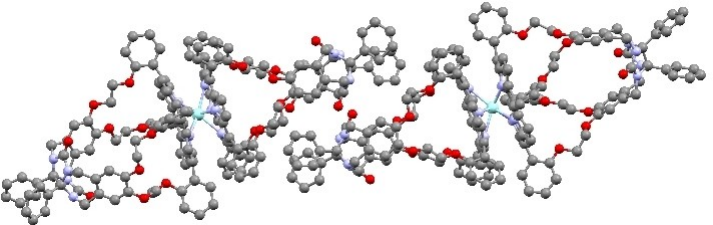
Crystal structure of **(−)‐Zr(1)_2_**, showing the two molecules present in the unit cell. Solvent molecules and hydrogen atoms are omitted for clarity. Within the crystal structure, also many intermolecular interactions are present between the molecules of **(−)‐Zr(1)_2_**, in particular offset π‐π stacking interactions between the xylylene sidewalls, which have been observed frequently in X‐ray structures of other molecular clips based on glycoluril.^[56]^ Within the unit cell, it is visible that the phenyl groups of the glycoluril framework act as a small molecular clip, which clamps one of the *meso*‐phenyl groups of the porphyrin rings of another cage.

The absolute configurations of the enantiomers could be assigned on the basis of the X‐ray structure of the compound that eluted second from the column during the purification (CCDC 2023416), i. e. **(−)‐Zr(1)_2_**, which turned out to correspond to the (*M*)‐isomer (Figure 3). The obtained crystals of **(+)‐Zr(1)_2_** were of insufficient quality to perform an absolute structure determination. The unit cell of the crystal contains two molecules of **(−)‐Zr(1)_2_**. The overall coordination geometry around the zirconium center is square antiprismatic. The porphyrin cages of these two molecules are rotated relative to each other by angles of 47° in one double cage molecule in the unit cell, and 58° in the other molecule, which is somewhat larger than the angle of 37° reported for Zr(TPP)_2_.[^52^, ^54^] The average distance between the mean planes through the porphyrin nitrogen atoms is 2.56 Å, which is the same distance as was found in the X‐ray structure of Zr(TPP)_2_.[^52^, ^54^] The average distance between the mean porphyrin planes (plane through the 24 atoms of the porphyrin ring) is 3.33 Å, which is close to the reported value of 3.28 Å for Zr(TPP)_2_.[^52^, ^54^] The dihedral angle between the pyrrole rings and the mean porphyrin planes is an indication of the distortion of the porphyrin. For Zr(TPP)_2_, these angles ranged from 9.9° to 24.2°,[^52^, ^54^] while for **(−)‐Zr(1)_2_** they vary from 11.2° to 26.2°, indicating that the porphyrins in **(−)‐Zr(1)_2_** are slightly more distorted than those in Zr(TPP)_2_. The average distance between the protons H‐30 (see Figure 5 for proton numbering) on opposite xylylene sidewalls at the same portal of the two cavities is 6.40 Å, which is similar to the distance in the X‐ray structure of these protons in the parent cage compound **H_2_1**, i. e., 6.34 Å.^[55]^ The distance from the carbonyl groups at the bottom of the cavity to the mean porphyrin plane is 8.67 Å in **H_2_1** and on average 9.03 Å in **(−)‐Zr(1)_2_**, indicating a slight elongation of the cavities in the latter complex, presumably due to the distortion of the porphyrin planes.

### Self‐assembly at a surface

**Figure 4:**
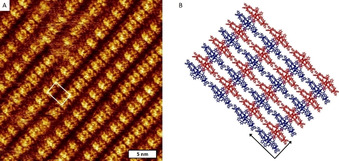
(A) STM image of a self‐assembled monolayer of **(±)‐Zr(1)_2_** at the interface of HOPG and 1‐phenyloctane; the unit cell is indicated in white; V_bias_=−570 mV, I_set_=3 pA. (B) Molecular model of a possible composition of the self‐assembled monolayer, in which the red and blue structures represent the two different enantiomers of **Zr(1)_2_, (+)‐Zr(1)_2_** and **(−)‐Zr(1)_2_**, respectively. The unit cell is indicated by the black arrows.

The solubility of **(±)‐Zr(1)_2_** in most common solvents and solvent mixtures turned out to be quite low, i. e. <1 mg/mL, except in dichloromethane, in which the compound showed a relatively high solubility of ≈5 mg/mL. We rationalized that in the poor solvents **(±)‐Zr(1)_2_** was prone to aggregation. To further investigate the ability of **(±)‐Zr(1)_2_** to self‐assemble, droplets of solutions of racemic and enantiopure **Zr(1)_2_** in 1‐phenyloctane (c≈10^−4^ M) were brought onto a freshly cleaved surface of highly oriented pyrolytic graphite (HOPG) in a scanning tunneling microscope (STM) operating at a solid/liquid interface. The pure enantiomers failed to form stable monolayers, but for the racemate extended domains of self‐assembled lamellar arrays, containing near‐rectangular features with dimensions close to that of a **(±)‐Zr(1)_2_** molecule were observed (Figure 4A). The slightly oblique unit cell appears to contain two molecules of **(±)‐Zr(1)_2_** and is defined by vectors with dimensions a=3.5±0.2 nm and b=2.4±0.15 nm, under an angle of 93±2°. Although the near‐rectangular features contain sub‐molecular features, the structure of **(±)‐Zr(1)_2_** cannot be directly identified in them. This is not surprising, given the fact that the adsorbed double cages are large 3D architectures of which the aromatic and aliphatic parts are superimposed from the point of view of the STM probe. Nevertheless, two types of roughly equally sized near‐rectangular features can be discerned, differing in brightness, which alternate along each of the lamellar arrays. The observed difference in brightness may be caused by (*i*) the alternating adsorption of the (*M*)‐isomer and the (*P*)‐isomer of **(±)‐Zr(1)_2_**, (*ii*) two different adsorption geometries of the double cage molecules (i. e., independent of the enantiomorph), or (*iii*) a combination of these two possibilities. Unfortunately, the complex appearance of the rectangular features prohibited identification of either the isomer identity or the cage orientation. A molecular model was constructed assuming the possibility that both enantiomers of **Zr(1)_2_** adsorb on the surface, i. e. as a 2D racemate (Figure 4B). Within a lamellar array, the 2D assembly of **(±)‐Zr(1)_2_** (the two enantiomers are indicated in red and blue, respectively) is stabilized by intermolecular π‐π stacking interactions between the *meso*‐phenyl rings of adjacent porphyrin planes, while between the lamellae the phenyl rings of the glycoluril frameworks of the double cages are within Van der Waals contact distance. As mentioned above, a stable monolayer of the racemate formed readily at the solid/liquid interface, while multiple attempts to construct and image stable monolayers of the pure enantiomers failed. This might imply that the presence of both enantiomers of the double cage compounded is required for the formation of a stable layer. However, since layer formation at a solid/liquid interface can be quite unpredictable and is dependent on a variety of conditions, such a conclusion may be premature and requires additional research.

### NMR characterization

**Figure 5:**
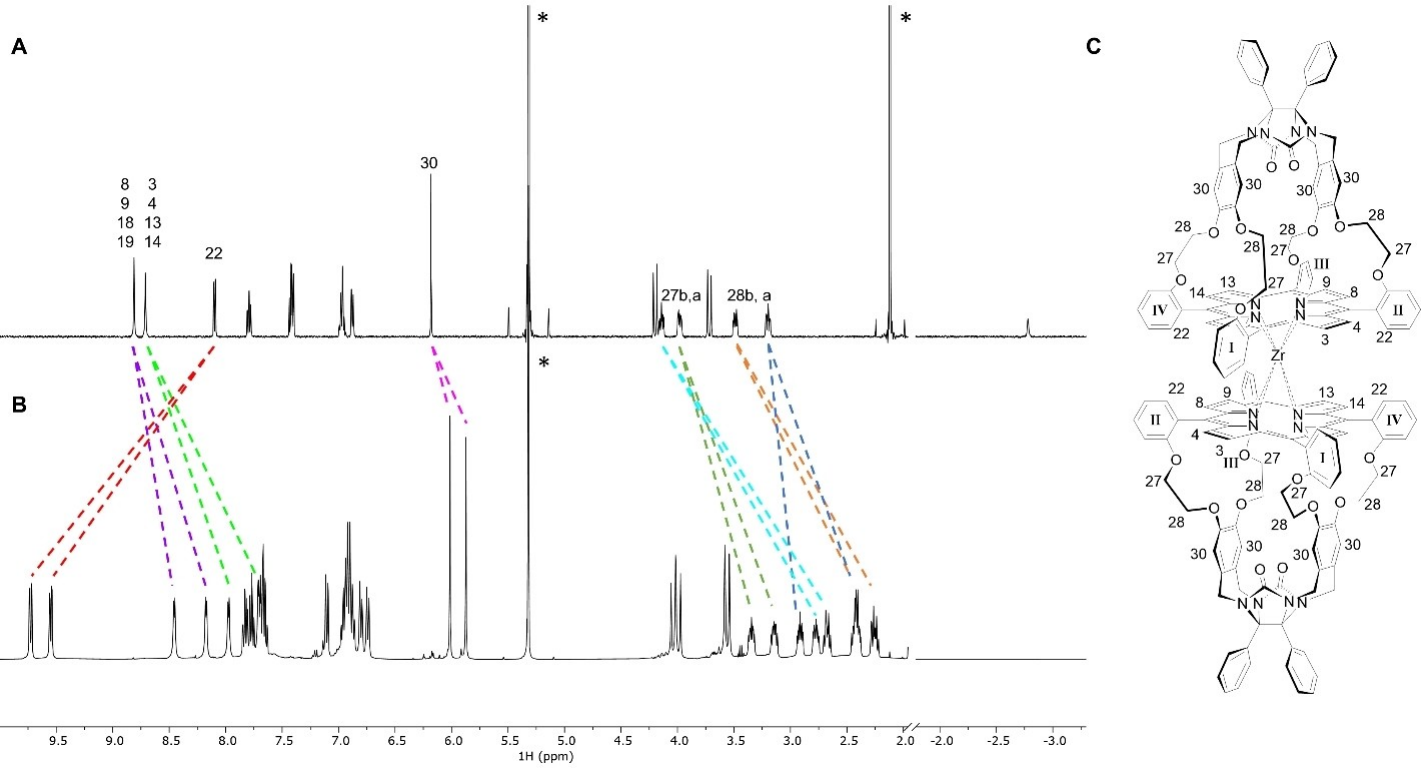
^1^H NMR spectra of (A) **H_2_1** (500 MHz) and (B) **(±)‐Zr(1)_2_** (400 MHz) in CD_2_Cl_2_; the dashed lines indicate the shifts of selected proton signals, and the asterisks the residual solvent peaks. (C) Atom numbering (selected) of **(±)‐Zr(1)_2_**; the signs I–IV indicate the four quadrants in which each of the porphyrin cages is divided for assignment of the NMR signals; see Figure S1 for a full atom numbering.

The structure of **(±)‐Zr(1)_2_** in solution was investigated by means of 1D and 2D NMR spectroscopy, which allowed the assignment of all proton and carbon signals. Compared to the ^1^H NMR spectrum of **H_2_1**, the spectrum of **(±)‐Zr(1)_2_** (Figure 5) is more complex, since asymmetry is induced into this compound upon coordination of the two cages to the zirconium(IV) center. This induced asymmetry causes a doubling of nearly all proton signals of the cages, making the protons located at opposite cage ‘quadrants’ (I and III, and II and IV, see Figure 5) chemically equivalent. In addition to this doubling of signals, the majority of them shifted upfield compared to the signals in **H_2_1** (Table S1). The porphyrin β‐pyrrole protons were assigned based on the observed shifts of their signals upon the inclusion of viologen guests **G1** inside the cavities (*vide infra*): the signals of protons H‐3, H‐4, H‐13, and H‐14, which are located directly above the large cavity openings in which the guest binds, experience significant downfield shifts upon binding, while the signals of protons H‐8, H‐9, H‐18, and H‐19 barely shift (see Figure 11 and Table 3). The two sets of protons display ROE contacts with different *meso*‐phenyl *ortho‐*protons H‐22 of the porphyrin, which allowed the assignment of each quadrant of the porphyrin cage. Compared to their resonances in **H_2_1**, the signals of protons H‐22 of **(±)‐Zr(1)_2_** are shifted significantly downfield (+1.63 and +1.45 ppm compared to **H_2_1**) as a result of their location in the deshielding zone of the close‐by porphyrin of the second cage in the sandwich complex. The distortion in the porphyrin planes results in a decrease in their aromaticity, causing an upfield shift of the β‐pyrrole signals compared to **H_2_1**. Simultaneously, the X‐ray structure shows that the *meso*‐phenyl rings are slightly tilted with respect to the porphyrin plane, which is likely the result of the distortion of the latter plane, combined with the steric hindrance imposed by the other, nearby porphyrin ring. This tilting in turn seems to cause the oxyethylene protons H‐27 and H‐28 to rotate to the inside of the cage and into the shielding zone of the porphyrin, resulting in strong upfield shifts (up to −1.32 ppm) of their proton signals in the NMR spectrum compared to those of **H_2_1**.

### Electrochemical properties

**Figure 6:**
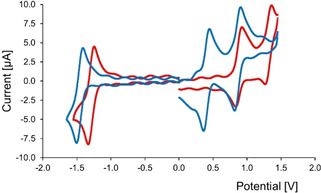
Cyclic voltammograms of **H_2_1** (red traces) and **(±)‐Zr(1)_2_** (blue traces) in CH_2_Cl_2_ with NBu_4_PF_6_ (0.1 M), scan rate 0.1 V/s, carbon electrode, platinum electrode, and a Ag KCl (3 M)/AgCl reference electrode.

The redox properties of **(±)‐Zr(1)_2_** were investigated by means of cyclic voltammetry, and compared to the properties of other zirconiumporphyrin sandwich complexes and the non‐metallated parent porphyrin cage **H_2_1** (Figure 6, Table 1). In CH_2_Cl_2_, both **(±)‐Zr(1)_2_** and **H_2_1** display two reversible oxidations and one reversible reduction of the porphyrin ligand. For both **(±)‐Zr(1 )_2_** and **H_2_1**, no second reduction was observed due to the limits of the combination of solvent and electrolyte. Compared to **H_2_1**, **(±)‐Zr(1)_2_** is easier to oxidize and more difficult to reduce, which indicates that both the HOMO and the LUMO of **(±)‐Zr(1)_2_** are higher in energy than those of **H_2_1**. The same trend is visible for TPP and Zr(TPP)_2_.^[54]^ This increase in energy observed for the porphyrins in zirconium(IV) sandwich complexes might be explained by the distortion in the porphyrin plane (visible in the crystal structure in Figure 3), which causes a reduction of the aromaticity of the porphyrin. Decreased oxidation potentials have been found before for other sandwich complexes and their corresponding monoporphyrinates.[^54^, ^57^] For Zr(TPP)_2_ it was hypothesized that the energy rise of the HOMO is the result of π‐π stacking interactions between the porphyrin planes.^[54]^

**Table 1:** Redox potentials (in [V]) of **H_2_1** and **(±)‐Zr(1)_2_**, and for comparison those of TPP^[58]^ and Zr(TPP)_2_.[^52^, ^54^, ^59^, ^60^] Solvent/supporting electrolyte: CH_2_Cl_2_/NBu_4_PF_6_ referenced to ferrocene (E_1/2_=0.497 mV). ΔE_2,3_ is the difference between the potentials for the first oxidation and the first reduction.

Compound	E_1_	E_2_	E_3_	E_4_	ΔE_2,3_
H_2_1	0.82	0.39	−1.79	–	2.18
(±)‐**Zr(1)_2_**	0.36	−0.10	−1.96	–	1.86
**TPP**	0.82	0.52	−1.64	−1.93	2.16
**Zr(TPP)_2_**	0.55	0.12	−1.73	−2.09	1.85

Compound **(±)‐Zr(1**)_**2**_ is easier to oxidize and more difficult to reduce compared to Zr(TPP)_2_, which is likely the result of the more electron‐rich porphyrin because of the alkoxy substituents at the *meso*‐phenyl groups. A similar redox behaviour was reported for a porphyrin with *meso*‐*o*‐methoxyphenyl substituents (T(*o*‐OMe)PP), of which the oxidation potentials are ∼0.1 V lower than those of TPP.^[61]^ Another consequence of the presence of the cage moieties is that the double cage sandwich complex is less flexible than the unfunctionalized sandwich complex, which may induce more strain in the porphyrin rings, causing their aromaticity to be reduced.

### Chiroptical properties

**Figure 7:**
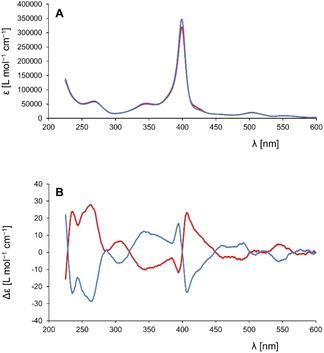
(A) UV‐vis and (B) ECD spectra of the two enantiomers of **Zr(1)_2_**. **(+)‐Zr(1)_2_** (P‐isomer, red traces) c=0.0555 mM in CH_2_Cl_2_, **(−)‐Zr(1)_2_** (M‐isomer, blue traces) c=0.0562 mM in CH_2_Cl_2_.

**Figure 8:**
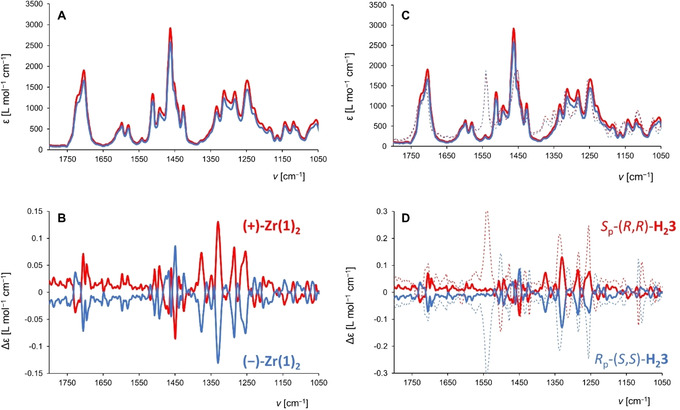
(A) IR and (B) VCD spectra of the two enantiomers of **Zr(1)_2_**. Red traces **(+)‐Zr(1)_2_**, blue traces **(−)‐Zr(1)_2_**; c=0.15–0.18 mM in CD_2_Cl_2_. (C) IR and (D) VCD spectra of the two enantiomers of **Zr(1)_2_** (solid traces) overlaid with the spectra^[63]^ of the two enantiomers of porphyrin cage **H_2_3** (dotted traces).

Compared to the porphyrin Soret band in the UV‐vis spectrum of **H_2_1** in CH_2_Cl_2_, the Soret band of **(±)‐Zr(1)**_2_ is blue‐shifted by ∼20 nm to 398 nm (Figure 7A). This increase in energy of the porphyrin π‐π* transition is likely the result of the decrease in conjugation due to the distortion of the porphyrin planes. The mirror‐like ECD and VCD spectra (Figure 7B and Figure 8B, respectively) of the two compounds separated by chiral HPLC revealed that they are enantiomers, as expected. The shape of the Soret band is correlated to a strong exciton coupling between the two porphyrin planes. The ECD spectra display a right‐handed couplet at 398 nm for the first eluted enantiomer **(+)‐Zr(1)_2_**, which corresponds to a clockwise orientation of the porphyrin planes, and thus this enantiomer is the (*P*)‐isomer.^[62]^ The second eluted enantiomer, **(−)‐Zr(1)_2_**_,_ displays a left‐handed couplet at 398 nm, which is in agreement with an anti‐clockwise orientation (*M*) of the porphyrin rings, which in turn corresponds to the X‐ray crystallography data. The Q bands around 500 and 550 nm are also weakly CD‐active, and other strong CD signals are present in the high‐energy region between 250 and 350 nm.

The VCD spectra of the enantiomers **(+)‐Zr(1)_2_** and **(−)‐Zr(1)_2_** in CD_2_Cl_2_ displayed many vibrations of the receptor‐parts of the molecules, implying that the chiral environment around the zirconium(IV) center is propagated to the entire cage structure (Figure 8A−B). Strikingly, these sandwich complexes display VCD fingerprints that are very similar to the recently reported single porphyrin cage compound **H_2_3** (Figure 1), in which the chirality was introduced into the molecule in a completely different manner, *i. e*. by substituting each of the xylylene side‐walls with a nitro‐group.^[63]^ The VCD spectra of **(+)‐Zr(1)_2_** and *S*_p_‐(*R*,*R*)‐**H_2_3** both exhibit four characteristic positive bands at 1253, 1285, 1330/1336 and 1375 cm^−1^, and a negative‐to‐positive couplet at 1705–1728 cm^−1^ (Figure 8C−D) and are rather intense (dissymmetry factors g_abs_ around 1.2×10^−1^). The positive band at 1336 cm^−1^ for *S*_p_‐(*R*,*R*)‐**H_2_3** is dominated by vibrational modes of deformations of the porphyrin part. In **(+)‐Zr(1)_2_**, the formation of bonds with the zirconium(IV) center leads to a red shift of this band of 6 cm^−1^. The characteristics of this band (sign, shape and intensity) can be related to the concave geometry adopted by the porphyrin in both molecules *S*_p_‐(*R*,*R*)‐**H_2_3** and **(+)‐Zr(1)_2_**. The complexation of the cage with zirconium(IV) significantly emphasizes this concavity. Similarly, the sign and intensity of the couplet, which results from the mixing of the two C=O stretching modes, depends on the relative asymmetry of these functional groups in the most abundant conformations of *S*_p_‐(*R*,*R*)‐**H_2_3** and **(+)‐Zr(1)_2_**.[^64^, ^65^] This phenomenon represents a rare example where the VCD spectra reflect a molecule with conformational chirality (atropisomerism) and it confirms the usefulness of VCD spectroscopy for characterizing porphyrin derivatives, an aspect that was not emphasized before.

Upon irradiation of the Soret band of **(±)‐Zr(1)_2_**_,_ an intense band at 658 nm in the emission spectrum appeared (Figure S9). This emission was determined to be fluorescence based on lifetime measurements (Figure S10). About 60 % of the fluorescence was quenched when oxygen was bubbled through the sample for 3 minutes (Figure S9).

### Host‐guest binding studies

**Figure 9:**
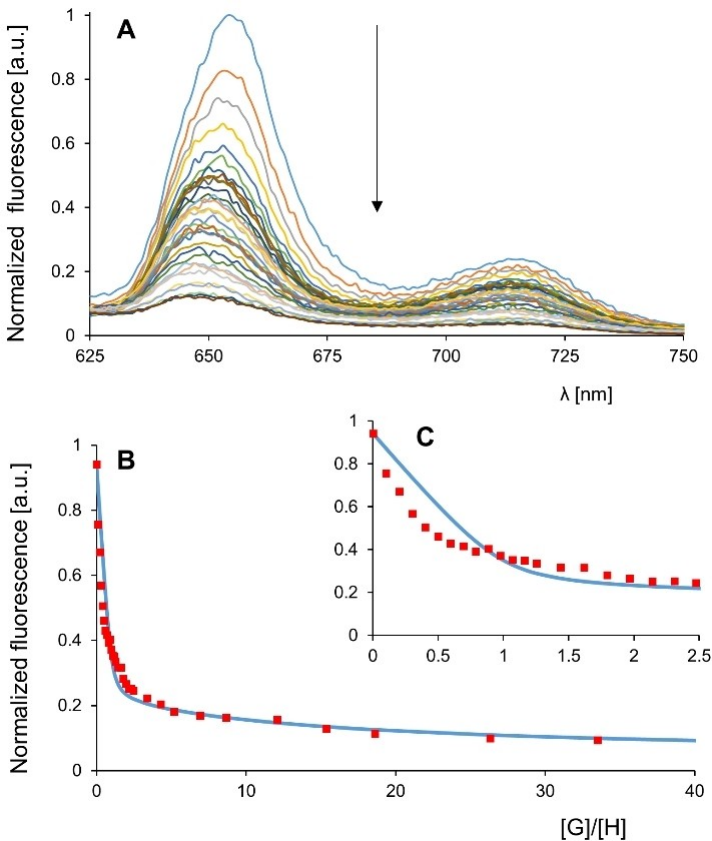
(**A**) Fluorescence spectra recorded during the titration of **(±)‐Zr(1)_2_** at 3.0 μM in CH_2_Cl_2_/CH_3_CN (1 : 1, v/v) with **G1**. (**B**) Titration curve at 653 nm and fit assuming a 1 : 2 host‐guest binding stoichiometry. (C) Zoom‐in of the first part of the titration curve.

**Figure 10:**
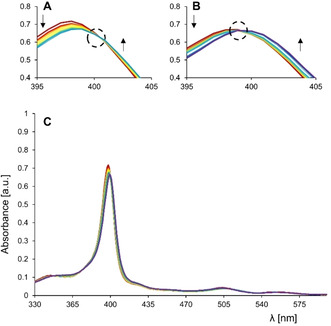
UV‐vis spectra during the titration of **(±)‐Zr(1)_2_** with **G1** in CH_2_Cl_2_/CH_3_CN (1 : 1, v/v) with host concentration remaining constant at 2.0 μM and **G1** ranging from 0 to 1370 equivalents. Isosbestic points are indicated by the black dotted circles. (**A**) Spectra taken in the first part of the titration (0–2 equiv. of **G1** present) showing a clear isosbestic point due to the conversion of free host to a 1 : 1 complex. (**B**) Spectra taken in the second part of the titration (2–1370 equiv. of **G1** present) showing a second isosbestic point, suggesting the transition of the 1 : 1 to the 1 : 2 complex. The arrows indicate the spectral changes upon the addition of increasing amounts of **G1**. (**C**) Full set of UV‐vis spectra of the titration.

To investigate the binding strength between **Zr(1)_2_** and methyl viologen (*N*,*N*‐dimethyl‐4,4’‐bipyridinium dihexafluorophosphate) (**G1**), and to study cooperative effects between the binding of the first and second guest, a fluorescence titration was carried out (Figure 9A−C, Table 2). As expected, the addition of increasing amounts of **G1** to a solution of **(±)‐Zr(1)_2_** 3.0 μM in CH_2_Cl_2_/CH_3_CN, 1 : 1 (v/v) caused quenching of the fluorescence (a solvent mixture was employed since neither the host nor the guest are sufficiently soluble in a single solvent). Fitting of the titration data to a 1 : 2 host‐guest stoichiometry revealed that the second viologen guest is bound almost 3 orders of magnitude weaker than the first one (*K*_1_=1.1×10^7^ M^−1^, *K*_2_=1.9×10^4^ M^−1^), indicating a strongly negative cooperative effect with an α‐value (4 *K*_2_/*K*_1_) of 0.0076. It should be noted that the first part of the titration curve could not be fitted completely, leading to somewhat higher errors. Interestingly, the binding of the viologen guest resulted not only in the fluorescence quenching of the adjacent porphyrin, but also in a partly fluorescence quenching of the porphyrin on the opposite side of the zirconium center. This can be concluded from the fact that the fluorescence at 1 equivalent of added **G1** is not 50 % but 35 %, i. e. 65 % is quenched (Figure 9C). This means that also the second porphyrin is within the quenching distance of the guest. The host‐guest binding properties were also evaluated by UV‐vis titration experiments (Figure 10 and Figure S13, Tables S13–19), which confirmed the 1 : 2 host‐guest complex formation with negative cooperativity. Taking into account the larger errors in the fits of the fluorescence titrations, rather similar values were obtained for the association constants measured by fluorescence and by UV‐Vis titrations (*K*_1_=2.1×10^6^ M^−1^, *K*_2_=5.0×10^3^ M^−1^, α‐value=0.0096). During the first part of the UV‐vis titration (in the presence of 0 to ∼2 equivalents of **G1**) a clear isosbestic point was observed at 400 nm (Figure 10A), suggesting the conversion of the free host to its 1 : 1 host‐guest complex without the significant formation of a 1 : 2 complex. When the amounts of guest were increased, the isosbestic point at 400 nm disappeared while a new one, albeit less prominent, appeared at ∼398 nm (Figure 10B). The latter isosbestic point may indicate the conversion of the 1 : 1 complex to the 1 : 2 complex. The host‐guest binding could also be monitored by CD spectroscopy (Figures S11‐13, Tables S10–12). Complexation between **(+)‐Zr(1)_2_** and **G1** induced the emergence of a new CD signal between 350 and 390 nm, but due to a poor signal to noise ratio the determination of the *K*‐values in a titration experiment turned out to be rather unreliable.

**Table 2:** Association constants (*K*) and binding free energies (Δ*G*) for complexes of porphyrin cage compounds with various guests. The cooperativity factor is defined as α=4 *K*_2_/*K_1_*. ΔΔ*G*_allost_ is defined as Δ*G*_2_–Δ*G*_1_.

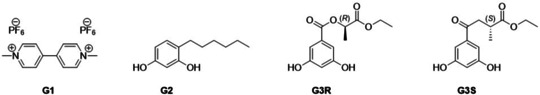							
Guest	Host	*K*_1_ [M^−1^]	Δ*G*_1_ [kJ mol^−1^]	*K*_2_ [M^−1^]	Δ*G*_2_ [kJ mol^−1^]	ΔΔ*G*_allost_ [kJ mol^−1^]	α
**G1**	**H_2_2** ^[50][a]^	7×10^7^	−43	5×10^4^	−28	15	0.003
	(±)‐**Zr(1)_2_** ^[b]^	1.11±0.42×10^7^	−40.2±0.9	1.87±0.20×10^4^	−24.4±0.3	15.8±1.2	0.0076±0.0032
	(±)‐**Zr(1)_2_** ^[c]^	2.11±0.47×10^6^	−36.2±0.5	5.00±1.08×10^3^	−21.1±0.6	15.1±1.1	0.0096±0.0023
**G2**	**H_2_1** ^[d]^	3.73±0.84×10^2^	−14.7±0.5	–	–	–	–
	(±)‐**Zr(1)_2_** ^[d]^	4.10±1.06×10^3^	−20.6±0.7	5.45±1.97×10^2^	−15.5±0.8	5.1±1.5	0.53±0.09
**G3R**	(+)‐**Zr(1)_2_** ^[d]^	9.80±3.30×10^2^	−17.0±1.0	1.44±0.60×10^2^	−12.1±1.2	5.1±2.2	0.58±0.12
	(−)‐**Zr(1)_2_** ^[d]^	1.24±0.20×10^3^	17.7±0.4	1.78±0.48×10^2^	−12.8±0.7	4.9±1.1	0.57±0.10
**G3S**	(+)‐**Zr(1)_2_** ^[d]^	1.10±0.26×10^3^	−17.3±0.6	1.58±0.53×10^2^	−12.4±0.9	4.9±1.5	0.57±0.10
	(−)‐**Zr(1)_2_** ^[d]^	1.19±0.24×10^3^	−17.5±0.5	1.68±0.57×10^2^	−12.6±0.9	4.9±1.4	0.56±0.11

[a] Determined by fluorescence titrations in CHCl_3_/CH_3_CN, 1 : 1 (v/v). [b] Determined by fluorescence titrations in CH_2_Cl_2_/CH_3_CN, 1 : 1, v/v. [c] Determined by UV‐vis titrations in CH_2_Cl_2_/CH_3_CN, 1 : 1, v/v. [d] Determined by ^1^H NMR titrations in CD_2_Cl_2_.

**Figure 11:**
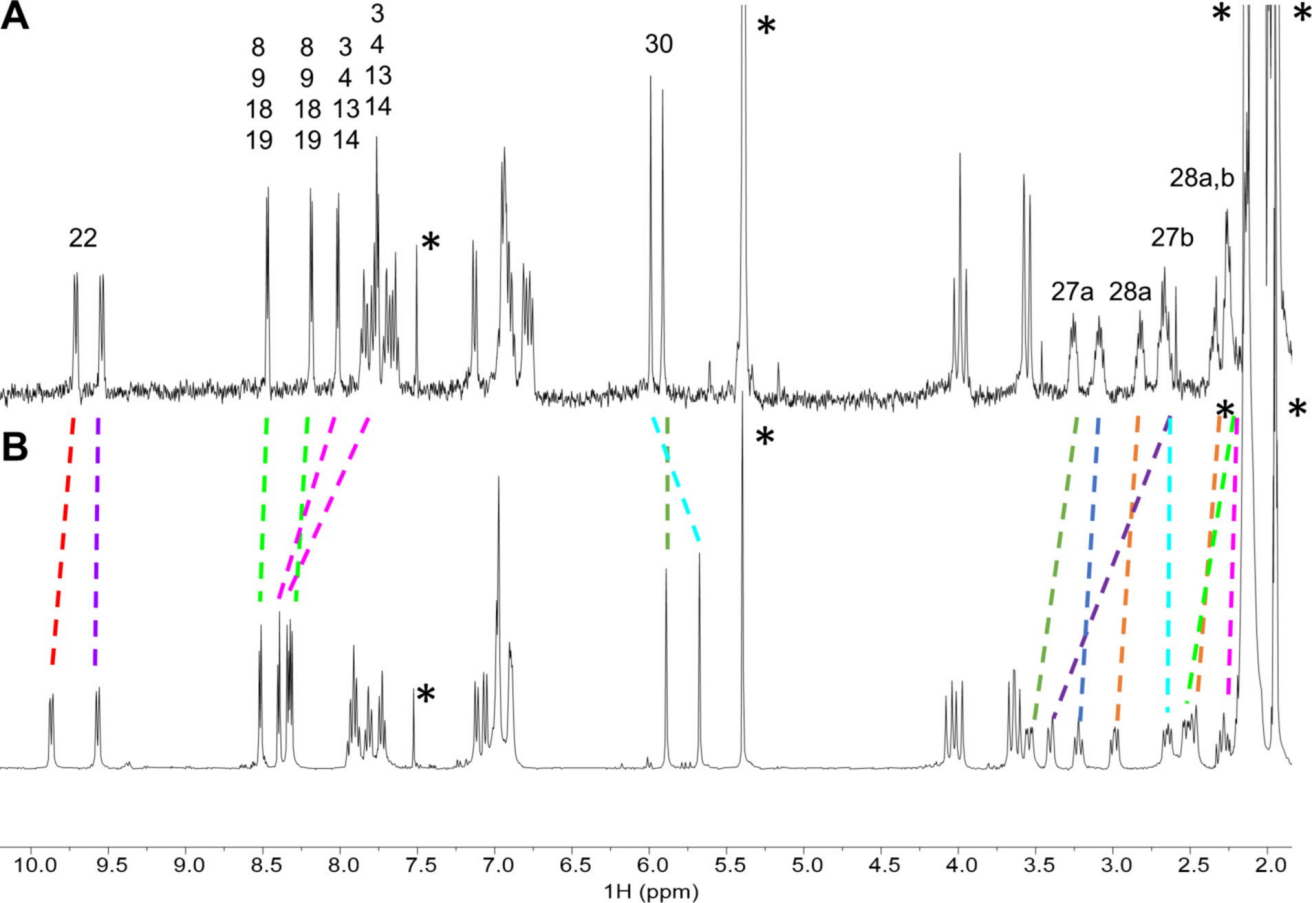
^1^H NMR spectra (400 MHz) of (A) **(±)‐Zr(1)_2_** and (B) its 1 : 2 host‐guest complex with **G1** in CD_2_Cl_2_/CD_3_CN (1 : 1, v/v); shifts of selected proton signals are indicated with dashed lines; see Figure 5C for atom labeling. The asterisks indicate residual solvent peaks.

The ^1^H NMR spectrum of the 1 : 2 complex between **(±)‐Zr(1)_2_** and **G1** in CD_2_Cl_2_/CD_3_CN 1 : 1 (v/v) indicated a similar binding geometry of the viologen guests as in the case of **H_2_1**. The downfield complexation induced shifts (CIS) of the signals of β‐pyrrole protons H‐3, H‐4, H‐13, H‐14 and the upfield shifts of the cavity side‐wall protons H‐30 upon the binding of **G1** (Figure 11, Table 3) indicate that the guests bind with their aromatic rings perpendicular to the porphyrin and parallel to the xylylene side‐walls.^[2]^ Remarkably, in contrast to the considerable upfield shifts that were observed for the proton signals of the ethyleneoxy spacers upon the binding of **G1** in **H_2_1**,^[31]^ for **(±)‐Zr(1)_2_** significant downfield shifts were observed for several of the signals of protons H‐27 upon binding of the guests. We attribute these shifts to a structural reorganization of the host in which the ethyleneoxy spacers move out of the shielding zone of the porphyrin by rotating outwards, thereby generating space in the cavities to accommodate the large viologen guests (induced‐fit). All proton signals of the guests were very broad, which is attributed to fast exchange between bound and unbound species. Compared to the complexation of **G1** in **H_2_1**,^[31]^ the first guest in **(±)‐Zr(1)_2_** is bound significantly stronger, and the second one significantly weaker. The difference in the Gibbs free energy of binding of **G1** in the two cavities of **(±)‐Zr(1)_2_** (ΔΔ*G*_allost_) is 15.1 kJ mol^−1^.

**Table 3:** ^1^H NMR chemical shifts [δ, ppm] and complexation induced shifts [CIS, δ, ppm] of selected proton signals of (±)‐**Zr(1)_2_** and its 1 : 2 host‐guest complexes with **G1** and **G2**; see Figure 5C for atom labeling.

Proton	(±)‐**Zr(1)_2_** ^[a]^	(±)‐**Zr(1)_2_** ⋅ **(G1)_2_** ^[a]^		(±)‐**Zr(1)_2_** ^[b]^	(±)‐**Zr(1)_2_** ⋅ **(G2)_2_** ^[b]^	
	δ	δ	CIS	δ	δ	CIS
3,4,13,14	8.02, 7.76	8.32, 8.26	+0.30, +0.50	7.97, 7.71	8.08, 7.89	+0.11, +0.18^[c]^
27a	3.26, 3.09	3.46, 3.15	+0.20, +0.06	3.34, 3.14	2.83, 2.62	−0.51, −0.52
27b	2.67	2.57, 3.33	−0.10, +0.66	2.77, 2.67	2.22, 2.05	−0.55, −0.62
28a	2.82, 2.34	2.91, 2.44	+0.09, +0.10	2.91, 2.43	2.25, 2.07	−0.66, −0.36^[c]^
28b	2.26	2.39, 2.20	+0.13, −0.06	2.41, 2.25	2.07,1.60	−0.36,^[c]^ −0.65^[c]^
30	5.99, 5.91	5.60, 5.81	−0.39, −0.10	6.01, 5.87	5.94, 5.91	−0.07, +0.04

[a] In CD_2_Cl_2_/CD_3_CN 1 : 1 (v/v). [b] In CD_2_Cl_2_. [c] Not used in the fitting due to the difficulty to distinguish signals during the titration.

**Figure 12:**
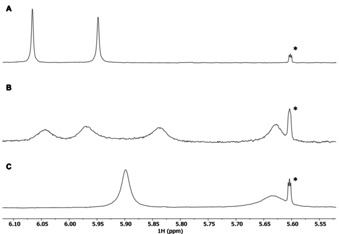
^1^H NMR spectra (500 MHz, CD_2_Cl_2_/CD_3_CN (1 : 1, v/v), 245 K) of the region of the signals of side‐wall protons H‐30 of solutions containing (A) **(±)‐Zr(1)_2_**, (B) a 1 : 1 mixture of **(±)‐Zr(1)_2_** and **G1**, and (C) a 1 : 2 mixture of **(±)‐Zr(1)_2_** and **G1**. [**(±)‐Zr(1)_2_**]=0.24 mM. See supporting information Figure S29 for the full spectra. The signal indicated by the asterisk is a ^13^C satellite of the CDHCl_2_ solvent peak.

In an effort to confirm the large difference in the association constants *K*_1_ and *K*_2_, solutions of **(±)‐Zr(1)_2_** and of this host in the presence of one and two equivalents of **G1** were investigated by ^1^H NMR spectroscopy at 245 K. This low temperature was chosen in order to induce slow host‐guest exchange in the complexes. The spectra of **(±)‐Zr(1)_2_** and of the 1 : 2 host‐guest mixture resembled the room temperature spectra, while the spectrum of the 1 : 1 host‐guest mixture showed a doubling of many of the proton signals (Figure 12 and Figure S29). In particular the doubling of the number of side‐wall proton signals H‐30 is illustrative (Figure 12B) and can be attributed to symmetry breaking within the host, which can be expected when only one single **G1** guest binds in one of the cavities of **(±)‐Zr(1)_2_**. As a result, the two cavities are no longer equivalent, resulting in different chemical shifts for previously identical protons. The four signals of the side‐wall protons are located at positions that are distinctly different from the positions of the side‐wall proton signals in the free host and the 1 : 2 host‐guest complex. This is further evidence for the presence of predominantly a 1 : 1 complex when one equivalent of **G1** is added. The upfield shift of the signals of proton H‐30 indicates that also in the 1 : 1 complex the guest binds with its aromatic rings parallel to the xylylene side‐walls.^[2]^ The addition of the second equivalent of guest restores the symmetry of the host. Unfortunately, due to the poor solubility of **(±)‐Zr(1)_2_** (0.24 mM) at low temperature we were unable to employ 2D NMR techniques to further characterize the precise geometry of the 1 : 1 complex.

The association constants for the binding of **G1** in **(±)‐Zr(1)_2_** shown in Table 2 are quite similar to those found for the binding of the same guest in our previously reported double porphyrin cage compound **H_2_2** (Figure 1), in which the two receptor cavities are separated by a single porphyrin.^[34]^ For the latter compound, binding of a viologen in the first cavity resulted in a pinching of the second, empty cavity, thereby causing an allosteric effect which made the cavity an inferior host for the second viologen. A similar allosteric mechanism may be operative in the case of **(±)‐Zr(1)_2_**, since the two receptor cavities are structurally connected via their coordination to the zirconium center.

**Figure 13:**
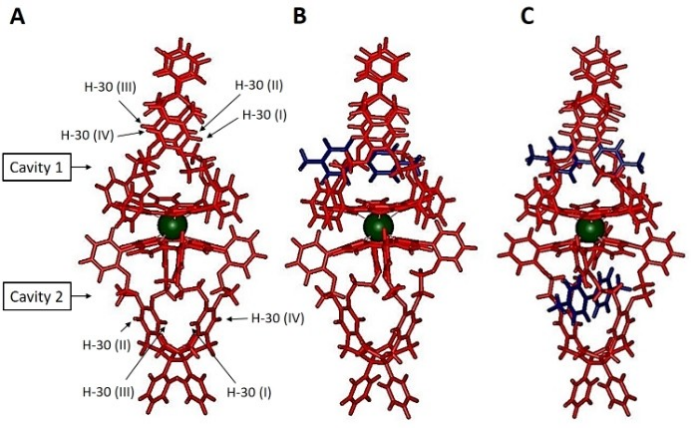
Calculated molecular models of (A) **(−)‐Zr(1)_2_**, (B) its 1 : 1 complex with **G1** (in blue), and (C) its 1 : 2 complex with **G1**. The calculated distances between the various side‐wall protons H‐30 are summarized in Table 4.

To investigate this possibility, we calculated the structures of the free host **(−)‐Zr(1)_2_** and its 1 : 1 and 1 : 2 host‐guest complexes with **G1** by molecular modelling (Spartan ‘14^TM^, semi‐empirical, PM3). The calculated energy‐minimized structures (Figure 13) show significant variations in the cavity widths of **(−)‐Zr(1)_2_**, depending on the presence or absence of guest molecules. As an indication of these widths, the distances between protons H‐30 of the two cavities at opposite xylylene side‐walls are summarized in Table 4. The calculations reveal that when the first viologen guest binds in cavity 1, the latter widens when compared to its width in the absence of the guest. At the same time, the width of cavity 2 decreases. When the second viologen guest binds, cavity 2 retains its narrow size, while cavity 1 further narrows. The observation that the second guest needs to bind in a relatively narrow cavity is in line with the observed negative cooperativity of guest binding. Since this narrowing is apparently caused by a structural rearrangement throughout the whole double cage complex, induced by the binding of the first guest, this negative cooperativity can indeed be attributed to allostery.

**Table 4:** Distances [Å] between side‐wall protons H‐30 calculated by molecular modeling of **(−)‐Zr(1)_2_** and its 1 : 1 and 1 : 2 complexes with **G1** and **G2**. See Figure 11 for atom labeling.

Host or host‐guest complex	Cavity 1		Cavity 2	
	H‐30 (I)–H‐30 (II)	H‐30 (III)–H‐30 (IV)	H‐30 (I)–H‐30 (II)	H‐30 (III)–H‐30 (IV)
(−)‐**Zr(1)_2_**	6.73	6.93	6.49	6.76
(−)‐**Zr(1)_2_** ⋅ **G1**	7.63	6.98	6.55	6.55
(−)‐**Zr(1)_2_** ⋅ **(G1)_2_**	6.73	6.71	6.49	6.38
(−)‐**Zr(1)_2_** ⋅ **G2**	6.91	6.94	6.54	6.38
(−)‐**Zr(1)_2_** ⋅ **(G2)_2_**	6.85	6.95	6.92	7.07

In addition to viologens, resorcinol (1,3‐dihydroxybenzene) derivatives are also suitable guests for the porphyrin cage compounds.^[31]^ They generally bind by means of hydrogen bonding interactions between their hydroxyl groups and the urea carbonyl oxygen atoms of the host, while their aromatic rings interact via π**‐**π stacking with the xylylene side‐walls of the host and with the porphyrin plane.^[66]^ The binding of resorcinol‐derived guest molecules **G2**, **G3R**, and **G3S** in the cavities of **(±)‐Zr(1)_2_** was investigated with the help of ^1^H NMR titrations. The titration curves for the binding of **G2** in **(±)‐Zr(1)_2_** could only be fitted to a binding equilibrium assuming the formation of a 1 : 2 host‐guest complex, and provided *K*_1_ and *K*_2_‐values which are summarized in Table 2. The CIS‐values obtained from the titrations (Table 3) indicate that the guests bind in the cavities of **(±)‐Zr(1)_2_** with their aromatic surface parallel to the xylylene side‐walls, thereby strongly shifting upfield the signals of particularly the ethyleneoxy protons H‐27 and H‐28. These upfield shifts indicate that, in contrast to what was observed for the binding of **G1**, the binding of the smaller guest **G2** does not lead to a significant movement of the ethyleneoxy spacers of **(±)‐Zr(1)_2_** out of the porphyrin shielding zone, and hence suggest that the host requires no or much less rearrangement during binding than in the case of **G1**. The proton signals of the guest were again broad due to fast exchange between bound and unbound species. Interestingly, the first **G2** guest was found to bind an order of magnitude stronger in **(±)‐Zr(1)_2_** than in **H_2_1**. This enhanced binding is attributed to the stretching of the cavities of the double cage complex. Since the aromatic ring of a resorcinol derivative bound in a glycoluril‐based clip molecule is clamped in a position slightly above the xylylene side‐walls of the cavity,^[66]^ this stretching and distortion of the cavities of **Zr(1)_2_** along the z‐axis, together with the distortion of the porphyrin plane, provides more space for the guest compared to the binding in **H_2_1**. For the binding of the two **G2** guests, an α‐value of 0.53 was observed, which is a much less prominent negative binding cooperativity than was found for the binding of **G1**. Molecular modeling calculations of the complexes of **(−)‐Zr(1)_2_** with **G2** (Figure S46) reveal that a similar, but less pronounced, allosteric effect is operative as in the complexes of the host with **G1**. Upon binding the first **G2** guest in cavity 1, the latter expands somewhat compared to the previously empty cavity, but significantly less than when it binds **G1** (Table 4). Simultaneously, cavity 2 narrows, thus disfavoring the binding of the second **G2** guest. Subsequent binding of the second guest widens cavity 2, while the width of cavity 1 remains nearly unaffected. The latter observation is different from what was found for the complexes of **(−)‐Zr(1)_2_** with **G1**, where the binding of the second guest leads to a more overall rearrangement of both cavity spaces. This requirement for more extensive rearrangement of the host is in line with the observed much more pronounced negative allosteric cooperativity for the binding of **G1** in **(±)‐Zr(1)_2_**.

In a final set of experiments we explored the potential of the enantiopure hosts **(+)‐Zr(1)_2_** and **(−)‐Zr(1)_2_** to enantioselectively bind chiral resorcinol derivatives **G3R** and **G3S** (Table 2). Within experimental error no chiral discrimination was found: all possible host‐guest combinations displayed similar *K*_1_ and *K*_2_‐values, which were all weaker than those observed for the binding of **G2** in **(±)‐Zr(1)_2_**. The lower binding affinities for guests **G3** are ascribed to their bulky 5‐substituents, which have more steric interactions with the porphyrin roof of the hosts. The CIS‐values obtained from the NMR titrations again show strong shielding of the ethyleneoxy protons H‐27 and H‐28 of the hosts upon guest binding, indicating that the host‐guest binding geometries are similar as those in the complex between **(±)‐Zr(1)_2_** and **G2** (Table S32). Because of their size, the ethoxycarbonyl substituents of **G3R** and **G3S** are probably directed to one of the cavity portals, which causes the methyl groups connected to the chiral center to point to one particular side of the cavity (Figure S14). As a result, one set of ethyleneoxy proton signals shifts more upfield than the other set. This effect is mirrored in the diasteromeric complexes and similar in the enantiomeric host‐guest complexes. Despite the relatively weak binding, still negative cooperativity was observed, with α‐values in the same range as those found for the host‐guest complexes between **(±)‐Zr(1)_2_** and **G2**.

## Conclusion

Double cage sandwich complex **Zr(1)_2_** was synthesized in a single step from **H_2_1** in a yield of 33 %. Its enantiomers have been resolved with excellent enantiomeric excesses and their absolute configurations could be assigned based on the crystal structure of **(−)‐Zr(1)_2_**, and were confirmed by circular dichroism spectroscopy. With the help of STM, the double cage complexes could be imaged at the sub‐molecular level in a self‐assembled monolayer of the racemate on a graphite surface, which was not possible with either of the pure enantiomers. VCD experiments of the enantiomers of **Zr(1)_2_** revealed that the chirality around the zirconium center is propagated throughout the whole cage structures. The VCD fingerprints are rather similar to the ones previously reported for the enantiomers of cage **H_2_3**. This represents a rare case in which the VCD spectra reflect a similarity between a molecule with axial chirality and a molecule with planar and point chirality. The double cage sandwich complexes formed 1 : 1 and 1 : 2 host‐guest complexes with viologen and 1,3‐dihydroxybenzene derivatives. No enantioselective binding of chiral 1,3‐dihydroxybenzene derivatives in the homochiral double cage complexes was observed. The binding of all guests showed negative cooperativity, i. e., the binding strength of the second guest was always weaker than that of the first one. The negative cooperativity is the result of allosteric effects, as the NMR data revealed a clear structural reorganization throughout the structure of both porphyrin cages upon the binding of the guests. Molecular modelling calculations confirmed these reorganizations and indicated that the binding of a guest in the first cavity of the double cage complex causes a narrowing of the second cavity, thereby disfavouring the binding of the second guest. The negative allosteric cooperativity was most pronounced for the binding of the large viologen guests, which require more structural reorganization of **Zr(1)_2_** than binding of the much smaller dihydroxybenzenes.

Future research will be directed towards providing **Zr(1)_2_** with a catalytic functionality, however as the zirconium center of **Zr(1)_2_** only serves as a non‐catalytic coordination site, we intend to provide the xylylene side‐walls of the double cage complex with oxidation catalysts (e. g. Mn‐porphyrins or Mn‐salens). These catalysts can modify a polymeric substrate that threads through one of the cages, while the properties of the substrate can be allosterically controlled, e. g. in terms of binding strength, threading speed, etc., via binding of an allosteric effector in the second cage.

Deposition Number 2023416 (for **(−)‐Zr(1)_2_**) contains the supplementary crystallographic data for this paper. These data are provided free of charge by the joint Cambridge Crystallographic Data Centre and Fachinformationszentrum Karlsruhe Access Structures service www.ccdc.cam.ac.uk/structures.

## Conflict of interest

The authors declare no conflict of interest.

## Supporting information

As a service to our authors and readers, this journal provides supporting information supplied by the authors. Such materials are peer reviewed and may be re‐organized for online delivery, but are not copy‐edited or typeset. Technical support issues arising from supporting information (other than missing files) should be addressed to the authors.

Supplementary
