# Supplementary material for: Allosteric Guest Binding in Chiral Zirconium(IV) Double Decker Porphyrin Cages
Source: European J Org Chem. 2021 Jan 5;2021(4):607–17. doi: 10.1002/ejoc.202001392 (PMC7898692; doi:10.1002/ejoc.202001392)
Supplement: Supplementary file 1 — Supplementary [file EJOC-2021-607-s001.pdf]

European Journal of Organic Chemistry

Supporting Information

Allosteric Guest Binding in Chiral Zirconium(IV) Double Decker Porphyrin Cages

Jeroen P. J. Bruekers, Matthijs A. Hellinghuizen, Nicolas Vanthuyne, Paul Tinnemans, Pieter J. Gilissen, Wybren Jan Buma, Jean-Valère Naubron,* Jeanne Crassous,* Johannes A. A. W. Elemans,* and Roeland J. M. Nolte*

Author Contributions

J.B. Data curation:Lead; Formal analysis:Lead; Investigation:Equal; Methodology:Equal; Writing – original draft:Equal; Writing – review & editing:Equal
M.H. Investigation:Supporting; Methodology:Supporting; Writing – review & editing:Supporting
N.V. Data curation:Equal; Formal analysis:Equal; Methodology:Equal; Writing – review & editing:Equal
P.T. Data curation:Supporting; Formal analysis:Supporting; Methodology:Supporting; Writing – review & editing:Supporting
P.G. Data curation:Equal; Formal analysis:Equal; Methodology:Equal; Writing – review & editing:Supporting
W.B. Data curation:Supporting; Formal analysis:Supporting; Methodology:Supporting; Validation:Supporting; Writing – review & editing:Supporting
J.-V.N. Data curation:Equal; Formal analysis:Equal; Methodology:Equal; Validation:Equal; Writing – review & editing:Equal
J.C. Data curation:Equal; Formal analysis:Equal; Methodology:Equal; Validation:Equal; Writing – review & editing:Equal
J.E. Conceptualization:Equal; Data curation:Equal; Formal analysis:Equal; Investigation:Equal; Methodology:Equal; Supervision:Equal; Validation:Equal; Visualization:Equal; Writing – review & editing:Equal
R.N. Conceptualization:Lead; Funding acquisition:Lead; Methodology:Lead; Supervision:Lead; Validation:Lead; Writing – original draft:Equal; Writing – review & editing:Lead

Contents

General methods 2

Syntheses 6

HPLC 9

Analytical chiral HPLC separation of **(±)-Zr(1)$_2$** ....

Preparative separation of **Zr(1)$_2$** ....

Chromatograms for **(*R*)-5**, **(*S*)-5**, **G3R**, and **G3S** ....

X-ray 14

Fluorescence 15

Oxygen quenching ....

Host guest binding 16

Host-guest binding of **(±)-Zr(1)$_2$** with **G1** as followed by NMR ....

Fluorescence titration data and fits ....

Fluorescence measured data ....

Fitted parameters for the fluorescence titration ....

Fitted data for the fluorescence titration ....

CD titration and fits for **(+)-Zr(1)$_2$** with **G1** ....

UV-Vis titration for **(±)-Zr(1)$_2$** with **G1** ....

Host-guest NMR titration data and fit for guests **G2**, **G3R** and **G3S** ....

NMR Spectral data 37

(*R*)-5 ....

(*S*)-5 ....

G3R ....

G3S ....

(±)-Zr(1)$_2$ ....

(±)-Zr(1)$_2$ with **G1** ....

(±)-Zr(1)$_2$ with **G1** (0, 1 and 2 equiv) at -28°C ....

Stacks of titration spectra 56

H$_2$1 with **G2** ....

(±)-Zr(1)$_2$ with **G2** ....

(+)-Zr(1)$_2$ with **G3R** ....

(+)-Zr(1)$_2$ with **G3S** ....

(–)-Zr(1)$_2$ with **G3R** ....
(–)-Zr(1)$_2$ with **G3S**..
Molecular Modelling 68
References 68

General methods

All solvents were dried and freshly distilled under an argon atmosphere using standard procedures. Reactions were followed using thin layer chromatography (TLC) on silica gel-coated plates (Merck 60-F254). Detection was performed with UV light at 254 nm. Column chromatography was performed manually using Acros silica gel, 0.035e0.070 mm, 60A or Silicagel for TLC 60 H, 0.005e0.040mm. NMR spectra were recorded at 298 K on a Bruker Avance III 500 spectrometer (500 MHz) equipped with a Prodigy BB cryoprobe, and on a Bruker Avance III 400 spectrometer (400 MHz) equipped with a BBFO probe. ^{1}H NMR chemical shifts (δ) are given in parts per million (ppm) and were referenced to tetramethylsilane (0.00 ppm) or to the residual solvent signal. Coupling constants are reported as *J* values in Hertz (Hz). Data for ^{1}H NMR spectra are reported as follows: chemical shift (multiplicity, coupling constant, integration). Multiplicities are abbreviated as s (singlet), d (doublet), t (triplet), m (multiplet), b (broad). Mass spectra were recorded on a Thermo Finnigan LCQ Advantage, Max mass spectrometer (MS) and on an JEOL AccuTOF CS JMS-T100CS mass spectrometer (HRMS) calibrated with CsI.

Reflections were measured on a Bruker D8 Quest diffractometer with sealed tube and Triumph monochromator (λ = 0.71073Å). Software package used for the intensity integration was Saint. Absorption correction was performed with SADABS. The structures were solved with direct methods using SHELXT. Least-squares refinement was performed with SHELXL-2018/3 against $|F_h^o|^2$ of all reflections. Non-hydrogen atoms were refined freely with anisotropic displacement parameters. Hydrogen atoms were placed on calculated positions or located in difference Fourier maps. All calculated hydrogen atoms were refined with a riding model. Diffracted intensity from disordered solvent was removed with SQUEEZE in PLATON.

The enantiomers of **Zr(1)$_2$** were separated by chiral HPLC using a Chiralpak IH (250 x 10 mm) column with ethanol/dichloromethane as the mobile phase, flow-rate = 5 mL/min, UV and circular dichroism detection at 254 nm.

UV-vis spectra were recorded on a JASCO V-630 UV-Vis spectrophotometer or on a JASCO J-815 CD spectrometer equipped with a JASCO Peltier cell holder PTC-423 to maintain the temperature at 25.0 ± 0.2°C. fluorescence spectra were recorded on a JASCO FP-8300ST spectrofluorometer or on a Spex Fluorolog 3 spectrometer. Electronic circular dichroism spectra were recorded on a JASCO J-815 CD spectrometer equipped with a JASCO Peltier cell holder PTC-423 to maintain temperature at 25.0 ± 0.2°C. The CD spectrometer was purged with nitrogen before recording each spectrum, which was baseline subtracted. The baseline was always measured for the same solvent and in the same cell as the samples. The spectra are presented without smoothing and further data processing. Infrared (IR) and vibrational circular dichroism (VCD) were recorded on a Jasco FSV-6000 spectrometer in CD_2Cl_2 in a 200 micron cell. The half-sum was used as the baseline. Melting points were recorded on a Jeneval THMS 600 hot-stage

polarization microscope and are uncorrected. Optical rotations were measured on an Anton Paar Polarimeter MCP100.

Cyclic voltammograms were measured in a 0.1 M solution of tetrabutyl ammonium hexafluorophosphate, with a platinum electrode, an amorphous carbon electrode and silver/silver choride 3M potassium chloride reference electrode.

Molecular models were compiled using the Spartan '14^{TM} chemistry software (equilibrium geometry, PM3, semi-empirical method, gas phase)

Fluorescence titrations were performed by preparing a 0.1 mM stock solution of **(±)-Zr(1)$_2$** and a 2 mM stock solution of **G1** in a degassed 1:1 v:v mixture of CH_2Cl_2 and MeCN. From these stock solutions the following solutions were prepared: three 3.0 µM solutions of **(±)-Zr(1)$_2$**, a mixture of 0.8 mM **G1** and **(±)-Zr(1)$_2$**, and a mixture of 0.08 mM **G1** and **(±)-Zr(1)$_2$**. The latter two mixtures were added in small quantities to one of the first three solutions under constant irradiation at 399 nm with a 5 nm excitation bandwidth, while obtaining the emission spectra between the additions to provide the data presented in Tables 3 – 5. This data at multiple wavelengths was fitted altogether using an online fitting tool: http://app.supramolecular.org/bindfit/ [s1,s2] to provide the binding parameters shown in Table S6 and the fits depicted in Tables S7 – S9. Circular dichroism titrations were performed in a similar way as the fluorescence titrations, but at a higher host concentration of 8 µM (Tables S10 – S12). A number was added to all data points to make them positive, because the fitting tool does not allow negative numbers. This number was subtracted for Figure S11. UV-Vis titrations were performed and analyzed analogous to the fluorescence titrations, and provided the spectra in Figure S13 and the binding parameters shown in Table S13. The concentration of **(±)-Zr(1)$_2$** in all solutions is 2.0 µM, solutions containing **G1** had concentrations of 0.08 mM, 0.8 mM and 8 mM. The measured and fitted data are depicted in Tables S14-16 and S17-19, respectively.

NMR titrations were performed by the addition of a solution of the appropriate guest with the host to a solution with the host (to account for dilution). Host concentrations varied from 0.20 to 1.0 mM. Chemical shifts obtained from the titrations for various host proton signals (Tables S20 – S32) were multiplied by the concentration of the host and fitted using the online program http://limhes.net/optim/ to yield the fits depicted in Tables S20 – S32. The titration spectra overlays are presented in Figures S34 – S45.

The emission life time was determined by a time correlated single photon counting experiment (TCSPC) irradiating a 0.65 [illegible]M solution of **(±)-Zr(1)$_2$**. Excitation is done by the output of a fully automatic tunable Ti:sapphire laser (Chameleon Ultra, Coherent), The final excitation wavelength (398 nm) is made with second harmonics generation (SHG APE). The repetition rate is decreased from the fundamental 80 MHz to a lower value (8 MHz) using a pulse picker (PulseSelect, APE). Fundamental light is guided via a delay line to a fast photodiode (PD) and use as the reference pulse. The excitation beam is directed to the cuvet, where the emission is collected using an uncoated, UV Fused Silica Aspheric Lens, f=50mm (Edmunds), at the magic angle (54.7) and focused on the entrance slit of the monochromator (Newport Cornerstone 260, f=250mm, grating 300ln/mm blaze 422 or grating 300ln/m blaze 750nm). A multichannel plate photomultiplier tube (MCP-PMT, R3809U-50, Hamamatsu) is used for wavelengths below 550nm, a second multichannel plate photomultiplier tube (MCP-PMT R3809U-51, Hamamatsu) is used above 550nm. The signal from the MCP's is amplified (RF Amplifier 8347A Hewlett Packard) to have a more stable output the CFD (constant fraction desciminator) in the TCSPC electronics. The time between reference signal and output the MCP is measured using a TCSPC board (SPC-130-EM, Becker & Hinkle). Delay

adjustments are done using an optical delay in the reference signal path and an electronic delay box (Ortec model 425). Although the excitation source produces sub-picosecond pulses, the electronics and the detector cause a broadening of the signal and are the limiting factor of the time resolution. The overall instrument response function (IRF) is around 20-25 ps (FWHM) measured from scattering from a ceramic plate at the excitation wavelength. The final histogram of the TCSPC is made using the SPCM program (Becker & Hinkle).

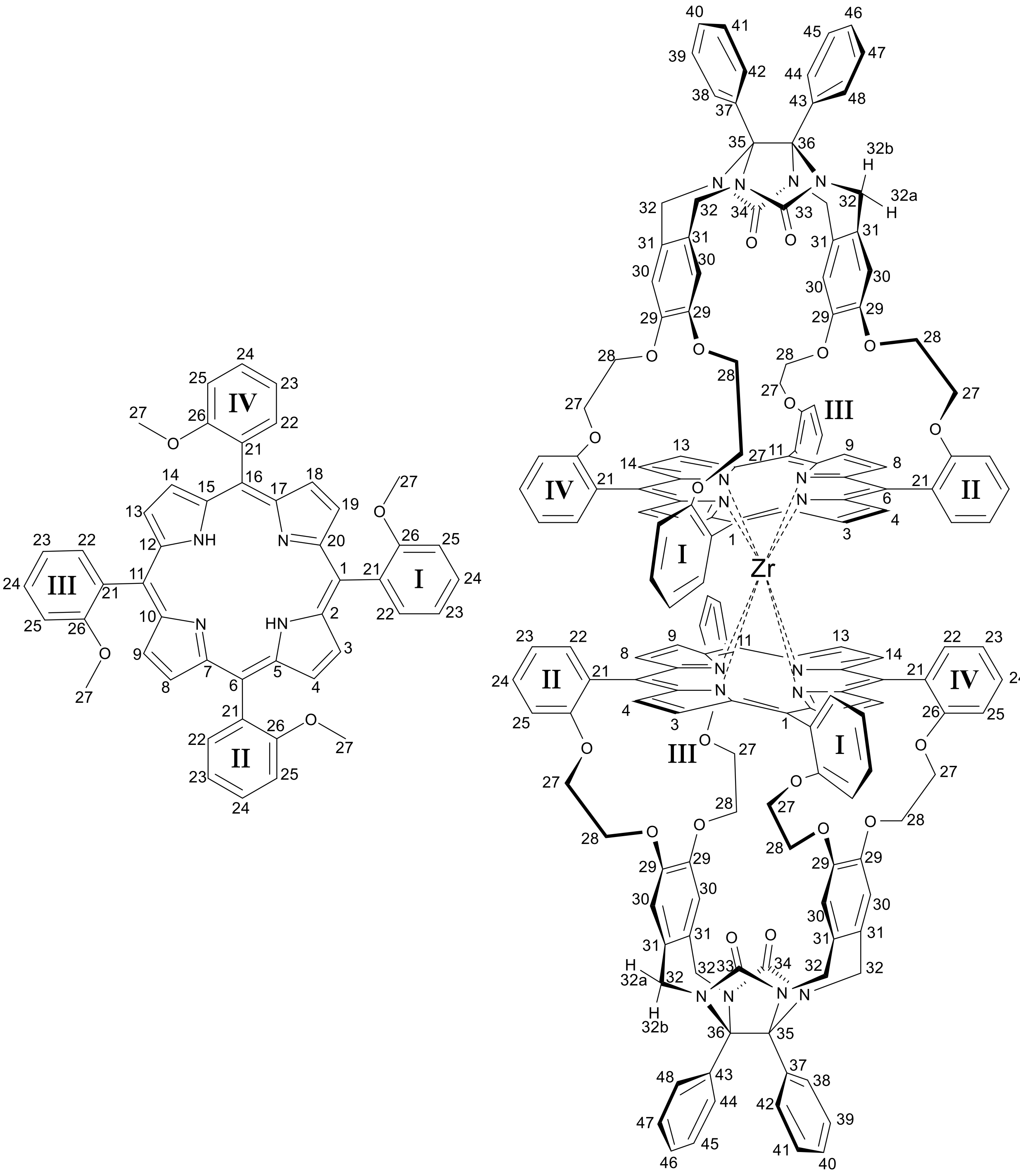

Figure S1. Numbering of the carbon atoms in **(±)-Zr(1)$_2$**; the signs I-IV indicate the four quadrants in which each of the porphyrin cages is divided for the assignment of the NMR signals.

Syntheses

Zr(Et$_2$N)$_4$ [s3]: $ZrCl_4$ (549 mg, 2.36 mmol) and Li(Et$_2$N) (760 mg, 9.61 mmol) were dissolved in argon-purged dry diethyl ether (9 mL) under an inert atmosphere. The mixture was stirred for 22 hours at 20 °C. The

suspension was filtered under an inert atmosphere and the filtrate was concentrated *in vacuo*, re-dissolved in *n*-pentane (10 mL), and filtered again. The *n*-pentane was removed *in vacuo* to yield **$Zr(Et_2N)_4$** as a yellow/orange liquid, which was used in the synthesis of **(±)-$Zr(1)_2$** without any further analysis or yield determination.

(±)-$Zr(1)_2$ The procedure as described by Kim et al. for $Zr(TPP)_2$ was followed[s3]**: $H_2$1**(450 mg, 0.334 mmol) (synthesized according to Gilissen et al[s4]) was dissolved in freshly distilled toluene (20 mL) and heated to reflux under an inert atmosphere. $Zr(Et_2N)_4$ (0.30 mL, 0.81 mmol) was added dropwise and the reaction was heated at reflux for 18 hours. The mixture was allowed to cool to room temperature, concentrated and purified by flash column chromatography (60H silicagel, eluent 5% MeCN in DCM). The product was precipitated from DCM and *n*-heptane and subsequently washed with *n*-pentane to yield **(±)-$Zr(1)_2$** (155 mg, 56 µmol, 33 %). The enantiomers were separated on a Chiralpak IH column (250 x 10 mm) with ethanol/DCM 40/60 v/v as the mobile phase, flow rate 5 mL/min, yielding both enantiomers with excellent purities. ee: >99.5% **(+)-$Zr(1)_2$**; >99.5% **(–)-$Zr(1)_2$**. Melting point > 300°C. HRMS (ESI-TOF) (*m/z*): [M + 2 Cs]$^+$ calcd. for $C_{168}H_{124}Cs_2N_{16}O_{20}Zr$, 3040.63337; found, 3040.63337.

Optical rotations could not be measured due to the intense brown/orange color of the products

^{1}H NMR (400 MHz, CD_2Cl_2) δ 9.73 (dd, *J* = 7.4, 1.9 Hz, 4H, **22(I, III)**), 9.55 (dd, *J* = 7.2, 2.1 Hz, 4H, **22(II, IV)**), 8.45 (d, *J* = 4.4 Hz, 4H, **8**,**18** or **9**,**19**), 8.17 (d, *J* = 4.4 Hz, 4H, **8**,**18** or **9**,**19**), 7.97 (d, *J* = 4.4 Hz, 4H, **3**, **13** or **4**, **14**), 7.83 (td, *J* = 7.9, 1.9 Hz, 4H, **24(I, III)**), 7.77 (td, *J* = 7.5, 1.2 Hz, 4H, **23(I, III)**), 7.73 – 7.62 (m, 12H, **23(II, IV), 24(II, IV)** and **3, 13** or **4, 14**), 7.13 – 7.07 (m, 4H, **25(I, III)**), 6.99 – 6.85 (m, 16H, **25(II, IV), 39-41**, **45-47**), 6.80 (d, *J* = 7.7 Hz, 4H, **38**,**42**,**44** or **48**), 6.77 – 6.71 (m, 4H, **38**,**42**,**44** or **48**), 6.01 (s, 4H, **30(II, IV)**), 5.87 (s, 4H, **30(I, III)**), 4.04 (d, *J* = 15.6 Hz, 4H, **32a(I, III)**), 3.99 (d, *J* = 15.9 Hz, 4H, **32a(II, IV)**), 3.56 (d, *J* = 15.7 Hz, 4H, **32b**), 3.56 (d, *J* = 15.4 Hz, 4H, **32b**), 3.39 – 3.30 (m, 4H, **27a(I, III)**), 3.18 – 3.10 (m, 4H, **27a(II, IV)**), 2.96 – 2.87 (m, 4H, **28a(I, III)**), 2.81 – 2.73 (m, 4H, **27b(I, III)**), 2.72 – 2.63 (m, 4H, **27b(II, IV)**), 2.47 – 2.37 (m, 8H, **28a(II, IV), 28b(I, III)**), 2.29 – 2.21 (m, 4H, **28b(II, IV)**).

^{13}C NMR (101 MHz, CD_2Cl_2) δ 158.41 (**26**), 158.22 (**26**), 157.20 (**33, 34**), 156.34 (**7, 17** or **10, 20**), 155.91 (**7, 17** or **10, 20**), 155.52 (**2, 12** or **5, 15**), 155.31 (**2, 12** or **5, 15**), 148.05 (**29(I, III)**), 146.80 (**29(II, IV)**), 138.13 (**22(I, III)**), 137.39 (**22(II, IV)**), 134.51, 132.80 (**21**), 132.75 (**21**), 131.84 (**31(I, III)**), 130.51 (**31(II, IV)**), 129.92 (**24(I, III)**), 129.77 (**24(II, IV)**), 129.39 (**8**,**18** or **9**,**19**), 129.10 (**8**,**18** or **9**,**19**), 128.92, 128.85, 128.81, 128.76 (**38**,**42**,**44** or **48**), 128.46 (**38**,**42**,**44** or **48**), 127.09 (**3**, **13** or **4**, **14**), 126.48 (**3**, **13** or **4**, **14**), 120.28 (**30(II, IV)**), 120.22 (**23**), 120.16 (**23**), 118.27, 117.75, 116.39 (**30(I, III)**), 113.66 (**25(I, III)**), 113.08 (**25(II, IV)**), 111.82 (**1, 11**), 111.50 (**6, 16**), 85.27 (**35, 36**), 68.24 (**28(II, IV)**), 68.03 (**27a(I, III)**), 67.10 (**28 (I, III)**), 66.88 (**27(II, IV)**), 44.61 (**32(II, IV)**), 44.32 (**32(I, III)**).

3,5-Bis(benzyloxy)benzoic acid (**4**)

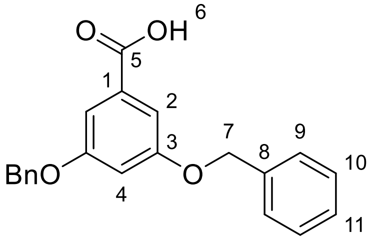

4

Benzyl bromide (5.3 mL, 45 mmol, 4.5 equiv) was added to a suspension of 3,5-dihydroxybenzoic acid (1.54 g, 10 mmol, 1.0 equiv) and potassium carbonate (6.2 g, 45 mmol, 4.5 equiv) in DMF (40 mL) and the mixture was stirred at 20 °C for 20 hours. The reaction mixture was diluted with EtOAc (200 mL) and successively washed with water (2 × 200 mL) and brine (2 × 200 mL). The organic layer was dried over Na_2SO_4 and the solvent was removed in vacuo. The residue was purified by silica gel column chromatography (60A silicagel, eluent EtOAc/*n*-heptane, 1:7, v/v) to afford benzyl 3,5-bis(benzyloxy)benzoate (4.2 g, quant.) as a white solid. Benzyl 3,5-

bis(benzyloxy)benzoate (4.2 g, 10 mmol, 1.0 equiv) was dissolved in THF/MeOH (50 mL, 1:1, v/v) and to this solution was added aqueous 2M NaOH (25 mL, 50 mmol, 5.0 equiv). The resulting turbid mixture was stirred at 20 °C for 70 hours, during which it slowly turned into a clear solution. After completion of the reaction (indicated by TLC, eluent: EtOAc/*n*-heptane, 1:3, v/v), most of the organic solvent was evaporated under reduced pressure. Then, aqueous 1M HCl was added until pH 1 was reached and the suspension was stirred vigorously. The resulting precipitate was filtered off, washed with water (2 × 50 mL), and dried under high vacuum to afford compound **4** (3.1 g, 94% over 2 steps) as a white solid.

^{1}H NMR (500 MHz, $CDCl_3$) δ 10.85 (bs, 1H, 6-O*H*), 7.47–7.31 (m, 10H, 9-C*H* + 10-C*H* + 11-C*H*), 7.35 (d, *J* = 2.2 Hz, 2H, 2-C*H*), 6.85 (t, *J* = 2.3 Hz, 1H, 4-C*H*), 5.09 (s, 4H, 7-CH_2). Note: the 6-O*H* signal displayed variable chemical shift and variable peak width. Spectral data were in agreement with literature values.[s5,s6]

(*R*)-1-Ethoxy-1-oxopropan-2-yl 3,5-bis(benzyloxy)benzoate ((*R*)-**5**) and **(*S*)-1-Ethoxy-1-oxopropan-2-yl 3,5-bis(benzyloxy)benzoate** ((*S*)-**5**)

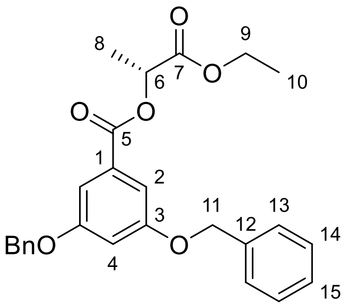

(*R*)-**5**

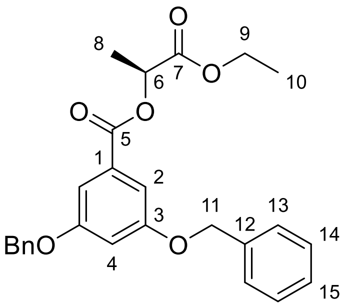

(*S*)-**5**

(*R*)-**5**: DIAD (1.05 mL, 5.4 mmol, 1.2 equiv) was added to a stirred solution of carboxylic acid **4**, (1.50 g, 4.5 mmol, 1.0 equiv), ethyl (*S*)-lactate (0.52 mL, 4.5 mmol, 1.0 equiv), and triphenylphosphine (1.42 g, 5.4 mmol, 1.2 equiv) in dry THF (20 mL) under an argon atmosphere. The resulting mixture was stirred at 20 °C for 3 hours. After completion (indicated by TLC, eluent: EtOAc), the mixture was evaporated to dryness and the residue was purified by silica gel column chromatography (60A silicagel, eluent: EtOAc/*n*-heptane, 1:7, v/v). The fractions containing the product were combined, evaporated, and purified by silica gel column chromatography (60A silicagel, eluent: DCM/*n*-heptane, 1:1, v/v) to afford ester (*R*)-**5** (1.92 g, 98%) as a pale yellow transparent oil.

$[\alpha]_D^{20}$ = −10.4° (c = 0.768 g/100 mL in EtOAc); ee >99%

^{1}H NMR (500 MHz, $CDCl_3$) δ 7.45–7.41 (m, 4H, 13-C*H*), 7.41–7.36 (m, 4H, 14-C*H*), 7.36–7.31 (m, 2H, 15-C*H*), 7.34 (d, *J* = 2.4 Hz, 2H, 2-C*H*), 6.82 (t, *J* = 2.4 Hz, 1H, 4-C*H*), 5.28 (q, *J* = 7.0 Hz, 1H, 6-C*H*), 5.07 (s, 4H, 11-CH_2), 4.23 (q, *J* = 7.1 Hz, 2H, 9-CH_2), 1.61 (d, *J* = 7.0 Hz, 3H, 8-CH_3), 1.28 (t, *J* = 7.1 Hz, 3H, 10-CH_3); ^{13}C NMR (126 MHz, $CDCl_3$) δ 170.84 (7-*C*), 165.78 (5-*C*), 159.94 (3-*C*), 136.58 (12-*C*), 131.54 (1-*C*), 128.78 (14-*C*), 128.29 (15-*C*), 127.76 (13-*C*), 108.87 (2-*C*), 107.59 (4-*C*), 70.49 (11-*C*), 69.52 (6-*C*), 61.56 (9-*C*), 17.21 (8-*C*), 14.28 (10-*C*); HRMS (ESI) calcd. for $[C_{26}H_{26}O_6 + Na]^+$ 457.16271, found 457.16323.

(*S*)-**5**: Following the procedure for (*R*)-**5**, the reaction of carboxylic acid **4** (1.50 g, 4.5 mmol, 1.0 equiv), ethyl (*R*)-lactate (0.52 mL, 4.5 mmol, 1.0 equiv), DIAD (1.05 mL, 5.4 mmol, 1.2 equiv), and triphenylphoshine (1.42 g, 5.4 mmol, 1.2 equiv) in dry THF (20 mL) afforded (*S*)-**5** (1.93 g, 99%) as a pale yellow transparent oil.

$[\alpha]_D^{20}$ +10.8° (c = 0.741 g/100 mL in EtOAc); ee >99%; ^{1}H NMR (500 MHz, $CDCl_3$) δ 7.45–7.41 (m, 4H, 13-C*H*), 7.41–7.36 (m, 4H, 14-C*H*), 7.36–7.31 (m, 2H, 15-C*H*), 7.34 (d, *J* = 2.4 Hz, 2H, 2-C*H*), 6.82 (t, *J* = 2.4 Hz, 1H, 4-C*H*), 5.28 (q, *J* = 7.0 Hz, 1H, 6-C*H*), 5.07 (s, 4H, 11-CH_2), 4.23 (q, *J* = 7.1 Hz, 2H, 9-CH_2), 1.61 (d, *J* = 7.0 Hz, 3H, 8-CH_3), 1.28 (t, *J* = 7.1 Hz, 3H, 10-CH_3); ^{13}C NMR (126 MHz, $CDCl_3$) δ 170.83 (7-*C*), 165.77 (5-*C*), 159.94 (3-*C*), 136.57 (12-*C*), 131.53 (1-*C*),

128.77 (14-*C*), 128.28 (15-*C*), 127.75 (13-*C*), 108.86 (2-*C*), 107.58 (4-*C*), 70.48 (11-*C*), 69.51 (6-*C*), 61.55 (9-*C*), 17.20 (8-*C*), 14.27 (10-*C*); HRMS (ESI) calcd. for $[C_{26}H_{26}O_6 + Na]^+$ 457.16271, found 457.16327

(*R*)-1-Ethoxy-1-oxopropan-2-yl 3,5-dihydroxybenzoate (G3R) and **(*S*)-1-Ethoxy-1-oxopropan-2-yl 3,5-dihydroxybenzoate (G3S)**

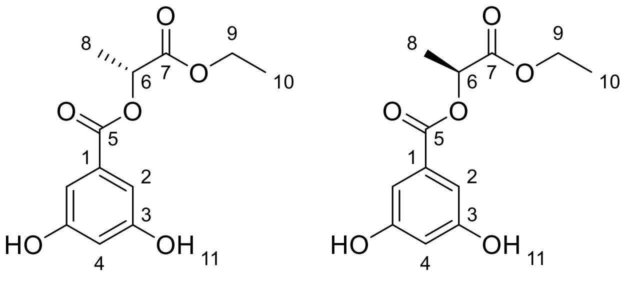

G3R **G3S**

G3R: Palladium on carbon (10 w%, 0.44 g, 0.41 mmol, 0.10 equiv) was added to a stirred solution of aryl benzyl ether (*R*)-**5** (1.8 g, 4.1 mmol, 1.0 equiv) in EtOAc (50 mL) under an argon atmosphere. The black suspension was purged with hydrogen (balloon) and the resulting mixture was stirred at 20 °C for 5.5 hours under a hydrogen atmosphere. Upon completion of the reaction (indicated by TLC, eluent: EtOAc/*n*-heptane, 1:1, v/v), the mixture was filtered through a plug of Celite and the filter cake was washed with EtOAc (2 × 50 mL). The combined filtrates were evaporated to dryness and dried under high vacuum to afford resorcylic ester **G3R** (1.1 g, quant.) as a pale brown sticky oil.

$[\alpha]_D^{20}$ −11.9° (c = 0.839 g/100 mL in EtOAc); ee 99%; ^{1}H NMR (500 MHz, $CDCl_3$) δ 7.05 (d, *J* = 2.3 Hz, 2H, 2-C*H*), 6.51 (t, *J* = 2.3 Hz, 1H, 4-C*H*), 6.33 (s, 2H, 11-O*H*), 5.26 (q, *J* = 7.0 Hz, 1H, 6-C*H*), 4.26 (q, *J* = 7.1 Hz, 2H, 9-CH_2), 1.61 (d, *J* = 7.0 Hz, 3H, 8-CH_3), 1.30 (t, *J* = 7.1 Hz, 3H, 10-CH_3). Note: the 11-O*H* signal displayed variable chemical shift and variable peak width; ^{13}C NMR (126 MHz, $CDCl_3$) δ 172.01 (7-*C*), 166.11 (5-*C*), 157.09 (3-*C*), 131.16 (1-*C*), 109.45 (2-*C*), 108.14 (4-*C*), 69.59 (6-*C*), 62.22 (9-*C*), 17.15 (8-*C*), 14.19 (10-*C*); HRMS (ESI) calcd. for $[C_{12}H_{14}O_6 + Na]^+$ 277.06881, found 277.06999.

G3S: Following the procedure for **G3R**, the reaction of (*S*)-**5** (1.8 g, 4.1 mmol, 1.0 equiv), palladium on carbon (10 w%, 0.44 g, 0.41 mmol, 0.10 equiv) and hydrogen (balloon) in EtOAc (50 mL) afforded **G3S** (1.1 g, quant.) as a pale brown sticky oil.

$[\alpha]_D^{20}$ +12.1° (c = 0.824 g/100 mL in EtOAc); ee >99%; ^{1}H NMR (500 MHz, $CDCl_3$) δ 7.06 (d, *J* = 2.3 Hz, 2H, 2-C*H*), 6.52 (t, *J* = 2.3 Hz, 1H, 4-C*H*), 6.09 (s, 2H, 11-O*H*), 5.26 (q, *J* = 7.0 Hz, 1H, 6-C*H*), 4.26 (q, *J* = 7.1 Hz, 2H, 9-CH_2), 1.62 (d, *J* = 7.0 Hz, 3H, 8-CH_3), 1.30 (t, *J* = 7.1 Hz, 3H, 10-CH_3). Note: the 11-O*H* signal displayed variable chemical shift and variable peak width; ^{13}C NMR (126 MHz, $CDCl_3$) δ 171.86 (7-*C*), 166.00 (5-*C*), 157.08 (3-*C*), 131.28 (1-*C*), 109.47 (2-*C*), 108.11 (4-*C*), 69.57 (6-*C*), 62.14 (9-*C*), 17.16 (8-*C*), 14.19 (10-*C*); HRMS (ESI) calcd. for $[C_{12}H_{14}O_6 + Na]^+$ 277.06881, found 277.06891.

HPLC

Analytical chiral HPLC separation of (±)-Zr(1)$_2$

• The sample was dissolved in dichloromethane, injected on the chiral column, and detected with an UV detector at 254 nm and a circular dichroism detector at 254 nm. The flow-rate was 1 mL/min.

Column	Mobile Phase	t1	k1	t2	k2	α	Rs
Chiralpak IH	Ethanol / dichloromethane (40/60)	4.45 (+)	0.51	7.82 (-)	1.65	3.25	4.82

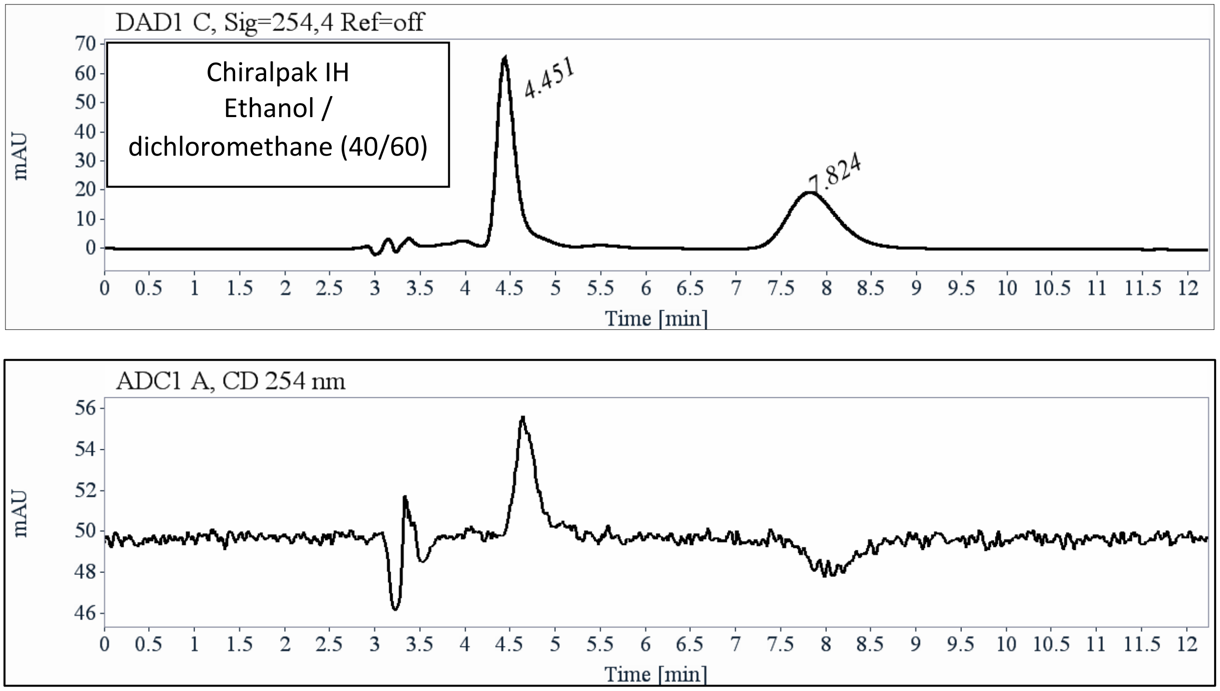

RT [min]	Area	Area%	Capacity Factor	Enantioselectivity	Resolution (USP)
4.45	773	54.12	0.51		
7.82	656	45.88	1.65	3.25	4.82
Sum	1429	100.00			

Figure S2. Chiral HPLC chromatograms (Chiralpak IH, ethanol/dichloromethane, 40:60, v/v,) **Zr(1)$_2$**

Preparative separation of **Zr(1)$_2$**

• Sample preparation: About 37 mg of compound **Zr(1)$_2$** was dissolved in 12 mL of dichloromethane.

• Chromatographic conditions: Chiralpak IH (250 x 10 mm), ethanol / dichloromethane (40/60, v/v) as mobile phase, flow-rate = 5 mL/min, UV detection at 254 nm.

• Injections (stacked): 40 times 300 µL, every 8 minutes.

• First fraction: 18 mg of the first eluted enantiomer with ee > 99.5 %

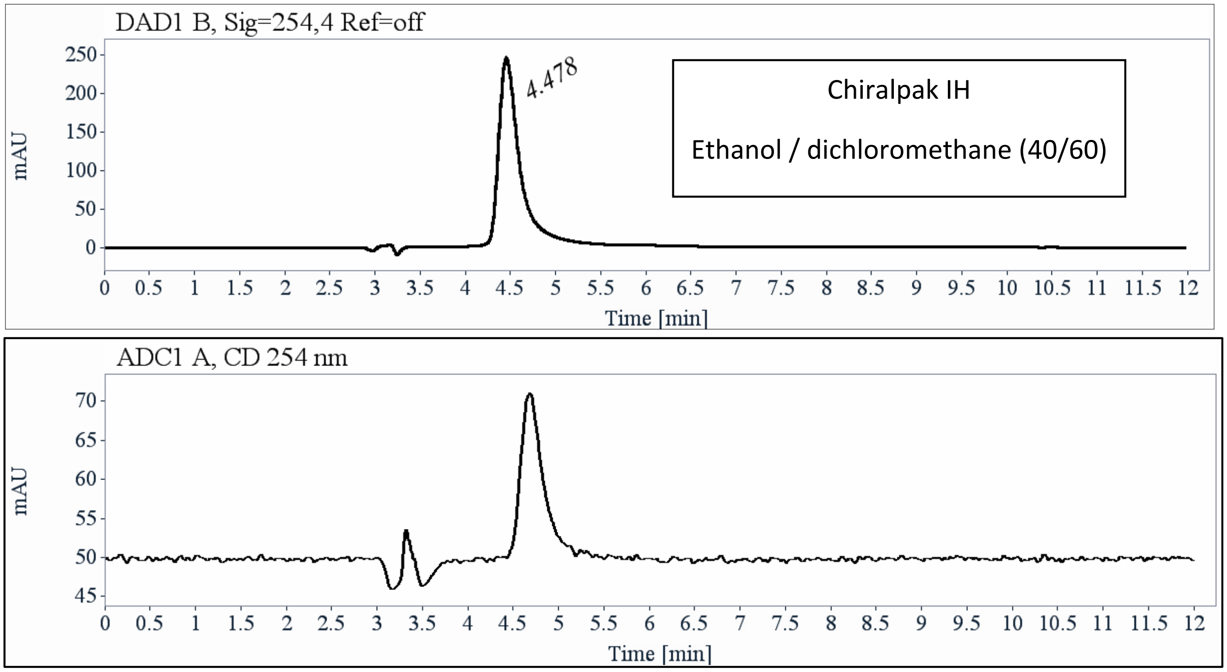

RT [min]	Area	Area%
4.48	3861	100.00
Sum	3861	100.00

Figure S3. Chiral HPLC chromatograms (Chiralpak IH, ethanol/dichloromethane, 40:60, v/v) **(+)-Zr(1)$_2$**

• Second fraction: 16 mg of the second eluted enantiomer with ee > 99.5 %

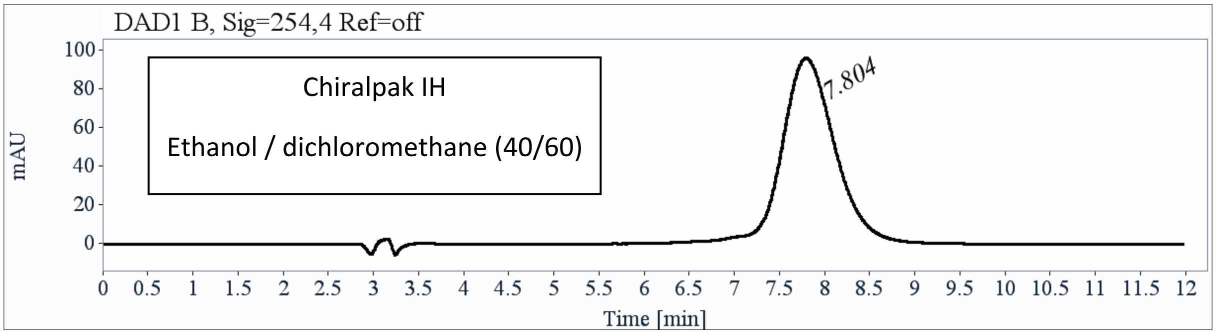

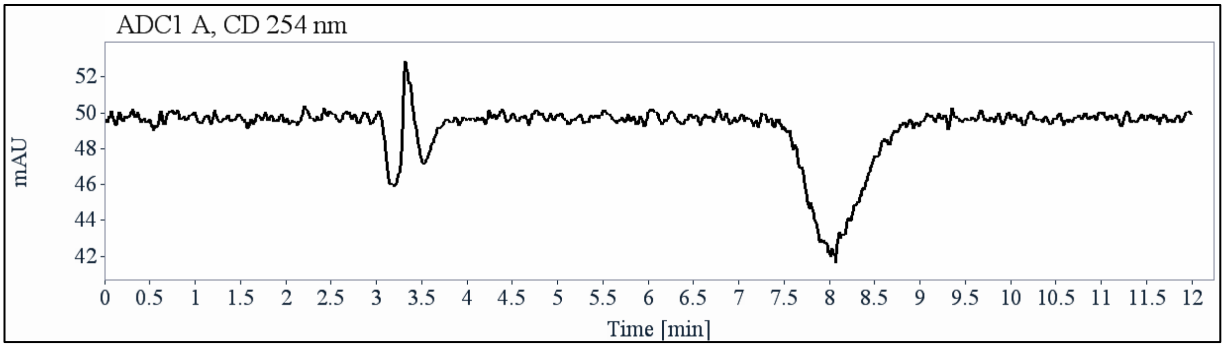

RT [min]	Area	Area%
7.80	3686	100.00
Sum	3686	100.00

Figure S4. Chiral HPLC chromatograms (Chiralpak IH, ethanol/dichloromethane, 40:60, v/v,) **(−)-Zr(1)$_2$**

Chromatograms for (*R*)-5, (*S*)-5, G3R, and G3S

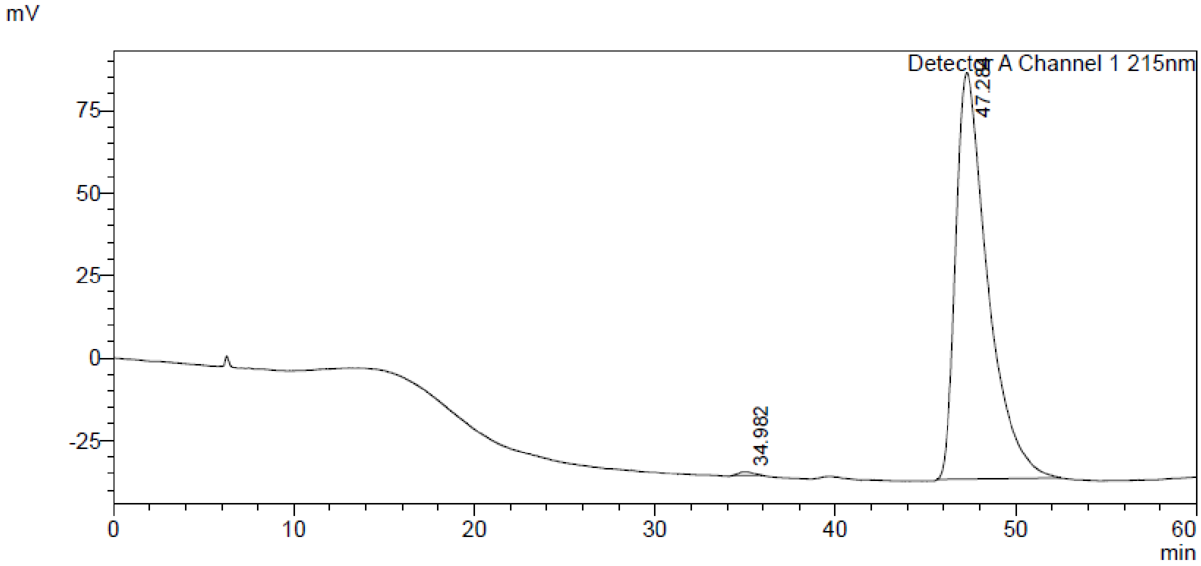

Peak#	Ret. Time	Area	Height	Area%	Height%
1	34.982	60848	1126	0.399	0.907
2	47.284	15172878	123024	99.601	99.093
Total		15233726	124150	100.000	100.000

Figure S5. Chiral HPLC chromatogram (Chiralpak AD-H, 2-propanol/*n*-heptane, 5:95, v/v, 308K) of (*R*)-1-ethoxy-1-oxopropan-2-yl 3,5-bis(benzyloxy)benzoate ((*R*)-**5**).

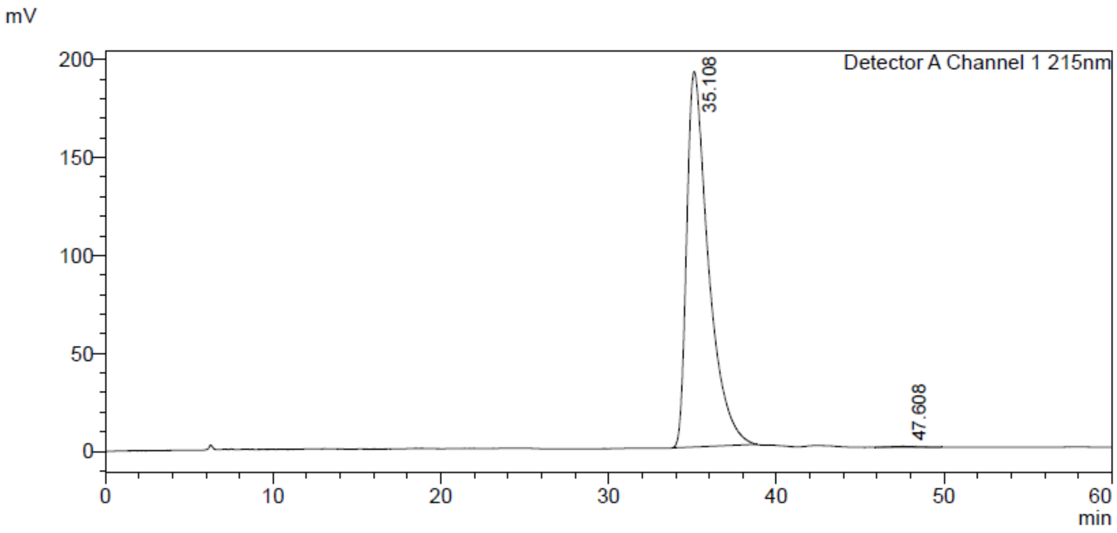

Peak#	Ret. Time	Area	Height	Area%	Height%
1	35.108	17285175	191588	99.685	99.763
2	47.608	54576	455	0.315	0.237
Total		17339751	192043	100.000	100.000

Figure S6. Chiral HPLC chromatogram (Chiralpak AD-H, 2-propanol/*n*-heptane, 5:95, v/v, 308K) of (*S*)-1-ethoxy-1-oxopropan-2-yl 3,5-bis(benzyloxy)benzoate ((*S*)-**5**).

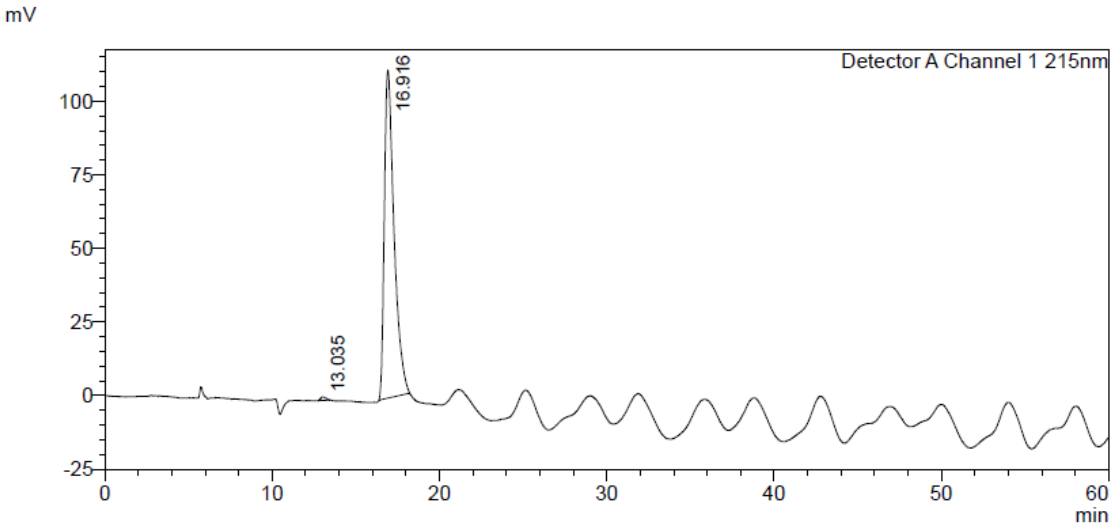

Peak#	Ret. Time	Area	Height	Area%	Height%
1	13.035	25884	1177	0.568	1.045
2	16.916	4530462	111413	99.432	98.955
Total		4556346	112589	100.000	100.000

Figure S7. Chiral HPLC chromatogram (Chiralpak AD-H, 2-propanol/*n*-heptane, 20:80, v/v, 308K) of (*R*)-1-ethoxy-1-oxopropan-2-yl 3,5-dihydroxybenzoate (**G3R**).

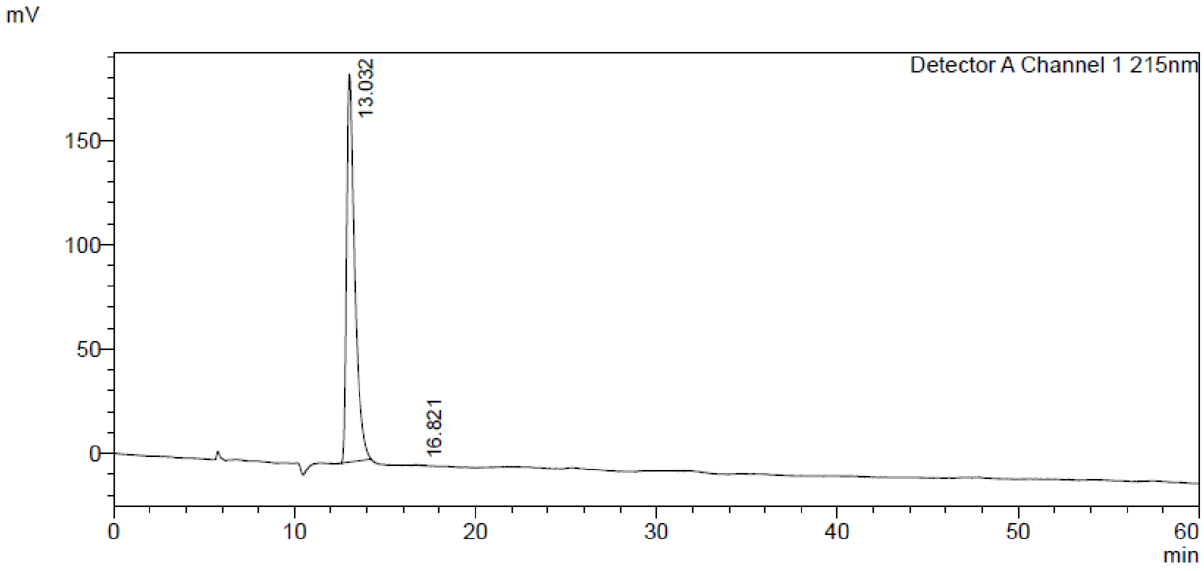

Peak#	Ret. Time	Area	Height	Area%	Height%
1	13.032	5768307	185169	99.900	99.852
2	16.821	5800	275	0.100	0.148
Total		5774107	185443	100.000	100.000

Figure S8. Chiral HPLC chromatogram (Chiralpak AD-H, 2-propanol/*n*-heptane, 20:80, v/v, 308K) of (*S*)-1-ethoxy-1-oxopropan-2-yl 3,5-dihydroxybenzoate (**G3S**).

X-ray

Data for the second eluted enantiomer ***M*-(–)-Zr(1)$_2$**

Table S1. Distances in the crystal structure; 4np = mean plane through the pyrrole nitrogens; 24mp = mean plane through the 24 atoms of the porphyrin; H-30 – H-30 is the distance between these protons on the same side of the same cavity; Zr3536p = plane through the zirconium center and carbons 35, 36. Cage 1 and 2 refer to either one of the **(–)-Zr(1)$_2$** cages in the unit cell.

	Structure element	Cage 1				Cage 2			
Distance	Zr – 24mp [Å]	1.672		1.657		1.679		1.651	
	Zr – 4np [Å]	1.292		1.286		1.275		1.272	
	24mp – 4np [Å]	0.380		0.371		0.404		0.379	
	H-30 – H-30 [Å]	6.703	6.721	6.265;	6.072	6.494	6.280	6.373	6.299
	carbonyls to 24mp [Å]	8.953	8.447	9.055	9.168	9.374	9.068	8.905	9.294
Angle	Zr3536p – Zr3536p [°]	46.84				58.40			
	Pyrrole 1 plane – 24mp[°]	20.04		17.33		11.94		14.69	
	Pyrrole 2 plane – 24mp [°]	15.64		17.42		26.21		19.55	
	Pyrrole 3 plane – 24mp [°]	16.68		14.80		11.17		16.08	
	Pyrrole 4 plane – 24mp [°]	18.66		20.22		25.37		19.96	

Table S2. Crystal data for **(–)-Zr(1)$_2$**

**Crystal structure and structure refinement**	
**General information**	
Identification code CCDC	2023416
Crystal colour	Red/blue
Crystal dimensions [mm] / shape	0.07 x 0.18 x 0.65/needle
Crystallization solvent	dichloromethane/heptane
Empirical formula	$C_{168}H_{124}N_{16}O_{20}Zr$ [+ solvent]
Formula weight [g/mol]	2778.04
**Crystal Data**	
Crystal system	triclinic
Space group	*P*1(No. 1)
Unit cell dimensions	
a, b, c [Å]	17.2672(8), 20.1516(11), 31.1628(16)
α, β, γ [°]	84.8154(18), 79.6418(17), 65.3056(16)
Volume [Å^3]	9689.9(9)
Z	2
Density (calculated) [g/cm^3]	0.952
Absorption coefficient (MoKα) [mm^{-1}]	0.112
F(000)	2888
**Data Collection**	
Temperature during experiment [K]	150
Wavelength [Å]	0.71073
θ Min-Max [°]	2.0, 28.4

Index range	-23≤ h ≤23 ; -26 ≤ k ≤26 ; -41 ≤ l ≤41
Tot., Uniq. Data, R(int)	600473, 95650, 0.112
Observed Data [I > 2.0 σ(I)]	57638
**Refinement**	
Nref, Npar	95650, 3691
R, wR2, S	0.0672, 0.1784, 1.03
Min. and Max. Resd. Dens. [e/ Å ^{3}]	-0.36, 0.70
**Bijvoet analysis**	
Number of Bijvoet Pairs	47317 (97%)
Flack x	0.050(18)
Parsons z	0.198(7)
P2(true)	1.000
P3(true)	1.000
P3(rac-twin)	0.000
G(su)	0.90(2)
Hooft y	0.049(12)

Fluorescence

Oxygen quenching

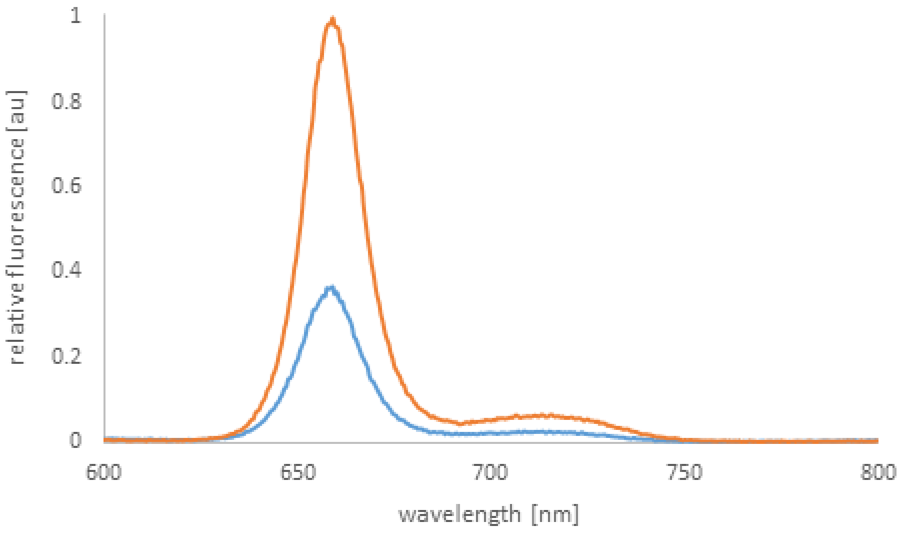

Figure S9. Oxygen quenching. fluorescence spectrum of **(±)-Zr(1)$_2$** in CH_2Cl_2: CH_3CN, 1:1 v/v 1.5 µM. Before (orange) and after (blue) 3 minutes of bubbling with oxygen.

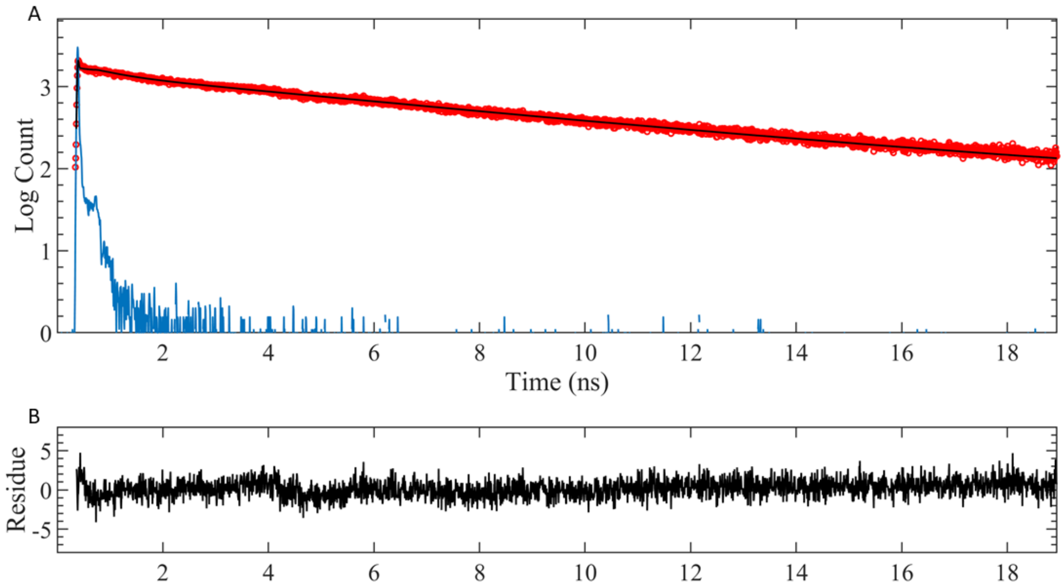

Figure S10. time correlated single photon counting experiment of a 0.65 μM **(±)-Zr(1)$_2$** solution in DCM:MeCN 1:1 v/v. (A) The deconvoluted data is displayed in black, the instrument response function (IRF) is displayed in blue, while the fit is displayed in red. (B) residual values of the fit.

Biexponential fitting of the TCSPC experiment was found to be the best descriptor of the emission decay process. The obtained lifetimes were τ = 0.60 ns and τ = 6.7 ns, which indicates that the observed emission is fluorescence. A Biexponential fluorescence decay has been reported for another distorted porphyrin (H_4TPP^{2+}) in literature.[s7]

Host guest binding

Host-guest binding of **(±)-Zr(1)$_2$** with **G1** as followed by NMR

(±)-Zr(1)$_2$ with **G1** (2 equiv). The guest signals were too broad to be analyzed due to fast exchange.

^{1}H NMR (400 MHz, CD_2Cl_2: CD_3CN, 1:1, v/v) δ 9.79 (dd, *J* = 7.2, 1.8 Hz, 4H, **22(I, III)**), 9.49 (dd, *J* = 7.5, 1.4 Hz, 4H, **22(II, IV)**), 8.44 (d, *J* = 4.6 Hz, 4H, **8**,**18** or **9**,**19**), 8.32 (d, *J* = 4.6 Hz, 4H, **3**, **13** or **4**, **14**), 8.26 (d, *J* = 4.6 Hz, 4H, **3**, **13** or **4**, **14**), 8.24 (d, *J* = 4.6 Hz, 4H, **8**,**18** or **9**,**19**), 7.89 – 7.79 (m, 8H, **23(I,III), 24(I,III)**), 7.78 – 7.69 (m, 4H, **24(II,IV)**), 7.69 – 7.60 (m, 4H, **23(II,IV)**), 7.04 (d, *J* = 7.9 Hz, 4H, **25(I,III)**), 6.98 (d, *J* = 8.1 Hz, 4H, **25(II,IV)**), 6.94 – 6.87 (m, 12H, **39**–**41, 45**–**47**), 6.85 – 6.79 (m, 8H, **38, 42, 44, 48**), 5.81 (s, 4H, **30(I,III)**), 5.60 (s, 4H, **30(II,IV)**), 3.98 (d, *J* = 15.7 Hz, 4H, **32a(I,III)**), 3.92 (d, *J* = 15.8 Hz, 4H, **32a(II,IV)**), 3.58 (d, *J* = 15.3 Hz, 4H, **32b(I,III)**), 3.54 (d, *J* = 15.7 Hz, 4H, **32b(II,IV)**), 3.46 (dd, *J* = 12.1, 4.6 Hz, 4H, **27a(I,III)**), 3.33 (d, *J* = 11.0 Hz, 4H, **27b(II,IV)**), 3.15 (t, *J* = 9.6 Hz, 4H, **27a(II,IV)**), 2.97 – 2.87 (m, 4H, **28a(I,III)**), 2.57 (dd, *J* = 12.2, 7.7 Hz, 4H, **27b(I,III)**), 2.49 – 2.33 (m, 8H, **28a(II,IV), 28b(I,III)**), 2.28 – 2.15 (m, 4H, **28b(II,IV)**).

^{13}C NMR (101 MHz, CD_2Cl_2: CD_3CN, 1:1, v/v) δ 157.83 (**33, 34**), 157.36 (**26**), 157.20 (**26**), 155.99, 155.55, 155.06, 146.91 (**29(I, III)**), 145.65 (**29(II, IV)**), 138.66 (**22(II, IV)**), 137.61 (**22(I, III)**), 133.74, 132.21 (**31(I, III)**), 131.11 (**8**,**18** or **9**,**19**), 130.73, 130.67, 130.66, 130.65, 130.07, 129.62 (**8**,**18** or **9**,**19**), 129.01, 128.65 (**3**, **13** or **4**, **14**), 128.47, 128.33,

128.03 (**3**, **13** or **4**, **14**), 120.41 (**23(II,IV)**), 120.32 (**30(II,IV)**), 120.13 (**23(I,III)**), 118.75, 118.42, 116.40 (**30(I,III)**), 113.11 (**25(II, IV)**), 112.89 **25(I, III)**, 85.44 (**35, 36**), 78.46, 78.33, 78.14, 77.81, 69.16 (**28(II, IV)**), 68.46 (**27(II, IV)**), 67.87 (**27(I, III)**), 66.86 (**28(I, III)**), 46.88, 43.94 (**32(I, III)**), 43.58 (**32(II, IV)**).

Fluorescence titration data and fits

Fluorescence measured data

Table S3a. Input data for fit of fluorescence titration measurement 1 of **(±)-Zr(1)$_2$** with **G1**

[H] [M]	[G] [M]	[G]/[H]	645.0 nm	646.0 nm	647.0 nm	648.0 nm	649.0 nm	650.0 nm	651.0 nm	652.0 nm	653.0 nm	654.0 nm
3.2435E-06	0	0	0.70284104	0.73421853	0.78243454	0.84036793	0.85193977	0.88910318	0.94481122	0.96624879	0.98583191	1
3.2435E-06	3.2806E-07	0.10114466	0.6130109	0.66768044	0.68029078	0.7160448	0.75535939	0.77546176	0.79786366	0.81818856	0.82686744	0.82486462
3.2435E-06	6.5351E-07	0.20148659	0.57243528	0.58897708	0.64861657	0.67131518	0.6802166	0.6940138	0.73102886	0.74245234	0.72991618	0.73206735
3.2435E-06	9.7639E-07	0.30103529	0.54728878	0.56575922	0.60707663	0.60596395	0.62168979	0.63845412	0.63986351	0.64861657	0.6619687	0.64490765
3.2435E-06	1.2967E-06	0.39980015	0.48572064	0.50864179	0.55092352	0.54128032	0.57013575	0.57473481	0.57206439	0.57911134	0.59439211	0.57903716
3.2435E-06	1.6146E-06	0.49779039	0.46465396	0.47236852	0.49150656	0.51301832	0.51746903	0.53230473	0.54573103	0.55722869	0.56056672	0.52970848
3.2435E-06	1.9299E-06	0.59501507	0.45449151	0.46049996	0.47963801	0.48253097	0.51079297	0.52622209	0.51249907	0.5172465	0.51057043	0.48720421
3.2435E-06	2.2428E-06	0.69148314	0.44811216	0.45894221	0.46524739	0.4906906	0.49209999	0.49907277	0.49454788	0.4948446	0.50307841	0.47644833
3.2435E-06	2.5533E-06	0.7872034	0.43898821	0.44937319	0.46287367	0.46873377	0.485869	0.47236852	0.49017135	0.47867369	0.48023144	0.47155256
3.2435E-06	2.8613E-06	0.88218451	0.4400267	0.46969809	0.46673095	0.48075069	0.49402863	0.49128403	0.49907277	0.48364365	0.48349529	0.47081077
3.2435E-06	3.167E-06	0.97643499	0.42941918	0.42059194	0.43824642	0.46294785	0.4607225	0.46213189	0.45412061	0.45182108	0.45345301	0.45011498
3.2435E-06	3.4704E-06	1.06996325	0.40812996	0.41977598	0.41599288	0.4238558	0.43550182	0.4414361	0.42771308	0.42800979	0.42333655	0.41940509
3.2435E-06	3.7714E-06	1.16277754	0.38149989	0.40264075	0.41154217	0.42474594	0.41510274	0.42504265	0.43913656	0.42615533	0.4083525	0.40397597
3.2435E-06	4.0702E-06	1.25488603	0.38216749	0.39188488	0.4004154	0.39789333	0.42437505	0.41725391	0.42125955	0.41436095	0.40004451	0.39173652
3.2435E-06	4.6609E-06	1.43701753	0.37163415	0.38313182	0.37719754	0.38654402	0.39166234	0.39589051	0.38194496	0.38313182	0.37467547	0.3566501
3.2435E-06	5.2428E-06	1.61642047	0.36555152	0.38780506	0.39707737	0.38572806	0.38506046	0.39581633	0.38825013	0.38676656	0.37200504	0.36035902
3.2435E-06	5.816E-06	1.79315571	0.34411394	0.34396558	0.36139752	0.36265856	0.35294118	0.36799941	0.37541725	0.35754024	0.34826793	0.33758623
3.2435E-06	6.3808E-06	1.9672823	0.31807729	0.34085009	0.34856465	0.34507826	0.35086418	0.34255619	0.33855055	0.32979749	0.33091017	0.31221719
3.2435E-06	6.9373E-06	2.1388576	0.30665381	0.29997775	0.32334397	0.32289889	0.32549514	0.31822565	0.31933833	0.31006602	0.31036273	0.29478525
3.2435E-06	7.4857E-06	2.30793725	0.32987167	0.33417402	0.32297307	0.34441065	0.33565759	0.33573177	0.34122098	0.31451673	0.30554113	0.30138714
3.2435E-06	8.0262E-06	2.47457532	0.30917588	0.32156368	0.31718715	0.33187449	0.32349232	0.31577776	0.3149618	0.31792894	0.29931014	0.29137304
3.2435E-06	1.0959E-05	3.37888655	0.27535049	0.27816928	0.28603219	0.28729323	0.29070544	0.28736741	0.28306505	0.28039463	0.2818782	0.27460871
3.2435E-06	1.3871E-05	4.27671526	0.25791855	0.26311105	0.26986129	0.27586974	0.26555893	0.2477561	0.2642979	0.25458052	0.25027817	0.23900304
3.2435E-06	1.6763E-05	5.16813091	0.23062087	0.23974483	0.25042653	0.25079742	0.25324531	0.24575328	0.23062087	0.22520585	0.2291373	0.2188265
3.2435E-06	2.2484E-05	6.93199592	0.22676359	0.22290631	0.22698613	0.22216453	0.22201617	0.21244715	0.22127439	0.20740301	0.20280395	0.20258141
3.2435E-06	2.8124E-05	8.67101776	0.20747719	0.21215043	0.22654106	0.21815889	0.21556264	0.20799644	0.21111194	0.20540019	0.20406498	0.19405089
3.2435E-06	3.917E-05	12.0766022	0.19204807	0.19701803	0.19486685	0.2033232	0.19323492	0.20451005	0.19902084	0.18900675	0.18047623	0.18166308
3.2435E-06	4.9913E-05	15.3888829	0.15725836	0.16207996	0.16534382	0.16578889	0.16148654	0.15206587	0.16304428	0.15659076	0.15354944	0.14828277
3.2435E-06	6.0366E-05	18.6116426	0.15651658	0.16052222	0.15666494	0.15614569	0.15577479	0.16378607	0.15518137	0.15139826	0.1547363	0.14360952
3.2435E-06	8.5303E-05	26.2999254	0.1261034	0.13240858	0.13174097	0.13493064	0.13188933	0.12588087	0.1323344	0.1212818	0.12276537	0.12054002
3.2435E-06	0.00010866	33.5016081	0.12283955	0.12654848	0.12321044	0.12788369	0.12602923	0.12313627	0.1195757	0.11616349	0.11720199	0.11690527
3.2435E-06	0.00013059	40.2614697	0.12209777	0.12306209	0.11742452	0.12157852	0.12009495	0.1174987	0.11126771	0.1206142	0.11920481	0.108894

Table S3b. Input data for fit of fluorescence titration measurement 1 of **(±)-Zr(1)$_2$** with **G1**

[H] [M]	[G] [M]	655.0 nm	656.0 nm	657.0 nm	658.0 nm	659.0 nm	660.0 nm	661.0 nm	662.0 nm	663.0 nm	664.0 nm	665.0 nm
3.2435E-06	0	0.99718122	0.98427416	0.97997181	0.94110229	0.91647504	0.85683555	0.82338105	0.75728804	0.69586826	0.64097619	0.59350197
3.2435E-06	3.2806E-07	0.81536978	0.80431719	0.80320451	0.7570655	0.72702322	0.68934055	0.6443884	0.59847192	0.53668126	0.51316668	0.46687931
3.2435E-06	6.5351E-07	0.71738002	0.71723166	0.69275276	0.67116683	0.63689637	0.59439211	0.56931978	0.52199392	0.45975818	0.43639196	0.39522291
3.2435E-06	9.7639E-07	0.64297901	0.61671983	0.60359024	0.56783621	0.55047845	0.51828499	0.47815444	0.45063423	0.40338254	0.37282101	0.34233365
3.2435E-06	1.2967E-06	0.56397893	0.5440991	0.52711223	0.50582301	0.48356947	0.46287367	0.41643795	0.39640976	0.34604258	0.31422001	0.29204065
3.2435E-06	1.6146E-06	0.52711223	0.514131	0.47882205	0.46124175	0.43372153	0.40575625	0.39314591	0.35635339	0.3177064	0.30146132	0.26733922
3.2435E-06	1.9299E-06	0.48742675	0.46895631	0.45946146	0.43045768	0.4179957	0.38164825	0.3673318	0.32289889	0.29337586	0.27134486	0.26185001
3.2435E-06	2.2428E-06	0.47800608	0.44840887	0.43461168	0.41636377	0.381129	0.37081819	0.33677027	0.31125287	0.27824345	0.26607818	0.24805282
3.2435E-06	2.5533E-06	0.46027743	0.43550182	0.41309992	0.39225577	0.37296936	0.35880128	0.33261627	0.30398338	0.2711965	0.26481715	0.23447815
3.2435E-06	2.8613E-06	0.47155256	0.43476003	0.42593279	0.40323418	0.37608486	0.35924635	0.33847637	0.31859654	0.29448854	0.26630072	0.24515985
3.2435E-06	3.167E-06	0.41562199	0.4148802	0.38409614	0.37081819	0.34544915	0.32987167	0.30754395	0.27994956	0.25710259	0.24931385	0.21763964
3.2435E-06	3.4704E-06	0.40100883	0.37868111	0.36970551	0.35301535	0.34203694	0.31251391	0.30316742	0.27460871	0.25101995	0.22898895	0.22253542
3.2435E-06	3.7714E-06	0.38454121	0.36488391	0.34997404	0.35138343	0.31637119	0.30613456	0.28135895	0.26733922	0.2408575	0.23403308	0.20495512
3.2435E-06	4.0702E-06	0.37957125	0.36540316	0.34960315	0.3342482	0.32178622	0.29612047	0.27357021	0.26066316	0.23625844	0.21556264	0.20280395
3.2435E-06	4.6609E-06	0.36384541	0.33788295	0.32237965	0.31726133	0.29137304	0.28098806	0.26095987	0.23173355	0.21830725	0.20495512	0.17995698
3.2435E-06	5.2428E-06	0.36540316	0.33966323	0.3332097	0.31629701	0.28788666	0.27045471	0.26437208	0.24738521	0.21637861	0.20910912	0.20287812
3.2435E-06	5.816E-06	0.32675618	0.30524442	0.29856836	0.28254581	0.26941622	0.25042653	0.23418144	0.21645279	0.19531192	0.1847044	0.16526964
3.2435E-06	6.3808E-06	0.30754395	0.30168385	0.27282843	0.2663749	0.25035235	0.23210444	0.2146725	0.21059269	0.18811661	0.17209406	0.16252504
3.2435E-06	6.9373E-06	0.29011201	0.27765003	0.26578147	0.25235517	0.23551665	0.21726875	0.2088124	0.19657295	0.17201988	0.16200579	0.15421705
3.2435E-06	7.4857E-06	0.29129887	0.28299088	0.27549885	0.25294859	0.24708849	0.22550256	0.21244715	0.19961427	0.19249314	0.16964617	0.16148654
3.2435E-06	8.0262E-06	0.2829167	0.28232327	0.2642979	0.24478896	0.23470069	0.22646688	0.20421334	0.19672131	0.17973444	0.17706402	0.15421705
3.2435E-06	1.0959E-05	0.25495141	0.2456791	0.24426971	0.22238706	0.20651287	0.19546028	0.17587716	0.17068467	0.15310437	0.14442549	0.14153253
3.2435E-06	1.3871E-05	0.22772791	0.23188191	0.21660114	0.20458423	0.20028188	0.18589125	0.17728655	0.16868185	0.15681329	0.13863957	0.13070247
3.2435E-06	1.6763E-05	0.22231288	0.20636451	0.19553446	0.18144055	0.17461613	0.16690157	0.15443958	0.15102737	0.1378236	0.12550998	0.12172687
3.2435E-06	2.2484E-05	0.19130628	0.18322083	0.17253913	0.16905274	0.15807433	0.15303019	0.14130999	0.13604332	0.12194941	0.11534753	0.11460574
3.2435E-06	2.8124E-05	0.18789407	0.17787998	0.1698687	0.16341518	0.1533269	0.13812032	0.14041985	0.13574661	0.12194941	0.12009495	0.11282546
3.2435E-06	3.917E-05	0.16883021	0.16578889	0.16200579	0.15592315	0.14138417	0.13493064	0.12729026	0.12766115	0.11430903	0.10622357	0.10244047
3.2435E-06	4.9913E-05	0.14309028	0.13493064	0.12684519	0.12936726	0.12039166	0.11312217	0.10652029	0.09687709	0.0961353	0.08382167	0.07736815
3.2435E-06	6.0366E-05	0.13596914	0.13463393	0.12365552	0.11334471	0.10867146	0.10763296	0.10147615	0.09390995	0.0854536	0.08678881	0.08827238
3.2435E-06	8.5303E-05	0.11319635	0.10985832	0.10444329	0.09984423	0.10325643	0.08612121	0.09450338	0.08575032	0.08078036	0.07677472	0.07276908
3.2435E-06	0.00010866	0.11801795	0.106817	0.10058601	0.09398413	0.09606112	0.08723388	0.08560196	0.07862918	0.08018693	0.07121133	0.07143387
3.2435E-06	0.00013059	0.1116386	0.09917662	0.09902826	0.09635784	0.09272309	0.0875306	0.08530524	0.08597285	0.07247237	0.06535124	0.06260663

Table S4a. Input data for fit of fluorescence titration measurement 2 of **(±)-Zr(1)$_2$** with **G1**

[H] [M]	[G] [M]	[G]/[H]	645.0 nm	646.0 nm	647.0 nm	648.0 nm	649.0 nm	650.0 nm	651.0 nm	652.0 nm	653.0 nm	654.0 nm
3.2435E-06	0	0	0.68932656	0.738723	0.79224905	0.84378971	0.87944727	0.9042249	0.93138501	0.97919314	0.98181385	0.96918679
3.2435E-06	3.2806E-07	0.10114466	0.56829733	0.60165184	0.63492694	0.65041296	0.67558767	0.68194091	0.70751271	0.6977446	0.72927255	0.71299238
3.2435E-06	6.5351E-07	0.20148659	0.55924396	0.5917249	0.60355781	0.66740788	0.65851334	0.67947903	0.70266836	0.70266836	0.71180114	0.71140407
3.2435E-06	9.7639E-07	0.30103529	0.44075604	0.44393266	0.48316391	0.49666455	0.50587675	0.5071474	0.51238882	0.53232211	0.5	0.50833863
3.2435E-06	1.2967E-06	0.39980015	0.41693139	0.43742058	0.45997459	0.47268107	0.49253494	0.48038437	0.48070203	0.4805432	0.46648666	0.48149619
3.2435E-06	1.6146E-06	0.49779039	0.41407243	0.4251906	0.44266201	0.45401842	0.46172173	0.47601652	0.46696315	0.47824015	0.46243647	0.45346252
3.2435E-06	1.9299E-06	0.59501507	0.41748729	0.41550191	0.44377382	0.44591804	0.45941868	0.44988882	0.46037166	0.4513183	0.44552097	0.43527637
3.2435E-06	2.2428E-06	0.69148314	0.39374206	0.39636277	0.4328939	0.43988247	0.43535578	0.43892948	0.44663278	0.44139136	0.43003494	0.41439009
3.2435E-06	2.5533E-06	0.7872034	0.37420584	0.39596569	0.40446315	0.40851334	0.42249047	0.41844028	0.41375476	0.40978399	0.41645489	0.4057338
3.2435E-06	2.8613E-06	0.88218451	0.37357052	0.40311309	0.41486658	0.42082274	0.44345616	0.44647395	0.42741423	0.43448221	0.41788437	0.41319886

3.2435E-06	3.167E-06	0.97643499	0.35054003	0.38190915	0.36316709	0.37944727	0.39040661	0.38738882	0.36880559	0.38786531	0.36809085	0.37055273
3.2435E-06	3.4704E-06	1.06996325	0.35459022	0.37539708	0.38325921	0.39263024	0.38603875	0.38357687	0.38365629	0.38381512	0.37515883	0.36078463
3.2435E-06	3.7714E-06	1.16277754	0.37492058	0.38905654	0.408831	0.40803685	0.39763342	0.39525095	0.39961881	0.39374206	0.38683291	0.37595299
3.2435E-06	4.0702E-06	1.25488603	0.37738247	0.39850699	0.40636912	0.41439009	0.41478717	0.41931385	0.4205845	0.40390724	0.40065121	0.38373571
3.2435E-06	4.6609E-06	1.43701753	0.3657878	0.36817027	0.37682656	0.3821474	0.38278272	0.37889136	0.37222046	0.36491423	0.35578145	0.35300191
3.2435E-06	5.2428E-06	1.61642047	0.33219504	0.34617217	0.34521919	0.36038755	0.34641042	0.35697268	0.35419314	0.3514136	0.33346569	0.31639136
3.2435E-06	5.816E-06	1.79315571	0.31226175	0.32449174	0.32655654	0.33457751	0.32512706	0.33886595	0.32758895	0.32647713	0.3263183	0.30400254
3.2435E-06	6.3808E-06	1.9672823	0.31162643	0.32385642	0.32075921	0.32465057	0.33060673	0.33521283	0.33179797	0.31551779	0.30813215	0.30336722
3.2435E-06	6.9373E-06	2.1388576	0.29367853	0.29788755	0.30281131	0.30614676	0.30614676	0.30598793	0.29375794	0.29606099	0.28923126	0.28256036
3.2435E-06	7.4857E-06	2.30793725	0.29383736	0.29074015	0.30138183	0.30638501	0.30471728	0.31162643	0.29955527	0.29264612	0.27731893	0.27565121
3.2435E-06	8.0262E-06	2.47457532	0.27112452	0.28136912	0.2874047	0.28359276	0.28549873	0.27882783	0.27684244	0.27843075	0.26564485	0.26215057
3.2435E-06	1.0959E-05	3.37888655	0.25595616	0.25825921	0.25214422	0.26270648	0.2556385	0.24841169	0.25698856	0.261277	0.24483799	0.23602287
3.2435E-06	1.3871E-05	4.27671526	0.24348793	0.24864994	0.25579733	0.24785578	0.25722681	0.24467916	0.23427573	0.23777001	0.23030496	0.21847205
3.2435E-06	1.6763E-05	5.16813091	0.23896125	0.24499682	0.24023189	0.24237611	0.23761118	0.23943774	0.24428208	0.21886912	0.21807497	0.21696315
3.2435E-06	2.2484E-05	6.93199592	0.21577192	0.22291931	0.22760483	0.22633418	0.21712198	0.21148348	0.20481258	0.21267471	0.19679161	0.19186785
3.2435E-06	2.8124E-05	8.67101776	0.20973634	0.21910737	0.20933926	0.21696315	0.2079892	0.21346887	0.19996823	0.20290661	0.19615629	0.18940597
3.2435E-06	3.917E-05	12.0766022	0.18829416	0.1931385	0.1952033	0.18607052	0.18321156	0.18980305	0.17249047	0.17042567	0.16677255	0.16113405
3.2435E-06	4.9913E-05	15.3888829	0.18638818	0.18948539	0.18781766	0.18146442	0.17741423	0.18710292	0.1741582	0.18400572	0.1634371	0.16677255
3.2435E-06	6.0366E-05	18.6116426	0.1808291	0.1741582	0.18503812	0.17963787	0.18591169	0.1751906	0.16724905	0.16502541	0.15978399	0.15430432
3.2435E-06	8.5303E-05	26.2999254	0.17757306	0.1839263	0.1797967	0.1772554	0.1828939	0.17789072	0.16176938	0.16304003	0.1511277	0.15684562
3.2435E-06	0.00010866	33.5016081	0.18011436	0.17908196	0.17812897	0.17598475	0.1695521	0.16756671	0.17090216	0.16296061	0.15795743	0.15176302
3.2435E-06	0.00013059	40.2614697	0.19290025	0.19250318	0.19139136	0.17844663	0.17971728	0.1803526	0.17995553	0.1690756	0.16709022	0.16041931

Table S4b. Input data for fit of fluorescence titration measurement 2 of **(±)-Zr(1)$_2$** with **G1**

[H] [M]	[H] [M]	[H] [M]	[H] [M]	[H] [M]	[H] [M]	[H] [M]	[H] [M]	[H] [M]	[H] [M]	[H] [M]	[H] [M]	[H] [M]
3.2435E-06	3.2435E-06	3.2435E-06	3.2435E-06	3.2435E-06	3.2435E-06	3.2435E-06	3.2435E-06	3.2435E-06	3.2435E-06	3.2435E-06	3.2435E-06	3.2435E-06
3.2435E-06	3.2435E-06	3.2435E-06	3.2435E-06	3.2435E-06	3.2435E-06	3.2435E-06	3.2435E-06	3.2435E-06	3.2435E-06	3.2435E-06	3.2435E-06	3.2435E-06
3.2435E-06	3.2435E-06	3.2435E-06	3.2435E-06	3.2435E-06	3.2435E-06	3.2435E-06	3.2435E-06	3.2435E-06	3.2435E-06	3.2435E-06	3.2435E-06	3.2435E-06
3.2435E-06	3.2435E-06	3.2435E-06	3.2435E-06	3.2435E-06	3.2435E-06	3.2435E-06	3.2435E-06	3.2435E-06	3.2435E-06	3.2435E-06	3.2435E-06	3.2435E-06
3.2435E-06	3.2435E-06	3.2435E-06	3.2435E-06	3.2435E-06	3.2435E-06	3.2435E-06	3.2435E-06	3.2435E-06	3.2435E-06	3.2435E-06	3.2435E-06	3.2435E-06
3.2435E-06	3.2435E-06	3.2435E-06	3.2435E-06	3.2435E-06	3.2435E-06	3.2435E-06	3.2435E-06	3.2435E-06	3.2435E-06	3.2435E-06	3.2435E-06	3.2435E-06
3.2435E-06	3.2435E-06	3.2435E-06	3.2435E-06	3.2435E-06	3.2435E-06	3.2435E-06	3.2435E-06	3.2435E-06	3.2435E-06	3.2435E-06	3.2435E-06	3.2435E-06
3.2435E-06	3.2435E-06	3.2435E-06	3.2435E-06	3.2435E-06	3.2435E-06	3.2435E-06	3.2435E-06	3.2435E-06	3.2435E-06	3.2435E-06	3.2435E-06	3.2435E-06
3.2435E-06	3.2435E-06	3.2435E-06	3.2435E-06	3.2435E-06	3.2435E-06	3.2435E-06	3.2435E-06	3.2435E-06	3.2435E-06	3.2435E-06	3.2435E-06	3.2435E-06
3.2435E-06	3.2435E-06	3.2435E-06	3.2435E-06	3.2435E-06	3.2435E-06	3.2435E-06	3.2435E-06	3.2435E-06	3.2435E-06	3.2435E-06	3.2435E-06	3.2435E-06
3.2435E-06	3.2435E-06	3.2435E-06	3.2435E-06	3.2435E-06	3.2435E-06	3.2435E-06	3.2435E-06	3.2435E-06	3.2435E-06	3.2435E-06	3.2435E-06	3.2435E-06
3.2435E-06	3.2435E-06	3.2435E-06	3.2435E-06	3.2435E-06	3.2435E-06	3.2435E-06	3.2435E-06	3.2435E-06	3.2435E-06	3.2435E-06	3.2435E-06	3.2435E-06
3.2435E-06	3.2435E-06	3.2435E-06	3.2435E-06	3.2435E-06	3.2435E-06	3.2435E-06	3.2435E-06	3.2435E-06	3.2435E-06	3.2435E-06	3.2435E-06	3.2435E-06
3.2435E-06	3.2435E-06	3.2435E-06	3.2435E-06	3.2435E-06	3.2435E-06	3.2435E-06	3.2435E-06	3.2435E-06	3.2435E-06	3.2435E-06	3.2435E-06	3.2435E-06
3.2435E-06	3.2435E-06	3.2435E-06	3.2435E-06	3.2435E-06	3.2435E-06	3.2435E-06	3.2435E-06	3.2435E-06	3.2435E-06	3.2435E-06	3.2435E-06	3.2435E-06
3.2435E-06	3.2435E-06	3.2435E-06	3.2435E-06	3.2435E-06	3.2435E-06	3.2435E-06	3.2435E-06	3.2435E-06	3.2435E-06	3.2435E-06	3.2435E-06	3.2435E-06
3.2435E-06	3.2435E-06	3.2435E-06	3.2435E-06	3.2435E-06	3.2435E-06	3.2435E-06	3.2435E-06	3.2435E-06	3.2435E-06	3.2435E-06	3.2435E-06	3.2435E-06
3.2435E-06	3.2435E-06	3.2435E-06	3.2435E-06	3.2435E-06	3.2435E-06	3.2435E-06	3.2435E-06	3.2435E-06	3.2435E-06	3.2435E-06	3.2435E-06	3.2435E-06
3.2435E-06	3.2435E-06	3.2435E-06	3.2435E-06	3.2435E-06	3.2435E-06	3.2435E-06	3.2435E-06	3.2435E-06	3.2435E-06	3.2435E-06	3.2435E-06	3.2435E-06
3.2435E-06	3.2435E-06	3.2435E-06	3.2435E-06	3.2435E-06	3.2435E-06	3.2435E-06	3.2435E-06	3.2435E-06	3.2435E-06	3.2435E-06	3.2435E-06	3.2435E-06
3.2435E-06	3.2435E-06	3.2435E-06	3.2435E-06	3.2435E-06	3.2435E-06	3.2435E-06	3.2435E-06	3.2435E-06	3.2435E-06	3.2435E-06	3.2435E-06	3.2435E-06
3.2435E-06	3.2435E-06	3.2435E-06	3.2435E-06	3.2435E-06	3.2435E-06	3.2435E-06	3.2435E-06	3.2435E-06	3.2435E-06	3.2435E-06	3.2435E-06	3.2435E-06
3.2435E-06	3.2435E-06	3.2435E-06	3.2435E-06	3.2435E-06	3.2435E-06	3.2435E-06	3.2435E-06	3.2435E-06	3.2435E-06	3.2435E-06	3.2435E-06	3.2435E-06
3.2435E-06	3.2435E-06	3.2435E-06	3.2435E-06	3.2435E-06	3.2435E-06	3.2435E-06	3.2435E-06	3.2435E-06	3.2435E-06	3.2435E-06	3.2435E-06	3.2435E-06
3.2435E-06	3.2435E-06	3.2435E-06	3.2435E-06	3.2435E-06	3.2435E-06	3.2435E-06	3.2435E-06	3.2435E-06	3.2435E-06	3.2435E-06	3.2435E-06	3.2435E-06
3.2435E-06	3.2435E-06	3.2435E-06	3.2435E-06	3.2435E-06	3.2435E-06	3.2435E-06	3.2435E-06	3.2435E-06	3.2435E-06	3.2435E-06	3.2435E-06	3.2435E-06
3.2435E-06	3.2435E-06	3.2435E-06	3.2435E-06	3.2435E-06	3.2435E-06	3.2435E-06	3.2435E-06	3.2435E-06	3.2435E-06	3.2435E-06	3.2435E-06	3.2435E-06
3.2435E-06	3.2435E-06	3.2435E-06	3.2435E-06	3.2435E-06	3.2435E-06	3.2435E-06	3.2435E-06	3.2435E-06	3.2435E-06	3.2435E-06	3.2435E-06	3.2435E-06
3.2435E-06	3.2435E-06	3.2435E-06	3.2435E-06	3.2435E-06	3.2435E-06	3.2435E-06	3.2435E-06	3.2435E-06	3.2435E-06	3.2435E-06	3.2435E-06	3.2435E-06
3.2435E-06	3.2435E-06	3.2435E-06	3.2435E-06	3.2435E-06	3.2435E-06	3.2435E-06	3.2435E-06	3.2435E-06	3.2435E-06	3.2435E-06	3.2435E-06	3.2435E-06
3.2435E-06	3.2435E-06	3.2435E-06	3.2435E-06	3.2435E-06	3.2435E-06	3.2435E-06	3.2435E-06	3.2435E-06	3.2435E-06	3.2435E-06	3.2435E-06	3.2435E-06
3.2435E-06	3.2435E-06	3.2435E-06	3.2435E-06	3.2435E-06	3.2435E-06	3.2435E-06	3.2435E-06	3.2435E-06	3.2435E-06	3.2435E-06	3.2435E-06	3.2435E-06

Table S5a. Input data for fit of fluorescence titration measurement 3 of **(±)-Zr(1)$_2$** with **G1**

[H] [M]	[H] [M]	[H] [M]	[H] [M]	[H] [M]	[H] [M]	[H] [M]	[H] [M]	[H] [M]	[H] [M]	[H] [M]	[H] [M]	[H] [M]
3.2435E-06	3.2435E-06	3.2435E-06	3.2435E-06	3.2435E-06	3.2435E-06	3.2435E-06	3.2435E-06	3.2435E-06	3.2435E-06	3.2435E-06	3.2435E-06	3.2435E-06
3.2435E-06	3.2435E-06	3.2435E-06	3.2435E-06	3.2435E-06	3.2435E-06	3.2435E-06	3.2435E-06	3.2435E-06	3.2435E-06	3.2435E-06	3.2435E-06	3.2435E-06
3.2435E-06	3.2435E-06	3.2435E-06	3.2435E-06	3.2435E-06	3.2435E-06	3.2435E-06	3.2435E-06	3.2435E-06	3.2435E-06	3.2435E-06	3.2435E-06	3.2435E-06
3.2435E-06	3.2435E-06	3.2435E-06	3.2435E-06	3.2435E-06	3.2435E-06	3.2435E-06	3.2435E-06	3.2435E-06	3.2435E-06	3.2435E-06	3.2435E-06	3.2435E-06
3.2435E-06	3.2435E-06	3.2435E-06	3.2435E-06	3.2435E-06	3.2435E-06	3.2435E-06	3.2435E-06	3.2435E-06	3.2435E-06	3.2435E-06	3.2435E-06	3.2435E-06
3.2435E-06	3.2435E-06	3.2435E-06	3.2435E-06	3.2435E-06	3.2435E-06	3.2435E-06	3.2435E-06	3.2435E-06	3.2435E-06	3.2435E-06	3.2435E-06	3.2435E-06
3.2435E-06	3.2435E-06	3.2435E-06	3.2435E-06	3.2435E-06	3.2435E-06	3.2435E-06	3.2435E-06	3.2435E-06	3.2435E-06	3.2435E-06	3.2435E-06	3.2435E-06
3.2435E-06	3.2435E-06	3.2435E-06	3.2435E-06	3.2435E-06	3.2435E-06	3.2435E-06	3.2435E-06	3.2435E-06	3.2435E-06	3.2435E-06	3.2435E-06	3.2435E-06
3.2435E-06	3.2435E-06	3.2435E-06	3.2435E-06	3.2435E-06	3.2435E-06	3.2435E-06	3.2435E-06	3.2435E-06	3.2435E-06	3.2435E-06	3.2435E-06	3.2435E-06
3.2435E-06	3.2435E-06	3.2435E-06	3.2435E-06	3.2435E-06	3.2435E-06	3.2435E-06	3.2435E-06	3.2435E-06	3.2435E-06	3.2435E-06	3.2435E-06	3.2435E-06
3.2435E-06	3.2435E-06	3.2435E-06	3.2435E-06	3.2435E-06	3.2435E-06	3.2435E-06	3.2435E-06	3.2435E-06	3.2435E-06	3.2435E-06	3.2435E-06	3.2435E-06
3.2435E-06	3.2435E-06	3.2435E-06	3.2435E-06	3.2435E-06	3.2435E-06	3.2435E-06	3.2435E-06	3.2435E-06	3.2435E-06	3.2435E-06	3.2435E-06	3.2435E-06
3.2435E-06	3.2435E-06	3.2435E-06	3.2435E-06	3.2435E-06	3.2435E-06	3.2435E-06	3.2435E-06	3.2435E-06	3.2435E-06	3.2435E-06	3.2435E-06	3.2435E-06
3.2435E-06	3.2435E-06	3.2435E-06	3.2435E-06	3.2435E-06	3.2435E-06	3.2435E-06	3.2435E-06	3.2435E-06	3.2435E-06	3.2435E-06	3.2435E-06	3.2435E-06
3.2435E-06	3.2435E-06	3.2435E-06	3.2435E-06	3.2435E-06	3.2435E-06	3.2435E-06	3.2435E-06	3.2435E-06	3.2435E-06	3.2435E-06	3.2435E-06	3.2435E-06
3.2435E-06	3.2435E-06	3.2435E-06	3.2435E-06	3.2435E-06	3.2435E-06	3.2435E-06	3.2435E-06	3.2435E-06	3.2435E-06	3.2435E-06	3.2435E-06	3.2435E-06
3.2435E-06	3.2435E-06	3.2435E-06	3.2435E-06	3.2435E-06	3.2435E-06	3.2435E-06	3.2435E-06	3.2435E-06	3.2435E-06	3.2435E-06	3.2435E-06	3.2435E-06
3.2435E-06	3.2435E-06	3.2435E-06	3.2435E-06	3.2435E-06	3.2435E-06	3.2435E-06	3.2435E-06	3.2435E-06	3.2435E-06	3.2435E-06	3.2435E-06	3.2435E-06
3.2435E-06	3.2435E-06	3.2435E-06	3.2435E-06	3.2435E-06	3.2435E-06	3.2435E-06	3.2435E-06	3.2435E-06	3.2435E-06	3.2435E-06	3.2435E-06	3.2435E-06
3.2435E-06	3.2435E-06	3.2435E-06	3.2435E-06	3.2435E-06	3.2435E-06	3.2435E-06	3.2435E-06	3.2435E-06	3.2435E-06	3.2435E-06	3.2435E-06	3.2435E-06
3.2435E-06	3.2435E-06	3.2435E-06	3.2435E-06	3.2435E-06	3.2435E-06	3.2435E-06	3.2435E-06	3.2435E-06	3.2435E-06	3.2435E-06	3.2435E-06	3.2435E-06
3.2435E-06	3.2435E-06	3.2435E-06	3.2435E-06	3.2435E-06	3.2435E-06	3.2435E-06	3.2435E-06	3.2435E-06	3.2435E-06	3.2435E-06	3.2435E-06	3.2435E-06
3.2435E-06	3.2435E-06	3.2435E-06	3.2435E-06	3.2435E-06	3.2435E-06	3.2435E-06	3.2435E-06	3.2435E-06	3.2435E-06	3.2435E-06	3.2435E-06	3.2435E-06
3.2435E-06	3.2435E-06	3.2435E-06	3.2435E-06	3.2435E-06	3.2435E-06	3.2435E-06	3.2435E-06	3.2435E-06	3.2435E-06	3.2435E-06	3.2435E-06	3.2435E-06
3.2435E-06	3.2435E-06	3.2435E-06	3.2435E-06	3.2435E-06	3.2435E-06	3.2435E-06	3.2435E-06	3.2435E-06	3.2435E-06	3.2435E-06	3.2435E-06	3.2435E-06
3.2435E-06	3.2435E-06	3.2435E-06	3.2435E-06	3.2435E-06	3.2435E-06	3.2435E-06	3.2435E-06	3.2435E-06	3.2435E-06	3.2435E-06	3.2435E-06	3.2435E-06
3.2435E-06	3.2435E-06	3.2435E-06	3.2435E-06	3.2435E-06	3.2435E-06	3.2435E-06	3.2435E-06	3.2435E-06	3.2435E-06	3.2435E-06	3.2435E-06	3.2435E-06
3.2435E-06	3.2435E-06	3.2435E-06	3.2435E-06	3.2435E-06	3.2435E-06	3.2435E-06	3.2435E-06	3.2435E-06	3.2435E-06	3.2435E-06	3.2435E-06	3.2435E-06
3.2435E-06	3.2435E-06	3.2435E-06	3.2435E-06	3.2435E-06	3.2435E-06	3.2435E-06	3.2435E-06	3.2435E-06	3.2435E-06	3.2435E-06	3.2435E-06	3.2435E-06
3.2435E-06	3.2435E-06	3.2435E-06	3.2435E-06	3.2435E-06	3.2435E-06	3.2435E-06	3.2435E-06	3.2435E-06	3.2435E-06	3.2435E-06	3.2435E-06	3.2435E-06
3.2435E-06	3.2435E-06	3.2435E-06	3.2435E-06	3.2435E-06	3.2435E-06	3.2435E-06	3.2435E-06	3.2435E-06	3.2435E-06	3.2435E-06	3.2435E-06	3.2435E-06
3.2435E-06	3.2435E-06	3.2435E-06	3.2435E-06	3.2435E-06	3.2435E-06	3.2435E-06	3.2435E-06	3.2435E-06	3.2435E-06	3.2435E-06	3.2435E-06	3.2435E-06

Table S5b. Input data for fit of fluorescence titration measurement 3 of **(±)-Zr(1)$_2$** with **G1**

[H] [M]	[H] [M]	[H] [M]	[H] [M]	[H] [M]	[H] [M]	[H] [M]	[H] [M]	[H] [M]	[H] [M]	[H] [M]	[H] [M]	[H] [M]
3.2435E-06	3.2435E-06	3.2435E-06	3.2435E-06	3.2435E-06	3.2435E-06	3.2435E-06	3.2435E-06	3.2435E-06	3.2435E-06	3.2435E-06	3.2435E-06	3.2435E-06
3.2435E-06	3.2435E-06	3.2435E-06	3.2435E-06	3.2435E-06	3.2435E-06	3.2435E-06	3.2435E-06	3.2435E-06	3.2435E-06	3.2435E-06	3.2435E-06	3.2435E-06
3.2435E-06	3.2435E-06	3.2435E-06	3.2435E-06	3.2435E-06	3.2435E-06	3.2435E-06	3.2435E-06	3.2435E-06	3.2435E-06	3.2435E-06	3.2435E-06	3.2435E-06
3.2435E-06	3.2435E-06	3.2435E-06	3.2435E-06	3.2435E-06	3.2435E-06	3.2435E-06	3.2435E-06	3.2435E-06	3.2435E-06	3.2435E-06	3.2435E-06	3.2435E-06
3.2435E-06	3.2435E-06	3.2435E-06	3.2435E-06	3.2435E-06	3.2435E-06	3.2435E-06	3.2435E-06	3.2435E-06	3.2435E-06	3.2435E-06	3.2435E-06	3.2435E-06
3.2435E-06	3.2435E-06	3.2435E-06	3.2435E-06	3.2435E-06	3.2435E-06	3.2435E-06	3.2435E-06	3.2435E-06	3.2435E-06	3.2435E-06	3.2435E-06	3.2435E-06
3.2435E-06	3.2435E-06	3.2435E-06	3.2435E-06	3.2435E-06	3.2435E-06	3.2435E-06	3.2435E-06	3.2435E-06	3.2435E-06	3.2435E-06	3.2435E-06	3.2435E-06
3.2435E-06	3.2435E-06	3.2435E-06	3.2435E-06	3.2435E-06	3.2435E-06	3.2435E-06	3.2435E-06	3.2435E-06	3.2435E-06	3.2435E-06	3.2435E-06	3.2435E-06
3.2435E-06	3.2435E-06	3.2435E-06	3.2435E-06	3.2435E-06	3.2435E-06	3.2435E-06	3.2435E-06	3.2435E-06	3.2435E-06	3.2435E-06	3.2435E-06	3.2435E-06
3.2435E-06	3.2435E-06	3.2435E-06	3.2435E-06	3.2435E-06	3.2435E-06	3.2435E-06	3.2435E-06	3.2435E-06	3.2435E-06	3.2435E-06	3.2435E-06	3.2435E-06
3.2435E-06	3.2435E-06	3.2435E-06	3.2435E-06	3.2435E-06	3.2435E-06	3.2435E-06	3.2435E-06	3.2435E-06	3.2435E-06	3.2435E-06	3.2435E-06	3.2435E-06
3.2435E-06	3.2435E-06	3.2435E-06	3.2435E-06	3.2435E-06	3.2435E-06	3.2435E-06	3.2435E-06	3.2435E-06	3.2435E-06	3.2435E-06	3.2435E-06	3.2435E-06
3.2435E-06	3.2435E-06	3.2435E-06	3.2435E-06	3.2435E-06	3.2435E-06	3.2435E-06	3.2435E-06	3.2435E-06	3.2435E-06	3.2435E-06	3.2435E-06	3.2435E-06
3.2435E-06	3.2435E-06	3.2435E-06	3.2435E-06	3.2435E-06	3.2435E-06	3.2435E-06	3.2435E-06	3.2435E-06	3.2435E-06	3.2435E-06	3.2435E-06	3.2435E-06
3.2435E-06	3.2435E-06	3.2435E-06	3.2435E-06	3.2435E-06	3.2435E-06	3.2435E-06	3.2435E-06	3.2435E-06	3.2435E-06	3.2435E-06	3.2435E-06	3.2435E-06
3.2435E-06	3.2435E-06	3.2435E-06	3.2435E-06	3.2435E-06	3.2435E-06	3.2435E-06	3.2435E-06	3.2435E-06	3.2435E-06	3.2435E-06	3.2435E-06	3.2435E-06
3.2435E-06	3.2435E-06	3.2435E-06	3.2435E-06	3.2435E-06	3.2435E-06	3.2435E-06	3.2435E-06	3.2435E-06	3.2435E-06	3.2435E-06	3.2435E-06	3.2435E-06
3.2435E-06	3.2435E-06	3.2435E-06	3.2435E-06	3.2435E-06	3.2435E-06	3.2435E-06	3.2435E-06	3.2435E-06	3.2435E-06	3.2435E-06	3.2435E-06	3.2435E-06
3.2435E-06	3.2435E-06	3.2435E-06	3.2435E-06	3.2435E-06	3.2435E-06	3.2435E-06	3.2435E-06	3.2435E-06	3.2435E-06	3.2435E-06	3.2435E-06	3.2435E-06
3.2435E-06	3.2435E-06	3.2435E-06	3.2435E-06	3.2435E-06	3.2435E-06	3.2435E-06	3.2435E-06	3.2435E-06	3.2435E-06	3.2435E-06	3.2435E-06	3.2435E-06
3.2435E-06	3.2435E-06	3.2435E-06	3.2435E-06	3.2435E-06	3.2435E-06	3.2435E-06	3.2435E-06	3.2435E-06	3.2435E-06	3.2435E-06	3.2435E-06	3.2435E-06
3.2435E-06	3.2435E-06	3.2435E-06	3.2435E-06	3.2435E-06	3.2435E-06	3.2435E-06	3.2435E-06	3.2435E-06	3.2435E-06	3.2435E-06	3.2435E-06	3.2435E-06
3.2435E-06	3.2435E-06	3.2435E-06	3.2435E-06	3.2435E-06	3.2435E-06	3.2435E-06	3.2435E-06	3.2435E-06	3.2435E-06	3.2435E-06	3.2435E-06	3.2435E-06
3.2435E-06	3.2435E-06	3.2435E-06	3.2435E-06	3.2435E-06	3.2435E-06	3.2435E-06	3.2435E-06	3.2435E-06	3.2435E-06	3.2435E-06	3.2435E-06	3.2435E-06
3.2435E-06	3.2435E-06	3.2435E-06	3.2435E-06	3.2435E-06	3.2435E-06	3.2435E-06	3.2435E-06	3.2435E-06	3.2435E-06	3.2435E-06	3.2435E-06	3.2435E-06
3.2435E-06	3.2435E-06	3.2435E-06	3.2435E-06	3.2435E-06	3.2435E-06	3.2435E-06	3.2435E-06	3.2435E-06	3.2435E-06	3.2435E-06	3.2435E-06	3.2435E-06
3.2435E-06	3.2435E-06	3.2435E-06	3.2435E-06	3.2435E-06	3.2435E-06	3.2435E-06	3.2435E-06	3.2435E-06	3.2435E-06	3.2435E-06	3.2435E-06	3.2435E-06
3.2435E-06	3.2435E-06	3.2435E-06	3.2435E-06	3.2435E-06	3.2435E-06	3.2435E-06	3.2435E-06	3.2435E-06	3.2435E-06	3.2435E-06	3.2435E-06	3.2435E-06
3.2435E-06	3.2435E-06	3.2435E-06	3.2435E-06	3.2435E-06	3.2435E-06	3.2435E-06	3.2435E-06	3.2435E-06	3.2435E-06	3.2435E-06	3.2435E-06	3.2435E-06
3.2435E-06	3.2435E-06	3.2435E-06	3.2435E-06	3.2435E-06	3.2435E-06	3.2435E-06	3.2435E-06	3.2435E-06	3.2435E-06	3.2435E-06	3.2435E-06	3.2435E-06
3.2435E-06	3.2435E-06	3.2435E-06	3.2435E-06	3.2435E-06	3.2435E-06	3.2435E-06	3.2435E-06	3.2435E-06	3.2435E-06	3.2435E-06	3.2435E-06	3.2435E-06
3.2435E-06	3.2435E-06	3.2435E-06	3.2435E-06	3.2435E-06	3.2435E-06	3.2435E-06	3.2435E-06	3.2435E-06	3.2435E-06	3.2435E-06	3.2435E-06	3.2435E-06

Fitted parameters for the fluorescence titration

Table S6. Fitted *K* values, error in the fit of the *K* values and the link to the fits

Measurement	K_{11} [M^{-1}]	K_{12} [M^{-1}]	K_{11} error [%]	K_{12} error [%]	Link to the fits
1	7475738.14	19726.1396	17.1102433	14.9704287	http://app.supramolecular.org/bindfit/view/12498751-c98d-4561-98a2-99c2d6a12a1d
2	10007265.3	19923.0891	28.1400686	44.2706587	http://app.supramolecular.org/bindfit/view/a49a2ddc-21d6-4c02-8e61-b6285cca3710
3	15755642	16352.4571	27.6604656	21.0595301	http://app.supramolecular.org/bindfit/view/ba6c9fca-76a0-4c57-9bb4-85437df6eff4

Fitted data for the fluorescence titration

Table S7a fitted data for the fluorescence titration measurement 1 of **(±)-Zr(1)$_2$** with **G1**

[H] [M]	[G] [M]	[G]/[H]	645.0 nm	646.0 nm	647.0 nm	648.0 nm	649.0 nm	650.0 nm	651.0 nm	652.0 nm	653.0 nm	654.0 nm
3.2435E-06	0	0	0.70284104	0.73421853	0.78243454	0.84036793	0.85193977	0.88910318	0.94481122	0.96624879	0.98583191	1
3.2435E-06	3.2806E-07	0.10114466	0.6660263	0.69497772	0.73877244	0.79071915	0.80149279	0.8344091	0.88369949	0.9018892	0.91860011	0.92970689
3.2435E-06	6.5351E-07	0.20148659	0.62987932	0.65644973	0.69590556	0.74197839	0.75196791	0.78071706	0.82371122	0.8387148	0.85260839	0.86071272
3.2435E-06	9.7639E-07	0.30103529	0.59451464	0.61875697	0.65397074	0.69430244	0.70352428	0.72820035	0.76504122	0.77693139	0.78807237	0.79324365
3.2435E-06	1.2967E-06	0.39980015	0.56009728	0.58207581	0.61316517	0.64791717	0.65639117	0.6771086	0.7079701	0.7168354	0.72530261	0.72762544
3.2435E-06	1.6146E-06	0.49779039	0.52686655	0.546662	0.57377475	0.60315011	0.61090105	0.62780373	0.65290472	0.65885661	0.66474934	0.66433036
3.2435E-06	1.9299E-06	0.59501507	0.49516764	0.51288443	0.53621183	0.5604736	0.56753337	0.58080772	0.6004318	0.60361478	0.60706182	0.6040394
3.2435E-06	2.2428E-06	0.69148314	0.46548338	0.4812591	0.50105322	0.52054831	0.52695878	0.5368506	0.55137191	0.55197668	0.55314805	0.54770494
3.2435E-06	2.5533E-06	0.7872034	0.43843751	0.45245256	0.46904393	0.48422752	0.49004343	0.49687527	0.5067848	0.50506167	0.50418062	0.49655681
3.2435E-06	2.8613E-06	0.88218451	0.41470767	0.42718892	0.44099332	0.45243809	0.45772819	0.46190581	0.46782139	0.46408535	0.46143285	0.45193047
3.2435E-06	3.167E-06	0.97643499	0.39479913	0.40600806	0.41750464	0.42587066	0.43071415	0.43270526	0.43533896	0.4299531	0.42585301	0.4148203
3.2435E-06	3.4704E-06	1.06996325	0.37877886	0.38898141	0.39865748	0.40461589	0.40909342	0.40937356	0.40944937	0.4027828	0.39756432	0.38535525
3.2435E-06	3.7714E-06	1.16277754	0.3662197	0.37565233	0.38394112	0.38808827	0.39227173	0.39126346	0.38942458	0.38180515	0.37576063	0.36268943
3.2435E-06	4.0702E-06	1.25488603	0.35642086	0.36527178	0.37251803	0.37532807	0.37927491	0.37731424	0.37407191	0.36576011	0.35912173	0.34543786
3.2435E-06	4.6609E-06	1.43701753	0.34241897	0.3504865	0.35634327	0.35743408	0.36102478	0.35783612	0.35281585	0.34364321	0.33628364	0.32187526
3.2435E-06	5.2428E-06	1.61642047	0.33281983	0.3404005	0.34540998	0.34552318	0.34885079	0.34495988	0.33895967	0.32933184	0.32161166	0.30686511
3.2435E-06	5.816E-06	1.79315571	0.32559338	0.33284445	0.33729344	0.33681841	0.33993402	0.33561705	0.32905473	0.3191834	0.31129007	0.29640545
3.2435E-06	6.3808E-06	1.9672823	0.31975098	0.32676198	0.33081324	0.32996841	0.33290267	0.32831471	0.32142365	0.31142649	0.30346359	0.28855074
3.2435E-06	6.9373E-06	2.1388576	0.3147851	0.32161098	0.32536383	0.32428041	0.32705338	0.32228767	0.31520722	0.30515393	0.29718229	0.28230518
3.2435E-06	7.4857E-06	2.30793725	0.31041564	0.31709232	0.32061132	0.31937278	0.32199867	0.31711463	0.30993269	0.29986683	0.29192399	0.27712155
3.2435E-06	8.0262E-06	2.47457532	0.30647704	0.31302931	0.31635863	0.31502059	0.31751012	0.31254742	0.30532193	0.29527181	0.28738178	0.27267845
3.2435E-06	1.0959E-05	3.37888655	0.28872648	0.29480954	0.29747588	0.29605576	0.29789614	0.2928334	0.28584705	0.27611381	0.26870558	0.2547378
3.2435E-06	1.3871E-05	4.27671526	0.27431611	0.28008874	0.2823646	0.28115996	0.28244676	0.27749872	0.27104186	0.26175527	0.25492597	0.2417778
3.2435E-06	1.6763E-05	5.16813091	0.26179452	0.26732496	0.26931928	0.26841215	0.26920755	0.26443577	0.25857007	0.24974563	0.24349277	0.23114375
3.2435E-06	2.2484E-05	6.93199592	0.24053743	0.24568872	0.24727224	0.24699839	0.24694747	0.24256396	0.23785423	0.22990044	0.22471151	0.21382026
3.2435E-06	2.8124E-05	8.67101776	0.22291571	0.22776994	0.22904905	0.22936917	0.22861017	0.22459646	0.22092702	0.2137413	0.20948027	0.19985191
3.2435E-06	3.917E-05	12.0766022	0.19514216	0.19954498	0.20037952	0.20170303	0.19982169	0.19643749	0.19448757	0.18855754	0.18580373	0.17821879
3.2435E-06	4.9913E-05	15.3888829	0.17417467	0.17824465	0.17876024	0.18087307	0.17814143	0.17525458	0.17464062	0.16967994	0.16808517	0.16206801
3.2435E-06	6.0366E-05	18.6116426	0.15777392	0.16158655	0.1618589	0.16460108	0.1612032	0.15871368	0.1591589	0.15496445	0.15428423	0.1495028
3.2435E-06	8.5303E-05	26.2999254	0.12906825	0.13243465	0.13229015	0.1361508	0.13158524	0.12980289	0.13212209	0.12928013	0.1302119	0.12760668
3.2435E-06	0.00010866	33.5016081	0.1105096	0.11358954	0.11317979	0.11777168	0.11245043	0.11113094	0.11467136	0.11270927	0.11468862	0.11349676
3.2435E-06	0.00013059	40.2614697	0.09753648	0.10041688	0.09982319	0.10492904	0.09907927	0.09808527	0.10248271	0.10113755	0.10385107	0.10364941

Table S7b fitted data for the fluorescence titration measurement 1 of **(±)-Zr(1)$_2$** with **G1**

[H] [M]	[G] [M]	655.0 nm	656.0 nm	657.0 nm	658.0 nm	659.0 nm	660.0 nm	661.0 nm	662.0 nm	663.0 nm	664.0 nm	665.0 nm
3.2435E-06	0	0.99718122	0.98427416	0.97997181	0.94110229	0.91647504	0.85683555	0.82338105	0.75728804	0.69586826	0.64097619	0.59350197
3.2435E-06	3.2806E-07	0.92613511	0.9128454	0.90725075	0.87100062	0.84690525	0.79210141	0.76023492	0.69963384	0.64243149	0.59223703	0.54826853

3.2435E-06	6.5351E-07	0.85640279	0.84273884	0.83587754	0.80219857	0.77862703	0.72856852	0.69826211	0.64305078	0.58998825	0.54440345	0.50387618
3.2435E-06	9.7639E-07	0.78821315	0.77418505	0.76608746	0.73492303	0.7118661	0.66644672	0.63766781	0.58772617	0.53871238	0.49763381	0.46047209
3.2435E-06	1.2967E-06	0.72189585	0.70751607	0.6982193	0.66950073	0.64694754	0.60603825	0.57874753	0.53392975	0.48885422	0.45215617	0.4182682
3.2435E-06	1.6146E-06	0.65792882	0.64321325	0.63276425	0.60640537	0.58434259	0.54778124	0.52192966	0.48205256	0.44077669	0.40830116	0.37757177
3.2435E-06	1.9299E-06	0.59700121	0.5819707	0.57043055	0.54631996	0.52473074	0.49230745	0.46783186	0.43265843	0.39500304	0.36654547	0.3388258
3.2435E-06	2.2428E-06	0.54007681	0.52475911	0.51220865	0.49019966	0.46906221	0.44050033	0.4173179	0.38653582	0.35226501	0.32755573	0.30264979
3.2435E-06	2.5533E-06	0.48840015	0.47283192	0.45937744	0.43927782	0.41856393	0.39350063	0.37150304	0.3447028	0.3135074	0.29219269	0.26984361
3.2435E-06	2.8613E-06	0.44332262	0.42755014	0.41332558	0.39489361	0.37456803	0.35254693	0.33159818	0.3082647	0.279756	0.26139089	0.24127571
3.2435E-06	3.167E-06	0.40585042	0.38992708	0.37508683	0.35804394	0.3380661	0.31856119	0.2985046	0.27804425	0.25177425	0.23584592	0.21759245
3.2435E-06	3.4704E-06	0.37611393	0.36009385	0.34479476	0.32885758	0.30918604	0.2916624	0.27233842	0.25414747	0.22966047	0.2156474	0.19887702
3.2435E-06	3.7714E-06	0.35325702	0.33718802	0.32156914	0.30648562	0.28708306	0.2710652	0.25233159	0.23587327	0.21276391	0.20020258	0.18457851
3.2435E-06	4.0702E-06	0.33587801	0.31979771	0.30396918	0.28953856	0.27037468	0.25548429	0.2372273	0.22207443	0.2000198	0.18854149	0.17379543
3.2435E-06	4.6609E-06	0.3121878	0.29615897	0.28013116	0.2666004	0.2478501	0.23445159	0.21691594	0.20351175	0.18291362	0.17285795	0.15932538
3.2435E-06	5.2428E-06	0.2971474	0.28122487	0.26516577	0.25221717	0.23382656	0.22132566	0.20432684	0.19199888	0.1723461	0.16313452	0.15039067
3.2435E-06	5.816E-06	0.28670702	0.2709167	0.25491145	0.2423756	0.22431173	0.21239458	0.19583099	0.18422314	0.16524297	0.15657042	0.14438857
3.2435E-06	6.3808E-06	0.27889803	0.26325196	0.24734567	0.23512523	0.2173656	0.20585453	0.18966508	0.17857492	0.16011055	0.15180474	0.14005454
3.2435E-06	6.9373E-06	0.27271287	0.25721617	0.24143367	0.22946823	0.21199589	0.20078282	0.18492731	0.174231	0.15618501	0.14814149	0.1367419
3.2435E-06	7.4857E-06	0.267598	0.25225209	0.23660725	0.2248567	0.20765791	0.19667292	0.18112277	0.17073958	0.15304723	0.14519872	0.13409585
3.2435E-06	8.0262E-06	0.26322837	0.24803262	0.23253299	0.22096918	0.20403224	0.19322772	0.17796133	0.16783578	0.15045152	0.14275247	0.13190839
3.2435E-06	1.0959E-05	0.2457231	0.23133352	0.21668025	0.20589474	0.19027669	0.18005745	0.16614694	0.15695917	0.14086656	0.13360199	0.12384552
3.2435E-06	1.3871E-05	0.23319688	0.21956141	0.20574306	0.19554064	0.18110018	0.17118041	0.15843002	0.14983153	0.13471288	0.12761688	0.11868273
3.2435E-06	1.6763E-05	0.22297114	0.21002991	0.19699483	0.18728001	0.17390457	0.16417636	0.15245723	0.14430353	0.13000201	0.12298052	0.1147372
3.2435E-06	2.2484E-05	0.20637732	0.19465994	0.18302174	0.17411253	0.16259369	0.15311082	0.1431694	0.1356926	0.12274436	0.11576566	0.10866759
3.2435E-06	2.8124E-05	0.1930335	0.18235504	0.17191098	0.16365769	0.15370406	0.14438162	0.1359284	0.12897066	0.11712602	0.11013775	0.10397422
3.2435E-06	3.917E-05	0.17240385	0.16338658	0.15485983	0.14762871	0.14016748	0.13105602	0.12496249	0.11878186	0.1086588	0.10161158	0.09690655
3.2435E-06	4.9913E-05	0.15701969	0.14926776	0.14220515	0.13574021	0.13017266	0.12120067	0.11689528	0.1112819	0.10245013	0.09533763	0.09172684
3.2435E-06	6.0366E-05	0.14505752	0.13829956	0.13238846	0.12652076	0.12243896	0.11356863	0.11066444	0.10548749	0.0976626	0.09049125	0.0877338
3.2435E-06	8.5303E-05	0.12422172	0.11920945	0.11532266	0.11049736	0.10902245	0.1003195	0.09987133	0.09544792	0.08938086	0.08209544	0.08082795
3.2435E-06	0.00010866	0.11079963	0.10691885	0.10434507	0.1001923	0.10040587	0.09180606	0.09294746	0.08900626	0.08407352	0.07670905	0.07640308
3.2435E-06	0.00013059	0.10143389	0.09834505	0.09669058	0.09300743	0.09440237	0.08587292	0.08812607	0.08452024	0.08037969	0.07295812	0.07332369

Table S8a fitted data for the fluorescence titration measurement 2 of **(±)-Zr(1)$_2$** with **G1**

[H] [M]	[G] [M]	[G]/[H]	645.0 nm	646.0 nm	647.0 nm	648.0 nm	649.0 nm	650.0 nm	651.0 nm	652.0 nm	653.0 nm	654.0 nm
3.2435E-06	0	0	0.68932656	0.738723	0.79224905	0.84378971	0.87944727	0.9042249	0.93138501	0.97919314	0.98181385	0.96918679
3.2435E-06	3.2806E-07	0.10114466	0.64950407	0.69465913	0.74290167	0.78931364	0.82093105	0.84291857	0.86644156	0.90847642	0.90964064	0.89751096
3.2435E-06	6.5351E-07	0.20148659	0.61031944	0.65130159	0.69434646	0.73571269	0.76335601	0.78259903	0.8025443	0.83890053	0.83863235	0.82699239
3.2435E-06	9.7639E-07	0.30103529	0.57187618	0.60876512	0.64671232	0.68312935	0.70687559	0.7234273	0.73986403	0.77065204	0.76897958	0.75782047
3.2435E-06	1.2967E-06	0.39980015	0.53432861	0.56722076	0.60019131	0.63177598	0.65171847	0.6656433	0.67865535	0.70400896	0.70096638	0.69027746
3.2435E-06	1.6146E-06	0.49779039	0.49791077	0.52692785	0.55507476	0.5819746	0.59823142	0.60961089	0.61930429	0.63939288	0.63502343	0.62479134
3.2435E-06	1.9299E-06	0.59501507	0.46298126	0.48828383	0.51180902	0.53421868	0.54694576	0.55588755	0.56240234	0.57744972	0.57181062	0.56201786
3.2435E-06	2.2428E-06	0.69148314	0.43008473	0.45189226	0.47107196	0.48925755	0.49866838	0.5053199	0.50884774	0.51916033	0.51233001	0.50295276
3.2435E-06	2.5533E-06	0.7872034	0.40000558	0.41862243	0.43383987	0.44817048	0.45456135	0.45912718	0.45993407	0.46593729	0.4580247	0.44903004
3.2435E-06	2.8613E-06	0.88218451	0.37372075	0.38955698	0.40132857	0.41230149	0.41607181	0.41882773	0.4172722	0.41953956	0.41069161	0.4020353
3.2435E-06	3.167E-06	0.97643499	0.3520943	0.36565337	0.37461328	0.3828391	0.38447938	0.38576405	0.38228639	0.38152224	0.37191939	0.36354715
3.2435E-06	3.4704E-06	1.06996325	0.33536675	0.34717807	0.35399304	0.3601138	0.36013968	0.36030915	0.35537223	0.352317	0.34214892	0.33400361
3.2435E-06	3.7714E-06	1.16277754	0.3229545	0.3334842	0.33874112	0.34332206	0.34218729	0.34155483	0.33556599	0.3308711	0.32030461	0.3123358
3.2435E-06	4.0702E-06	1.25488603	0.3138324	0.32343559	0.32758128	0.33105297	0.32910279	0.32790681	0.32117604	0.31533704	0.30449892	0.29666802
3.2435E-06	4.6609E-06	1.43701753	0.30178063	0.31019799	0.3129596	0.31502141	0.31208732	0.31021095	0.30257734	0.29537811	0.28423372	0.27660534
3.2435E-06	5.2428E-06	1.61642047	0.29421491	0.30192795	0.30390884	0.30514393	0.30169014	0.29945393	0.29133473	0.28344034	0.27215887	0.26467896
3.2435E-06	5.816E-06	1.79315571	0.28888389	0.29613059	0.29762721	0.29832335	0.29457651	0.29213699	0.28373626	0.27547059	0.26413374	0.25677438
3.2435E-06	6.3808E-06	1.9672823	0.28478723	0.29169749	0.29287015	0.29318402	0.28926552	0.28670653	0.27813381	0.26966959	0.25832035	0.25106528
3.2435E-06	6.9373E-06	2.1388576	0.28144061	0.28809208	0.28903536	0.28906031	0.28504077	0.28241111	0.27373038	0.26516749	0.25383019	0.24666886
3.2435E-06	7.4857E-06	2.30793725	0.27858689	0.28502955	0.28580333	0.28559917	0.28152246	0.27885245	0.27010363	0.26150356	0.25019279	0.24311769
3.2435E-06	8.0262E-06	2.47457532	0.27607827	0.28234627	0.28299058	0.28259796	0.27849272	0.27580214	0.26701147	0.25841391	0.24713869	0.24014413
3.2435E-06	1.0959E-05	3.37888655	0.26531937	0.27091976	0.27118797	0.27010565	0.26607705	0.263435	0.25462978	0.24636557	0.23535493	0.22874913
3.2435E-06	1.3871E-05	4.27671526	0.25698888	0.26213732	0.26225717	0.26073537	0.25692484	0.25442938	0.24574443	0.23799554	0.22727918	0.22100895
3.2435E-06	1.6763E-05	5.16813091	0.2499047	0.25469512	0.25474661	0.25288935	0.24932848	0.2470018	0.23847234	0.23126576	0.22083583	0.21486496
3.2435E-06	2.2484E-05	6.93199592	0.23805965	0.24228245	0.24228783	0.2399148	0.23684716	0.2348547	0.22664804	0.22047102	0.21056271	0.20510887
3.2435E-06	2.8124E-05	8.67101776	0.22834146	0.23211543	0.23212006	0.22934841	0.22672664	0.2250367	0.217129	0.21186345	0.2024063	0.19738562
3.2435E-06	3.917E-05	12.0766022	0.21313165	0.2162197	0.21625948	0.21288801	0.21100442	0.20981567	0.20240936	0.19863574	0.18990745	0.18557351
3.2435E-06	4.9913E-05	15.3888829	0.20170628	0.20428692	0.20437037	0.20055976	0.19924986	0.19845077	0.19143697	0.18881516	0.18064517	0.17683127
3.2435E-06	6.0366E-05	18.6116426	0.19279446	0.19498227	0.19510627	0.19095741	0.1901022	0.189612	0.18291035	0.18119862	0.17346818	0.17006148
3.2435E-06	8.5303E-05	26.2999254	0.17723884	0.17874516	0.17894914	0.17421597	0.17416465	0.17422063	0.1680723	0.16796572	0.16100825	0.15831456
3.2435E-06	0.00010866	33.5016081	0.16720641	0.16827522	0.1685352	0.16342812	0.16390017	0.16431178	0.15852438	0.15946098	0.15300481	0.15077202
3.2435E-06	0.00013059	40.2614697	0.16020359	0.1609677	0.1612683	0.15590122	0.15674029	0.15740131	0.15186724	0.15353474	0.14742944	0.14551875

Table S8b fitted data for the fluorescence titration measurement 2 of **(±)-Zr(1)$_2$** with **G1**

[H] [M]	[G] [M]	655.0 nm	656.0 nm	657.0 nm	658.0 nm	659.0 nm	660.0 nm	661.0 nm	662.0 nm	663.0 nm	664.0 nm	665.0 nm
3.2435E-06	0	1	0.97363405	0.97998729	0.95155654	0.90803685	0.86459657	0.80908513	0.76087992	0.69139136	0.63826239	0.59648983
3.2435E-06	3.2806E-07	0.92345038	0.89803221	0.90209955	0.87473851	0.83466469	0.79425492	0.74299642	0.6985562	0.63479835	0.58598135	0.54726693
3.2435E-06	6.5351E-07	0.84813839	0.82365373	0.82547302	0.79916558	0.76248183	0.7250537	0.67797976	0.63724364	0.57912403	0.53454915	0.49884358
3.2435E-06	9.7639E-07	0.77426686	0.75069929	0.75031472	0.72504235	0.69168368	0.65718035	0.61421143	0.57710856	0.52451961	0.4841055	0.45135146
3.2435E-06	1.2967E-06	0.70213806	0.67946793	0.67693317	0.65267367	0.62256145	0.59091419	0.55195414	0.5183988	0.47121036	0.43485858	0.40498677
3.2435E-06	1.6146E-06	0.63221031	0.61041309	0.60579612	0.58252177	0.55555667	0.52667869	0.49160617	0.4614901	0.41953787	0.38712405	0.36004696
3.2435E-06	1.9299E-06	0.56518582	0.54422943	0.53762023	0.51529482	0.49134579	0.46512264	0.43377773	0.40695812	0.37002541	0.34138546	0.31698774
3.2435E-06	2.2428E-06	0.50213087	0.48197184	0.4734937	0.45206813	0.43095592	0.40723121	0.37939534	0.35567693	0.32346756	0.29837707	0.27650104
3.2435E-06	2.5533E-06	0.44458101	0.4251594	0.4149836	0.39439016	0.3758662	0.35442304	0.32979325	0.30890524	0.28100849	0.25915619	0.23958326
3.2435E-06	2.8613E-06	0.39444823	0.37568355	0.36404111	0.34418888	0.32791821	0.30846445	0.28663265	0.26821019	0.24407276	0.22503924	0.2074749
3.2435E-06	3.167E-06	0.35342274	0.33521628	0.32239094	0.30316842	0.28873975	0.27091666	0.25138178	0.23497689	0.21391939	0.19718965	0.18127232
3.2435E-06	3.4704E-06	0.3219733	0.30422113	0.29051111	0.27180092	0.25878179	0.24221216	0.22444732	0.20958894	0.19089701	0.17592954	0.16127902
3.2435E-06	3.7714E-06	0.29895497	0.2815652	0.26723278	0.24893112	0.23694081	0.2212925	0.20483366	0.19110707	0.1741517	0.16046981	0.14675127
3.2435E-06	4.0702E-06	0.28235891	0.26526102	0.25050552	0.23253273	0.22128131	0.20630123	0.19079474	0.17788403	0.16218596	0.14942661	0.13638493
3.2435E-06	4.6609E-06	0.26122945	0.24458061	0.22935144	0.21188428	0.20156623	0.18744696	0.17318012	0.16130784	0.14722386	0.13562801	0.12346051
3.2435E-06	5.2428E-06	0.24880085	0.23250045	0.21706342	0.19998828	0.19021127	0.17660928	0.16310119	0.15183936	0.13871932	0.12779587	0.11615608
3.2435E-06	5.816E-06	0.24066746	0.22466227	0.20914561	0.19240243	0.18297311	0.16971835	0.15673036	0.14586773	0.13339008	0.12289707	0.1116134
3.2435E-06	6.3808E-06	0.23487402	0.21913207	0.20360311	0.1871558	0.17796912	0.16496857	0.15236962	0.1417911	0.12978015	0.1195862	0.10856469
3.2435E-06	6.9373E-06	0.23047573	0.21497543	0.19947224	0.18329649	0.17429005	0.16148792	0.149199	0.138836	0.12718655	0.1172137	0.10639792
3.2435E-06	7.4857E-06	0.22697251	0.21169797	0.19624322	0.18032111	0.17145509	0.15881531	0.14678499	0.13659352	0.12523774	0.11543624	0.10478966
3.2435E-06	8.0262E-06	0.22407822	0.2090169	0.19362457	0.17794196	0.16918942	0.15668724	0.14487994	0.13483005	0.1237215	0.11405777	0.10355527
3.2435E-06	1.0959E-05	0.21336349	0.19935178	0.18440901	0.16990521	0.16154808	0.14958908	0.13869869	0.12917187	0.11902337	0.10983261	0.0999052
3.2435E-06	1.3871E-05	0.20642136	0.19333018	0.17888048	0.16541032	0.15728659	0.14571072	0.13549876	0.1263098	0.11682474	0.10790616	0.09838992
3.2435E-06	1.6763E-05	0.20106582	0.18880108	0.17482936	0.16228711	0.15433243	0.14306721	0.13341941	0.1244906	0.11553738	0.10681222	0.09763184
3.2435E-06	2.2484E-05	0.19275758	0.18192616	0.16882285	0.15788982	0.15018304	0.13941939	0.13069984	0.12217396	0.11407185	0.10562521	0.09699009
3.2435E-06	2.8124E-05	0.18629265	0.17666517	0.16431199	0.15473062	0.14720834	0.13684648	0.12888032	0.12066759	0.11324302	0.10500048	0.09680387
3.2435E-06	3.917E-05	0.17651892	0.16880314	0.15766084	0.15022568	0.14297366	0.13323109	0.12643604	0.11869635	0.11231186	0.10436484	0.09684585
3.2435E-06	4.9913E-05	0.16934067	0.16307395	0.15285877	0.14705042	0.13999262	0.13071074	0.12479158	0.11739912	0.11178627	0.10404988	0.09703578
3.2435E-06	6.0366E-05	0.16380311	0.15867157	0.14918612	0.14465219	0.13774257	0.12881833	0.12358095	0.11645627	0.11144166	0.1038653	0.09724347

3.2435E-06	8.5303E-05	0.15422455	0.15108141	0.14287906	0.1405774	0.13392175	0.12561936	0.12156998	0.11490841	0.11093289	0.1036298	0.09769047
3.2435E-06	0.00010866	0.14808888	0.14623147	0.13886114	0.1380029	0.13150879	0.12360629	0.12032213	0.11395714	0.11064931	0.10351954	0.09801934
3.2435E-06	0.00013059	0.14382052	0.14286172	0.1360737	0.13622428	0.12984216	0.12221839	0.11946803	0.11330936	0.11046672	0.10345693	0.09826288

Table S9a fitted data for the fluorescence titration measurement 3 of **(±)-Zr(1)$_2$** with **G1**

[H] [M]	[G] [M]	[G]/[H]	645.0 nm	646.0 nm	647.0 nm	648.0 nm	649.0 nm	650.0 nm	651.0 nm	652.0 nm	653.0 nm	654.0 nm
3.2435E-06	0	0	0.677933	0.73343849	0.79022082	0.83070452	0.85774373	0.89837765	0.93307796	0.96672675	0.99887337	0.98092234
3.2435E-06	3.2806E-07	0.10114466	0.63631197	0.68723758	0.73793217	0.77486241	0.79905425	0.8353335	0.86569533	0.89498298	0.92274333	0.90583842
3.2435E-06	6.5351E-07	0.20148659	0.59523858	0.64164507	0.68633333	0.71975744	0.74113979	0.77312279	0.79920434	0.82418955	0.84762252	0.83175014
3.2435E-06	9.7639E-07	0.30103529	0.55478636	0.59674276	0.63551732	0.66548912	0.68410501	0.71185826	0.73372584	0.75447548	0.7736481	0.75879288
3.2435E-06	1.2967E-06	0.39980015	0.51506987	0.55265805	0.58562902	0.61221241	0.62811296	0.65171555	0.66944799	0.68604167	0.70103368	0.68717744
3.2435E-06	1.6146E-06	0.49779039	0.47627303	0.50959555	0.53690109	0.56017617	0.5734254	0.59297662	0.60667293	0.61921066	0.63012219	0.61724229
3.2435E-06	1.9299E-06	0.59501507	0.43870087	0.46789453	0.48971926	0.50979299	0.52047637	0.53610901	0.54590158	0.55451719	0.56148214	0.54954854
3.2435E-06	2.2428E-06	0.69148314	0.40287179	0.4281316	0.4447391	0.46176407	0.47000351	0.48190724	0.48798522	0.49287002	0.49607972	0.48504971
3.2435E-06	2.5533E-06	0.7872034	0.36966201	0.39128137	0.40306866	0.41727458	0.42325354	0.43171419	0.43436262	0.43580501	0.43554777	0.42535736
3.2435E-06	2.8613E-06	0.88218451	0.34042485	0.35884909	0.36641925	0.37815487	0.38215191	0.38760374	0.38725571	0.38569404	0.38240794	0.37296015
3.2435E-06	3.167E-06	0.97643499	0.31671746	0.33256666	0.33675969	0.34651076	0.34891376	0.35196174	0.34922057	0.34526555	0.33956088	0.33072081
3.2435E-06	3.4704E-06	1.06996325	0.29926072	0.31323577	0.31500107	0.32331661	0.32456399	0.32589186	0.32143945	0.31578132	0.30834783	0.29996298
3.2435E-06	3.7714E-06	1.16277754	0.28719449	0.29989954	0.3000551	0.30740822	0.30787786	0.3080746	0.30249835	0.29573156	0.28716341	0.27910216
3.2435E-06	4.0702E-06	1.25488603	0.27887412	0.29072855	0.28984158	0.29656053	0.29651462	0.29598852	0.28969554	0.28223199	0.27294099	0.26511173
3.2435E-06	4.6609E-06	1.43701753	0.26839148	0.2792323	0.27718789	0.283176	0.28252856	0.28122383	0.27416256	0.26597773	0.25591376	0.24839721
3.2435E-06	5.2428E-06	1.61642047	0.26183417	0.27209707	0.26947948	0.2750763	0.27409898	0.27243487	0.26502319	0.25653852	0.24612418	0.23882299
3.2435E-06	5.816E-06	1.79315571	0.2570494	0.26692851	0.26399456	0.26935035	0.26816366	0.26632356	0.25874413	0.25014292	0.23956285	0.23243216
3.2435E-06	6.3808E-06	1.9672823	0.25320085	0.26279661	0.2596763	0.26486796	0.26353381	0.2616098	0.25395422	0.24532759	0.23467432	0.22768969
3.2435E-06	6.9373E-06	2.1388576	0.24991289	0.25928376	0.25605039	0.261122	0.25967608	0.25771946	0.25003868	0.24143666	0.23076163	0.22390783
3.2435E-06	7.4857E-06	2.30793725	0.24699454	0.25617774	0.25287609	0.25785514	0.25631986	0.25436144	0.24668595	0.23813795	0.22747188	0.2207384
3.2435E-06	8.0262E-06	2.47457532	0.24433885	0.25335975	0.25001877	0.25492356	0.253314	0.25137324	0.24372227	0.23524631	0.22460855	0.21798755
3.2435E-06	1.0959E-05	3.37888655	0.23205441	0.24039913	0.23707665	0.24172542	0.23983385	0.23814447	0.23077985	0.22283823	0.21250827	0.20643383
3.2435E-06	1.3871E-05	4.27671526	0.22174259	0.22957482	0.22641589	0.23091442	0.22883166	0.22747859	0.22048212	0.21313757	0.20319667	0.19760042
3.2435E-06	1.6763E-05	5.16813091	0.21257963	0.21997718	0.21701923	0.22140855	0.21917303	0.21816591	0.2115446	0.20478646	0.19524039	0.19007633
3.2435E-06	2.2484E-05	6.93199592	0.19665014	0.20331582	0.20077125	0.20499866	0.20251724	0.20216571	0.19625184	0.19057734	0.18177414	0.17736984
3.2435E-06	2.8124E-05	8.67101776	0.18311326	0.18916975	0.18701074	0.19111553	0.18843569	0.18867028	0.18338717	0.17866792	0.17052628	0.16677209
3.2435E-06	3.917E-05	12.0766022	0.1611812	0.16626318	0.16476242	0.1686832	0.16569221	0.16690485	0.16267265	0.15953478	0.1524946	0.14979812
3.2435E-06	4.9913E-05	15.3888829	0.14413193	0.14846232	0.14748925	0.15127398	0.14804604	0.15003252	0.14663108	0.14473858	0.13856876	0.1366966
3.2435E-06	6.0366E-05	18.6116426	0.13049127	0.13422259	0.13367777	0.13735628	0.13394061	0.13655134	0.13381979	0.13292974	0.12746161	0.12624976
3.2435E-06	8.5303E-05	26.2999254	0.10594566	0.10860221	0.10883671	0.1123279	0.10857712	0.11231853	0.11079991	0.11172245	0.10752467	0.10750215
3.2435E-06	0.00010866	33.5016081	0.08960264	0.09154516	0.09230278	0.0956711	0.09169849	0.09619631	0.09548895	0.09762261	0.09427438	0.0950443
3.2435E-06	0.00013059	40.2614697	0.07794835	0.07938224	0.08051436	0.08379571	0.07966536	0.08470383	0.08457625	0.08757503	0.08483393	0.08616913

Table S9b fitted data for the fluorescence titration measurement 3 of **(±)-Zr(1)$_2$** with **G1**

[H] [M]	[G] [M]	655.0 nm	656.0 nm	657.0 nm	658.0 nm	659.0 nm	660.0 nm	661.0 nm	662.0 nm	663.0 nm	664.0 nm	665.0 nm
3.2435E-06	0	0.99098693	1	0.98955986	0.94464473	0.91212258	0.84625207	0.80381553	0.76438336	0.69468229	0.64894096	0.60935857
3.2435E-06	3.2806E-07	0.913789	0.92019966	0.90938134	0.86795863	0.83724193	0.7768732	0.73752507	0.70062167	0.63683661	0.59462667	0.5579774
3.2435E-06	6.5351E-07	0.83761527	0.84145902	0.83026808	0.79229147	0.76335667	0.70841652	0.67211598	0.63770814	0.57976047	0.54103497	0.50728013
3.2435E-06	9.7639E-07	0.76260513	0.76392251	0.7523654	0.71778225	0.69060268	0.6410079	0.60770865	0.57575868	0.52355904	0.48826466	0.45736032
3.2435E-06	1.2967E-06	0.68897563	0.687815	0.67589955	0.6446474	0.61919155	0.57484336	0.54449046	0.51495375	0.46839609	0.43646952	0.40836365
3.2435E-06	1.6146E-06	0.6170752	0.61349752	0.60123374	0.57323437	0.5494629	0.51023751	0.48276226	0.45558307	0.41453452	0.38589644	0.36052392
3.2435E-06	1.9299E-06	0.54748151	0.54156857	0.52897008	0.50411916	0.4819798	0.44771195	0.42302277	0.39812688	0.36241011	0.33695467	0.31422877
3.2435E-06	2.2428E-06	0.48117624	0.47304524	0.46013179	0.43828057	0.41769905	0.38815305	0.36611947	0.34340129	0.31276343	0.29033968	0.27013686
3.2435E-06	2.5533E-06	0.41981845	0.40964637	0.39644809	0.37737277	0.35823765	0.33305872	0.31348458	0.29278548	0.26684597	0.24722676	0.22936131
3.2435E-06	2.8613E-06	0.36597019	0.35402632	0.3405893	0.32395021	0.30609257	0.28474211	0.2673298	0.24840918	0.22659033	0.20943082	0.19362101
3.2435E-06	3.167E-06	0.32257855	0.30923828	0.29562693	0.28095107	0.26413592	0.24586383	0.230199	0.21272201	0.19421933	0.17903931	0.16489294
3.2435E-06	3.4704E-06	0.29100605	0.27669359	0.26298067	0.24973345	0.2336951	0.2176536	0.20326788	0.18685601	0.17076021	0.1570169	0.14409065
3.2435E-06	3.7714E-06	0.26962128	0.25470175	0.24094965	0.22867023	0.21317946	0.19863789	0.18512747	0.16945426	0.15498165	0.14220723	0.13011884
3.2435E-06	4.0702E-06	0.25530822	0.24003419	0.22628573	0.21465423	0.19955155	0.18600292	0.17308735	0.1579259	0.14453259	0.13240241	0.12088636
3.2435E-06	4.6609E-06	0.23827667	0.22270422	0.20903121	0.19817109	0.18358167	0.17118838	0.1590021	0.14449098	0.13236502	0.12099133	0.1101838
3.2435E-06	5.2428E-06	0.22859058	0.21297476	0.19941745	0.18899643	0.17475156	0.16298856	0.15123898	0.13714006	0.12571752	0.11476368	0.10438725
3.2435E-06	5.816E-06	0.22217651	0.20662582	0.19319923	0.18306927	0.16909168	0.15772614	0.14628206	0.13248756	0.12151794	0.11083443	0.10076436
3.2435E-06	6.3808E-06	0.21745438	0.20202077	0.18873023	0.17881478	0.16506278	0.15397521	0.14276804	0.12922083	0.11857517	0.10808501	0.09825589
3.2435E-06	6.9373E-06	0.21371636	0.19842664	0.18527331	0.17552782	0.16197574	0.15109735	0.14008662	0.12675234	0.11635608	0.10601478	0.0963878
3.2435E-06	7.4857E-06	0.21060421	0.19547265	0.18245567	0.1728518	0.15948216	0.1487698	0.13792929	0.12478513	0.11459126	0.10437074	0.09492061
3.2435E-06	8.0262E-06	0.20791857	0.19295261	0.18007007	0.17058851	0.15738845	0.14681318	0.13612461	0.1231543	0.11313108	0.1030124	0.09372136
3.2435E-06	1.0959E-05	0.1967813	0.18277175	0.17060209	0.1616286	0.14924414	0.13918023	0.12916865	0.11700961	0.10765707	0.09793861	0.08936736
3.2435E-06	1.3871E-05	0.18838231	0.17531621	0.1638122	0.15522256	0.14354579	0.13382041	0.12435797	0.11288522	0.10400797	0.0945731	0.08659417
3.2435E-06	1.6763E-05	0.18127616	0.16910126	0.15821401	0.14994944	0.13891018	0.12945151	0.12046984	0.10960892	0.10112102	0.09191846	0.08446139
3.2435E-06	2.2484E-05	0.16933287	0.15876809	0.14898213	0.14126424	0.13134324	0.12230902	0.11415488	0.10435988	0.09651097	0.08768964	0.08113466
3.2435E-06	2.8124E-05	0.15940335	0.1502394	0.14140487	0.13414169	0.12517632	0.1164818	0.10902647	0.10013852	0.09281232	0.08430284	0.07851169
3.2435E-06	3.917E-05	0.14353124	0.13666878	0.12939098	0.12285485	0.11544299	0.10727826	0.10095079	0.0935336	0.08703439	0.07901829	0.07446199
3.2435E-06	4.9913E-05	0.13129546	0.12623729	0.12017692	0.11420135	0.10799964	0.10023694	0.09478416	0.08851088	0.08264506	0.07500684	0.07140918
3.2435E-06	6.0366E-05	0.12154472	0.11793586	0.11285227	0.10732345	0.10209088	0.09464614	0.0898924	0.08453453	0.07917189	0.07183387	0.0690027
3.2435E-06	8.5303E-05	0.10405472	0.10306209	0.09974011	0.09501269	0.09152541	0.08464746	0.08115047	0.07744017	0.07297781	0.06617687	0.06472428
3.2435E-06	0.00010866	0.09243664	0.09318998	0.09104283	0.08684779	0.08452321	0.07802006	0.0753593	0.07274612	0.06888068	0.06243586	0.06190082
3.2435E-06	0.00013059	0.08416115	0.08616095	0.08485226	0.08103643	0.07954121	0.07330443	0.07123979	0.06940904	0.0659684	0.05977701	0.05989615

CD titration and fits for **(+)-Zr(1)$_2$** with **G1**

Table S10 Fitted *K* values, error in the fit of the *K* values and the link to the fits for CD titrations

Measurement	K_{11} [M^{-1}]	K_{12} [M^{-1}]	K_{11} error [%]	K_{12} error [%]	Link to the fits
1	1.57 * 10^6	3.20 * 10^3	22	8.7	http://app.supramolecular.org/bindfit/view/f51cb4ac-ebb5-42b4-819f-95286bc55778
2	1.74 * 10^6	2.47 * 10^3	29	9.5	http://app.supramolecular.org/bindfit/view/00ab7035-0241-48e3-adc1-c0c56291d493
Total	1.66 ± 0.12 * 10^6	2.84 ± 0.51* 10^3			α =0.0069 ± 0.0017

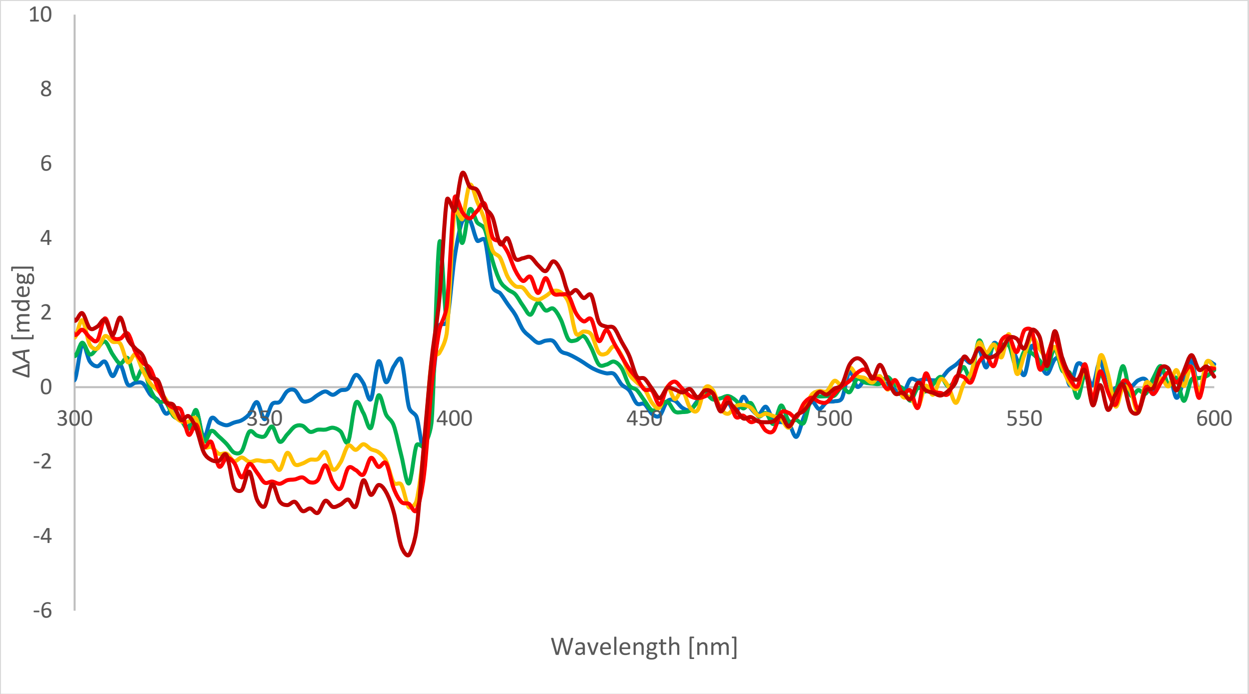

Figure S11. Selected spectra of the CD titration of **(+)-Zr(1)$_2$** with **G1** in MeCN:CH_2Cl_2. Host concentration 7.91 µM with 0 (blue), 0.5 (green), 1.0 (yellow), 2.0 (red), 10 (brown) equivalents of guest.

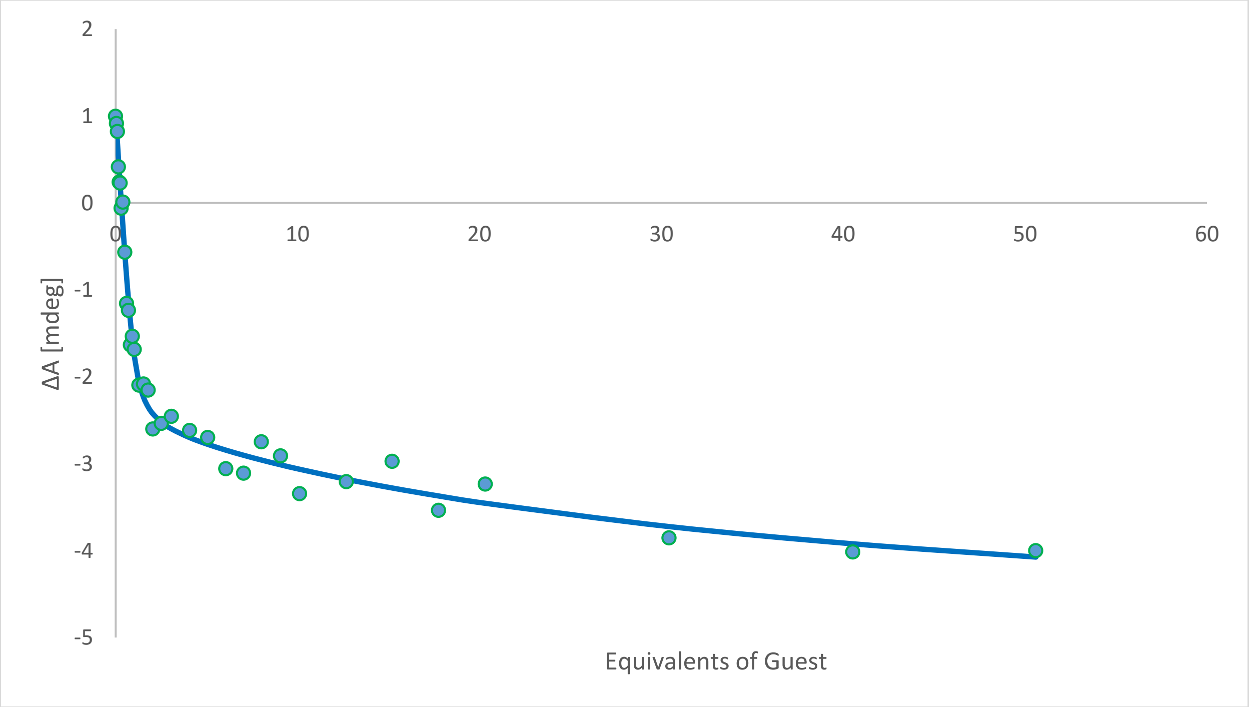

Figure S12. CD titration curve for **(+)-Zr(1)$_2$** with **G1** in MeCN/CH_2Cl_2 1:1, v/v at 382 nm and the corresponding fit assuming a 1:2 host-guest binding stoichiometry.

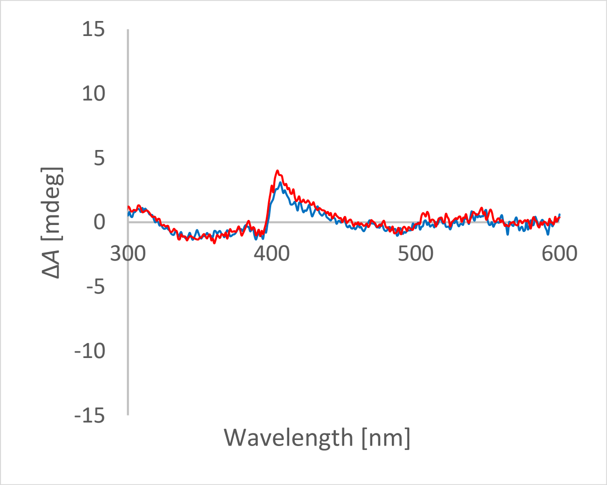

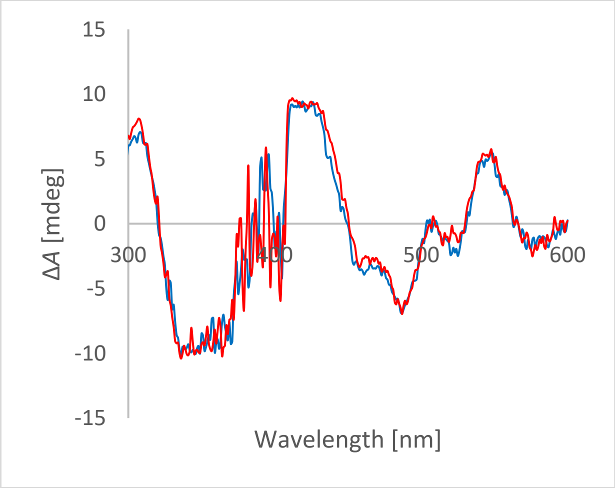

Figure S12. CD signal before (blue) and after (red) heating **(+)-Zr(1)$_2$** at reflux temperature in toluene for 18 h at concentrations of 4.6 µM (left) and 46 µM (right). The maximum absorbance around the Soret band causes a large amount of noise at that region at the higher concentration.

Table S11: fitted data for the CD titration measurement 1 of **(+)-Zr(1)$_2$** with **G1**. A value of 7 was added to all data points since the fitting program is not able to handle negative values

			observed					fitted				
**[H] [M]**	**[G] [M]**	**[G]/[H]**	**384.0 nm**	**382.0 nm**	**380.0 nm**	**378.0 nm**	**376.0 nm**	**384.0 nm**	**382.0 nm**	**380.0 nm**	**378.0 nm**	**376.0 nm**
7.898E-06	0	0	7.73766	7.99556	7.31187	7.42626	7.44601	7.53141665	8.0083188	7.71054829	7.65690172	7.4836267
7.8983E-06	4.0516E-07	0.05129718	7.20443	7.91309	7.78694	7.67631	7.55218	7.36225447	7.83751456	7.56880474	7.52167012	7.34988721
7.8986E-06	8.0871E-07	0.10238615	7.15427	7.82092	7.67422	7.20714	7.33576	7.19510985	7.66875831	7.42874723	7.38804642	7.21773576
7.8989E-06	1.2107E-06	0.15326817	6.67728	7.41348	7.14501	6.92423	7.07242	7.030139	7.50220907	7.2905061	7.25615489	7.08729496
7.8992E-06	1.611E-06	0.2039445	6.61908	7.23987	7.12941	7.53088	6.73478	6.86752152	7.33804964	7.15423122	7.12613843	6.95870588
7.8995E-06	2.0098E-06	0.25441638	7.03969	7.22775	7.27952	7.30744	6.89418	6.70746363	7.17648996	7.0200947	6.99816112	6.83213044
7.8998E-06	2.407E-06	0.30468505	7.10199	6.93864	6.64058	6.83794	6.56969	6.55020155	7.01777045	6.88829364	6.87241092	6.70775413
7.9004E-06	3.1966E-06	0.40461764	6.358	7.00866	6.22589	6.83566	6.45605	6.24517841	6.70998544	6.6326273	6.62847789	6.46647346
7.901E-06	3.9801E-06	0.50375196	6.10925	6.43225	6.7784	6.1826	6.08544	5.95504907	6.41733781	6.38939947	6.39640573	6.2369033
7.9015E-06	4.7575E-06	0.60209752	5.33087	5.843	6.20591	6.32476	6.01352	5.68299715	6.14306776	6.16126707	6.1787277	6.02154393
7.9021E-06	5.6056E-06	0.70937782	5.00559	5.76301	6.07841	5.8044	5.73697	5.40892874	5.86698247	5.93135314	5.95933618	5.80444652
7.9028E-06	6.4465E-06	0.81572734	5.24353	5.36486	5.86345	5.77644	5.68979	5.16578515	5.62234523	5.72725878	5.76456409	5.61165302
7.9034E-06	7.2803E-06	0.92115816	4.66262	5.46422	5.69569	5.30614	5.7496	4.95687546	5.41252295	5.55174608	5.59704504	5.44576291
7.904E-06	8.107E-06	1.02568211	4.69086	5.31343	5.59253	5.88883	5.24174	4.78282417	5.23814994	5.40533684	5.45727662	5.30726757
7.9055E-06	1.0107E-05	1.278469	4.39492	4.90411	5.12482	5.16326	5.13254	4.48329827	4.93997421	5.15258923	5.21587483	5.0676917
7.9069E-06	1.2138E-05	1.53509988	4.69901	4.91531	4.57567	4.64889	5.17598	4.30109516	4.76124991	4.99773868	5.06781117	4.92022935
7.9085E-06	1.4197E-05	1.79516105	4.61729	4.84663	4.61832	5.05652	4.76428	4.18354952	4.64835253	4.89684318	4.97119053	4.82353579
7.9099E-06	1.6212E-05	2.04957445	4.06663	4.39825	4.88319	4.72225	4.60443	4.10290505	4.57279511	4.82683719	4.90403587	4.75596722
7.9102E-06	1.9869E-05	2.51187673	4.57518	4.46351	4.46376	4.68247	4.44279	3.99861529	4.47818265	4.73454641	4.81522202	4.666033
7.9105E-06	2.4241E-05	3.0643639	4.03612	4.54447	4.84152	5.08158	4.6089	3.90917147	4.40061249	4.65376607	4.73724589	4.58641361
7.911E-06	3.2205E-05	4.07086229	3.75861	4.38123	4.39842	4.57384	4.33269	3.78683971	4.2997809	4.54089018	4.62794402	4.47385979
7.9116E-06	4.0105E-05	5.06917082	3.42354	4.30111	4.41447	4.54396	4.43926	3.68843574	4.22207987	4.44854498	4.53830495	4.38095406
7.9121E-06	4.7943E-05	6.05938905	3.82115	3.94128	4.4964	4.45946	4.24927	3.60227303	4.15570577	4.36693576	4.45898338	4.29845495
7.9127E-06	5.5718E-05	7.04161491	3.3037	3.88922	4.38618	4.18067	4.13852	3.52401292	4.0963252	4.29240373	4.38648488	4.2228973
7.9132E-06	6.3432E-05	8.01594479	3.22098	4.25138	4.26681	4.41392	4.08319	3.4515989	4.04191995	4.22319822	4.31913492	4.1526136
7.9138E-06	7.1777E-05	9.06995617	3.31838	4.0894	4.139	4.59326	4.07685	3.37792734	3.98695706	4.15261963	4.25042564	4.08084563
7.9143E-06	8.0052E-05	10.1148046	3.21928	3.65428	4.0398	3.86131	4.11411	3.30891219	3.93573422	4.0863858	4.18593055	4.01343426
7.9157E-06	0.00010043	12.6876132	3.17092	3.79159	4.16863	4.22318	4.15889	3.15298644	3.82067213	3.93646091	4.03990416	3.86069168
7.9171E-06	0.00012039	15.2056949	2.73943	4.02709	4.12421	4.05178	3.51899	3.01623773	3.72027911	3.8047655	3.91160626	3.72640425
7.9185E-06	0.00014057	17.7520537	2.50547	3.46346	3.36288	3.93615	3.9231	2.89105094	3.62866059	3.68409944	3.79404028	3.60329999
7.9199E-06	0.00016096	20.3231284	2.60056	3.76432	3.68237	3.64447	3.34854	2.77597743	3.54462355	3.57312394	3.68590942	3.49004343
7.9254E-06	0.00024113	30.4246805	2.48949	3.14667	3.11336	3.4742	3.39019	2.40799695	3.2767197	3.21807344	3.33994707	3.12752818
7.931E-06	0.00032148	40.5353858	2.57324	2.98298	2.96018	2.85878	2.61705	2.13424494	3.07806706	2.95392761	3.08257568	2.8577133
7.9365E-06	0.00040155	50.5951922	1.99282	2.99869	2.68719	2.81978	2.59251	1.92361155	2.92558826	2.75076658	2.88464914	2.65013685

Table S12: fitted data for the CD titration measurement 2 of **(+)-Zr(1)$_2$** with **G1**. A value of 7 was added to all data points since the fitting program is not able to handle negative values

			observed					fitted				
**[H] [M]**	**[G] [M]**	**[G]/[H]**	**384.0 nm**	**382.0 nm**	**380.0 nm**	**378.0 nm**	**376.0 nm**	**384.0 nm**	**382.0 nm**	**380.0 nm**	**378.0 nm**	**376.0 nm**
7.898E-06	0	0	7.55594	7.13293	7.69381	6.67882	7.1081	7.52350562	7.70283204	7.829796	7.23803374	7.16691872
7.8983E-06	4.0516E-07	0.05129718	7.26301	7.92785	7.76382	7.29912	7.04102	7.3585954	7.55473621	7.68484676	7.11761941	7.05286818
7.8986E-06	8.0871E-07	0.10238615	6.96195	7.63034	7.6133	7.16113	7.08725	7.19554084	7.4083053	7.54152791	6.9985518	6.94009186
7.8989E-06	1.2107E-06	0.15326817	7.2827	7.43768	7.25846	6.6651	6.8881	7.03448935	7.26367197	7.39996951	6.88093835	6.8286915
7.8992E-06	1.611E-06	0.2039445	7.50131	6.83914	7.23122	6.82812	6.48691	6.87560825	7.12098629	7.2603186	6.76490016	6.71878161
7.8995E-06	2.0098E-06	0.25441638	6.56317	7.17499	7.03053	6.86796	6.70998	6.71909133	6.98042213	7.1227455	6.65057749	6.61049486
7.8998E-06	2.407E-06	0.30468505	6.49096	6.52646	7.20606	6.44539	6.64139	6.56516274	6.84218055	6.98744718	6.53813259	6.50398469
7.9004E-06	3.1966E-06	0.40461764	6.25155	6.88948	6.74295	6.43501	5.78729	6.26614277	6.57362704	6.72461722	6.31965226	6.29702812

7.901E-06	3.9801E-06	0.50375196	5.9728	6.26303	6.77996	5.90727	6.30834	5.98109336	6.3176089	6.47406538	6.11130144	6.09965426
7.9015E-06	4.7575E-06	0.60209752	5.77764	6.61351	5.97478	6.13267	5.82264	5.71323429	6.07701456	6.23862131	5.91541096	5.91406752
7.9021E-06	5.6056E-06	0.70937782	5.0463	5.67328	5.71116	6.06522	5.77872	5.44298006	5.83424444	6.0010686	5.71760685	5.72664185
7.9028E-06	6.4465E-06	0.81572734	5.04307	5.38431	5.78708	5.49006	5.84385	5.20329987	5.61890519	5.7903858	5.54195557	5.56017072
7.9034E-06	7.2803E-06	0.92115816	4.98099	5.14552	6.24846	5.45217	5.29964	4.99813191	5.43452987	5.61003398	5.39130867	5.41735109
7.904E-06	8.107E-06	1.02568211	4.43502	5.02032	5.25366	5.33728	5.46814	4.82855363	5.28208519	5.46096006	5.26644694	5.29892156
7.9055E-06	1.0107E-05	1.278469	4.52944	5.1766	5.34612	5.17523	5.52059	4.54258711	5.02478331	5.20954054	5.05436863	5.09752677
7.9069E-06	1.2138E-05	1.53509988	4.25175	4.98416	5.07525	5.18882	4.80025	4.37413972	4.8729024	5.0614014	4.92732501	4.97654831
7.9085E-06	1.4197E-05	1.79516105	4.03633	4.53323	4.66794	4.91664	5.17446	4.26800393	4.77691857	4.96802513	4.84536627	4.89820569
7.9099E-06	1.6212E-05	2.04957445	4.28132	4.95739	4.85898	5.10088	4.65343	4.19622132	4.7117775	4.90484457	4.78843296	4.84355657
7.9102E-06	1.9869E-05	2.51187673	4.49531	4.44988	4.31307	4.85388	4.56384	4.10414442	4.627552	4.82338345	4.71233639	4.77005061
7.9105E-06	2.4241E-05	3.0643639	4.4799	4.61245	5.20473	4.52525	4.38006	4.02556703	4.55515605	4.7536932	4.64437699	4.70397432
7.911E-06	3.2205E-05	4.07086229	3.99328	4.62569	4.75535	4.29534	4.39971	3.91796863	4.45525791	4.65801157	4.54686662	4.60855946
7.9116E-06	4.0105E-05	5.06917082	4.13941	4.36359	4.3515	4.1609	4.59564	3.83084377	4.37387297	4.58037361	4.46502045	4.52809653
7.9121E-06	4.7943E-05	6.05938905	3.61981	4.13286	4.72181	4.33143	4.41366	3.75392626	4.30178238	4.51175386	4.39135439	4.45549883
7.9127E-06	5.5718E-05	7.04161491	3.69152	4.10474	4.28093	4.13584	4.57333	3.68346875	4.23561601	4.4488561	4.32310744	4.38814724
7.9132E-06	6.3432E-05	8.01594479	3.803	3.91752	4.31437	4.09173	3.91806	3.61772951	4.17380362	4.39014694	4.2589742	4.32479994
7.9138E-06	7.1777E-05	9.06995617	3.71339	4.33701	4.15203	4.12275	4.08085	3.55028426	4.11033265	4.32989843	4.19284998	4.25944669
7.9143E-06	8.0052E-05	10.1148046	3.6495	4.18193	4.37322	4.10851	4.49861	3.48656721	4.05033347	4.27297044	4.13015752	4.19745839
7.9157E-06	0.00010043	12.6876132	2.77645	3.80379	4.29083	3.86055	3.80603	3.34063445	3.9128272	4.14256676	3.98601661	4.05487037
7.9171E-06	0.00012039	15.2056949	2.89499	3.83035	4.20894	3.41507	4.18808	3.21030755	3.78996191	4.02609944	3.85686755	3.9270627
7.9185E-06	0.00014057	17.7520537	2.78352	3.85123	3.94261	4.13858	4.10202	3.08900602	3.6755755	3.91769968	3.73643957	3.80785944
7.9199E-06	0.00016096	20.3231284	3.21988	3.82021	3.91797	3.45996	3.89839	2.97576841	3.56877921	3.81651312	3.62388687	3.69643626
7.9254E-06	0.00024113	30.4246805	2.51523	3.42168	3.59769	3.15464	3.60189	2.60193267	3.2161925	3.48255549	3.25180748	3.32803427
7.931E-06	0.00032148	40.5353858	2.51226	3.22555	3.1021	3.12912	2.81374	2.31151131	2.94233679	3.22327377	2.96250281	3.04156421
7.9365E-06	0.00040155	50.5951922	2.06771	2.12298	2.89471	2.9522	2.60341	2.08039782	2.72449072	3.01709465	2.73224665	2.81356457

UV-Vis titration for **(±)-Zr(1)$_2$** with **G1**

Table S13. Fitted *K* values, error in the fit of the *K* values and the link to the fits of the UV-Vis titration with **G1**

Measurement	K_{11} [M^{-1}]	K_{12} [M^{-1}]	K_{11} error [%]	K_{12} error [%]	Link to the fits
1	1869260.27	5714.28	4.19	3.50	http://app.supramolecular.org/bindfit/view/99845dc9-b386-4309-bb61-8f3c35af3013
2	1812146.69	3762.94	5.85	3.92	http://app.supramolecular.org/bindfit/view/438184d2-4f80-4b5f-bdc1-fd59799ab262
3	2653332.15	5528.86	4.25	2.75	http://app.supramolecular.org/bindfit/view/1d0c15cb-91f8-4031-afb2-782e4d30d72d

Table S14a measured data for the UV-Vis titration measurement 1 of **(±)-Zr(1)$_2$** with **G1**

[H] [M]	[G] [M]	410 nm	409 nm	408 nm	407 nm	406 nm	405 nm	404 nm	403 nm	402 nm	401 nm	400 nm
2.0257E-06	0	0.12884035	0.15154906	0.176858	0.21639962	0.25307497	0.30498596	0.3668116	0.45313191	0.52193569	0.60254216	0.65370166
2.0257E-06	1.9374E-07	0.11862984	0.14291664	0.16899984	0.20796409	0.24859932	0.30365685	0.3661674	0.4495998	0.5218764	0.6015119	0.65481395
2.0257E-06	3.87E-07	0.13082291	0.15271886	0.18021748	0.21851617	0.25598124	0.31256536	0.37306547	0.45568192	0.52407098	0.60234797	0.64912433
2.0257E-06	5.7978E-07	0.13130467	0.15628217	0.18301065	0.22342859	0.2600505	0.31558877	0.37711766	0.45946985	0.52716049	0.60324576	0.65136388
2.0257E-06	7.7208E-07	0.13365125	0.15761591	0.18396046	0.22506913	0.2618761	0.31569614	0.3743945	0.4569227	0.52479022	0.60407209	0.64911127
2.0257E-06	9.6391E-07	0.13586662	0.15931376	0.18597696	0.22504033	0.26355309	0.32225988	0.38265291	0.46046013	0.52907086	0.60539574	0.6497286
2.0257E-06	1.1553E-06	0.13704192	0.16112078	0.1888897	0.22799825	0.26587136	0.32230769	0.38348513	0.46367948	0.53025677	0.60362907	0.64895089
2.0257E-06	1.5361E-06	0.13968643	0.16447236	0.19235251	0.23216859	0.27166905	0.32874354	0.38467412	0.46609741	0.53610908	0.60562824	0.646578
2.0257E-06	1.9151E-06	0.14161258	0.16873989	0.19425288	0.23591693	0.27542961	0.33258036	0.39125258	0.47165617	0.54037741	0.6074343	0.64821413
2.0257E-06	2.2922E-06	0.14495344	0.16927311	0.19799962	0.23996983	0.2778418	0.33328396	0.39420789	0.47335308	0.53678921	0.60631355	0.6471056
2.0257E-06	2.6675E-06	0.14484413	0.17159729	0.19908541	0.24044824	0.27933097	0.3359707	0.39347166	0.47539219	0.53912973	0.60918015	0.6452446
2.0257E-06	3.041E-06	0.14714418	0.17256325	0.20174992	0.24253949	0.28362999	0.33853973	0.39771895	0.47645299	0.54144178	0.61048261	0.64497827
2.0257E-06	3.4127E-06	0.14813338	0.17321462	0.20209488	0.24391439	0.28333412	0.34073795	0.39755867	0.47841472	0.54201622	0.60774762	0.64495314
2.0257E-06	3.7827E-06	0.14914539	0.1760015	0.2051316	0.24736505	0.28530531	0.34241968	0.39845529	0.48041322	0.54072069	0.60762083	0.64539313
2.0257E-06	4.1509E-06	0.148804	0.17568472	0.20543138	0.24664443	0.28554425	0.34359727	0.39934633	0.48081726	0.5445484	0.61051625	0.64541138
2.0257E-06	5.9875E-06	0.15198057	0.17850045	0.20713628	0.24874979	0.28899005	0.34717345	0.40513199	0.48390251	0.54420676	0.60739246	0.64301964
2.0257E-06	7.8198E-06	0.15296349	0.18087154	0.20932315	0.25246144	0.29305045	0.34984608	0.40857364	0.48589231	0.5487215	0.61073805	0.64336831
2.0257E-06	9.6478E-06	0.15324979	0.18003963	0.21134477	0.25321756	0.29313775	0.3495463	0.40949567	0.48489247	0.54993443	0.61029057	0.64462696
2.0257E-06	1.1472E-05	0.1541985	0.18013421	0.21024719	0.25515135	0.29433941	0.35080658	0.40902709	0.48699592	0.55010568	0.61345177	0.64298307
2.0257E-06	1.3291E-05	0.15408069	0.1814574	0.21047549	0.25336109	0.29403179	0.35289668	0.41287391	0.49046932	0.549605	0.61279188	0.64501195
2.0257E-06	1.5106E-05	0.15500458	0.18309925	0.21215978	0.25771657	0.29577086	0.35515554	0.41198386	0.48997805	0.55189318	0.61385358	0.64581943
2.0257E-06	1.6917E-05	0.15563807	0.18213399	0.21094913	0.25575956	0.29607739	0.35329588	0.41188057	0.48942318	0.55177324	0.61150657	0.64520525
2.0257E-06	1.8724E-05	0.15585792	0.18188116	0.21341601	0.2564951	0.29619135	0.35535721	0.41329081	0.48968999	0.55172126	0.61546258	0.64668069
2.0257E-06	2.0526E-05	0.1561648	0.18318043	0.21378782	0.25739204	0.29710563	0.35508379	0.41122197	0.4932669	0.55307331	0.61628266	0.64715781
2.0257E-06	3.851E-05	0.15788238	0.18492808	0.2154985	0.25879093	0.29899769	0.35963135	0.41809874	0.49480303	0.55661093	0.61645054	0.64694088
2.0257E-06	7.4312E-05	0.15885981	0.18892522	0.21805916	0.26099084	0.30334855	0.36220232	0.42305309	0.50066141	0.56039754	0.6210281	0.64999584
2.0257E-06	0.00010995	0.16108069	0.18930228	0.2210194	0.26800719	0.30919862	0.36981133	0.42843482	0.50509506	0.56494355	0.62308365	0.65176898
2.0257E-06	0.00014542	0.16376195	0.19226917	0.22448234	0.27100406	0.3134918	0.37220905	0.43232096	0.50676651	0.56900681	0.627459	0.65432454
2.0257E-06	0.00023311	0.1658071	0.19607431	0.22920317	0.27598901	0.31750377	0.38241844	0.44149687	0.51911797	0.57833012	0.63253798	0.6570548
2.0257E-06	0.00031982	0.16908317	0.20084279	0.23262014	0.28108177	0.32466588	0.38420454	0.44657076	0.52471128	0.5828301	0.63711623	0.65881774
2.0257E-06	0.00040557	0.16903638	0.19963955	0.23308263	0.28432072	0.32957492	0.38893808	0.44964964	0.52979422	0.58567477	0.63744707	0.65985001
2.0257E-06	0.00049038	0.17009604	0.20122591	0.23634395	0.28448446	0.32700445	0.39272522	0.45097585	0.52909465	0.58487584	0.63918861	0.66072758
2.0257E-06	0.00081544	0.1713077	0.20250234	0.23708843	0.28746888	0.33193752	0.39272874	0.45608401	0.53130957	0.59032878	0.6396344	0.66189352
2.0257E-06	0.00112745	0.1710184	0.2034	0.23803848	0.28688176	0.33376901	0.3927295	0.45862157	0.53418819	0.58878921	0.64308111	0.66235803
2.0257E-06	0.00142741	0.17199963	0.20452026	0.23895415	0.28633363	0.33306188	0.39713911	0.45843721	0.53471733	0.59002174	0.64239276	0.66163696
2.0257E-06	0.00171622	0.17264022	0.2035041	0.2394984	0.28948918	0.3360776	0.3959516	0.45863616	0.53624871	0.5917094	0.6422883	0.66184494
2.0257E-06	0.00199468	0.17137206	0.20419221	0.23851801	0.28945114	0.33328884	0.39730256	0.4607056	0.53489607	0.5924068	0.64097904	0.66129719
2.0257E-06	0.0022635	0.17244662	0.20532113	0.24032943	0.2895359	0.33380254	0.40039517	0.46071966	0.53882869	0.59269382	0.64368965	0.66274531
2.0257E-06	0.00252333	0.17327093	0.20605982	0.23896553	0.28818484	0.33517085	0.39828672	0.46008672	0.53750427	0.59286507	0.64385315	0.66340085
2.0257E-06	0.00277476	0.17213556	0.20545256	0.2415713	0.29037068	0.33476214	0.39828692	0.46093998	0.53790484	0.59283916	0.64252792	0.66332714

Table S14b measured data for the UV-Vis titration measurement 1 of **(±)-Zr(1)$_2$** with **G1**

[H] [M]	[G] [M]	399 nm	398 nm	397 nm	396 nm	395 nm	394 nm	393 nm	392 nm	391 nm	390 nm
2.0257E-06	0	0.69949751	0.7174257	0.70830023	0.67250925	0.63220435	0.57710724	0.51091521	0.45208751	0.40748187	0.36412063
2.0257E-06	1.9374E-07	0.70054483	0.71776927	0.70918131	0.66991353	0.6312269	0.5781821	0.51131195	0.45545796	0.38850242	0.33913344
2.0257E-06	3.87E-07	0.69329101	0.70744044	0.69857651	0.65971691	0.62320662	0.56732738	0.49806339	0.44468445	0.40121323	0.35734952
2.0257E-06	5.7978E-07	0.69157603	0.70448968	0.6920816	0.65605792	0.61440578	0.55773166	0.49479383	0.43967909	0.39542416	0.35277745
2.0257E-06	7.7208E-07	0.69068366	0.70206373	0.68902248	0.64912456	0.61293154	0.55566757	0.4927732	0.43910282	0.39737316	0.35418856
2.0257E-06	9.6391E-07	0.68909854	0.69907749	0.68487716	0.64593399	0.60797894	0.55543024	0.48902354	0.43658057	0.39240185	0.35198889
2.0257E-06	1.1553E-06	0.68448212	0.69518389	0.68024834	0.63823416	0.60553535	0.54780628	0.48307641	0.43238978	0.39229109	0.34954404
2.0257E-06	1.5361E-06	0.68231069	0.68869286	0.67304694	0.63205861	0.5935251	0.53997563	0.47982677	0.42621972	0.38616516	0.34701448
2.0257E-06	1.9151E-06	0.67924061	0.6843842	0.66707441	0.62409124	0.5888904	0.53664813	0.47376254	0.42166972	0.38294098	0.343808
2.0257E-06	2.2922E-06	0.67657831	0.68010589	0.66161254	0.62047866	0.5820184	0.5294815	0.4722197	0.42073983	0.37879297	0.33956191
2.0257E-06	2.6675E-06	0.67554027	0.67770654	0.65920657	0.6182977	0.58212656	0.52721697	0.4675779	0.41871142	0.37925455	0.33952737
2.0257E-06	3.041E-06	0.67382709	0.67449878	0.65560405	0.61517946	0.57447497	0.52242259	0.46249793	0.41587741	0.37655894	0.33628209
2.0257E-06	3.4127E-06	0.67159457	0.67212112	0.65270282	0.61088795	0.57235188	0.51924641	0.46073405	0.41440902	0.37553791	0.33587358
2.0257E-06	3.7827E-06	0.67265337	0.67141419	0.65026575	0.60801106	0.5723837	0.51610463	0.46126252	0.4125272	0.37428814	0.33642548
2.0257E-06	4.1509E-06	0.6717524	0.66944528	0.64917213	0.60734088	0.56911785	0.51838726	0.45779029	0.41046554	0.37219578	0.33607355
2.0257E-06	5.9875E-06	0.66845157	0.66533389	0.64338108	0.59893599	0.56150421	0.51206347	0.45326617	0.40739026	0.36925312	0.33369383
2.0257E-06	7.8198E-06	0.66589178	0.66266329	0.64058949	0.59773738	0.55894036	0.50813849	0.45123724	0.40368236	0.36609067	0.33195148
2.0257E-06	9.6478E-06	0.665785	0.66150884	0.63847421	0.59232526	0.55560093	0.50695662	0.44970508	0.40471782	0.36736514	0.32980158
2.0257E-06	1.1472E-05	0.6659007	0.66115867	0.63807766	0.59502851	0.55502557	0.50511287	0.44864999	0.4020672	0.36715456	0.3306775
2.0257E-06	1.3291E-05	0.66569208	0.66013073	0.63654547	0.59322678	0.55505137	0.50480746	0.44710748	0.40110485	0.36678036	0.33073004
2.0257E-06	1.5106E-05	0.66643734	0.66069288	0.63687784	0.59080304	0.55466075	0.50089873	0.44882093	0.40220777	0.36436562	0.3300365
2.0257E-06	1.6917E-05	0.66673826	0.66104232	0.63683104	0.59329038	0.55517422	0.50594979	0.44705119	0.40177165	0.36655675	0.33228256
2.0257E-06	1.8724E-05	0.66706423	0.66065464	0.63658671	0.59106169	0.554022	0.50377111	0.44736196	0.4025685	0.36558394	0.33105887
2.0257E-06	2.0526E-05	0.66714843	0.6615571	0.6363714	0.59281709	0.55476221	0.50193407	0.44868433	0.4029172	0.36637723	0.32958295
2.0257E-06	3.851E-05	0.66735529	0.6595928	0.63472675	0.58799153	0.55171113	0.50212805	0.44452624	0.4008392	0.36482595	0.32844519
2.0257E-06	7.4312E-05	0.66713444	0.65919773	0.63340835	0.58607058	0.54854677	0.49795274	0.44321565	0.39577765	0.36043636	0.32626949
2.0257E-06	0.00010995	0.66689658	0.65458155	0.6287024	0.58010316	0.54229271	0.49156341	0.43847546	0.39369112	0.35892615	0.3231245
2.0257E-06	0.00014542	0.66622986	0.65395231	0.62323083	0.57797409	0.53832246	0.48585598	0.43170984	0.38966171	0.3546075	0.3203258

2.0257E-06	0.00023311	0.66609915	0.64972242	0.61771097	0.57001408	0.53147944	0.4804505	0.42682548	0.38212164	0.34886633	0.31572666
2.0257E-06	0.00031982	0.66618684	0.64723721	0.61204532	0.56338731	0.52420452	0.47509805	0.42181805	0.38004697	0.34471068	0.31334716
2.0257E-06	0.00040557	0.66613871	0.64614511	0.61440206	0.55939735	0.5184126	0.47083742	0.41746125	0.37742651	0.34062446	0.31175048
2.0257E-06	0.00049038	0.66729804	0.64660813	0.6113552	0.56084015	0.5236602	0.47340204	0.41823207	0.37539269	0.34255787	0.31060095
2.0257E-06	0.00081544	0.66567108	0.64307609	0.60730788	0.5590634	0.51942256	0.46759906	0.41674086	0.37287131	0.34101495	0.30604839
2.0257E-06	0.00112745	0.66491405	0.64194427	0.60722409	0.55559741	0.51683895	0.46764905	0.41603587	0.37277265	0.33782758	0.30718367
2.0257E-06	0.00142741	0.66484243	0.64165235	0.60656626	0.55660731	0.51716114	0.46455634	0.41173352	0.36957351	0.33847756	0.30606563
2.0257E-06	0.00171622	0.66527876	0.64103839	0.60603156	0.55416563	0.5135214	0.46595984	0.41086435	0.370967	0.33462289	0.30542439
2.0257E-06	0.00199468	0.66514581	0.64144175	0.60504126	0.55393641	0.51847874	0.46469682	0.41177162	0.36936169	0.33784526	0.30438872
2.0257E-06	0.0022635	0.66550888	0.6435342	0.60633166	0.55191483	0.51346256	0.46338539	0.41246872	0.37231566	0.33813353	0.30545826
2.0257E-06	0.00252333	0.66572924	0.64167865	0.60596903	0.55315455	0.51429017	0.46276708	0.41143485	0.36786597	0.33622282	0.3053089
2.0257E-06	0.00277476	0.66553376	0.64157281	0.60572867	0.55373714	0.51600931	0.46517907	0.41265551	0.37046395	0.33656321	0.30522979

Table S15a measured data for the UV-Vis titration measurement 2 of **(±)-Zr(1)$_2$** with **G1**

[H] [M]	[G] [M]	410 nm	409 nm	408 nm	407 nm	406 nm	405 nm	404 nm	403 nm	402 nm	401 nm	400 nm
2.0257E-06	0	0.16637195	0.18953688	0.21697816	0.25619596	0.29301777	0.34968534	0.40935221	0.49589127	0.56762701	0.65025371	0.70157259
2.0257E-06	1.9374E-07	0.16786221	0.19222264	0.21905397	0.25770377	0.29522054	0.35232939	0.41444399	0.4994684	0.57012506	0.65361359	0.70163043
2.0257E-06	3.87E-07	0.17000777	0.19394326	0.22216928	0.26114318	0.29755625	0.35414934	0.41811043	0.50258207	0.57195598	0.65154129	0.69733912
2.0257E-06	5.7978E-07	0.17245368	0.1958399	0.22376417	0.26374908	0.30191054	0.36047714	0.41976767	0.50189905	0.57441502	0.65164631	0.69950121
2.0257E-06	7.7208E-07	0.17379563	0.19877163	0.22641242	0.2668863	0.30627269	0.36184714	0.42277161	0.50769887	0.57689384	0.65036568	0.69849846
2.0257E-06	9.6391E-07	0.17567088	0.20019202	0.22875704	0.26885416	0.30745577	0.36398705	0.4247192	0.50932514	0.57468664	0.65247004	0.69727688
2.0257E-06	1.1553E-06	0.17732804	0.20184173	0.23080268	0.27205136	0.30920257	0.36662131	0.42545747	0.51053216	0.57799276	0.65294173	0.69769707
2.0257E-06	1.5361E-06	0.18044653	0.20547945	0.23429839	0.27428276	0.31418387	0.3714991	0.43118436	0.51360817	0.58179433	0.65478564	0.69705672
2.0257E-06	1.9151E-06	0.18271961	0.20793096	0.23775784	0.27955926	0.31870995	0.37465761	0.43412784	0.51943926	0.58319727	0.65425382	0.69701341
2.0257E-06	2.2922E-06	0.18354833	0.20890887	0.23888712	0.28058697	0.32008405	0.37824811	0.43730263	0.51729463	0.58758716	0.65448944	0.69740562
2.0257E-06	2.6675E-06	0.1853687	0.21210761	0.24106054	0.28195076	0.32229002	0.38087539	0.4395975	0.52028836	0.5880471	0.65740503	0.6953472
2.0257E-06	3.041E-06	0.18671435	0.21311333	0.24164986	0.28555753	0.32519638	0.38237035	0.44171848	0.52344438	0.58816608	0.65620025	0.69606336
2.0257E-06	3.4127E-06	0.18836385	0.21401883	0.24385671	0.28622414	0.32553429	0.3833455	0.44393031	0.52433584	0.58696971	0.6587684	0.69509492
2.0257E-06	3.7827E-06	0.18900338	0.21560033	0.24491687	0.28714376	0.32655405	0.38740011	0.44649642	0.52351688	0.5902959	0.65987067	0.69594042
2.0257E-06	4.1509E-06	0.19048922	0.21740348	0.24592761	0.28911493	0.33146135	0.3878544	0.44882933	0.52797047	0.59377114	0.66007499	0.69528697
2.0257E-06	5.9875E-06	0.19253485	0.22002389	0.24935787	0.29156663	0.33497174	0.39076398	0.45197435	0.53255399	0.59628709	0.65933539	0.69665296
2.0257E-06	7.8198E-06	0.19392819	0.22054378	0.25147007	0.29415412	0.33483434	0.39461131	0.45417217	0.53381882	0.59853689	0.66345195	0.69662098
2.0257E-06	9.6478E-06	0.19474156	0.22111357	0.25159428	0.29541985	0.33650456	0.39588455	0.45471252	0.5333323	0.5994441	0.66181446	0.69566012
2.0257E-06	1.1472E-05	0.19575707	0.22365514	0.25400383	0.29751671	0.34048313	0.39798901	0.45873347	0.53800741	0.60077265	0.66529826	0.69919846
2.0257E-06	1.3291E-05	0.19636049	0.22384449	0.25336128	0.29832571	0.33999696	0.39918801	0.45971703	0.53960537	0.60144251	0.6654371	0.69965457
2.0257E-06	1.5106E-05	0.19690316	0.22430246	0.25531569	0.2997862	0.34175598	0.40189009	0.46361267	0.5416159	0.60147529	0.6658883	0.70127165
2.0257E-06	1.6917E-05	0.19709642	0.22447525	0.25637887	0.30056794	0.33955754	0.40085698	0.46181173	0.53977532	0.60356713	0.66853918	0.7016672
2.0257E-06	1.8724E-05	0.19758329	0.2250861	0.25588304	0.30048988	0.34113026	0.4013691	0.46496422	0.54366804	0.60211778	0.67040539	0.70218558
2.0257E-06	2.0526E-05	0.19741796	0.22556509	0.2564355	0.30075867	0.34564848	0.40331133	0.46411969	0.54647829	0.60791369	0.67157151	0.70349468
2.0257E-06	3.851E-05	0.19894203	0.22661292	0.25822818	0.30301529	0.34455162	0.40445601	0.46617659	0.54484412	0.60837337	0.67416581	0.70617422
2.0257E-06	5.6452E-05	0.19995478	0.22845087	0.25969506	0.30659429	0.34876803	0.40967653	0.4678045	0.549563	0.61303366	0.67696453	0.70846111
2.0257E-06	9.2172E-05	0.20268542	0.23144952	0.26310269	0.31005681	0.35312594	0.4140513	0.47601131	0.55642036	0.61909763	0.68109278	0.7107359
2.0257E-06	0.00012773	0.2041675	0.23366045	0.26669483	0.31465601	0.35716583	0.41985504	0.48136448	0.56114577	0.62481479	0.68378244	0.71315716
2.0257E-06	0.00021561	0.20734293	0.23860998	0.27170882	0.32046584	0.36430249	0.42783645	0.49353719	0.57194889	0.63202263	0.69041801	0.71673179
2.0257E-06	0.00030251	0.20973852	0.24057126	0.27473071	0.32365951	0.37099531	0.4356049	0.4966352	0.57901448	0.63678098	0.69511163	0.71972722
2.0257E-06	0.00038845	0.21056409	0.24272273	0.27800576	0.32763703	0.37420891	0.43812852	0.50272415	0.58435564	0.64379732	0.69933049	0.72280632
2.0257E-06	0.00047345	0.21273685	0.24393547	0.28049297	0.33104376	0.37983847	0.44166336	0.50685618	0.58831129	0.64812628	0.70224092	0.72511712
2.0257E-06	0.0007992	0.21333082	0.24690859	0.28248127	0.33219074	0.38281871	0.4475096	0.5101784	0.59091134	0.64900257	0.70378389	0.7264684
2.0257E-06	0.00111183	0.21605631	0.25006869	0.28777494	0.33706709	0.38623898	0.45314165	0.51743724	0.59876063	0.65799107	0.71121737	0.73198064
2.0257E-06	0.00141237	0.22027373	0.25406665	0.29102928	0.34375879	0.39146131	0.45832709	0.52378062	0.60596791	0.66521403	0.7178655	0.73911867
2.0257E-06	0.00170172	0.22042192	0.25483114	0.2910995	0.34403572	0.39343599	0.46004555	0.52497152	0.60637293	0.66817067	0.72110317	0.74234787
2.0257E-06	0.00198068	0.22188125	0.25680719	0.2933139	0.34675238	0.39496542	0.46115805	0.52784301	0.61076672	0.66943378	0.72219587	0.74396295
2.0257E-06	0.00224997	0.22223625	0.25609389	0.29230288	0.34634722	0.39378343	0.46134034	0.52587691	0.60999337	0.67309306	0.72304848	0.74474928
2.0257E-06	0.00251023	0.22251059	0.25679414	0.29430769	0.34721555	0.39599599	0.46421389	0.53099047	0.61018579	0.67296742	0.72560887	0.74704824
2.0257E-06	0.00276206	0.22243387	0.25636799	0.29389063	0.34703705	0.39784217	0.46480546	0.52913896	0.61394588	0.67528394	0.72652701	0.74823794

Table S15b measured data for the UV-Vis titration measurement 2 of **(±)-Zr(1)$_2$** with **G1**

[H] [M]	[G] [M]	399 nm	398 nm	397 nm	396 nm	395 nm	394 nm	393 nm	392 nm	391 nm	390 nm
2.0257E-06	0	0.74875098	0.76716226	0.75856906	0.72162777	0.68404364	0.62453156	0.55533468	0.49483579	0.45239448	0.40541616
2.0257E-06	1.9374E-07	0.74666013	0.76316245	0.75237687	0.71408053	0.67627873	0.61879821	0.54811646	0.49367829	0.44874056	0.40795575
2.0257E-06	3.87E-07	0.74322188	0.75872576	0.74748003	0.70740449	0.66843414	0.61370468	0.54963112	0.4901461	0.44506425	0.40300888
2.0257E-06	5.7978E-07	0.74182766	0.75479888	0.74373966	0.70401572	0.66973197	0.61246412	0.54198491	0.48913836	0.44592523	0.40130137
2.0257E-06	7.7208E-07	0.73866925	0.75067845	0.73715231	0.69814065	0.6595929	0.60253355	0.53667304	0.48525885	0.44180778	0.39862809
2.0257E-06	9.6391E-07	0.73693751	0.74717538	0.73291438	0.69110303	0.65662001	0.59979301	0.53525667	0.48187201	0.43580948	0.3985187
2.0257E-06	1.1553E-06	0.73596587	0.74537172	0.73102238	0.69259372	0.65291151	0.5975556	0.53392716	0.48097452	0.43649041	0.39651351
2.0257E-06	1.5361E-06	0.73174895	0.73904807	0.72424526	0.68165011	0.64697719	0.58954633	0.52591706	0.47104359	0.43442936	0.39232693
2.0257E-06	1.9151E-06	0.7297493	0.73429648	0.71914951	0.67657975	0.63995103	0.58598421	0.52245543	0.46995965	0.4315762	0.38840804
2.0257E-06	2.2922E-06	0.72761224	0.7318009	0.71355115	0.67086153	0.6342399	0.58069561	0.51536439	0.4662277	0.42778905	0.38847066
2.0257E-06	2.6675E-06	0.72607161	0.72901548	0.70895292	0.66289081	0.62922348	0.57987316	0.51877655	0.46696628	0.42729042	0.38711198
2.0257E-06	3.041E-06	0.72521492	0.72729447	0.7067947	0.66464218	0.62699809	0.57491579	0.5128701	0.46524569	0.42469969	0.38509389
2.0257E-06	3.4127E-06	0.72382025	0.72512017	0.70406018	0.66062574	0.62411211	0.57085172	0.50991498	0.46230833	0.42152299	0.38352258
2.0257E-06	3.7827E-06	0.72267251	0.72357802	0.70279907	0.65934413	0.61949902	0.57315426	0.51180362	0.46087437	0.42139863	0.3835028
2.0257E-06	4.1509E-06	0.72395561	0.72211841	0.70244405	0.65800235	0.61974136	0.56985823	0.50991616	0.46271215	0.42261375	0.38209209
2.0257E-06	5.9875E-06	0.72101642	0.71788611	0.69617654	0.6523902	0.61688639	0.56436522	0.50142028	0.45719065	0.4181899	0.38076784
2.0257E-06	7.8198E-06	0.72046004	0.71752665	0.69495735	0.65110598	0.61419288	0.56273345	0.50422887	0.45328841	0.41851494	0.3801806
2.0257E-06	9.6478E-06	0.72023393	0.71716715	0.69320006	0.64671237	0.60871006	0.56014807	0.49998243	0.45473213	0.4165769	0.37971861
2.0257E-06	1.1472E-05	0.72107098	0.71500448	0.69052622	0.64826307	0.60911504	0.55947188	0.49999664	0.4558867	0.41414577	0.38021264
2.0257E-06	1.3291E-05	0.72219431	0.71716606	0.69356	0.64596712	0.60898434	0.5552631	0.49959337	0.45262944	0.41484084	0.37749689
2.0257E-06	1.5106E-05	0.72291028	0.71748596	0.69250738	0.64896458	0.61272197	0.55904334	0.50285601	0.45354223	0.41744553	0.37910964
2.0257E-06	1.6917E-05	0.72450216	0.71886302	0.69563187	0.65261884	0.61142529	0.55598874	0.5031891	0.45667018	0.41740395	0.3779598
2.0257E-06	1.8724E-05	0.72451407	0.71736926	0.6950962	0.6536485	0.61036927	0.55899156	0.50081748	0.45366282	0.41701499	0.37866447
2.0257E-06	2.0526E-05	0.72650506	0.71975948	0.69747759	0.6497248	0.612781	0.56057711	0.50002308	0.45741536	0.41827311	0.38048573
2.0257E-06	3.851E-05	0.72756591	0.72091689	0.69703826	0.65040156	0.61488875	0.55821338	0.49848052	0.45288601	0.41688367	0.38102677
2.0257E-06	5.6452E-05	0.72820885	0.71949381	0.69386077	0.64738596	0.60844482	0.55892164	0.49830759	0.45192373	0.4147406	0.38011334
2.0257E-06	9.2172E-05	0.72889648	0.71809164	0.69201402	0.64283095	0.60488001	0.55385963	0.49587796	0.45161746	0.40902553	0.37704984
2.0257E-06	0.00012773	0.72855429	0.7169752	0.68963049	0.63917886	0.59999399	0.54820018	0.48787175	0.44632964	0.40992032	0.37572883
2.0257E-06	0.00021561	0.72858877	0.71307719	0.68052614	0.62837053	0.59154553	0.54105455	0.48292518	0.43896861	0.40383357	0.36838073
2.0257E-06	0.00030251	0.72910559	0.71144897	0.67987186	0.62976956	0.58530939	0.53602415	0.48060983	0.43519938	0.40163335	0.36720854
2.0257E-06	0.00038845	0.73133974	0.71328448	0.67754118	0.62392138	0.58572529	0.5329368	0.47856919	0.43050717	0.39793698	0.36479647
2.0257E-06	0.00047345	0.73298291	0.71073846	0.67626802	0.62529627	0.58285669	0.53377626	0.47774903	0.43207667	0.39845074	0.3638571
2.0257E-06	0.0007992	0.72994466	0.70889236	0.67255904	0.61725678	0.5772735	0.52786578	0.47168096	0.42895936	0.39327	0.35933988
2.0257E-06	0.00111183	0.73644026	0.71367183	0.67567547	0.61890199	0.57729896	0.530144	0.4731982	0.43045192	0.39621486	0.36076587
2.0257E-06	0.00141237	0.74234262	0.71989837	0.6827828	0.62507495	0.586629	0.534188	0.47913334	0.43198574	0.40092552	0.36503038
2.0257E-06	0.00170172	0.74669329	0.72103141	0.68366007	0.62830673	0.58937297	0.53857211	0.47753004	0.436366	0.39958692	0.36508424
2.0257E-06	0.00198068	0.74682588	0.72286291	0.68708933	0.63048232	0.58709169	0.5354848	0.48202164	0.43542869	0.40206251	0.36726353
2.0257E-06	0.00224997	0.74735156	0.72404829	0.68569831	0.63335345	0.59081963	0.53531084	0.47910205	0.4357771	0.40205688	0.3683424
2.0257E-06	0.00251023	0.75115661	0.72750859	0.69036721	0.63357978	0.59057079	0.53920661	0.48288791	0.43652876	0.40344422	0.36601864

2.0257E-06	0.00276206	0.75257996	0.72930894	0.6873608	0.63443104	0.58979664	0.5360463	0.48262218	0.43663368	0.4031072	0.36974749

Table S16a measured data for the UV-Vis titration measurement 3 of **(±)-Zr(1)$_2$** with **G1**

[H] [M]	[G] [M]	410 nm	409 nm	408 nm	407 nm	406 nm	405 nm	404 nm	403 nm	402 nm	401 nm	400 nm
2.0257E-06	0	0.12824523	0.1511887	0.1769453	0.2152638	0.25386338	0.30944882	0.36337794	0.44550733	0.51853095	0.59926121	0.65080975
2.0257E-06	1.9374E-07	0.13135951	0.15417716	0.17975129	0.21792653	0.25736998	0.31113396	0.3704174	0.45123	0.52216126	0.60231264	0.65102203
2.0257E-06	3.87E-07	0.1322873	0.15547211	0.18225203	0.22142051	0.25941011	0.31466847	0.3711189	0.45550566	0.52511271	0.60352572	0.65193709
2.0257E-06	5.7978E-07	0.13535314	0.15892678	0.18464816	0.2244238	0.26154562	0.31947874	0.37864735	0.46339612	0.52931689	0.60305601	0.6506226
2.0257E-06	7.7208E-07	0.13769276	0.16175383	0.18947736	0.22813285	0.2684608	0.32169937	0.38180172	0.46020748	0.52749708	0.60443028	0.65124079
2.0257E-06	9.6391E-07	0.13855719	0.16357831	0.19090494	0.23131371	0.26909743	0.32283834	0.38147549	0.46373705	0.53182388	0.60639543	0.64898206
2.0257E-06	1.1553E-06	0.14071712	0.16604756	0.19212322	0.23253607	0.2713375	0.32850369	0.38562368	0.46514149	0.53255357	0.60747846	0.64757653
2.0257E-06	1.5361E-06	0.14322348	0.16748518	0.19409277	0.23546915	0.27732511	0.33133714	0.38913788	0.47156228	0.53294353	0.60628758	0.64621393
2.0257E-06	1.9151E-06	0.14497627	0.16953531	0.19883607	0.2410741	0.27733381	0.33212937	0.39228798	0.47088881	0.53695886	0.60836927	0.64764552
2.0257E-06	2.2922E-06	0.14714636	0.17324959	0.20186309	0.24241035	0.28157596	0.33812089	0.39625443	0.47608356	0.53992049	0.60938911	0.64749716
2.0257E-06	2.6675E-06	0.14761202	0.17353721	0.20282676	0.24435191	0.28261297	0.33985351	0.39647959	0.47769632	0.54164072	0.60866584	0.64742156
2.0257E-06	3.041E-06	0.14826713	0.17400313	0.20409318	0.24470777	0.28426777	0.34237348	0.39823567	0.47819438	0.54274951	0.60766005	0.64532702
2.0257E-06	3.4127E-06	0.15009156	0.17552041	0.20365974	0.24571465	0.28888901	0.34379281	0.40388422	0.48015531	0.54430987	0.60941877	0.64790918
2.0257E-06	3.7827E-06	0.15106476	0.17682782	0.20613015	0.24928393	0.28784027	0.3448877	0.40222444	0.4830121	0.5452502	0.60990997	0.64668067
2.0257E-06	4.1509E-06	0.15242704	0.17805722	0.20735505	0.24905558	0.28992493	0.34689147	0.4042473	0.48592095	0.54753475	0.61116044	0.64632027
2.0257E-06	5.9875E-06	0.15460682	0.18155866	0.21087876	0.25428839	0.29312058	0.35131567	0.41170778	0.48750709	0.54700439	0.61204885	0.647453
2.0257E-06	7.8198E-06	0.15620439	0.18260566	0.212214	0.25494269	0.29421795	0.35135282	0.41215999	0.48797808	0.54913656	0.6124613	0.64693383
2.0257E-06	9.6478E-06	0.15764561	0.1840613	0.21446917	0.25559535	0.29631933	0.35391107	0.40967598	0.48975065	0.55404147	0.61407569	0.64848014
2.0257E-06	1.1472E-05	0.15692281	0.1836754	0.2137778	0.25608317	0.29787309	0.35458244	0.41208769	0.49171258	0.55303956	0.6158284	0.64898301
2.0257E-06	1.3291E-05	0.15775205	0.18453112	0.2154442	0.2584633	0.29767663	0.35485775	0.41106839	0.4906816	0.55353273	0.61720628	0.64879841
2.0257E-06	1.5106E-05	0.15848402	0.1847525	0.21443009	0.25847534	0.30186117	0.35672537	0.41680706	0.49340952	0.55509815	0.61954389	0.65255336
2.0257E-06	1.6917E-05	0.15978131	0.18642696	0.21715789	0.26014401	0.29998963	0.35675227	0.41605993	0.49240117	0.55560111	0.61749857	0.65221964
2.0257E-06	1.8724E-05	0.15923286	0.18536365	0.21431972	0.25980201	0.30018693	0.35667002	0.41631794	0.49285689	0.55462554	0.62045881	0.65144405
2.0257E-06	2.0526E-05	0.15966085	0.18659244	0.21698351	0.26165727	0.29990449	0.35823604	0.41778332	0.4952153	0.55804902	0.61873519	0.65272134
2.0257E-06	3.851E-05	0.15946311	0.18794099	0.21940701	0.26172904	0.30322415	0.36046875	0.42384112	0.49655375	0.55812768	0.62065283	0.65357748
2.0257E-06	7.4312E-05	0.16114976	0.18889539	0.22133908	0.26446662	0.30467916	0.367239	0.42187819	0.50108558	0.56376815	0.62382412	0.65370721
2.0257E-06	0.00010995	0.16478945	0.19360711	0.22405212	0.26967094	0.31288625	0.37019549	0.42872924	0.50845338	0.56808645	0.62811191	0.65635503
2.0257E-06	0.00014542	0.16517482	0.1950987	0.22821099	0.27304544	0.31627323	0.37784254	0.43609594	0.51337086	0.5729075	0.62995463	0.65836709
2.0257E-06	0.00023311	0.1700796	0.19973446	0.23155213	0.27795884	0.32285282	0.38585382	0.44408288	0.52284088	0.58024111	0.63621881	0.66124567
2.0257E-06	0.00031982	0.17083511	0.20187137	0.23435033	0.28229483	0.32862481	0.38915013	0.45120101	0.52783169	0.58419301	0.64046069	0.66339894
2.0257E-06	0.00040557	0.17111583	0.20337582	0.23660656	0.28402874	0.32752639	0.39272452	0.45256311	0.53037775	0.59056741	0.64115096	0.66434211
2.0257E-06	0.00049038	0.17194135	0.2030174	0.23955338	0.28785054	0.33100448	0.39426981	0.45680837	0.53421159	0.59305931	0.64336397	0.66494186
2.0257E-06	0.00081544	0.17562084	0.2080733	0.24152859	0.29072665	0.33609916	0.39869447	0.46052189	0.54074173	0.59634148	0.64680772	0.66772567
2.0257E-06	0.00112745	0.17381086	0.20732164	0.24283028	0.29211242	0.33728127	0.39945755	0.46297056	0.53977303	0.59511761	0.64646778	0.66684762
2.0257E-06	0.00142741	0.17565797	0.20793736	0.24274251	0.29217644	0.33870806	0.40064298	0.4615414	0.54218151	0.59765615	0.64822562	0.66894873
2.0257E-06	0.00171622	0.17709664	0.20962388	0.24360385	0.29331177	0.34120946	0.4051853	0.46580794	0.54451003	0.60095545	0.65066507	0.67034437
2.0257E-06	0.00199468	0.17549676	0.20817107	0.24242377	0.29107724	0.33581193	0.40353172	0.46512561	0.5436749	0.59878273	0.65009607	0.66957124
2.0257E-06	0.0022635	0.17466815	0.20702729	0.24414416	0.29295023	0.33769535	0.40221246	0.46297734	0.5425397	0.59818982	0.64972125	0.66982304
2.0257E-06	0.00252333	0.17724866	0.20848951	0.2424706	0.29342493	0.33968057	0.40339526	0.46841257	0.54474967	0.60263919	0.65193986	0.67179524
2.0257E-06	0.00277476	0.17719757	0.21095293	0.24491387	0.293998	0.34097167	0.40424135	0.46589928	0.54516091	0.60044613	0.65255877	0.67250934

Table S16b measured data for the UV-Vis titration measurement 3 of **(±)-Zr(1)$_2$** with **G1**

[H] [M]	[G] [M]	399 nm	398 nm	397 nm	396 nm	395 nm	394 nm	393 nm	392 nm	391 nm	390 nm
2.0257E-06	0	0.69659398	0.71479825	0.70560627	0.6697409	0.63203482	0.57647733	0.51087378	0.45206494	0.4060405	0.36148219
2.0257E-06	1.9374E-07	0.69358869	0.71090813	0.70121289	0.6608621	0.62422199	0.56973315	0.50403894	0.44752337	0.40385811	0.35920022
2.0257E-06	3.87E-07	0.693369	0.70821198	0.69694581	0.65983071	0.62259885	0.56477847	0.49974539	0.44508746	0.40248152	0.36024695
2.0257E-06	5.7978E-07	0.68972712	0.7022584	0.69021385	0.65250444	0.61392813	0.56007009	0.49550679	0.44028532	0.39863085	0.35570978
2.0257E-06	7.7208E-07	0.68784269	0.6976188	0.68650439	0.64647128	0.60931357	0.55488171	0.49204128	0.43786418	0.39570784	0.35454457
2.0257E-06	9.6391E-07	0.68530328	0.69493664	0.68146581	0.64176978	0.602493	0.54967434	0.48839537	0.43562443	0.39092571	0.35189188
2.0257E-06	1.1553E-06	0.68312653	0.69196208	0.67707737	0.6396202	0.59838815	0.54625066	0.48351662	0.43301578	0.39158501	0.34987911
2.0257E-06	1.5361E-06	0.68155532	0.68778639	0.6716467	0.63106899	0.59587924	0.53873353	0.47792841	0.42849042	0.38980539	0.34972058
2.0257E-06	1.9151E-06	0.6805567	0.68434314	0.66873	0.62772993	0.58834515	0.53636281	0.47685767	0.42571248	0.38384781	0.34528206
2.0257E-06	2.2922E-06	0.67739282	0.68003259	0.6628118	0.62334047	0.58532666	0.53049187	0.47311069	0.42358695	0.38248767	0.3447115
2.0257E-06	2.6675E-06	0.67638363	0.67805006	0.65977927	0.6180404	0.57803847	0.52930214	0.46831791	0.42152275	0.38159587	0.3427393
2.0257E-06	3.041E-06	0.6753801	0.67593585	0.65666651	0.61371844	0.57792835	0.52611833	0.46815862	0.41866045	0.37948679	0.34125557
2.0257E-06	3.4127E-06	0.6743802	0.6755897	0.65464623	0.61269911	0.57509829	0.52421858	0.46733917	0.41835801	0.37997075	0.34039708
2.0257E-06	3.7827E-06	0.67473485	0.67362048	0.65446504	0.6104854	0.57309385	0.52154174	0.46314823	0.41623228	0.37782171	0.33879202
2.0257E-06	4.1509E-06	0.67384652	0.67277406	0.65323339	0.61081342	0.57444362	0.52228735	0.46044211	0.41333218	0.37582265	0.33827082
2.0257E-06	5.9875E-06	0.67173098	0.66843848	0.64590489	0.60396384	0.56417458	0.51409392	0.46044629	0.41312446	0.37435665	0.3369471
2.0257E-06	7.8198E-06	0.67112563	0.66804055	0.64590303	0.60245303	0.56514392	0.51408873	0.45817149	0.40955857	0.37244693	0.33763612
2.0257E-06	9.6478E-06	0.67015609	0.66695502	0.6447021	0.59932342	0.56255385	0.51171359	0.45652103	0.40928095	0.37177753	0.33505287
2.0257E-06	1.1472E-05	0.67158301	0.66668756	0.64280308	0.59791578	0.56330467	0.51432771	0.45374934	0.40715502	0.37152008	0.33520151
2.0257E-06	1.3291E-05	0.67180438	0.66577447	0.64504422	0.5985185	0.5629663	0.51160396	0.45380772	0.40788908	0.37226377	0.33525811
2.0257E-06	1.5106E-05	0.67285249	0.66692213	0.64348785	0.59839926	0.56030963	0.51237283	0.45394833	0.40945638	0.37079064	0.33583502
2.0257E-06	1.6917E-05	0.67197697	0.66728436	0.64262431	0.5968117	0.56162857	0.51076441	0.45449548	0.40718715	0.37034367	0.33643504
2.0257E-06	1.8724E-05	0.67284671	0.66698364	0.64308682	0.59911993	0.56148949	0.51278451	0.45495042	0.40886805	0.37218139	0.33681628
2.0257E-06	2.0526E-05	0.67355978	0.66888696	0.64472628	0.59939331	0.56304747	0.50927788	0.45551366	0.41172802	0.37183091	0.33659571
2.0257E-06	3.851E-05	0.6747264	0.66843418	0.64398703	0.59977946	0.55982975	0.51020292	0.45431942	0.40752438	0.37000329	0.33465872
2.0257E-06	7.4312E-05	0.67173112	0.66324175	0.6372822	0.59199977	0.55209542	0.50084901	0.44460833	0.40356571	0.36729059	0.33126936
2.0257E-06	0.00010995	0.67298359	0.66117901	0.63499976	0.58912516	0.54918469	0.49941451	0.44489611	0.39985082	0.36435268	0.3302807
2.0257E-06	0.00014542	0.6734324	0.66059386	0.63164764	0.58432346	0.54613613	0.49527444	0.44139012	0.39536207	0.35874473	0.32621108
2.0257E-06	0.00023311	0.67207393	0.6569347	0.62546107	0.57651773	0.53762176	0.48687288	0.43317383	0.38911071	0.3566834	0.3213703
2.0257E-06	0.00031982	0.6713809	0.65366783	0.62230923	0.56993844	0.5338005	0.48309892	0.42782952	0.38511845	0.35162299	0.32005983
2.0257E-06	0.00040557	0.67192531	0.65289265	0.61819595	0.56811375	0.53004963	0.48235172	0.42410428	0.38234261	0.34821579	0.31443778
2.0257E-06	0.00049038	0.67213406	0.65141196	0.6175728	0.56794716	0.52910544	0.47882096	0.42383791	0.38029133	0.34622077	0.31524001
2.0257E-06	0.00081544	0.67227535	0.65015553	0.61570119	0.5638672	0.52334909	0.47441228	0.42149763	0.38064407	0.34558116	0.31306841
2.0257E-06	0.00112745	0.67183773	0.64932803	0.61316494	0.56419364	0.52504871	0.47386915	0.42033703	0.37733183	0.34294278	0.31291673
2.0257E-06	0.00142741	0.67320086	0.64978249	0.61437054	0.56316532	0.52318396	0.47483139	0.42151563	0.37698261	0.34425856	0.31162141
2.0257E-06	0.00171622	0.67421838	0.65088012	0.61867311	0.56370862	0.52472868	0.47379755	0.42030775	0.37822222	0.34444567	0.31314563
2.0257E-06	0.00199468	0.67353286	0.64992787	0.61442156	0.5615405	0.52370419	0.47308262	0.42100106	0.37746067	0.34425788	0.31267722
2.0257E-06	0.0022635	0.67380946	0.65178196	0.61556577	0.55956446	0.5224291	0.47244321	0.42006146	0.3768308	0.34338521	0.31129243
2.0257E-06	0.00252333	0.67565565	0.65158193	0.61914449	0.56480723	0.52427863	0.47198369	0.42075147	0.37797528	0.34394281	0.3116674
2.0257E-06	0.00277476	0.67580786	0.65326896	0.61939969	0.56476297	0.52544691	0.47429543	0.42079171	0.37880721	0.34463289	0.31230908

Table S17a fitted data for the UV-Vis titration measurement 1 of **(±)-Zr(1)$_2$** with **G1**

[H] [M]	[G] [M]	410 nm	409 nm	408 nm	407 nm	406 nm	405 nm	404 nm	403 nm	402 nm	401 nm	400 nm
2.0257E-06	0	0.12884035	0.15154906	0.176858	0.21639962	0.25307497	0.30498596	0.3668116	0.45313191	0.52193569	0.60254216	0.65370166
2.0257E-06	1.9374E-07	0.13069341	0.1537111	0.17933793	0.21911776	0.25601941	0.30841836	0.3698954	0.45555084	0.52388408	0.60314639	0.65290725
2.0257E-06	3.87E-07	0.13247468	0.15578949	0.18172197	0.22173098	0.25885033	0.3117183	0.37286063	0.45787723	0.52575806	0.60372839	0.65214487

2.0257E-06	5.7978E-07	0.1341763	0.15777505	0.18399962	0.2242278	0.26155527	0.31487126	0.37569431	0.46010096	0.52754951	0.60428573	0.65141804
2.0257E-06	7.7208E-07	0.13579043	0.15965866	0.18616042	0.22659678	0.26412186	0.31786279	0.37838351	0.46221194	0.52925032	0.604816	0.65073025
2.0257E-06	9.6391E-07	0.13730976	0.16143182	0.18819461	0.22882723	0.26653855	0.32067943	0.38091617	0.46420075	0.53085292	0.60531693	0.65008478
2.0257E-06	1.1553E-06	0.1387281	0.16308729	0.19009392	0.2309101	0.26879553	0.32330973	0.38328204	0.46605942	0.53235088	0.60578661	0.64948439
2.0257E-06	1.5361E-06	0.1412432	0.16602348	0.193463	0.23460589	0.27280091	0.32797694	0.38748266	0.46936225	0.53501357	0.60662639	0.64842708
2.0257E-06	1.9151E-06	0.14333161	0.16846247	0.19626219	0.23767815	0.27613154	0.33185687	0.39097862	0.47211519	0.53723418	0.60733412	0.64756023
2.0257E-06	2.2922E-06	0.1450231	0.17043893	0.19853124	0.24017037	0.27883447	0.33500443	0.39381906	0.47435655	0.53904351	0.60791896	0.64687047
2.0257E-06	2.6675E-06	0.14637406	0.17201856	0.20034544	0.24216492	0.28099882	0.33752363	0.39609702	0.47615892	0.54049989	0.60839828	0.64633256
2.0257E-06	3.041E-06	0.14744989	0.17327757	0.20179214	0.24375734	0.28272798	0.33953508	0.39792044	0.47760648	0.541671	0.60879222	0.64591721
2.0257E-06	3.4127E-06	0.14831148	0.1742869	0.20295264	0.24503658	0.2841182	0.3411511	0.39938982	0.47877764	0.54261987	0.60911956	0.64559718
2.0257E-06	3.7827E-06	0.14900911	0.17510514	0.20389411	0.24607613	0.28524899	0.34246446	0.40058817	0.47973715	0.54339855	0.60939581	0.64534994
2.0257E-06	4.1509E-06	0.14958175	0.17577771	0.20466857	0.24693288	0.28618194	0.34354703	0.4015798	0.48053519	0.54404736	0.60963299	0.64515808
2.0257E-06	5.9875E-06	0.15138248	0.17790216	0.20712131	0.24966301	0.28916507	0.34699816	0.40478092	0.48315295	0.54618771	0.6104869	0.64466965
2.0257E-06	7.8198E-06	0.15235583	0.17906198	0.20846807	0.25118219	0.29083719	0.34892023	0.4066113	0.48469872	0.54746555	0.61107905	0.64454445
2.0257E-06	9.6478E-06	0.15300024	0.17983795	0.2093745	0.25221865	0.29198636	0.35023274	0.40789379	0.4858145	0.54839697	0.61156334	0.64455964
2.0257E-06	1.1472E-05	0.15348355	0.18042567	0.21006481	0.25301769	0.29287801	0.35124539	0.40890562	0.48671662	0.5491559	0.61199175	0.64464031
2.0257E-06	1.3291E-05	0.15387584	0.18090681	0.21063259	0.25368175	0.29362303	0.35208754	0.4097626	0.48749551	0.54981504	0.61238607	0.64475532
2.0257E-06	1.5106E-05	0.15421117	0.18132107	0.21112338	0.25426067	0.29427535	0.3528221	0.41052112	0.48819524	0.55040985	0.61275701	0.64488963
2.0257E-06	1.6917E-05	0.15450804	0.18169002	0.2115619	0.25478152	0.29486428	0.35348327	0.41121182	0.48883979	0.55095962	0.61311044	0.64503516
2.0257E-06	1.8724E-05	0.15477734	0.18202636	0.21196272	0.25526027	0.29540713	0.3540912	0.41185282	0.48944337	0.55147579	0.61344991	0.64518722
2.0257E-06	2.0526E-05	0.1550259	0.18233808	0.212335	0.25570697	0.29591479	0.35465861	0.41245555	0.49001495	0.5519656	0.6137777	0.64534294
2.0257E-06	3.851E-05	0.15695733	0.18478861	0.21527958	0.25928535	0.30000687	0.35920742	0.41738627	0.49477956	0.55607047	0.61664746	0.64689599
2.0257E-06	7.4312E-05	0.1596209	0.18820467	0.21940716	0.26435857	0.30584009	0.36566089	0.42450475	0.50176678	0.56211644	0.62102091	0.6494798
2.0257E-06	0.00010995	0.16153502	0.19066798	0.22238878	0.26803627	0.31007586	0.37034017	0.42969387	0.50688417	0.56655015	0.62425983	0.65143868
2.0257E-06	0.00014542	0.16300018	0.19255551	0.22467467	0.27085887	0.31332844	0.3739317	0.43368318	0.51082389	0.56996484	0.62676164	0.65296211
2.0257E-06	0.00023311	0.16550797	0.19578835	0.22859113	0.2756981	0.31890662	0.38008949	0.44052982	0.5175913	0.5758318	0.63106787	0.65559524
2.0257E-06	0.00031982	0.1671037	0.19784627	0.23108473	0.27878051	0.3224604	0.38401186	0.44489368	0.52190699	0.5795738	0.63381747	0.65728084
2.0257E-06	0.00040557	0.16820909	0.19927209	0.23281256	0.28091671	0.32492349	0.38673021	0.44791881	0.52489941	0.5821686	0.63572502	0.65845151
2.0257E-06	0.00049038	0.1690202	0.20031842	0.23408057	0.28248457	0.32673134	0.38872534	0.45013942	0.5270963	0.58407363	0.63712587	0.65931172
2.0257E-06	0.00081544	0.17083643	0.20266159	0.23692036	0.28599624	0.33078077	0.39319406	0.45511396	0.53201836	0.58834197	0.64026541	0.66124086
2.0257E-06	0.00112745	0.17172398	0.20380675	0.23830828	0.28771269	0.33276014	0.39537831	0.45754576	0.53442478	0.59042885	0.64180076	0.66218478
2.0257E-06	0.00142741	0.17225043	0.20448601	0.23913155	0.28873089	0.33393433	0.39667401	0.45898839	0.53585242	0.59166693	0.64271173	0.66274497
2.0257E-06	0.00171622	0.1725991	0.20493591	0.23967684	0.28940529	0.33471206	0.39753221	0.45994395	0.53679808	0.59248704	0.64331519	0.66311611
2.0257E-06	0.00199468	0.17284718	0.20525602	0.24006483	0.28988515	0.33526544	0.39814285	0.46062387	0.53747097	0.59307059	0.6437446	0.66338022
2.0257E-06	0.0022635	0.17303282	0.20549556	0.24035516	0.29024424	0.33567955	0.39859981	0.46113268	0.53797452	0.59350729	0.64406595	0.66357789
2.0257E-06	0.00252333	0.17317703	0.20568164	0.2405807	0.29052319	0.33600125	0.39895479	0.46152795	0.53836571	0.59384654	0.64431561	0.66373146
2.0257E-06	0.00277476	0.17329235	0.20583045	0.24076106	0.29074626	0.3362585	0.39923866	0.46184403	0.53867853	0.59411784	0.64451525	0.66385427

Table S17b fitted data for the UV-Vis titration measurement 1 of **(±)-Zr(1)$_2$** with **G1**

[H] [M]	[G] [M]	399 nm	398 nm	397 nm	396 nm	395 nm	394 nm	393 nm	392 nm	391 nm	390 nm
2.0257E-06	0	0.69949751	0.7174257	0.70830023	0.67250925	0.63220435	0.57710724	0.51091521	0.45208751	0.40748187	0.36412063
2.0257E-06	1.9374E-07	0.6969423	0.71318304	0.70301939	0.66659397	0.62653672	0.57173381	0.50626929	0.44845905	0.40443884	0.36161178
2.0257E-06	3.87E-07	0.69448727	0.70910586	0.69794414	0.66090883	0.62108942	0.56656921	0.50180386	0.44497136	0.40151379	0.35920018
2.0257E-06	5.7978E-07	0.6921434	0.7052123	0.69309703	0.65547903	0.61588661	0.56163633	0.49753872	0.44163985	0.39871963	0.35689648
2.0257E-06	7.7208E-07	0.68992162	0.70152042	0.68850047	0.65032968	0.61095231	0.55695794	0.49349355	0.43847988	0.39606925	0.35471129
2.0257E-06	9.6391E-07	0.68783214	0.69804707	0.6841754	0.64548422	0.60630896	0.55255532	0.48968673	0.43550579	0.39357464	0.35265452
2.0257E-06	1.1553E-06	0.68588361	0.69480654	0.68013959	0.64096252	0.6019756	0.5484465	0.48613385	0.43272972	0.39124599	0.35073455
2.0257E-06	1.5361E-06	0.68243532	0.68906677	0.67298893	0.63295	0.59429587	0.54116433	0.47983665	0.42780815	0.38711718	0.34733025
2.0257E-06	1.9151E-06	0.67958251	0.68431063	0.66706031	0.6263053	0.58792577	0.53512342	0.47461231	0.42372323	0.38368955	0.34450395
2.0257E-06	2.2922E-06	0.6772836	0.68046944	0.66226842	0.62093297	0.58277387	0.53023713	0.47038594	0.42041657	0.38091418	0.34221532
2.0257E-06	2.6675E-06	0.67545979	0.67741313	0.65845168	0.61665216	0.57866707	0.52634137	0.46701573	0.4177776	0.3786984	0.34038798
2.0257E-06	3.041E-06	0.67401973	0.67499086	0.65542272	0.61325316	0.57540457	0.52324587	0.46433721	0.41567807	0.37693473	0.33893332
2.0257E-06	3.4127E-06	0.67287839	0.6730622	0.65300711	0.61054073	0.57279946	0.52077344	0.46219725	0.41399859	0.37552313	0.33776888
2.0257E-06	3.7827E-06	0.67196549	0.67151116	0.65106076	0.6083536	0.57069734	0.51877777	0.46046938	0.41264052	0.37438092	0.33682652
2.0257E-06	4.1509E-06	0.67122665	0.67024789	0.64947204	0.60656683	0.5689786	0.51714548	0.45905561	0.41152747	0.37344409	0.33605346
2.0257E-06	5.9875E-06	0.66901206	0.66637784	0.64456871	0.60103642	0.56364387	0.51207307	0.45465688	0.40804503	0.37050571	0.33362726
2.0257E-06	7.8198E-06	0.66794643	0.66440971	0.6420301	0.59815367	0.56084489	0.50940434	0.45233601	0.40618397	0.36892657	0.33232159
2.0257E-06	9.6478E-06	0.6673338	0.66319415	0.64042842	0.5963205	0.55905163	0.50768915	0.4508396	0.40496693	0.36788759	0.33146126
2.0257E-06	1.1472E-05	0.66693991	0.66234424	0.63928294	0.59499883	0.55774889	0.50643921	0.44974563	0.40406478	0.36711291	0.33081889
2.0257E-06	1.3291E-05	0.6666671	0.66169856	0.63839309	0.59396411	0.5567217	0.50545075	0.44887794	0.40334014	0.36648739	0.33029956
2.0257E-06	1.5106E-05	0.66646799	0.66117871	0.63766143	0.59310727	0.55586558	0.50462476	0.44815096	0.40272623	0.36595507	0.32985712
2.0257E-06	1.6917E-05	0.66631692	0.66074223	0.63703513	0.59236917	0.55512389	0.50390752	0.44751825	0.40218683	0.36548556	0.32946654
2.0257E-06	1.8724E-05	0.66619887	0.66036417	0.63648315	0.59171502	0.55446331	0.50326745	0.44695251	0.4017006	0.36506098	0.32911307
2.0257E-06	2.0526E-05	0.66610444	0.66002889	0.63598603	0.59112302	0.55386294	0.50268474	0.4464366	0.40125417	0.3646701	0.32878745
2.0257E-06	3.851E-05	0.6656956	0.65772967	0.63239966	0.58678664	0.54940712	0.49833759	0.44256825	0.39783796	0.3616555	0.32627159
2.0257E-06	7.4312E-05	0.6655503	0.65495297	0.6278098	0.58114599	0.54353166	0.49257525	0.43741432	0.39319463	0.35752731	0.32282041
2.0257E-06	0.00010995	0.66554254	0.65304857	0.62459363	0.57717089	0.53937163	0.48848803	0.43375233	0.38987349	0.35456742	0.32034456
2.0257E-06	0.00014542	0.66555933	0.65161225	0.62215116	0.5741466	0.53620198	0.48537211	0.43095909	0.38733502	0.35230336	0.31845042
2.0257E-06	0.00023311	0.66561231	0.64917666	0.6179912	0.56898983	0.53079234	0.48005232	0.4261886	0.38299405	0.34842984	0.31520943
2.0257E-06	0.00031982	0.6656556	0.6476359	0.61535234	0.56571632	0.52735633	0.47667263	0.42315724	0.38023342	0.34596576	0.31314759
2.0257E-06	0.00040557	0.66568845	0.64657126	0.61352676	0.56345099	0.52497795	0.47433302	0.42105858	0.37832153	0.34425903	0.31171943
2.0257E-06	0.00049038	0.6657137	0.64579114	0.61218819	0.5617897	0.52323352	0.47261693	0.41951915	0.37691883	0.34300677	0.31067155
2.0257E-06	0.00081544	0.66577302	0.64404693	0.60919322	0.55807198	0.51932916	0.4687758	0.41607324	0.37377835	0.34020289	0.30832523
2.0257E-06	0.00112745	0.66580314	0.64319564	0.60773061	0.55625614	0.51742192	0.46689935	0.41438979	0.37224385	0.33883278	0.30717869
2.0257E-06	0.00142741	0.6658213	0.64269097	0.60686332	0.55517932	0.51629084	0.46578652	0.41339139	0.37133372	0.33802012	0.30649864
2.0257E-06	0.00171622	0.66583344	0.64235683	0.60628899	0.55446621	0.51554177	0.46504953	0.41273019	0.37073094	0.3374819	0.30604824
2.0257E-06	0.00199468	0.66584212	0.64211913	0.6058804	0.55395887	0.51500884	0.46452518	0.41225975	0.37030207	0.33709895	0.30572777
2.0257E-06	0.0022635	0.66584864	0.64194128	0.60557467	0.55357925	0.51461007	0.46413283	0.41190774	0.36998115	0.33681239	0.30548798
2.0257E-06	0.00252333	0.66585372	0.64180314	0.60533718	0.55328436	0.51430029	0.46382804	0.41163429	0.36973185	0.33658979	0.30530169
2.0257E-06	0.00277476	0.6658578	0.64169268	0.60514728	0.55304856	0.51405259	0.46358433	0.41141563	0.3695325	0.33641178	0.30515273

Table S18a fitted data for the UV-Vis titration measurement 2 of **(±)-Zr(1)$_2$** with **G1**

[H] [M]	[G] [M]	410 nm	409 nm	408 nm	407 nm	406 nm	405 nm	404 nm	403 nm	402 nm	401 nm	400 nm
2.0257E-06	0	0.16637195	0.18953688	0.21697816	0.25619596	0.29301777	0.34968534	0.40935221	0.49589127	0.56762701	0.65025371	0.70157259
2.0257E-06	1.9374E-07	0.16852386	0.19198717	0.21963772	0.25918814	0.29634649	0.35316123	0.4128762	0.4988376	0.56989994	0.65116771	0.7011605
2.0257E-06	3.87E-07	0.17059122	0.1943413	0.22219302	0.26206316	0.29954494	0.35650127	0.41626258	0.50166933	0.5720849	0.65204725	0.70076613
2.0257E-06	5.7978E-07	0.17256503	0.19658901	0.22463296	0.26480853	0.30259924	0.35969099	0.41949671	0.50437427	0.57417252	0.65288865	0.70039137
2.0257E-06	7.7208E-07	0.17443638	0.19872021	0.22694658	0.26741194	0.30549571	0.36271612	0.42256411	0.50694038	0.57615356	0.65368832	0.70003812
2.0257E-06	9.6391E-07	0.17619708	0.20072554	0.22912376	0.269862	0.3082217	0.36556346	0.42545143	0.50935652	0.57801949	0.65444289	0.69970808
2.0257E-06	1.1553E-06	0.17784023	0.20259719	0.23115601	0.2721492	0.31076659	0.36822195	0.42814747	0.51161339	0.57976314	0.65514958	0.69940273
2.0257E-06	1.5361E-06	0.18075362	0.20591632	0.23476068	0.27620681	0.31528183	0.37293977	0.43293264	0.51562169	0.58286244	0.65641096	0.6988702
2.0257E-06	1.9151E-06	0.18317386	0.20867457	0.2377573	0.27958109	0.31903732	0.37686529	0.43691529	0.51896172	0.58544875	0.65747144	0.69844117
2.0257E-06	2.2922E-06	0.18513623	0.21091204	0.24018935	0.28232087	0.32208737	0.38005515	0.44015279	0.52168123	0.58755868	0.65834531	0.69810819
2.0257E-06	2.6675E-06	0.18670557	0.21270249	0.24213677	0.28451601	0.32453187	0.3826135	0.44275061	0.52386802	0.58925965	0.65905893	0.69785756
2.0257E-06	3.041E-06	0.18795666	0.21413094	0.24369175	0.28627008	0.32648598	0.38466043	0.44483039	0.52562335	0.59062934	0.65964268	0.69767348
2.0257E-06	3.4127E-06	0.18895913	0.21527661	0.24494013	0.28767957	0.32805696	0.38630778	0.44650542	0.52704154	0.59174013	0.66012483	0.69754128
2.0257E-06	3.7827E-06	0.18977072	0.21620514	0.24595309	0.28882445	0.32933372	0.38764828	0.4478696	0.52820077	0.59265202	0.66052887	0.69744876

2.0257E-06	4.1509E-06	0.19043631	0.21696759	0.24678597	0.28976693	0.33038543	0.38875402	0.44899596	0.52916183	0.59341168	0.66087303	0.69738646
2.0257E-06	5.9875E-06	0.19251699	0.21936105	0.24941205	0.29275026	0.33372145	0.39227758	0.45259652	0.53227487	0.59591013	0.66208317	0.69733409
2.0257E-06	7.8198E-06	0.19362174	0.22064416	0.25083401	0.29437995	0.33555222	0.39423084	0.4546061	0.53406159	0.59738902	0.66289101	0.69748157
2.0257E-06	9.6478E-06	0.19433762	0.2214846	0.25177563	0.29546936	0.33678203	0.39555676	0.4559798	0.53531714	0.59845861	0.66353525	0.69770517
2.0257E-06	1.1472E-05	0.19486364	0.22210871	0.25248228	0.29629423	0.33771745	0.39657503	0.45704142	0.53631116	0.59932586	0.66409688	0.69796305
2.0257E-06	1.3291E-05	0.19528313	0.22261126	0.25305669	0.29697004	0.33848687	0.39741954	0.45792658	0.53715659	0.60007748	0.66460989	0.69823776
2.0257E-06	1.5106E-05	0.19563663	0.22303838	0.25354889	0.297553	0.33915277	0.39815547	0.45870133	0.53790844	0.60075572	0.66509089	0.69852087
2.0257E-06	1.6917E-05	0.19594615	0.2234151	0.25398601	0.29807362	0.3397491	0.39881823	0.45940151	0.5385966	0.60138357	0.66554892	0.69880787
2.0257E-06	1.8724E-05	0.19622462	0.22375614	0.25438403	0.29854984	0.34029582	0.39942864	0.46004824	0.53923869	0.60197456	0.66598936	0.69909614
2.0257E-06	2.0526E-05	0.19648014	0.22407073	0.25475294	0.29899295	0.34080545	0.39999979	0.46065478	0.53984578	0.60253723	0.66641562	0.69938411
2.0257E-06	3.851E-05	0.19845705	0.22654276	0.25769298	0.302563	0.34493318	0.40467461	0.46565142	0.54495849	0.6073638	0.6702272	0.70215674
2.0257E-06	5.6452E-05	0.19998982	0.22848433	0.2600285	0.30542356	0.34825415	0.40846619	0.46972381	0.54919379	0.61141472	0.67351749	0.7046617
2.0257E-06	9.2172E-05	0.20245979	0.23162916	0.26382825	0.31009305	0.35368366	0.41468409	0.47641446	0.55619408	0.61814215	0.6790362	0.70892798
2.0257E-06	0.00012773	0.20442909	0.2341425	0.26687122	0.31383822	0.35804153	0.41968166	0.48179647	0.56184046	0.62357995	0.68351661	0.71241468
2.0257E-06	0.00021561	0.20800739	0.23871484	0.27241276	0.32066376	0.36598651	0.42879926	0.49161949	0.57215991	0.63352867	0.69173151	0.71882841
2.0257E-06	0.00030251	0.21044106	0.24182654	0.27618611	0.32531328	0.37139962	0.43501358	0.49831607	0.57919991	0.64031952	0.69734524	0.72321874
2.0257E-06	0.00038845	0.21220637	0.24408424	0.27892443	0.32868797	0.37532882	0.43952497	0.50317797	0.58431254	0.64525225	0.7014247	0.72641123
2.0257E-06	0.00047345	0.21354593	0.24579765	0.28100282	0.33124957	0.37831142	0.44294977	0.50686901	0.58819448	0.64899799	0.7045232	0.72883685
2.0257E-06	0.0007992	0.21669109	0.2498211	0.28588386	0.33726589	0.38531683	0.45099438	0.51553939	0.59731458	0.65779912	0.71180521	0.73453943
2.0257E-06	0.00111183	0.21830635	0.25188761	0.28839107	0.34035645	0.38891559	0.45512723	0.51999389	0.60200065	0.6623217	0.71554784	0.73747108
2.0257E-06	0.00141237	0.21929022	0.25314641	0.28991836	0.34223915	0.39110791	0.45764496	0.5227076	0.60485558	0.66507712	0.71782823	0.73925755
2.0257E-06	0.00170172	0.21995278	0.25399414	0.29094693	0.34350709	0.39258437	0.4593406	0.52453525	0.60677838	0.66693294	0.71936418	0.74046089
2.0257E-06	0.00198068	0.22042963	0.25460425	0.2916872	0.34441965	0.393647	0.460561	0.52585067	0.6081623	0.66826866	0.72046971	0.74132704
2.0257E-06	0.00224997	0.22078944	0.25506463	0.2922458	0.34510825	0.39444886	0.46148191	0.52684327	0.60920661	0.66927662	0.72130397	0.74198068
2.0257E-06	0.00251023	0.22107074	0.25542456	0.29268252	0.34564662	0.39507577	0.4622019	0.52761932	0.6100231	0.67006468	0.72195624	0.74249175
2.0257E-06	0.00276206	0.22129682	0.25571383	0.2930335	0.3460793	0.39557961	0.46278055	0.52824303	0.61067931	0.67069805	0.72248048	0.7429025

Table S18b fitted data for the UV-Vis titration measurement 2 of **(±)-Zr(1)$_2$** with **G1**

[H] [M]	[G] [M]	399 nm	398 nm	397 nm	396 nm	395 nm	394 nm	393 nm	392 nm	391 nm	390 nm
2.0257E-06	0	0.74875098	0.76716226	0.75856906	0.72162777	0.68404364	0.62453156	0.55533468	0.49483579	0.45239448	0.40541616
2.0257E-06	1.9374E-07	0.74652934	0.76319808	0.75352022	0.71596984	0.67840254	0.61954606	0.55113855	0.49177566	0.44962593	0.40344542
2.0257E-06	3.87E-07	0.74439657	0.75939126	0.74867128	0.71053565	0.67298438	0.61475747	0.54710812	0.4888362	0.44696665	0.40155237
2.0257E-06	5.7978E-07	0.74236213	0.75575858	0.74404356	0.70534903	0.66781289	0.61018676	0.54326103	0.48603026	0.44442827	0.39974529
2.0257E-06	7.7208E-07	0.74043538	0.75231658	0.73965806	0.70043351	0.66291152	0.60585464	0.53961471	0.48337053	0.44202229	0.39803234
2.0257E-06	9.6391E-07	0.73862495	0.74908057	0.73553423	0.69581083	0.65830193	0.60178025	0.53618526	0.48086873	0.43975931	0.39642106
2.0257E-06	1.1553E-06	0.7369381	0.74606336	0.73168833	0.69149921	0.65400228	0.59797962	0.53298617	0.47853469	0.43764824	0.39491779
2.0257E-06	1.5361E-06	0.73395632	0.740723	0.72487824	0.68386275	0.6463862	0.59124682	0.52731879	0.47439883	0.43390801	0.39225389
2.0257E-06	1.9151E-06	0.73149296	0.73630059	0.71923422	0.67753132	0.64007044	0.58566256	0.52261788	0.47096678	0.4308051	0.39004312
2.0257E-06	2.2922E-06	0.72951088	0.73273041	0.71467281	0.67241153	0.63496194	0.58114466	0.51881429	0.46818821	0.4282939	0.38825305
2.0257E-06	2.6675E-06	0.72794181	0.72989167	0.71104059	0.66833167	0.63088964	0.57754201	0.51578086	0.46597052	0.42629057	0.38682409
2.0257E-06	3.041E-06	0.72670706	0.72764509	0.70816067	0.6650938	0.6276563	0.57468042	0.51337103	0.46420698	0.42469847	0.38568751
2.0257E-06	3.4127E-06	0.72573333	0.72586094	0.70586829	0.66251355	0.62507824	0.57239765	0.51144826	0.46279818	0.42342756	0.38477932
2.0257E-06	3.7827E-06	0.7249599	0.72443174	0.7040269	0.66043812	0.6230032	0.5705592	0.5098994	0.46166171	0.42240322	0.38404647
2.0257E-06	4.1509E-06	0.7243395	0.72327383	0.70253029	0.65874862	0.62131275	0.56906049	0.50863642	0.46073348	0.42156743	0.38344769
2.0257E-06	5.9875E-06	0.72254595	0.71980324	0.69799388	0.6535994	0.61614706	0.56447007	0.50476452	0.45787172	0.41899951	0.38159941
2.0257E-06	7.8198E-06	0.72177324	0.71814406	0.69576016	0.65102821	0.61355054	0.5621493	0.5028026	0.45640142	0.41769128	0.38064704
2.0257E-06	9.6478E-06	0.72140369	0.71720297	0.69444044	0.64948098	0.61197479	0.56073054	0.50159983	0.45548459	0.41688386	0.38005107
2.0257E-06	1.1472E-05	0.72122801	0.71660944	0.69356409	0.64843099	0.610895	0.55975026	0.50076616	0.45483715	0.41632004	0.37962863
2.0257E-06	1.3291E-05	0.72115866	0.71620843	0.69293413	0.64765779	0.6100915	0.55901441	0.50013832	0.4543402	0.41589216	0.37930317
2.0257E-06	1.5106E-05	0.72115309	0.71592454	0.69245476	0.64705415	0.60945744	0.55842863	0.49963687	0.45393591	0.41554785	0.37903743
2.0257E-06	1.6917E-05	0.72118829	0.71571692	0.69207406	0.64656195	0.6089349	0.55794173	0.49921877	0.45359289	0.41525869	0.37881123
2.0257E-06	1.8724E-05	0.72125076	0.71556162	0.69176155	0.64614705	0.60848986	0.55752366	0.4988587	0.45329271	0.415008	0.37861268
2.0257E-06	2.0526E-05	0.72133209	0.71544365	0.69149817	0.64578809	0.60810104	0.55715561	0.49854085	0.45302383	0.41478535	0.37843437
2.0257E-06	3.851E-05	0.72251946	0.7151014	0.69000409	0.64351698	0.6055473	0.55467035	0.49637355	0.45109649	0.41323438	0.37714493
2.0257E-06	5.6452E-05	0.723804	0.715208	0.68920011	0.64208603	0.60386363	0.55297959	0.4948834	0.4497018	0.41214374	0.37620392
2.0257E-06	9.2172E-05	0.72610926	0.71562027	0.68813371	0.63999344	0.60134206	0.55040769	0.49260511	0.44751876	0.41045855	0.37472551
2.0257E-06	0.00012773	0.72803453	0.71603819	0.68736849	0.63840417	0.5994027	0.54841377	0.49083427	0.44580213	0.4091418	0.37356087
2.0257E-06	0.00021561	0.73161299	0.7168795	0.68605613	0.63558906	0.59594407	0.54484275	0.48765848	0.44270489	0.40677384	0.3714576
2.0257E-06	0.00030251	0.73407569	0.71748126	0.68519175	0.63370069	0.59361535	0.54243281	0.48551369	0.44060632	0.40517225	0.37003179
2.0257E-06	0.00038845	0.73587013	0.71792599	0.68457259	0.63233821	0.59193272	0.54068993	0.48396213	0.43908628	0.40401297	0.36899886
2.0257E-06	0.00047345	0.73723495	0.7182667	0.68410584	0.63130721	0.59065848	0.53936945	0.48278643	0.43793371	0.40313425	0.36821555
2.0257E-06	0.0007992	0.74044705	0.71907445	0.6830174	0.62889343	0.5876729	0.53627404	0.48002998	0.43522967	0.40107347	0.36637765
2.0257E-06	0.00111183	0.74209975	0.71949241	0.68246139	0.62765656	0.5861421	0.53468632	0.47861596	0.4338418	0.40001605	0.36543427
2.0257E-06	0.00141237	0.74310721	0.7197478	0.68212348	0.62690388	0.5852103	0.53371973	0.47775507	0.43299664	0.39937221	0.36485976
2.0257E-06	0.00170172	0.74378594	0.71992007	0.6818962	0.62639726	0.58458304	0.53306898	0.47717547	0.43242757	0.39893871	0.36447292
2.0257E-06	0.00198068	0.74427455	0.72004419	0.68173276	0.62603277	0.58413171	0.53260073	0.47675841	0.43201805	0.39862677	0.36419453
2.0257E-06	0.00224997	0.74464331	0.72013792	0.68160949	0.6257578	0.5837912	0.53224745	0.47644374	0.43170905	0.39839141	0.36398448
2.0257E-06	0.00251023	0.74493165	0.72021124	0.68151316	0.62554286	0.58352503	0.53197128	0.47619775	0.43146749	0.39820742	0.36382027
2.0257E-06	0.00276206	0.74516341	0.72027018	0.68143577	0.62537014	0.58331113	0.53174934	0.47600007	0.43127336	0.39805955	0.3636883

Table S19a fitted data for the UV-Vis titration measurement 3 of **(±)-Zr(1)$_2$** with **G1**

[H] [M]	[G] [M]	410 nm	409 nm	408 nm	407 nm	406 nm	405 nm	404 nm	403 nm	402 nm	401 nm	400 nm
2.0257E-06	0	0.12824523	0.1511887	0.1769453	0.2152638	0.25386338	0.30944882	0.36337794	0.44550733	0.51853095	0.59926121	0.65080975
2.0257E-06	1.9374E-07	0.13050217	0.15372059	0.17980511	0.21847318	0.25717771	0.31284704	0.36718397	0.44897355	0.52107716	0.60041664	0.65053805
2.0257E-06	3.87E-07	0.13268553	0.15617009	0.18257191	0.22157827	0.26038452	0.31613524	0.37086666	0.45232764	0.52354132	0.60153534	0.6502761
2.0257E-06	5.7978E-07	0.13478422	0.15852476	0.18523167	0.22456336	0.2634676	0.31929685	0.37440742	0.45555269	0.52591108	0.60261179	0.65002538
2.0257E-06	7.7208E-07	0.13678607	0.16077097	0.187769	0.2274112	0.26640918	0.32231368	0.37778585	0.45863016	0.52817287	0.6036399	0.6497875
2.0257E-06	9.6391E-07	0.13867833	0.16289445	0.19016779	0.23010372	0.26919062	0.32516669	0.38098061	0.46154065	0.53031249	0.60461334	0.64956417
2.0257E-06	1.1553E-06	0.14044855	0.16488128	0.19241232	0.2326233	0.27179377	0.32783727	0.38397083	0.46426518	0.53231607	0.6055259	0.64935704
2.0257E-06	1.5361E-06	0.14357843	0.16839514	0.19638235	0.23708056	0.27640013	0.33256464	0.3892631	0.46908861	0.53586552	0.60714616	0.64899727
2.0257E-06	1.9151E-06	0.14613399	0.17126582	0.19962636	0.24072389	0.28016734	0.33643347	0.39359278	0.47303693	0.53877478	0.60847992	0.64871378
2.0257E-06	2.2922E-06	0.14813877	0.17351971	0.20217413	0.24358666	0.2831298	0.33947896	0.39699934	0.47614601	0.54107005	0.6095389	0.64850341
2.0257E-06	2.6675E-06	0.14967491	0.17524875	0.20412942	0.24578519	0.28540743	0.34182376	0.3996203	0.47854086	0.54284277	0.61036396	0.64835516
2.0257E-06	3.041E-06	0.15084606	0.17656902	0.20562328	0.24746639	0.28715166	0.3436228	0.40162939	0.48037939	0.54420843	0.61100673	0.64825517
2.0257E-06	3.4127E-06	0.15174706	0.17758671	0.20677556	0.24876461	0.28850098	0.34501773	0.40318544	0.48180598	0.54527263	0.61151443	0.64819078
2.0257E-06	3.7827E-06	0.15245198	0.17838476	0.20767989	0.24978482	0.28956362	0.34611927	0.40441258	0.48293349	0.5461179	0.61192397	0.6481521
2.0257E-06	4.1509E-06	0.15301458	0.17902337	0.20840422	0.25060319	0.29041809	0.34700773	0.40540087	0.48384378	0.54680411	0.61226212	0.64813194
2.0257E-06	5.9875E-06	0.15470273	0.18095628	0.21060319	0.25309971	0.2930451	0.34976591	0.40845456	0.48667833	0.54897805	0.61338855	0.64817755
2.0257E-06	7.8198E-06	0.15558153	0.181982	0.21177778	0.25444719	0.29448644	0.35130966	0.41014738	0.48827456	0.55024412	0.6141057	0.64832539
2.0257E-06	9.6478E-06	0.15616118	0.18267183	0.21257288	0.2553686	0.29548742	0.35240147	0.41133427	0.48940964	0.5511708	0.61466787	0.64850751
2.0257E-06	1.1472E-05	0.1565992	0.18320222	0.21318765	0.25608721	0.29627828	0.35327688	0.41227931	0.49032369	0.55193378	0.61515372	0.64870306
2.0257E-06	1.3291E-05	0.15695856	0.1836437	0.21370172	0.25669233	0.29695108	0.35403014	0.41308815	0.49111278	0.55260338	0.61559478	0.64890379
2.0257E-06	1.5106E-05	0.15726912	0.18402972	0.21415288	0.25722631	0.29754953	0.35470599	0.41381095	0.49182253	0.55321304	0.61600611	0.64910588
2.0257E-06	1.6917E-05	0.15754683	0.18437817	0.2145613	0.25771181	0.29809703	0.35532842	0.41447455	0.4924774	0.5537807	0.61639581	0.64930737
2.0257E-06	1.8724E-05	0.15780098	0.18469948	0.21493878	0.25816206	0.29860723	0.35591142	0.41509464	0.49309167	0.55431687	0.61676868	0.64950716
2.0257E-06	2.0526E-05	0.15803736	0.18500015	0.21529267	0.25858533	0.29908869	0.3564638	0.41568107	0.49367432	0.55482817	0.61712774	0.64970462
2.0257E-06	3.851E-05	0.15991895	0.18743383	0.21817142	0.26205351	0.30307371	0.36108391	0.42056241	0.49856171	0.55917602	0.62025639	0.65153316

2.0257E-06	7.4312E-05	0.1625856	0.19093354	0.22232879	0.26709295	0.30891293	0.36791177	0.42774814	0.50580137	0.56568667	0.62502978	0.6544467
2.0257E-06	0.00010995	0.16452884	0.19349521	0.22537577	0.27079322	0.31321115	0.37295035	0.43304473	0.51114746	0.57050954	0.62858458	0.6566423
2.0257E-06	0.00014542	0.16602751	0.19547349	0.22772974	0.27365347	0.31653608	0.37685092	0.43714363	0.51528692	0.57424734	0.63134393	0.65835249
2.0257E-06	0.00023311	0.16861265	0.19888877	0.23179458	0.27859423	0.32228216	0.3835949	0.44422904	0.52244483	0.58071441	0.63612267	0.66132053
2.0257E-06	0.00031982	0.17027001	0.20107947	0.23440231	0.28176456	0.32597028	0.38792474	0.44877751	0.52704078	0.58486825	0.6391939	0.66323051
2.0257E-06	0.00040557	0.17142362	0.20260464	0.23621793	0.28397209	0.32853867	0.39094039	0.45194525	0.53024186	0.58776183	0.64133386	0.66456207
2.0257E-06	0.00049038	0.17227295	0.20372766	0.23755486	0.28559768	0.33043011	0.39316135	0.45427815	0.53259944	0.58989311	0.64291028	0.66554327
2.0257E-06	0.00081544	0.17418345	0.20625413	0.24056269	0.28925514	0.33468601	0.39815906	0.45952758	0.53790466	0.59468953	0.64645851	0.66775249
2.0257E-06	0.00112745	0.17512142	0.20749465	0.24203961	0.29105111	0.33677597	0.40061345	0.46210553	0.54051012	0.59704527	0.64820143	0.66883796
2.0257E-06	0.00142741	0.17567913	0.20823228	0.24291781	0.29211905	0.33801875	0.40207298	0.46363851	0.54205949	0.59844619	0.64923797	0.66948358
2.0257E-06	0.00171622	0.17604905	0.20872156	0.24350035	0.29282745	0.33884315	0.40304116	0.46465541	0.54308727	0.59937552	0.64992559	0.66991191
2.0257E-06	0.00199468	0.17631253	0.20907005	0.24391526	0.29333201	0.33943033	0.40373076	0.46537971	0.54381933	0.60003745	0.65041538	0.67021701
2.0257E-06	0.0022635	0.17650983	0.20933102	0.24422597	0.29370986	0.33987005	0.40424719	0.46592212	0.54436755	0.60053316	0.65078218	0.67044551
2.0257E-06	0.00252333	0.17666319	0.20953387	0.24446748	0.29400355	0.34021184	0.4046486	0.46634373	0.54479368	0.60091848	0.6510673	0.67062312
2.0257E-06	0.00277476	0.17678587	0.20969615	0.24466068	0.29423851	0.34048528	0.40496974	0.46668102	0.54513458	0.60122674	0.65129539	0.67076522

Table S19b fitted data for the UV-Vis titration measurement 3 of **(±)-Zr(1)$_2$** with **G1**

[H] [M]	[G] [M]	399 nm	398 nm	397 nm	396 nm	395 nm	394 nm	393 nm	392 nm	391 nm	390 nm
2.0257E-06	0	0.69659398	0.71479825	0.70560627	0.6697409	0.63203482	0.57647733	0.51087378	0.45206494	0.4060405	0.36148219
2.0257E-06	1.9374E-07	0.69451999	0.71095594	0.70072335	0.66421752	0.62653568	0.57138632	0.50648482	0.44872924	0.40340933	0.35952816
2.0257E-06	3.87E-07	0.69251436	0.7072395	0.6960001	0.65887456	0.6212161	0.56646144	0.50223902	0.44550213	0.40086369	0.35763754
2.0257E-06	5.7978E-07	0.69058741	0.70366792	0.69146058	0.65373922	0.61610315	0.56172774	0.49815796	0.4424	0.3984165	0.3558199
2.0257E-06	7.7208E-07	0.68875046	0.70026204	0.68713124	0.64884137	0.61122655	0.55721272	0.49426533	0.43944081	0.39608191	0.35408573
2.0257E-06	9.6391E-07	0.68701538	0.69704367	0.68303972	0.64421225	0.60661741	0.55294515	0.49058594	0.43664338	0.39387472	0.35244601
2.0257E-06	1.1553E-06	0.68539371	0.6940341	0.67921304	0.63988237	0.60230609	0.54895314	0.48714398	0.43402606	0.3918094	0.35091145
2.0257E-06	1.5361E-06	0.68253196	0.68871742	0.67245066	0.63222941	0.59468544	0.54189617	0.48105892	0.42939742	0.38815609	0.34819617
2.0257E-06	1.9151E-06	0.68020405	0.68438347	0.6669347	0.62598484	0.58846646	0.53613604	0.47609133	0.42561647	0.38517049	0.34597585
2.0257E-06	2.2922E-06	0.67838804	0.68099187	0.66261399	0.62109085	0.58359164	0.53161955	0.47219538	0.42264845	0.38282519	0.34423019
2.0257E-06	2.6675E-06	0.67700754	0.6784021	0.65931031	0.61734606	0.57986055	0.52816128	0.46921129	0.42037217	0.38102478	0.34288845
2.0257E-06	3.041E-06	0.6759661	0.67643667	0.65679858	0.61449619	0.57702013	0.52552708	0.46693732	0.41863462	0.37964873	0.34186131
2.0257E-06	3.4127E-06	0.67517553	0.67493331	0.65487301	0.61230872	0.57483896	0.52350287	0.46518899	0.41729587	0.37858685	0.34106709
2.0257E-06	3.7827E-06	0.67456693	0.67376521	0.65337278	0.61060192	0.57313621	0.52192133	0.46382213	0.41624657	0.377753	0.34044196
2.0257E-06	4.1509E-06	0.67409029	0.67284037	0.65218122	0.60924398	0.57178068	0.5206611	0.46273216	0.4154074	0.3770847	0.33993959
2.0257E-06	5.9875E-06	0.6727501	0.67013877	0.64866292	0.60521146	0.5677473	0.51689927	0.45947069	0.41287226	0.37505174	0.33839813
2.0257E-06	7.8198E-06	0.67215772	0.66881835	0.64689825	0.60316155	0.56568746	0.51496398	0.45778352	0.41153255	0.37396108	0.33755585
2.0257E-06	9.6478E-06	0.67183879	0.66800605	0.64577988	0.60184308	0.564356	0.5137032	0.45667797	0.41063527	0.37321963	0.33697313
2.0257E-06	1.1472E-05	0.67164692	0.66743234	0.64496596	0.60086977	0.56336844	0.51276121	0.45584752	0.40994789	0.37264424	0.3365142
2.0257E-06	1.3291E-05	0.6715237	0.66698958	0.64431991	0.60008725	0.56257116	0.51199585	0.45516966	0.40937746	0.37216166	0.33612474
2.0257E-06	1.5106E-05	0.67144149	0.66662676	0.64377701	0.59942237	0.56189133	0.51133973	0.45458631	0.40887987	0.37173716	0.33577898
2.0257E-06	1.6917E-05	0.67138561	0.66631672	0.64330273	0.59883607	0.56129009	0.51075688	0.45406645	0.40843158	0.37135214	0.33546313
2.0257E-06	1.8724E-05	0.67134753	0.66604364	0.64287698	0.59830564	0.5607448	0.51022632	0.45359202	0.40801884	0.37099577	0.33516913
2.0257E-06	2.0526E-05	0.67132199	0.66579772	0.64248726	0.5978169	0.56024136	0.50973502	0.45315176	0.40763307	0.37066128	0.33489194
2.0257E-06	3.851E-05	0.67133652	0.6640181	0.63952346	0.59402867	0.55631648	0.50587193	0.44966939	0.40452109	0.36793181	0.33260316
2.0257E-06	7.4312E-05	0.67163036	0.66171904	0.63549654	0.58878778	0.55085737	0.50045674	0.44476172	0.40005877	0.36397952	0.32925602
2.0257E-06	0.00010995	0.67190596	0.66009388	0.63260108	0.58499745	0.54690248	0.49652404	0.44119165	0.39679533	0.36108052	0.32679364
2.0257E-06	0.00014542	0.67213285	0.65885222	0.63037714	0.58208098	0.5438578	0.49349419	0.43843978	0.39427577	0.35884034	0.32488917
2.0257E-06	0.00023311	0.67253952	0.65672291	0.62655066	0.57705735	0.53861166	0.48827118	0.43369448	0.38992674	0.35497143	0.32159826
2.0257E-06	0.00031982	0.67280628	0.65536272	0.62410129	0.57383947	0.53525058	0.48492396	0.43065282	0.38713737	0.35248917	0.31948613
2.0257E-06	0.00040557	0.67299377	0.65441742	0.62239755	0.57160051	0.53291177	0.48259452	0.42853585	0.38519548	0.35076083	0.3180153
2.0257E-06	0.00049038	0.67313253	0.65372206	0.62114366	0.56995245	0.53119014	0.48087967	0.42697734	0.38376566	0.34948814	0.31693214
2.0257E-06	0.00081544	0.67344642	0.65215933	0.61832424	0.56624607	0.52731812	0.4770226	0.42347176	0.38054905	0.34662479	0.314495
2.0257E-06	0.00112745	0.67360124	0.65139268	0.61694048	0.56442674	0.52541739	0.47512911	0.42175074	0.37896969	0.34521878	0.3132982
2.0257E-06	0.00142741	0.67369348	0.650937	0.61611784	0.56334509	0.52428733	0.47400332	0.42072748	0.3780306	0.34438274	0.31258653
2.0257E-06	0.00171622	0.67375474	0.6506348	0.61557223	0.56262765	0.52353778	0.47325658	0.42004875	0.37740769	0.34382817	0.31211445
2.0257E-06	0.00199468	0.67379839	0.65041958	0.61518364	0.56211668	0.52300393	0.47272474	0.41956534	0.37696402	0.34343318	0.31177821
2.0257E-06	0.0022635	0.6738311	0.65025844	0.61489266	0.56173406	0.52260417	0.47232648	0.41920334	0.37663178	0.34313738	0.31152641
2.0257E-06	0.00252333	0.67385654	0.65013319	0.61466649	0.56143665	0.52229345	0.47201692	0.41892196	0.37637353	0.34290746	0.31133069
2.0257E-06	0.00277476	0.67387689	0.65003299	0.61448556	0.56119873	0.52204487	0.47176928	0.41869687	0.37616693	0.34272353	0.31117411

Host-guest NMR titration data and fit for guests **G2**, **G3R** and **G3S**

Table S20: NMR titration data and fit for **$H_2$1** with **G2**. Data is given as the product of the chemical shift and the host concentration.

proton		3,4,13,14		8,9,18,19		27a		27B		28A		28B	
K		538.0042821		498.0877025		285.0462248		378.5823388		403.3715621		394.0658	
R^2 of the fit		0.998568009		0.997996674		0.997201601		0.999754606		0.999729899		0.9998138	
[H] [M]	[G] [M]	observed	fitted	observed	fitted	observed	fitted	observed	fitted	observed	fitted	observed	fitted
0.000204	0	0.001657	0.001657	0.001801	0.001801	0.000846	0.000846	0.00081	0.00081	0.000711	0.000711	0.000654	0.000654
0.000204	2.07E-05	0.001657	0.001657	0.001801	0.001801	0.000846	0.000845	0.000809	0.000809	0.000709	0.00071	0.000652	0.000653
0.000204	3.46E-05	0.001658	0.001658	0.001801	0.001801	0.000846	0.000845	0.000808	0.000809	0.000709	0.000709	0.000652	0.000652
0.000204	6.92E-05	0.001658	0.001658	0.001802	0.001802	0.000845	0.000844	0.000807	0.000807	0.000708	0.000708	0.000651	0.000651
0.000204	0.000138	0.001659	0.001659	0.001802	0.001802	0.000843	0.000843	0.000806	0.000805	0.000705	0.000705	0.000648	0.000648
0.000204	0.000173	0.001659	0.001659	0.001802	0.001802	0.000843	0.000842	0.000805	0.000804	0.000703	0.000703	0.000647	0.000647
0.000204	0.000221	0.001659	0.00166	0.001803	0.001803	0.000842	0.000841	0.000803	0.000803	0.000702	0.000701	0.000646	0.000645
0.000204	0.000263	0.00166	0.00166	0.001803	0.001803	0.000841	0.000841	0.000802	0.000802	0.0007	0.000699	0.000644	0.000644
0.000204	0.00036	0.001661	0.001661	0.001803	0.001803	0.000839	0.000839	0.000799	0.0008	0.000696	0.000696	0.000641	0.000641
0.000204	0.000526	0.001662	0.001662	0.001804	0.001804	0.000836	0.000836	0.000795	0.000796	0.000689	0.00069	0.000635	0.000636
0.000204	0.000706	0.001664	0.001664	0.001805	0.001805	0.000833	0.000833	0.000792	0.000792	0.000684	0.000684	0.000631	0.000631
0.000204	0.000885	0.001665	0.001665	0.001806	0.001806	0.000831	0.000831	0.000788	0.000788	0.000679	0.000679	0.000626	0.000626
0.000204	0.001314	0.001667	0.001667	0.001807	0.001807	0.000825	0.000825	0.000781	0.000781	0.000669	0.000668	0.000618	0.000617
0.000204	0.001729	0.001669	0.001669	0.001808	0.001808	0.000821	0.000821	0.000775	0.000776	0.00066	0.00066	0.00061	0.00061

proton	32A	32B	NH protons
K	529.4025046	426.042313	369.3632487
R^2 of the fit	0.997925269	0.99953637	0.993904112

[H] [M]	[G] [M]	observed	fitted	observed	fitted	observed	fitted
0.000204	0	0.000861	0.000861	0.000763	0.000763	-0.00057	-0.00057
0.000204	2.07E-05	0.000861	0.000861	0.000763	0.000763	-0.00057	-0.00057
0.000204	3.46E-05	0.000862	0.000861	0.000763	0.000763	-0.00057	-0.00057
0.000204	6.92E-05	0.000862	0.000862	0.000764	0.000764	-0.00057	-0.00057
0.000204	0.000138	0.000862	0.000862	0.000764	0.000764	-0.00057	-0.00057
0.000204	0.000173	0.000862	0.000862	0.000764	0.000764	-0.00057	-0.00057
0.000204	0.000221	0.000863	0.000863	0.000765	0.000765	-0.00057	-0.00057
0.000204	0.000263	0.000863	0.000863	0.000765	0.000765	-0.00057	-0.00057
0.000204	0.00036	0.000863	0.000863	0.000766	0.000766	-0.00057	-0.00057
0.000204	0.000526	0.000864	0.000864	0.000767	0.000767	-0.00056	-0.00056
0.000204	0.000706	0.000865	0.000865	0.000768	0.000768	-0.00056	-0.00056
0.000204	0.000885	0.000866	0.000866	0.000769	0.000769	-0.00056	-0.00056
0.000204	0.001314	0.000867	0.000867	0.000771	0.000771	-0.00056	-0.00056
0.000204	0.001729	0.000869	0.000868	0.000772	0.000772	-0.00056	-0.00056

Table S21: NMR titration data and fit for **$H_2$1** with **G2**. Data is given as the product of the chemical shift and the host concentration. Duplo experiment.

proton		3,4,13,14		8,9,18,19		27a		27B		28A		28B	
K		319.7708743		368.5353654		308.3920691		291.2737015		294.3760554		301.5361	
R^2 of the fit		0.999750556		0.993225825		0.999786351		0.999691042		0.999796391		0.9997485	
[H] [M]	[G] [M]	observed	fitted	observed	fitted	observed	fitted	observed	fitted	observed	fitted	observed	fitted
0.000212	0	0.001714	0.001714	0.001864	0.001864	0.000875	0.000875	0.000843	0.000843	0.000741	0.000741	0.000675	0.000675
0.000212	2.31E-05	0.001714	0.001714	0.001864	0.001864	0.000874	0.000875	0.000842	0.000842	0.00074	0.00074	0.000674	0.000674
0.000212	3.87E-05	0.001714	0.001714	0.001864	0.001864	0.000874	0.000874	0.000842	0.000842	0.000739	0.000739	0.000674	0.000674
0.000212	8.49E-05	0.001715	0.001715	0.001864	0.001864	0.000873	0.000874	0.000841	0.000841	0.000738	0.000738	0.000672	0.000672
0.000212	0.00017	0.001715	0.001715	0.001864	0.001865	0.000872	0.000872	0.000839	0.000839	0.000735	0.000735	0.00067	0.00067
0.000212	0.000209	0.001716	0.001716	0.001865	0.001865	0.000871	0.000871	0.000838	0.000838	0.000734	0.000733	0.000669	0.000669
0.000212	0.000263	0.001716	0.001716	0.001865	0.001865	0.000871	0.00087	0.000837	0.000837	0.000732	0.000732	0.000668	0.000667
0.000212	0.000317	0.001716	0.001717	0.001865	0.001865	0.00087	0.00087	0.000836	0.000836	0.00073	0.00073	0.000666	0.000666
0.000212	0.000426	0.001717	0.001717	0.001866	0.001866	0.000868	0.000868	0.000833	0.000833	0.000726	0.000727	0.000663	0.000663
0.000212	0.000634	0.001719	0.001719	0.001866	0.001867	0.000865	0.000865	0.000829	0.00083	0.000721	0.000721	0.000658	0.000658
0.000212	0.00085	0.00172	0.00172	0.001867	0.001867	0.000862	0.000862	0.000826	0.000826	0.000716	0.000716	0.000654	0.000654
0.000212	0.00106	0.001721	0.001721	0.001868	0.001868	0.00086	0.00086	0.000823	0.000823	0.00071	0.000711	0.000649	0.000649
0.000212	0.001586	0.001723	0.001724	0.001869	0.00187	0.000855	0.000854	0.000816	0.000816	0.000701	0.0007	0.000641	0.000641
0.000212	0.002117	0.001726	0.001726	0.00187	0.001871	0.00085	0.00085	0.00081	0.00081	0.000692	0.000692	0.000633	0.000633
0.000212	0.005409	0.001733	0.001732	0.001875	0.001875	0.000835	0.000835	0.000789	0.000789	0.000661	0.000661	0.000607	0.000608

proton		32A		32B		NH protons	
K		284.8139752		303.3997713		417.7637253	
R^2 of the fit		0.999611574		0.999703984		0.99148379	
[H] [M]	[G] [M]	observed	fitted	observed	fitted	observed	fitted
0.000212	0	0.000884	0.000884	0.000789	0.000789	-0.00059	-0.00059
0.000212	2.31E-05	0.000885	0.000885	0.00079	0.00079	-0.00059	-0.00059
0.000212	3.87E-05	0.000885	0.000885	0.00079	0.00079	-0.00059	-0.00059
0.000212	8.49E-05	0.000885	0.000885	0.00079	0.00079	-0.00059	-0.00059
0.000212	0.00017	0.000885	0.000885	0.000791	0.000791	-0.00059	-0.00059
0.000212	0.000209	0.000885	0.000885	0.000791	0.000791	-0.00059	-0.00059
0.000212	0.000263	0.000886	0.000886	0.000791	0.000791	-0.00059	-0.00059
0.000212	0.000317	0.000886	0.000886	0.000791	0.000791	-0.00059	-0.00059
0.000212	0.000426	0.000887	0.000886	0.000792	0.000792	-0.00059	-0.00059
0.000212	0.000634	0.000887	0.000887	0.000793	0.000793	-0.00058	-0.00058
0.000212	0.00085	0.000888	0.000888	0.000794	0.000794	-0.00058	-0.00058
0.000212	0.00106	0.000889	0.000889	0.000795	0.000795	-0.00058	-0.00058
0.000212	0.001586	0.00089	0.00089	0.000797	0.000797	-0.00058	-0.00058
0.000212	0.002117	0.000892	0.000892	0.000799	0.000799	-0.00058	-0.00058
0.000212	0.005409	0.000896	0.000896	0.000804	0.000804	-0.00057	-0.00057

Table S22: NMR titration data and fit for **(±)-Zr(1)$_2$** with **G2**. Data is given as the product of the chemical shift and the host concentration.

proton		3,4,13,14		27a(I,III)		27a(II,IV)		27b(I,III)		27b(II,IV)		28a(I,III)	
K		2264.869841		2042.515422		1816.968222		2267.896101		2180.498514		2911.872	
α		0.466009868		0.531068967		0.462880278		0.525487555		0.52990514		0.719463	
R^2 of the fit		0.996598626		0.999291046		0.999678932		0.997453817		0.999019217		0.9981406	
[H] [M]	[G] [M]	observed	fitted	observed	fitted	observed	fitted	observed	fitted	observed	fitted	observed	fitted
0.00019	0	0.001517	0.001517	0.000636	0.000636	0.000599	0.000599	0.000529	0.000529	0.00051	0.00051	0.000555	0.000555
0.00019	2.08E-05	0.001517	0.001517	0.000637	0.000635	0.000598	0.000598	0.000528	0.000528	0.000509	0.000509	0.000553	0.000554
0.00019	4.16E-05	0.001517	0.001517	0.000635	0.000635	0.000597	0.000597	0.000527	0.000527	0.000508	0.000508	0.000552	0.000552
0.00019	9.01E-05	0.001517	0.001517	0.000633	0.000633	0.000595	0.000595	0.000524	0.000525	0.000505	0.000506	0.000548	0.000549
0.00019	0.00018	0.001518	0.001518	0.000629	0.000629	0.000591	0.000592	0.00052	0.00052	0.0005	0.000501	0.000541	0.000543
0.00019	0.000221	0.001518	0.001518	0.000627	0.000628	0.00059	0.000591	0.000518	0.000519	0.000499	0.000499	0.000539	0.00054
0.00019	0.000277	0.001519	0.001518	0.000625	0.000626	0.000589	0.000589	0.000515	0.000516	0.000497	0.000497	0.000536	0.000536
0.00019	0.000333	0.001519	0.001519	0.000624	0.000624	0.000587	0.000587	0.000513	0.000513	0.000494	0.000494	0.000532	0.000532
0.00019	0.000449	0.00152	0.00152	0.000619	0.000619	0.000583	0.000583	0.000509	0.000509	0.000489	0.000489	0.000525	0.000524
0.00019	0.000692	0.001521	0.001521	0.000612	0.000612	0.000577	0.000576	0.000501	0.0005	0.00048	0.000479	0.000512	0.000509
0.00019	0.000899	0.001522	0.001522	0.000606	0.000606	0.000571	0.000571	0.000493	0.000493	0.000473	0.000472	0.0005	0.000499
0.00019	0.001107	0.001523	0.001524	0.000601	0.000601	0.000566	0.000566	0.00049	0.000488	0.000466	0.000466	0.00049	0.000492
0.00019	0.00166	0.001525	0.001526	0.000591	0.000591	0.000557	0.000557	0.000476	0.000476	0.000453	0.000453	0.000476	0.000477
0.00019	0.002214	0.001527	0.001527	0.000583	0.000583	0.000549	0.000549	0.000465	0.000468	0.000442	0.000444	0.000465	0.000468
0.00019	0.004842	0.001532	0.001531	0.000566	0.000566	0.000531	0.000531	0.000452	0.00045	0.000424	0.000424	0.000452	0.00045

proton	30(I,III)	30(II,IV)	32a(I,III)	32a(II,IV)	32b	32b
K	2937.269973	2789.929888	2257.244641	1275.019345	1957.318766	1915.491

α		0.627490598		0.55743343		0.554075929		0.686950575		0.467602223		0.552623	
R^2 of the fit		0.997077664		0.995623985		0.996996244		0.999675477		0.999519621		0.9995813	
[H] [M]	[G] [M]	observed	fitted	observed	fitted	observed	fitted	observed	fitted	observed	fitted	observed	fitted
0.00019	0	0.001117	0.001117	0.001144	0.001144	0.000771	0.000771	0.000756	0.000756	0.00068	0.00068	0.000679	0.000679
0.00019	2.08E-05	0.001117	0.001117	0.001144	0.001144	0.000771	0.000771	0.000756	0.000756	0.000681	0.000681	0.00068	0.00068
0.00019	4.16E-05	0.001117	0.001117	0.001144	0.001144	0.000771	0.000771	0.000757	0.000757	0.000681	0.000681	0.00068	0.00068
0.00019	9.01E-05	0.001117	0.001117	0.001143	0.001144	0.000771	0.000771	0.000757	0.000757	0.000681	0.000681	0.000681	0.00068
0.00019	0.00018	0.001117	0.001117	0.001143	0.001143	0.000772	0.000772	0.000758	0.000758	0.000682	0.000682	0.000682	0.000681
0.00019	0.000221	0.001118	0.001118	0.001143	0.001143	0.000772	0.000772	0.000758	0.000758	0.000683	0.000683	0.000682	0.000682
0.00019	0.000277	0.001118	0.001118	0.001142	0.001142	0.000772	0.000772	0.000759	0.000759	0.000683	0.000683	0.000682	0.000682
0.00019	0.000333	0.001118	0.001118	0.001142	0.001142	0.000773	0.000773	0.000759	0.000759	0.000684	0.000684	0.000683	0.000683
0.00019	0.000449	0.001118	0.001118	0.001141	0.001141	0.000773	0.000773	0.00076	0.00076	0.000684	0.000685	0.000684	0.000684
0.00019	0.000692	0.001119	0.001119	0.00114	0.00114	0.000775	0.000775	0.000762	0.000762	0.000686	0.000686	0.000686	0.000686
0.00019	0.000899	0.00112	0.00112	0.001139	0.001139	0.000775	0.000776	0.000763	0.000763	0.000688	0.000688	0.000688	0.000688
0.00019	0.001107	0.00112	0.00112	0.001138	0.001138	0.000776	0.000776	0.000764	0.000764	0.000689	0.000689	0.000689	0.000689
0.00019	0.00166	0.001121	0.001121	0.001137	0.001137	0.000778	0.000778	0.000766	0.000766	0.000692	0.000692	0.000692	0.000692
0.00019	0.002214	0.001121	0.001122	0.001135	0.001135	0.000779	0.000779	0.000768	0.000768	0.000693	0.000694	0.000693	0.000694
0.00019	0.004842	0.001123	0.001123	0.001133	0.001133	0.000782	0.000782	0.000772	0.000772	0.000698	0.000698	0.000698	0.000698

Table S23: NMR titration data and fit for **(±)-Zr(1)$_2$** with **G2**. Data is given as the product of the chemical shift and the host concentration. Duplo experiment.

proton		3,4,13,14		27a(I,III)		27a(II,IV)		27b(I,III)		27b(II,IV)		28a(I,III)	
K		2374.655624		1251.689806		1866.855849		2016.882038		1799.0163		2693.79096	
α		0.504560769		0.536476103		0.361196251		0.494656132		0.462203061		0.637642876	
R^2 of the fit		0.989455886		0.999689969		0.999756743		0.99916709		0.99905053		0.999157524	
[H] [M]	[G] [M]	observed	fitted	observed	fitted	observed	fitted	observed	fitted	observed	fitted	observed	fitted
0.000194	0	0.001548	0.001548	0.00065	0.00065	0.000611	0.000611	0.000539	0.000539	0.000522	0.000522	0.000566	0.000566
0.000194	2.50E-05	0.001548	0.001548	0.000648	0.000649	0.000609	0.00061	0.000538	0.000538	0.000521	0.000521	0.000563	0.000564
0.000194	3.34E-05	0.001548	0.001548	0.000647	0.000648	0.000609	0.000609	0.000536	0.000537	0.000519	0.00052	0.000562	0.000563
0.000194	6.68E-05	0.001548	0.001548	0.000646	0.000646	0.000607	0.000608	0.000535	0.000535	0.000518	0.000518	0.00056	0.00056
0.000194	0.00015	0.001549	0.001549	0.000641	0.000642	0.000603	0.000604	0.000529	0.000531	0.000511	0.000513	0.000552	0.000554
0.000194	0.0002	0.001549	0.001549	0.00064	0.00064	0.000602	0.000602	0.000528	0.000528	0.000511	0.000511	0.00055	0.00055
0.000194	0.00025	0.00155	0.001549	0.000638	0.000638	0.0006	0.0006	0.000526	0.000526	0.000508	0.000508	0.000546	0.000546
0.000194	0.0003	0.00155	0.00155	0.000636	0.000636	0.000598	0.000598	0.000524	0.000523	0.000506	0.000506	0.000542	0.000542
0.000194	0.000384	0.001551	0.00155	0.000633	0.000632	0.000595	0.000595	0.00052	0.00052	0.000502	0.000502	0.000537	0.000536
0.000194	0.000584	0.001552	0.001552	0.000625	0.000626	0.000589	0.000589	0.000511	0.000512	0.000493	0.000493	0.000525	0.000524
0.000194	0.000784	0.001553	0.001553	0.000619	0.00062	0.000583	0.000584	0.000505	0.000505	0.000486	0.000486	0.000515	0.000514
0.000194	0.000968	0.001554	0.001554	0.000616	0.000615	0.000579	0.000579	0.000501	0.0005	0.000481	0.00048	0.000504	0.000506
0.000194	0.001452	0.001556	0.001556	0.000606	0.000606	0.000571	0.00057	0.000489	0.000489	0.000469	0.000468	0.000492	0.000491
0.000194	0.002086	0.001558	0.001559	0.000597	0.000597	0.000562	0.000562	0.000477	0.000478	0.000455	0.000456	0.000477	0.000479
0.000194	0.005841	0.001565	0.001564	0.000573	0.000573	0.000537	0.000537	0.000454	0.000454	0.000427	0.000427	0.000454	0.000454

proton		30(I,III)		30(II,IV)		32a(I,III)		32a(II,IV)		32b		32b	
K		2358.946098		2551.223244		1425.976365		927.3825429		1727.960615		1587.318449	
α		0.479289463		0.52000856		0.43883619		0.637160666		0.393174371		0.49509744	
R^2 of the fit		0.998478487		0.998666936		0.999262241		0.999489509		0.999459471		0.99968643	
[H] [M]	[G] [M]	observed	fitted	observed	fitted	observed	fitted	observed	fitted	observed	fitted	observed	fitted
0.000194	0	0.001138	0.001138	0.001167	0.001167	0.000786	0.000786	0.000771	0.000771	0.000694	0.000694	0.000693	0.000693
0.000194	2.50E-05	0.001139	0.001139	0.001166	0.001167	0.000786	0.000786	0.000771	0.000771	0.000694	0.000694	0.000693	0.000693
0.000194	3.34E-05	0.001139	0.001139	0.001166	0.001166	0.000786	0.000786	0.000771	0.000771	0.000694	0.000694	0.000693	0.000693
0.000194	6.68E-05	0.001139	0.001139	0.001166	0.001166	0.000786	0.000786	0.000772	0.000772	0.000695	0.000694	0.000694	0.000694
0.000194	0.00015	0.001139	0.001139	0.001165	0.001166	0.000787	0.000787	0.000773	0.000772	0.000696	0.000695	0.000695	0.000695
0.000194	0.0002	0.001139	0.001139	0.001165	0.001165	0.000787	0.000787	0.000773	0.000773	0.000696	0.000696	0.000695	0.000695
0.000194	0.00025	0.00114	0.00114	0.001165	0.001165	0.000787	0.000788	0.000773	0.000773	0.000696	0.000696	0.000696	0.000696
0.000194	0.0003	0.00114	0.00114	0.001164	0.001164	0.000788	0.000788	0.000774	0.000774	0.000697	0.000697	0.000696	0.000696
0.000194	0.000384	0.00114	0.00114	0.001164	0.001164	0.000788	0.000788	0.000774	0.000774	0.000698	0.000698	0.000697	0.000697
0.000194	0.000584	0.001141	0.001141	0.001163	0.001163	0.000789	0.000789	0.000776	0.000776	0.0007	0.000699	0.000699	0.000699
0.000194	0.000784	0.001141	0.001141	0.001162	0.001162	0.00079	0.00079	0.000777	0.000777	0.000701	0.000701	0.000701	0.0007
0.000194	0.000968	0.001142	0.001142	0.001161	0.001161	0.000791	0.000791	0.000778	0.000778	0.000702	0.000702	0.000702	0.000702
0.000194	0.001452	0.001143	0.001143	0.00116	0.001159	0.000793	0.000793	0.00078	0.00078	0.000704	0.000704	0.000704	0.000704
0.000194	0.002086	0.001143	0.001143	0.001158	0.001158	0.000794	0.000794	0.000782	0.000782	0.000707	0.000707	0.000707	0.000707
0.000194	0.005841	0.001145	0.001145	0.001155	0.001155	0.000799	0.000799	0.000788	0.000788	0.000713	0.000713	0.000713	0.000713

Table S24: NMR titration data and fit for **(+)-Zr(1)$_2$** with **G3R**. Data is given as the product of the chemical shift with the host concentration.

proton		3,4,13,14		27a(I,III)		27a(II,IV)		27b(I,III)		27b(II,IV)		28a(I,III)	
K		627.1960494		191.5968815		321.1519418		219.6112679		330.4938126		199.9525	
α		0.531073655		0.522087577		0.323626447		0.511306727		0.620268911		0.503972	
R^2 of the fit		0.999003451		0.990772623		0.996369014		0.898425792		0.997327687		0.974251	
[H] [M]	[G] [M]	observed	fitted	observed	fitted	observed	fitted	observed	fitted	observed	fitted	observed	fitted
0.001117	0	0.008903	0.008903	0.003733	0.003733	0.003512	0.003512	0.003095	0.003095	0.002988	0.002988	0.003249	0.003249
0.001118	0.000162	0.008908	0.008909	0.003732	0.003732	0.003507	0.003507	0.003095	0.003095	0.002981	0.00298	0.003247	0.003248
0.001118	0.00032	0.008912	0.008914	0.003729	0.00373	0.0035	0.003501	0.003095	0.003094	0.002972	0.002973	0.003246	0.003247
0.001119	0.000476	0.00892	0.00892	0.003729	0.003729	0.003494	0.003495	0.003093	0.003094	0.002962	0.002965	0.003244	0.003246
0.00112	0.000778	0.008931	0.008931	0.003725	0.003726	0.003483	0.003484	0.003092	0.003093	0.002946	0.00295	0.003242	0.003244
0.001121	0.001069	0.008945	0.008942	0.003724	0.003723	0.003473	0.003474	0.003092	0.003092	0.002932	0.002935	0.003242	0.003242
0.001122	0.001349	0.008955	0.008953	0.003721	0.003721	0.003463	0.003464	0.003091	0.003091	0.002915	0.00292	0.00324	0.00324
0.001123	0.001619	0.008968	0.008964	0.003718	0.003719	0.003454	0.003455	0.003092	0.003091	0.002907	0.002906	0.003238	0.003238
0.001124	0.00213	0.008986	0.008984	0.003717	0.003715	0.003437	0.003438	0.003089	0.003089	0.002879	0.002879	0.003236	0.003236
0.001128	0.003267	0.00903	0.009029	0.003707	0.003708	0.003403	0.003402	0.003088	0.003087	0.002831	0.002824	0.003232	0.003231
0.001131	0.004235	0.009063	0.009065	0.003704	0.003704	0.003377	0.003375	0.003084	0.003086	0.00279	0.002781	0.003229	0.003228
0.001134	0.005069	0.009084	0.009095	0.003701	0.003701	0.003355	0.003353	0.003085	0.003085	0.002755	0.002749	0.003227	0.003227
0.001138	0.006434	0.009139	0.009143	0.003699	0.003697	0.003327	0.003322	0.003087	0.003085	0.002685	0.002703	0.003226	0.003226
0.001151	0.01017	0.009269	0.009264	0.003694	0.003697	0.003239	0.003255	0.003087	0.00309	0.002608	0.002615	0.003226	0.003229
0.001156	0.011859	0.009319	0.009316	0.003701	0.003699	0.003243	0.003233	0.003096	0.003094	0.002596	0.002588	0.003235	0.003233

proton		30(I,III)		30(II,IV)		32a(I,III)		32a(II,IV)		32b		32b	
K		626.181745		646.0405669		508.0517726		432.048428		524.0057953		281.557	
α		0.752772118		0.755859653		0.693226998		0.621868456		0.673127162		0.754384	

R² of the fit		0.999728697		0.999948057		0.99973292		0.999814715		0.999512175		0.9999034	
[H] [M]	[G] [M]	observed	fitted	observed	fitted	observed	fitted	observed	fitted	observed	fitted	observed	fitted
0.001117	0	0.006543	0.006543	0.006699	0.006699	0.004503	0.004503	0.004419	0.004419	0.003976	0.003976	0.003981	0.003981
0.001118	0.000162	0.006547	0.006547	0.006702	0.006702	0.004506	0.004506	0.004422	0.004423	0.00398	0.00398	0.003984	0.003985
0.001118	0.00032	0.006551	0.006551	0.006705	0.006705	0.00451	0.004509	0.004426	0.004426	0.003984	0.003983	0.003988	0.003988
0.001119	0.000476	0.006556	0.006555	0.006708	0.006708	0.004512	0.004512	0.004429	0.00443	0.003987	0.003986	0.003992	0.003992
0.00112	0.000778	0.006565	0.006564	0.006713	0.006714	0.004519	0.004519	0.004436	0.004436	0.003994	0.003993	0.003998	0.003999
0.001121	0.001069	0.006574	0.006572	0.00672	0.00672	0.004527	0.004525	0.004444	0.004443	0.004002	0.004	0.004007	0.004005
0.001122	0.001349	0.006581	0.00658	0.006725	0.006725	0.004532	0.004532	0.004451	0.00445	0.004008	0.004007	0.004012	0.004012
0.001123	0.001619	0.006589	0.006588	0.00673	0.006731	0.004538	0.004538	0.004457	0.004456	0.004014	0.004013	0.004017	0.004018
0.001124	0.00213	0.006604	0.006603	0.006741	0.00674	0.00455	0.004549	0.004469	0.004468	0.004026	0.004025	0.00403	0.00403
0.001128	0.003267	0.006634	0.006635	0.006763	0.006762	0.004574	0.004575	0.004495	0.004495	0.00405	0.004051	0.004055	0.004055
0.001131	0.004235	0.006659	0.006661	0.006782	0.006781	0.004594	0.004595	0.004515	0.004516	0.00407	0.004072	0.004075	0.004075
0.001134	0.005069	0.00668	0.006682	0.006798	0.006797	0.00461	0.004612	0.004533	0.004534	0.004087	0.004089	0.004091	0.004092
0.001138	0.006434	0.006714	0.006716	0.006825	0.006824	0.004636	0.004638	0.00456	0.004562	0.004113	0.004115	0.004117	0.004118
0.001151	0.01017	0.006803	0.006801	0.006897	0.006898	0.004705	0.004703	0.004632	0.00463	0.004181	0.004178	0.004184	0.004183
0.001156	0.011859	0.006839	0.006837	0.00693	0.006931	0.004731	0.00473	0.004659	0.004659	0.004204	0.004203	0.00421	0.00421

Table S25: NMR titration data and fit for **(+)-Zr(1)$_2$** with **G3R**. Data is given as the product of the chemical shift and the host concentration. Duplo experiment.

proton		3,4,13,14		27a(I,III)		27a(II,IV)		27b(I,III)		27b(II,IV)		28a(I,III)	
K		602.3836056		523.9141721		557.5069442		506.9938046		501.7003226		479.0828	
α		0.677470018		0.463912462		0.506829669		0.428828255		0.492390874		0.472663	
R² of the fit		0.999849067		0.999990224		0.999249717		0.999975032		0.999795994		0.9999831	
[H] [M]	[G] [M]	observed	fitted	observed	fitted	observed	fitted	observed	fitted	observed	fitted	observed	fitted
0.000656	0	0.00522	0.00522	0.002193	0.002193	0.002076	0.002076	0.001819	0.001819	0.001762	0.001762	0.00191	0.00191
0.000655	0.00018	0.005217	0.005217	0.002187	0.002187	0.002065	0.002065	0.001814	0.001814	0.00175	0.00175	0.001905	0.001904
0.000655	0.000357	0.005214	0.005213	0.002181	0.00218	0.002054	0.002054	0.00181	0.00181	0.001738	0.001737	0.001899	0.001899
0.000654	0.00053	0.00521	0.00521	0.002175	0.002174	0.002045	0.002044	0.001805	0.001805	0.001725	0.001725	0.001895	0.001894
0.000653	0.0007	0.005207	0.005206	0.002169	0.002169	0.002034	0.002035	0.001801	0.001801	0.001713	0.001714	0.001889	0.00189
0.000652	0.00103	0.005201	0.0052	0.002158	0.002158	0.002016	0.002016	0.001793	0.001793	0.001692	0.001692	0.001881	0.001881
0.000651	0.001348	0.005194	0.005194	0.002148	0.002148	0.001999	0.002	0.001786	0.001786	0.001672	0.001673	0.001872	0.001873
0.00065	0.001655	0.005188	0.005187	0.002139	0.002139	0.001985	0.001984	0.00178	0.001779	0.001654	0.001655	0.001865	0.001865
0.000647	0.002235	0.005175	0.005176	0.002123	0.002123	0.001958	0.001958	0.001767	0.001767	0.001623	0.001623	0.001852	0.001852
0.000642	0.003523	0.005145	0.005147	0.002092	0.002092	0.001907	0.001906	0.001743	0.001743	0.001565	0.001562	0.001826	0.001825
0.000638	0.004619	0.005118	0.00512	0.002068	0.002068	0.00187	0.00187	0.001725	0.001724	0.001522	0.001519	0.001805	0.001805
0.000634	0.005562	0.005094	0.005095	0.002049	0.002048	0.001839	0.001842	0.00171	0.001709	0.00148	0.001486	0.001789	0.001789
0.000628	0.007104	0.005053	0.005054	0.002019	0.002019	0.001798	0.001802	0.001686	0.001686	0.001441	0.001441	0.001763	0.001764
0.000618	0.009697	0.004981	0.00498	0.001975	0.001975	0.001757	0.001746	0.001651	0.00165	0.00138	0.001378	0.001726	0.001726
0.000608	0.012406	0.004904	0.004901	0.001933	0.001933	0.001692	0.001697	0.001615	0.001616	0.001326	0.001326	0.001689	0.001689

proton		30(I,III)		30(II,IV)		32a(I,III)		32a(II,IV)		32b		32b	
K		728.7680075		628.9466726		792.1398857		604.4988708		546.4705338		372.583185	
α		0.557453993		0.55090977		0.568779135		0.746854487		0.545450696		0.680247924	
R² of the fit		0.999953166		0.999998368		0.999917323		0.99981365		0.999970544		0.999986673	
[H] [M]	[G] [M]	observed	fitted	observed	fitted	observed	fitted	observed	fitted	observed	fitted	observed	fitted
0.000656	0	0.003845	0.003845	0.00394	0.00394	0.002654	0.002654	0.002603	0.002603	0.002342	0.002342	0.002339	0.002339
0.000655	0.00018	0.003842	0.003842	0.003935	0.003935	0.002652	0.002652	0.002602	0.002602	0.002342	0.002342	0.002339	0.002338
0.000655	0.000357	0.00384	0.003839	0.003931	0.003931	0.00265	0.00265	0.002601	0.002601	0.002341	0.002341	0.002338	0.002338
0.000654	0.00053	0.003837	0.003836	0.003926	0.003926	0.002649	0.002648	0.0026	0.002599	0.00234	0.00234	0.002337	0.002337
0.000653	0.0007	0.003834	0.003834	0.003922	0.003922	0.002647	0.002647	0.002599	0.002598	0.00234	0.00234	0.002337	0.002337
0.000652	0.00103	0.003829	0.003828	0.003914	0.003914	0.002644	0.002644	0.002596	0.002596	0.002338	0.002338	0.002335	0.002335
0.000651	0.001348	0.003824	0.003823	0.003906	0.003906	0.002641	0.002641	0.002594	0.002594	0.002337	0.002336	0.002333	0.002333
0.00065	0.001655	0.003818	0.003818	0.003898	0.003898	0.002638	0.002638	0.002591	0.002591	0.002335	0.002335	0.002332	0.002332
0.000647	0.002235	0.003808	0.003808	0.003884	0.003884	0.002632	0.002632	0.002586	0.002586	0.002331	0.002331	0.002328	0.002328
0.000642	0.003523	0.003784	0.003785	0.003853	0.003853	0.002617	0.002617	0.002573	0.002574	0.00232	0.00232	0.002318	0.002318
0.000638	0.004619	0.003763	0.003764	0.003827	0.003827	0.002603	0.002604	0.00256	0.002561	0.00231	0.00231	0.002307	0.002308
0.000634	0.005562	0.003744	0.003745	0.003804	0.003804	0.002591	0.002591	0.002549	0.00255	0.0023	0.002301	0.002298	0.002298
0.000628	0.007104	0.003713	0.003713	0.003768	0.003768	0.00257	0.00257	0.002529	0.002529	0.002284	0.002284	0.002281	0.002281
0.000618	0.009697	0.003658	0.003658	0.003707	0.003707	0.002532	0.002532	0.002494	0.002493	0.002253	0.002253	0.002251	0.002251
0.000608	0.012406	0.0036	0.003599	0.003643	0.003643	0.002492	0.002492	0.002455	0.002454	0.002219	0.002219	0.002217	0.002217

Table S26: NMR titration data and fit for **(+)-Zr(1)$_2$** with **G3S**. Data is given as the product of the chemical shift and the host concentration.

proton		3,4,13,14		27a(I,III)		27a(II,IV)		27b(I,III)		27b(II,IV)		28a(I,III)	
K		582.4452084		399.4933018		329.6964122		529.6627915		617.1910593		279.40912	
α		0.520360192		0.563277821		0.446020855		0.559274973		0.369579637		0.512108125	
R² of the fit		0.999942331		0.999537528		0.99841138		0.993415677		0.998335068		0.991639507	
[H] [M]	[G] [M]	observed	fitted	observed	fitted	observed	fitted	observed	fitted	observed	fitted	observed	fitted
0.001074	0	0.008554	0.008554	0.003589	0.003589	0.003373	0.003373	0.002977	0.002977	0.002883	0.002883	0.003126	0.003126
0.001075	0.000187	0.008566	0.008566	0.003582	0.003582	0.003372	0.003373	0.002968	0.002968	0.002884	0.002885	0.003125	0.003124
0.001076	0.00037	0.008578	0.008578	0.003575	0.003575	0.003372	0.003373	0.002956	0.002959	0.002885	0.002886	0.003123	0.003123
0.001078	0.00055	0.008591	0.008589	0.003569	0.003569	0.003373	0.003373	0.002947	0.00295	0.002887	0.002888	0.003123	0.003122
0.00108	0.000899	0.008613	0.008613	0.003556	0.003556	0.003373	0.003373	0.002927	0.002931	0.002889	0.002891	0.00312	0.00312
0.001082	0.001235	0.008637	0.008635	0.003544	0.003544	0.003375	0.003374	0.002909	0.002912	0.002893	0.002894	0.003119	0.003118
0.001084	0.001558	0.008658	0.008657	0.003532	0.003533	0.003375	0.003375	0.002889	0.002894	0.002894	0.002897	0.003117	0.003117
0.001086	0.00187	0.008679	0.008678	0.003521	0.003522	0.003376	0.003376	0.002876	0.002876	0.002899	0.002899	0.003117	0.003116
0.00109	0.002462	0.008718	0.008718	0.003505	0.003504	0.003378	0.003378	0.002848	0.002843	0.002905	0.002905	0.003115	0.003115
0.001099	0.003774	0.008804	0.008805	0.003471	0.003471	0.003385	0.003386	0.002791	0.00278	0.002918	0.002918	0.003114	0.003115
0.001107	0.004893	0.008876	0.008877	0.003449	0.00345	0.003394	0.003395	0.00275	0.002738	0.002931	0.00293	0.003115	0.003117
0.001113	0.005857	0.008936	0.008937	0.003434	0.003436	0.003402	0.003404	0.002712	0.002708	0.002943	0.002941	0.003118	0.00312
0.001124	0.007434	0.009028	0.009034	0.003426	0.003422	0.003426	0.003421	0.002639	0.002672	0.002966	0.002961	0.003135	0.003129
0.001152	0.01175	0.00929	0.009289	0.003416	0.003419	0.003474	0.003476	0.002632	0.002625	0.003019	0.003021	0.003163	0.003165
0.001166	0.013702	0.009404	0.009401	0.003429	0.003428	0.003504	0.003504	0.002622	0.002618	0.003046	0.00305	0.003185	0.003185

proton	30(I,III)	30(II,IV)	32a(I,III)	32a(II,IV)	32b	32b
K	734.4884401	790.8249907	564.7479561	714.2498081	465.459364	475.2223418
α	0.621979834	0.668005455	0.704725184	0.508307573	0.599901856	0.598519197
R² of the fit	0.999992645	0.999984169	0.999954847	0.999948178	0.999945983	0.999936077

[H] [M]	[G] [M]	observed	fitted	observed	fitted	observed	fitted	observed	fitted	observed	fitted	observed	fitted
0.001074	0	0.006297	0.006297	0.006451	0.006451	0.004345	0.004345	0.004263	0.004263	0.003836	0.003836	0.003831	0.003831
0.001075	0.000187	0.006305	0.006304	0.006459	0.006459	0.004352	0.004351	0.00427	0.00427	0.003842	0.003842	0.003838	0.003837
0.001076	0.00037	0.006311	0.006312	0.006466	0.006467	0.004357	0.004358	0.004276	0.004276	0.003848	0.003848	0.003843	0.003843
0.001078	0.00055	0.006319	0.006319	0.006475	0.006474	0.004365	0.004364	0.004283	0.004282	0.003855	0.003855	0.00385	0.00385
0.00108	0.000899	0.006333	0.006333	0.00649	0.00649	0.004376	0.004376	0.004295	0.004295	0.003866	0.003867	0.003861	0.003862
0.001082	0.001235	0.006347	0.006347	0.006506	0.006505	0.004389	0.004388	0.004308	0.004307	0.003879	0.003878	0.003874	0.003874
0.001084	0.001558	0.006359	0.00636	0.006519	0.00652	0.0044	0.0044	0.004318	0.004318	0.003889	0.003889	0.003885	0.003885
0.001086	0.00187	0.006373	0.006372	0.006534	0.006534	0.004412	0.004411	0.00433	0.00433	0.003901	0.0039	0.003896	0.003896
0.00109	0.002462	0.006397	0.006396	0.006561	0.006561	0.004433	0.004432	0.004351	0.004351	0.003921	0.00392	0.003916	0.003916
0.001099	0.003774	0.006449	0.00645	0.006619	0.006619	0.004477	0.004478	0.004396	0.004396	0.003964	0.003964	0.003959	0.003959
0.001107	0.004893	0.006494	0.006495	0.006667	0.006668	0.004515	0.004515	0.004433	0.004433	0.003999	0.003999	0.003995	0.003995
0.001113	0.005857	0.006533	0.006533	0.006709	0.00671	0.004546	0.004546	0.004464	0.004464	0.004029	0.004029	0.004024	0.004024
0.001124	0.007434	0.006596	0.006596	0.006775	0.006777	0.004593	0.004596	0.004511	0.004513	0.004073	0.004076	0.004068	0.004071
0.001152	0.01175	0.006768	0.006768	0.006957	0.006956	0.004726	0.004725	0.004642	0.004641	0.004197	0.004197	0.004193	0.004192
0.001166	0.013702	0.006846	0.006845	0.007038	0.007037	0.004783	0.004782	0.004699	0.004698	0.00425	0.004249	0.004245	0.004244

Table S27: NMR titration data and fit for **(+)-Zr(1)$_2$** with **G3S**. Data is given as the product of the chemical shift and the host concentration. Duplo experiment.

proton		3,4,13,14		27a(I,III)		27a(II,IV)		27b(I,III)		27b(II,IV)		28a(I,III)	
K		642.3277109		511.0686857		528.0501059		387.7976876		419.1532195		487.8773	
α		0.608571434		0.531363839		0.463491345		0.575931478		0.419481625		0.46745	
R^2 of the fit		0.999978504		0.99991977		0.999993558		0.999910723		0.999970376		0.9999906	
[H] [M]	[G] [M]	observed	fitted	observed	fitted	observed	fitted	observed	fitted	observed	fitted	observed	fitted
0.000789	0	0.006282	0.006282	0.002639	0.002639	0.00248	0.00248	0.00219	0.00219	0.002105	0.002105	0.002298	0.002298
0.000788	0.000176	0.006272	0.006272	0.002624	0.002623	0.002472	0.002471	0.002172	0.002171	0.002099	0.002099	0.002289	0.002289
0.000786	0.000349	0.006263	0.006262	0.002608	0.002607	0.002463	0.002463	0.002155	0.002154	0.002093	0.002093	0.00228	0.00228
0.000785	0.000518	0.006254	0.006253	0.002592	0.002592	0.002455	0.002455	0.002137	0.002137	0.002088	0.002088	0.002272	0.002272
0.000783	0.000684	0.006245	0.006244	0.002577	0.002578	0.002448	0.002448	0.002119	0.00212	0.002082	0.002083	0.002263	0.002263
0.000781	0.001007	0.006226	0.006226	0.002552	0.00255	0.002433	0.002433	0.002094	0.00209	0.002073	0.002072	0.002249	0.002248
0.000778	0.001318	0.00621	0.006209	0.002524	0.002525	0.002419	0.00242	0.00206	0.002063	0.002062	0.002063	0.002233	0.002233
0.000775	0.001617	0.006193	0.006192	0.0025	0.002502	0.002406	0.002407	0.002035	0.002037	0.002053	0.002054	0.002219	0.00222
0.00077	0.002185	0.00616	0.00616	0.002461	0.00246	0.002383	0.002383	0.001992	0.001992	0.002037	0.002037	0.002195	0.002195
0.00076	0.003443	0.006085	0.006086	0.002378	0.002378	0.002335	0.002335	0.001905	0.001905	0.002002	0.002001	0.002144	0.002144
0.00075	0.004514	0.006019	0.006021	0.002319	0.002318	0.002297	0.002297	0.00184	0.001842	0.001972	0.001972	0.002104	0.002104
0.000742	0.005436	0.005961	0.005963	0.002272	0.00227	0.002265	0.002265	0.001798	0.001793	0.001948	0.001948	0.002072	0.002072
0.000729	0.006942	0.005865	0.005866	0.002201	0.002201	0.002216	0.002216	0.001722	0.001723	0.001909	0.001909	0.002022	0.002023
0.000708	0.009477	0.005699	0.005699	0.002095	0.0021	0.002139	0.002138	0.001624	0.001624	0.001847	0.001846	0.001946	0.001946
0.000685	0.012124	0.005523	0.005521	0.00201	0.002007	0.002061	0.002061	0.001538	0.001537	0.001781	0.001782	0.00187	0.001871

proton		30(I,III)		30(II,IV)		32a(I,III)		32a(II,IV)		32b		32b	
K		636.4316319		674.5011986		639.3322271		662.1091462		634.7375037		475.8918	
α		0.525480064		0.657721813		0.653023607		0.735906761		0.558380712		0.727004	
R^2 of the fit		0.999998726		0.999996372		0.999988592		0.999964298		0.999986187		0.9999856	
[H] [M]	[G] [M]	observed	fitted	observed	fitted	observed	fitted	observed	fitted	observed	fitted	observed	fitted
0.000789	0	0.004626	0.004626	0.00474	0.00474	0.003193	0.003193	0.003133	0.003133	0.002819	0.002819	0.002815	0.002815
0.000788	0.000176	0.004618	0.004618	0.004732	0.004732	0.003188	0.003188	0.003128	0.003128	0.002815	0.002815	0.002811	0.002811
0.000786	0.000349	0.004609	0.004609	0.004724	0.004724	0.003184	0.003184	0.003124	0.003124	0.002812	0.002812	0.002808	0.002808
0.000785	0.000518	0.004601	0.004601	0.004716	0.004716	0.00318	0.00318	0.00312	0.00312	0.002809	0.002808	0.002805	0.002805
0.000783	0.000684	0.004593	0.004593	0.004709	0.004708	0.003176	0.003175	0.003116	0.003116	0.002806	0.002805	0.002802	0.002802
0.000781	0.001007	0.004577	0.004577	0.004693	0.004693	0.003167	0.003167	0.003108	0.003107	0.002799	0.002799	0.002795	0.002795
0.000778	0.001318	0.004562	0.004562	0.004679	0.004678	0.003159	0.003159	0.0031	0.0031	0.002793	0.002792	0.002789	0.002789
0.000775	0.001617	0.004547	0.004547	0.004664	0.004664	0.003151	0.00315	0.003092	0.003092	0.002786	0.002786	0.002783	0.002782
0.00077	0.002185	0.004519	0.004519	0.004637	0.004637	0.003134	0.003135	0.003077	0.003077	0.002773	0.002773	0.00277	0.00277
0.00076	0.003443	0.004456	0.004457	0.004576	0.004576	0.003097	0.003098	0.003041	0.003041	0.002743	0.002743	0.00274	0.00274
0.00075	0.004514	0.004403	0.004403	0.004523	0.004524	0.003064	0.003065	0.003008	0.003009	0.002715	0.002716	0.002712	0.002713
0.000742	0.005436	0.004357	0.004357	0.004477	0.004478	0.003035	0.003036	0.00298	0.002981	0.002691	0.002691	0.002688	0.002688
0.000729	0.006942	0.004282	0.004282	0.004402	0.004402	0.002987	0.002987	0.002933	0.002933	0.002649	0.002649	0.002647	0.002647
0.000708	0.009477	0.004155	0.004155	0.004273	0.004273	0.002902	0.002902	0.00285	0.00285	0.002576	0.002576	0.002574	0.002574
0.000685	0.012124	0.004022	0.004022	0.004137	0.004137	0.002812	0.002811	0.002762	0.002761	0.002498	0.002497	0.002496	0.002495

Table S28: NMR titration data and fit for **(–)-Zr(1)$_2$** with **G3R**. Data is given as the product of the chemical shift and the host concentration.

proton		3,4,13,14		27a(I,III)		27a(II,IV)		27b(I,III)		27b(II,IV)		28a(I,III)	
K		630.4267308		576.3631958		533.8253701		522.1630415		514.9251801		567.5416307	
α		0.534293134		0.511663154		0.460858869		0.516863366		0.447343909		0.476390462	
R^2 of the fit		0.982850646		0.99977769		0.999735977		0.998394715		0.998912318		0.999047532	
[H] [M]	[G] [M]	observed	fitted	observed	fitted	observed	fitted	observed	fitted	observed	fitted	observed	fitted
0.000735	0	0.005852	0.005852	0.002457	0.002457	0.002308	0.002308	0.002038	0.002038	0.001973	0.001973	0.00214	0.00214
0.000735	0.000157	0.005853	0.005853	0.002447	0.002448	0.002305	0.002305	0.002027	0.002027	0.001971	0.001971	0.002136	0.002136
0.000735	0.000311	0.005854	0.005854	0.002438	0.002439	0.002302	0.002302	0.002015	0.002016	0.001969	0.001969	0.002132	0.002132
0.000735	0.000461	0.005855	0.005855	0.002431	0.00243	0.002299	0.002299	0.002007	0.002005	0.001968	0.001968	0.002129	0.002128
0.000735	0.000754	0.005857	0.005857	0.002414	0.002414	0.002292	0.002293	0.001986	0.001985	0.001964	0.001964	0.002121	0.002121
0.000734	0.001036	0.005859	0.005858	0.0024	0.002399	0.002288	0.002287	0.001967	0.001967	0.001961	0.001961	0.002115	0.002115
0.000734	0.001308	0.00586	0.00586	0.002385	0.002386	0.002282	0.002282	0.00195	0.00195	0.001958	0.001959	0.002109	0.002108
0.000734	0.00157	0.005861	0.005861	0.002372	0.002373	0.002277	0.002278	0.001934	0.001934	0.001956	0.001956	0.002103	0.002103
0.000734	0.002066	0.005864	0.005864	0.00235	0.00235	0.00227	0.00227	0.001906	0.001906	0.001952	0.001952	0.002092	0.002092
0.000733	0.003168	0.005867	0.005867	0.002307	0.002307	0.002254	0.002254	0.001852	0.001852	0.001943	0.001943	0.002073	0.002072
0.000732	0.004107	0.005868	0.005868	0.002276	0.002276	0.002241	0.002242	0.001812	0.001814	0.001937	0.001937	0.002058	0.002058
0.000731	0.004916	0.005868	0.005869	0.002254	0.002253	0.002233	0.002233	0.001785	0.001785	0.001931	0.001932	0.002047	0.002047
0.00073	0.006241	0.005867	0.005867	0.002221	0.002222	0.002221	0.00222	0.001743	0.001746	0.001925	0.001924	0.002031	0.002032
0.000727	0.009864	0.005855	0.005857	0.002166	0.002162	0.002191	0.002193	0.001686	0.00167	0.00191	0.001908	0.002005	0.002
0.000726	0.011501	0.005853	0.005851	0.002139	0.002142	0.002184	0.002183	0.001634	0.001645	0.001899	0.001901	0.001986	0.001989

proton		30(I,III)		30(II,IV)		32a(I,III)		32a(II,IV)		32b		32b	
K		742.480942		636.5996848		657.0506854		755.0925952		651.0774744		641.3942325	

α		0.736737323		0.693962653		0.563605343		0.474513281		0.535000659		0.542459855	
R^2 of the fit		0.999713776		0.998847081		0.993311121		0.995466271		0.99810476		0.996492396	
[H] [M]	[G] [M]	observed	fitted	observed	fitted	observed	fitted	observed	fitted	observed	fitted	observed	fitted
0.000735	0	0.004311	0.004311	0.004416	0.004416	0.002974	0.002974	0.002918	0.002918	0.002626	0.002626	0.002622	0.002622
0.000735	0.000157	0.004311	0.00431	0.004416	0.004416	0.002975	0.002975	0.002919	0.002919	0.002627	0.002627	0.002624	0.002623
0.000735	0.000311	0.00431	0.00431	0.004416	0.004416	0.002976	0.002976	0.00292	0.00292	0.002628	0.002628	0.002625	0.002625
0.000735	0.000461	0.004309	0.004309	0.004416	0.004415	0.002976	0.002976	0.002921	0.002921	0.002629	0.002629	0.002626	0.002626
0.000735	0.000754	0.004308	0.004308	0.004416	0.004415	0.002978	0.002978	0.002923	0.002922	0.002631	0.002631	0.002628	0.002628
0.000734	0.001036	0.004307	0.004307	0.004415	0.004415	0.002979	0.002979	0.002924	0.002924	0.002633	0.002633	0.00263	0.00263
0.000734	0.001308	0.004306	0.004306	0.004415	0.004415	0.00298	0.00298	0.002925	0.002925	0.002635	0.002635	0.002632	0.002632
0.000734	0.00157	0.004306	0.004305	0.004415	0.004415	0.002981	0.002981	0.002926	0.002926	0.002636	0.002636	0.002633	0.002633
0.000734	0.002066	0.004304	0.004304	0.004414	0.004414	0.002983	0.002983	0.002928	0.002928	0.002639	0.002639	0.002636	0.002636
0.000733	0.003168	0.0043	0.0043	0.004413	0.004413	0.002986	0.002986	0.002931	0.002931	0.002644	0.002644	0.002641	0.002641
0.000732	0.004107	0.004296	0.004296	0.00441	0.004411	0.002987	0.002987	0.002933	0.002933	0.002646	0.002646	0.002644	0.002644
0.000731	0.004916	0.004293	0.004293	0.004408	0.004409	0.002987	0.002988	0.002933	0.002933	0.002648	0.002648	0.002645	0.002645
0.00073	0.006241	0.004287	0.004287	0.004405	0.004405	0.002988	0.002987	0.002934	0.002934	0.002649	0.002649	0.002647	0.002647
0.000727	0.009864	0.004272	0.004272	0.004391	0.004391	0.002982	0.002983	0.002929	0.00293	0.002647	0.002648	0.002644	0.002645
0.000726	0.011501	0.004265	0.004264	0.004386	0.004385	0.002981	0.00298	0.002928	0.002927	0.002647	0.002646	0.002645	0.002644

Table S29: NMR titration data and fit for **(–)-Zr(1)$_2$** with **G3R**. Data is given as the product of the chemical shift and the host concentration. Duplo experiment.

proton		3,4,13,14		27a(I,III)		27a(II,IV)		27b(I,III)		27b(II,IV)		28a(I,III)	
K		754.7429745		640.8955291		585.9199878		546.8128819		445.2094626		529.0390605	
α		0.656416352		0.511231375		0.49486341		0.552726944		0.40991645		0.511944978	
R^2 of the fit		0.999959534		0.999969077		0.999986279		0.999929923		0.999988372		0.999989352	
[H] [M]	[G] [M]	observed	fitted	observed	fitted	observed	fitted	observed	fitted	observed	fitted	observed	fitted
0.000598	0	0.004761	0.004761	0.002001	0.002001	0.00188	0.00188	0.00166	0.00166	0.001608	0.001608	0.001742	0.001742
0.000597	0.000136	0.004756	0.004755	0.001991	0.001991	0.001874	0.001875	0.001649	0.00165	0.001604	0.001604	0.001737	0.001737
0.000596	0.000269	0.00475	0.004749	0.001983	0.001982	0.00187	0.00187	0.00164	0.001639	0.001601	0.001601	0.001732	0.001732
0.000596	0.000399	0.004744	0.004743	0.001972	0.001972	0.001865	0.001865	0.001628	0.001629	0.001597	0.001597	0.001726	0.001726
0.000594	0.000652	0.004733	0.004732	0.001954	0.001955	0.001855	0.001855	0.001609	0.001609	0.00159	0.001591	0.001716	0.001716
0.000592	0.000896	0.004723	0.004722	0.001937	0.001938	0.001846	0.001846	0.00159	0.001591	0.001584	0.001584	0.001707	0.001707
0.000591	0.001131	0.004712	0.004712	0.001924	0.001923	0.001839	0.001838	0.001575	0.001574	0.001579	0.001578	0.001698	0.001698
0.000589	0.001357	0.004702	0.004702	0.001907	0.001908	0.00183	0.00183	0.001557	0.001557	0.001572	0.001573	0.001689	0.001689
0.000586	0.001786	0.004683	0.004683	0.001882	0.001881	0.001815	0.001816	0.001529	0.001528	0.001562	0.001562	0.001674	0.001674
0.000579	0.002739	0.004639	0.00464	0.001829	0.001828	0.001785	0.001785	0.00147	0.00147	0.00154	0.00154	0.001641	0.001641
0.000574	0.003551	0.0046	0.004601	0.001789	0.001788	0.00176	0.00176	0.001427	0.001427	0.001522	0.001521	0.001616	0.001616
0.000569	0.004251	0.004566	0.004567	0.001757	0.001757	0.00174	0.00174	0.001394	0.001393	0.001506	0.001506	0.001595	0.001595
0.000561	0.005396	0.004509	0.00451	0.001709	0.001711	0.001709	0.001709	0.001342	0.001345	0.001481	0.001481	0.001562	0.001563
0.000548	0.007326	0.00441	0.00441	0.001643	0.001643	0.001659	0.001659	0.001279	0.001277	0.001441	0.001441	0.001513	0.001513
0.000534	0.009348	0.004305	0.004303	0.001582	0.001582	0.00161	0.00161	0.001217	0.001218	0.001401	0.001401	0.001465	0.001465

proton		30(I,III)		30(II,IV)		32a(I,III)		32a(II,IV)		32b		32b	
K		844.0850269		776.9919963		696.2292463		659.3042704		538.7880336		485.5921329	
α		0.536093596		0.637183182		0.647408256		0.715365552		0.731000979		0.724508941	
R^2 of the fit		0.999997374		0.999995531		0.999986647		0.999975647		0.999983394		0.999986974	
[H] [M]	[G] [M]	observed	fitted	observed	fitted	observed	fitted	observed	fitted	observed	fitted	observed	fitted
0.000598	0	0.003507	0.003507	0.003593	0.003593	0.00242	0.00242	0.002375	0.002375	0.002136	0.002136	0.002134	0.002134
0.000597	0.000136	0.003502	0.003502	0.003588	0.003588	0.002418	0.002417	0.002372	0.002372	0.002134	0.002134	0.002132	0.002131
0.000596	0.000269	0.003496	0.003496	0.003583	0.003583	0.002415	0.002415	0.002369	0.002369	0.002132	0.002132	0.002129	0.002129
0.000596	0.000399	0.003492	0.003491	0.003579	0.003578	0.002412	0.002412	0.002367	0.002367	0.00213	0.00213	0.002128	0.002127
0.000594	0.000652	0.003482	0.003481	0.003569	0.003569	0.002407	0.002407	0.002362	0.002362	0.002127	0.002126	0.002124	0.002124
0.000592	0.000896	0.003472	0.003472	0.00356	0.00356	0.002402	0.002402	0.002357	0.002357	0.002123	0.002122	0.00212	0.00212
0.000591	0.001131	0.003463	0.003463	0.003551	0.003551	0.002397	0.002397	0.002352	0.002352	0.002118	0.002119	0.002116	0.002116
0.000589	0.001357	0.003454	0.003454	0.003543	0.003542	0.002392	0.002392	0.002348	0.002348	0.002115	0.002115	0.002113	0.002112
0.000586	0.001786	0.003437	0.003437	0.003526	0.003526	0.002383	0.002383	0.002338	0.002339	0.002107	0.002108	0.002105	0.002105
0.000579	0.002739	0.0034	0.0034	0.00349	0.00349	0.002361	0.002361	0.002318	0.002318	0.00209	0.00209	0.002088	0.002088
0.000574	0.003551	0.003368	0.003368	0.003458	0.003459	0.002342	0.002342	0.002299	0.002299	0.002074	0.002075	0.002072	0.002072
0.000569	0.004251	0.00334	0.00334	0.003431	0.003431	0.002325	0.002325	0.002282	0.002283	0.00206	0.00206	0.002058	0.002058
0.000561	0.005396	0.003295	0.003295	0.003386	0.003386	0.002296	0.002296	0.002255	0.002255	0.002036	0.002036	0.002034	0.002034
0.000548	0.007326	0.003218	0.003218	0.003308	0.003308	0.002246	0.002246	0.002206	0.002205	0.001993	0.001993	0.001991	0.001991
0.000534	0.009348	0.003138	0.003138	0.003227	0.003226	0.002192	0.002192	0.002153	0.002152	0.001946	0.001946	0.001945	0.001945

Table S30: NMR titration data and fit for **(–)-Zr(1)$_2$** with **G3S**. Data is given as the product of the chemical shift and the host concentration.

proton		3,4,13,14		27a(I,III)		27a(II,IV)		27b(I,III)		27b(II,IV)		28a(I,III)	
K		497.0175569		469.2898393		406.8358836		455.2114778		426.0573095		413.871173	
α		0.554582997		0.465482843		0.406807077		0.460910568		0.450990012		0.450380941	
R^2 of the fit		0.99993459		0.998478137		0.999921409		0.995772155		0.999168223		0.994503489	
[H] [M]	[G] [M]	observed	fitted	observed	fitted	observed	fitted	observed	fitted	observed	fitted	observed	fitted
0.000656	0	0.005224	0.005224	0.002192	0.002192	0.00206	0.00206	0.001819	0.001819	0.001761	0.001761	0.001909	0.001909
0.000656	0.000161	0.005228	0.005229	0.002191	0.002191	0.002057	0.002056	0.001818	0.001818	0.001755	0.001755	0.001909	0.001909
0.000657	0.000319	0.005235	0.005234	0.00219	0.00219	0.002052	0.002052	0.001818	0.001818	0.001748	0.001749	0.001908	0.001908
0.000657	0.000473	0.005239	0.005239	0.00219	0.002189	0.002047	0.002048	0.001817	0.001817	0.001743	0.001743	0.001908	0.001907
0.000658	0.000774	0.005249	0.005249	0.002187	0.002188	0.00204	0.00204	0.001816	0.001817	0.001732	0.001732	0.001906	0.001906
0.000659	0.001063	0.005259	0.005258	0.002186	0.002186	0.002033	0.002033	0.001816	0.001816	0.001722	0.001722	0.001905	0.001905
0.00066	0.001342	0.005268	0.005267	0.002184	0.002184	0.002027	0.002026	0.001815	0.001815	0.001711	0.001711	0.001904	0.001904
0.000661	0.001611	0.005276	0.005276	0.002183	0.002183	0.00202	0.00202	0.001815	0.001815	0.001701	0.001702	0.001903	0.001903
0.000662	0.00212	0.005293	0.005293	0.002181	0.002181	0.002009	0.002009	0.001813	0.001814	0.001685	0.001684	0.001902	0.001902
0.000666	0.003251	0.005328	0.005329	0.002178	0.002178	0.001987	0.001987	0.001813	0.001813	0.001652	0.00165	0.0019	0.0019
0.000669	0.004214	0.005357	0.005358	0.002177	0.002177	0.001971	0.001971	0.001814	0.001813	0.001626	0.001624	0.001901	0.0019
0.000671	0.005045	0.005383	0.005383	0.002177	0.002177	0.001959	0.001959	0.001814	0.001814	0.001599	0.001605	0.0019	0.001901
0.000675	0.006403	0.005422	0.005423	0.00218	0.002179	0.001944	0.001944	0.001817	0.001817	0.001583	0.00158	0.001904	0.001903
0.000686	0.010121	0.005526	0.005525	0.002192	0.002193	0.001918	0.001918	0.001831	0.001831	0.001531	0.001532	0.001915	0.001915

proton		30(I,III)		30(II,IV)		32a(I,III)		32a(II,IV)		32b		32b	

K		549.6210929		570.8126162		656.7174904		497.036848		515.972998		526.7530117	
α		0.776954033		0.544636607		0.540138869		0.577962399		0.506814454		0.51703026	
R^2 of the fit		0.999883859		0.999995863		0.999923843		0.99996188		0.999933125		0.999958828	
[H] [M]	[G] [M]	observed	fitted	observed	fitted	observed	fitted	observed	fitted	observed	fitted	observed	fitted
0.000656	0	0.003847	0.003847	0.00394	0.00394	0.002654	0.002654	0.002604	0.002604	0.002343	0.002343	0.00234	0.00234
0.000656	0.000161	0.003851	0.00385	0.003943	0.003943	0.002657	0.002657	0.002607	0.002607	0.002346	0.002346	0.002343	0.002343
0.000657	0.000319	0.003854	0.003854	0.003946	0.003946	0.00266	0.002659	0.00261	0.00261	0.002349	0.002349	0.002346	0.002346
0.000657	0.000473	0.003858	0.003857	0.003948	0.003948	0.002662	0.002662	0.002613	0.002613	0.002352	0.002351	0.002349	0.002348
0.000658	0.000774	0.003865	0.003864	0.003953	0.003953	0.002667	0.002667	0.002619	0.002618	0.002357	0.002357	0.002354	0.002354
0.000659	0.001063	0.003871	0.003871	0.003958	0.003958	0.002673	0.002672	0.002624	0.002624	0.002363	0.002362	0.002359	0.002359
0.00066	0.001342	0.003878	0.003877	0.003963	0.003963	0.002677	0.002677	0.002629	0.002629	0.002368	0.002367	0.002365	0.002364
0.000661	0.001611	0.003884	0.003884	0.003967	0.003967	0.002682	0.002682	0.002634	0.002634	0.002372	0.002372	0.00237	0.002369
0.000662	0.00212	0.003895	0.003895	0.003976	0.003976	0.002691	0.002691	0.002643	0.002643	0.002381	0.002381	0.002379	0.002378
0.000666	0.003251	0.00392	0.003921	0.003995	0.003995	0.002709	0.00271	0.002663	0.002663	0.0024	0.0024	0.002397	0.002398
0.000669	0.004214	0.003941	0.003941	0.004011	0.004011	0.002724	0.002725	0.002679	0.002679	0.002415	0.002416	0.002413	0.002413
0.000671	0.005045	0.003958	0.003959	0.004026	0.004025	0.002737	0.002737	0.002692	0.002692	0.002428	0.002429	0.002426	0.002426
0.000675	0.006403	0.003986	0.003986	0.004049	0.004049	0.002757	0.002757	0.002713	0.002713	0.002448	0.002449	0.002446	0.002446
0.000686	0.010121	0.00406	0.004058	0.004114	0.004114	0.00281	0.002809	0.002767	0.002767	0.0025	0.002499	0.002497	0.002497

Table S31: NMR titration data and fit for **(–)-Zr(1)$_2$** with **G3S**. Data is given as the product of the chemical shift and the host concentration. Duplo experiment.

proton		3,4,13,14		27a(I,III)		27a(II,IV)		27b(I,III)		27b(II,IV)		28a(I,III)	
K		682.6226		691.1509		513.1727		622.3729		647.1982		653.7975	
α		0.668555		0.490096		0.536598		0.482746		0.395927		0.492554	
R^2 of the fit		0.9999627		0.999993		0.999922		0.9999889		0.9999601		0.9999904	
[H] [M]	[G] [M]	observed	fitted	observed	fitted	observed	fitted	observed	fitted	observed	fitted	observed	fitted
0.000573	0	0.00456	0.00456	0.001917	0.001917	0.001801	0.001801	0.00159	0.00159	0.001529	0.001529	0.001657	0.001657
0.000572	0.000231	0.004552	0.004552	0.001907	0.001907	0.001787	0.001786	0.001583	0.001583	0.001513	0.001512	0.00165	0.001649
0.000571	0.000343	0.004548	0.004547	0.001903	0.001903	0.00178	0.001779	0.00158	0.00158	0.001505	0.001504	0.001646	0.001646
0.00057	0.000453	0.004544	0.004543	0.001899	0.001899	0.001773	0.001773	0.001577	0.001577	0.001497	0.001496	0.001642	0.001642
0.00057	0.000561	0.00454	0.004539	0.001894	0.001894	0.001766	0.001766	0.001573	0.001573	0.001489	0.001489	0.001639	0.001639
0.000568	0.000771	0.004532	0.004531	0.001886	0.001886	0.001754	0.001754	0.001567	0.001567	0.001475	0.001475	0.001632	0.001632
0.000567	0.000973	0.004524	0.004524	0.001879	0.001879	0.001742	0.001743	0.001561	0.001562	0.001461	0.001462	0.001626	0.001626
0.000566	0.001168	0.004517	0.004517	0.001871	0.001872	0.001732	0.001732	0.001556	0.001556	0.001449	0.001449	0.00162	0.00162
0.000564	0.001538	0.004503	0.004503	0.001858	0.001859	0.001711	0.001712	0.001546	0.001546	0.001426	0.001427	0.001608	0.001609
0.000559	0.002358	0.00447	0.004471	0.001832	0.001832	0.001672	0.001673	0.001526	0.001526	0.001382	0.001382	0.001586	0.001586
0.000555	0.003056	0.004442	0.004443	0.00181	0.00181	0.001642	0.001642	0.001509	0.001509	0.001348	0.001348	0.001568	0.001568
0.000551	0.003658	0.004417	0.004418	0.001793	0.001793	0.001621	0.001618	0.001496	0.001496	0.001322	0.001321	0.001553	0.001553
0.000545	0.004644	0.004375	0.004376	0.001766	0.001766	0.001581	0.001582	0.001474	0.001474	0.001283	0.001282	0.00153	0.00153
0.000535	0.006305	0.004302	0.004302	0.001724	0.001724	0.001528	0.001529	0.001441	0.001441	0.001225	0.001226	0.001494	0.001494
0.000525	0.008046	0.004224	0.004223	0.001684	0.001684	0.001481	0.00148	0.001408	0.001408	0.001176	0.001176	0.001459	0.001459

proton		30(I,III)		30(II,IV)		32a(I,III)		32a(II,IV)		32b		32b	
K		765.87		806.4178		807.2963		722.7164		656.3981		668.5846	
α		0.633717		0.573735		0.601839		0.67676		0.759725		0.762614	
R^2 of the fit		0.9999828		0.9999996		0.9999766		0.999953		0.9998951		0.9998986	
[H] [M]	[G] [M]	observed	fitted	observed	fitted	observed	fitted	observed	fitted	observed	fitted	observed	fitted
0.000573	0	0.003359	0.003359	0.003442	0.003442	0.002318	0.002318	0.002274	0.002274	0.002046	0.002046	0.002044	0.002044
0.000572	0.000231	0.003352	0.003352	0.003433	0.003433	0.002314	0.002314	0.002271	0.002271	0.002043	0.002043	0.002041	0.002041
0.000571	0.000343	0.003349	0.003349	0.003429	0.003429	0.002312	0.002312	0.002269	0.002269	0.002042	0.002042	0.002039	0.002039
0.00057	0.000453	0.003346	0.003346	0.003425	0.003425	0.00231	0.00231	0.002267	0.002267	0.002041	0.002041	0.002038	0.002038
0.00057	0.000561	0.003343	0.003343	0.003421	0.003421	0.002308	0.002308	0.002266	0.002265	0.00204	0.002039	0.002037	0.002036
0.000568	0.000771	0.003337	0.003337	0.003413	0.003413	0.002304	0.002304	0.002262	0.002262	0.002037	0.002036	0.002034	0.002034
0.000567	0.000973	0.003331	0.003331	0.003405	0.003405	0.002301	0.0023	0.002259	0.002259	0.002034	0.002034	0.002031	0.002031
0.000566	0.001168	0.003325	0.003325	0.003398	0.003398	0.002297	0.002297	0.002256	0.002256	0.002032	0.002031	0.002029	0.002029
0.000564	0.001538	0.003315	0.003314	0.003384	0.003384	0.00229	0.00229	0.002249	0.002249	0.002027	0.002026	0.002024	0.002024
0.000559	0.002358	0.003289	0.00329	0.003354	0.003354	0.002274	0.002274	0.002234	0.002235	0.002014	0.002014	0.002011	0.002012
0.000555	0.003056	0.003268	0.003268	0.003328	0.003328	0.002259	0.00226	0.002221	0.002221	0.002003	0.002003	0.002	0.002001
0.000551	0.003658	0.003249	0.003249	0.003306	0.003306	0.002246	0.002247	0.002209	0.00221	0.001992	0.001993	0.00199	0.001991
0.000545	0.004644	0.003217	0.003217	0.00327	0.00327	0.002225	0.002225	0.002189	0.002189	0.001975	0.001975	0.001973	0.001973
0.000535	0.006305	0.003162	0.003162	0.00321	0.00321	0.002188	0.002188	0.002153	0.002153	0.001944	0.001944	0.001942	0.001942
0.000525	0.008046	0.003104	0.003103	0.003147	0.003147	0.002148	0.002148	0.002115	0.002114	0.00191	0.001909	0.001908	0.001907

Table S32: Calculated CIS values of proton signals of the complexes of **(+)-Zr(1)$_2$** and **(–)-Zr(1)$_2$** with **G3R** and **G3S** [ppm]

Proton	δ	(+)-Zr(1)$_2$		(–)-Zr(1)$_2$	
		**G3R**	**G3S**	**G3R**	**G3S**
**3,4,13,14**	7.97	0.14	0.15	0.14	0.14
**27a(I, III)**	3.34	–0.30	–0.63	–0.60	–0.25
**27a(II, IV)**	3.14	–0.72	–0.22	–0.21	–0.65
**27b(I, III)**	2.77	–0.21	–0.81	–0.79	–0.18
**27b(II, IV)**	2.67	–0.79	–0.11	–0.12	–0.84
**28a(I, III)**	2.91	–0.24	–0.31	–0.28	–0.21
**30(II, IV)**	6.01	–0.01	0.04	0.05	–0.02
**30(I, III)**	5.87	0.08	0.01	0.01	0.07
**32a(I, III)**	4.04	0.08	0.08	0.08	0.07
**32a(II, IV)**	3.99	0.10	0.08	0.09	0.09
**32b**	3.56	0.12	0.11	0.11	0.11
**32b**	3.56	0.12	0.11	0.11	0.11

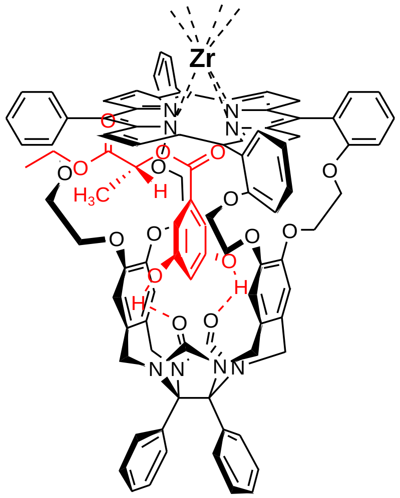

Figure S14. Proposed binding geometry of **G3R** inside one of the cavities of **Zr(1)₂;** for clarity only one of the porphyrin cages connected to the Zr center is shown.

NMR Spectral data

(*R*)-5

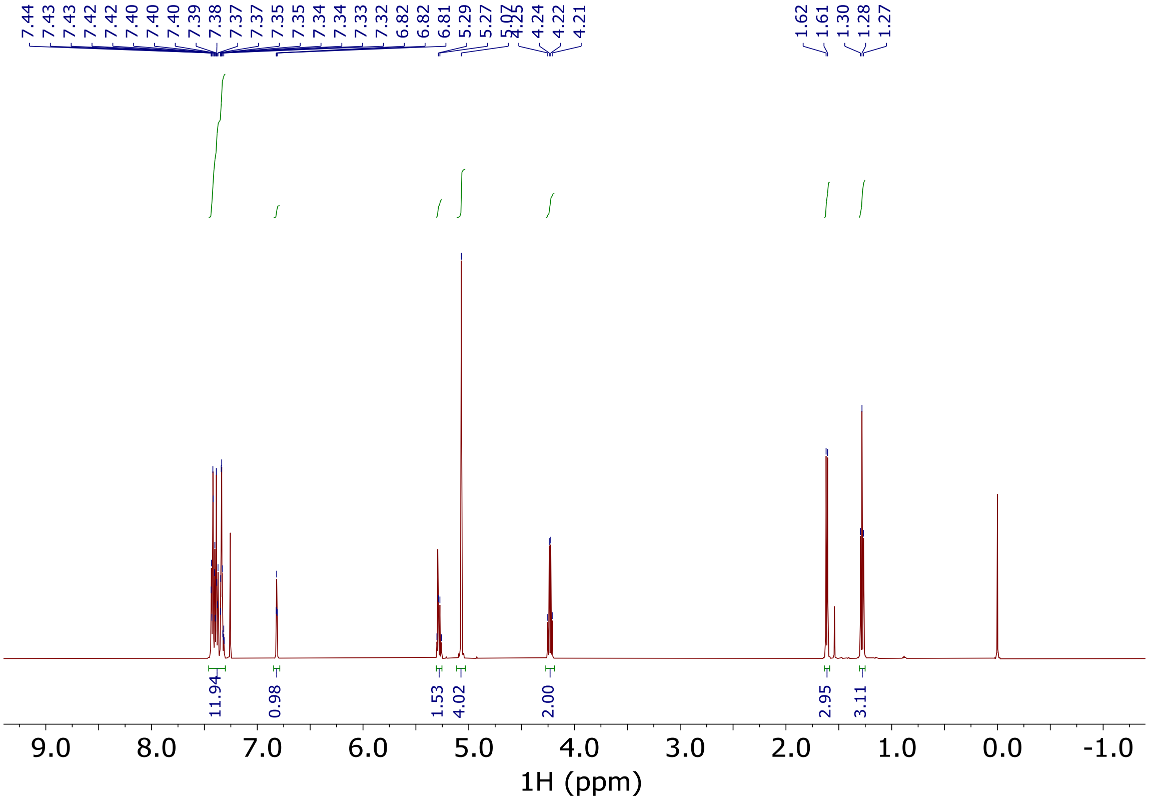

Figure S15. ^{1}H NMR spectrum (500 MHz, $CDCl_3$, 298K) of (*R*)-**5**.

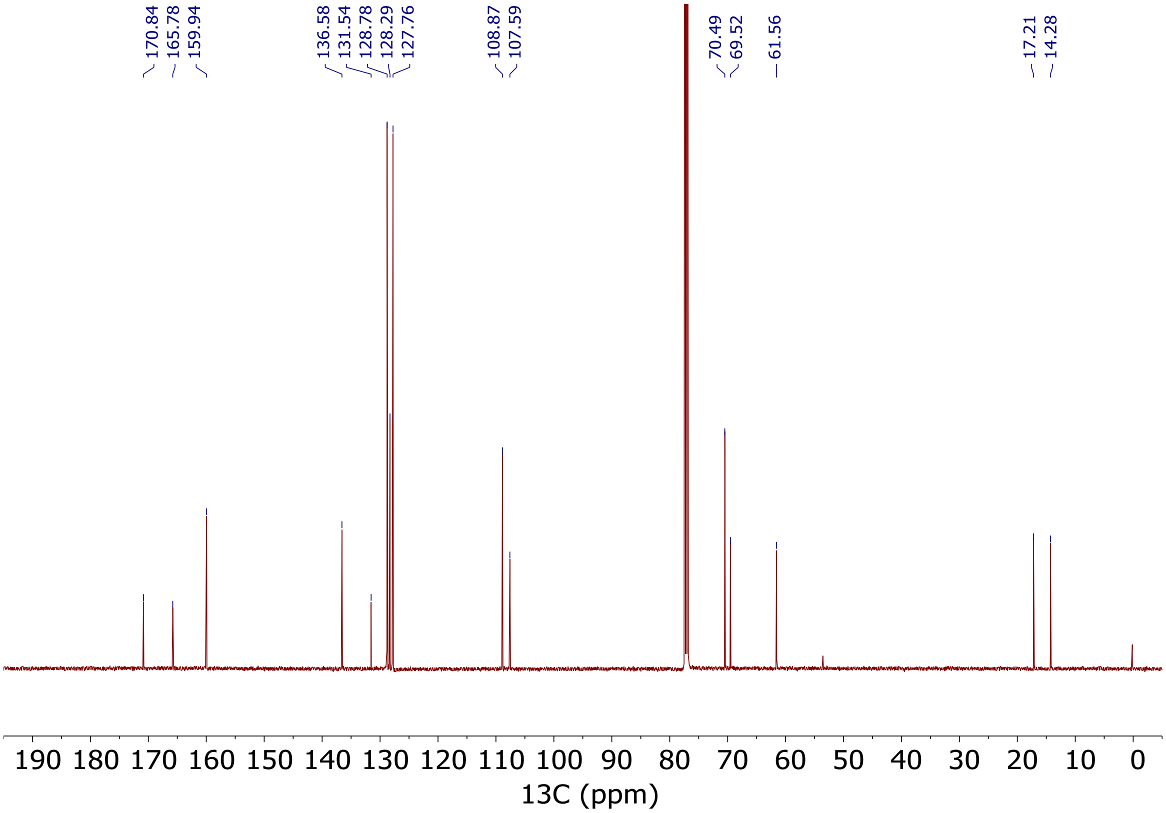

Figure S16. ^{13}C NMR spectrum (126 MHz, $CDCl_3$, 298K) of (*R*)-**5**.

(*S*)-5

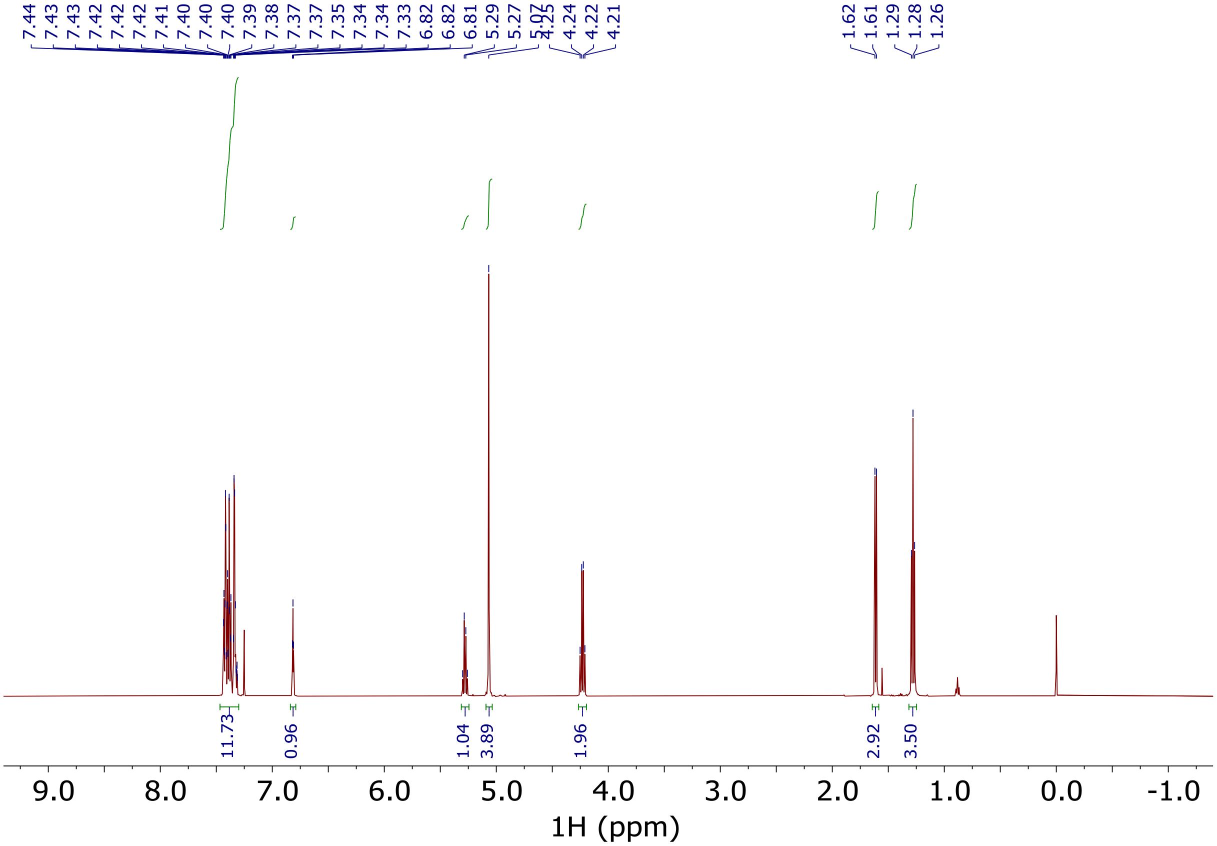

Figure S17. ^{1}H NMR spectrum (500 MHz, $CDCl_3$, 298K) of (*S*)-**5**.

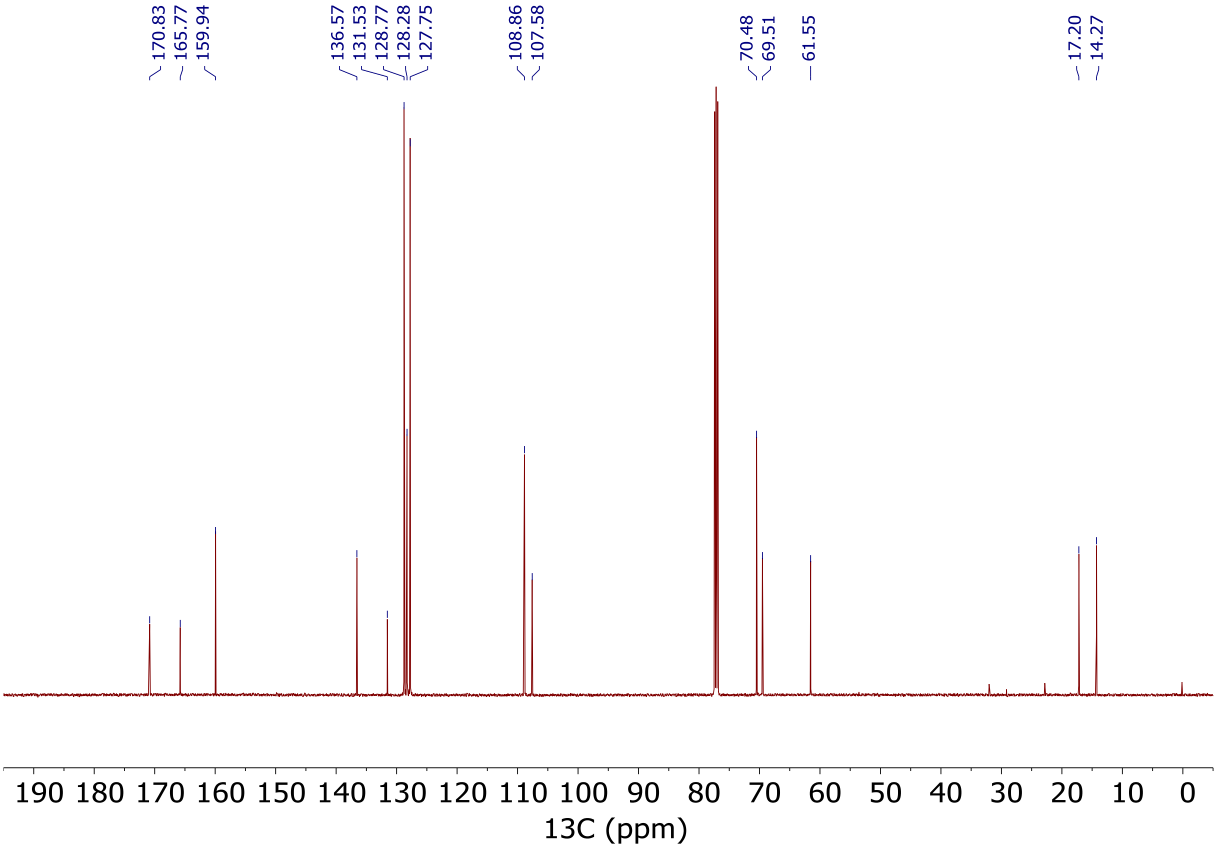

Figure S18. ^{13}C NMR spectrum (126 MHz, $CDCl_3$, 298K) of (*S*)-**5**.

G3R

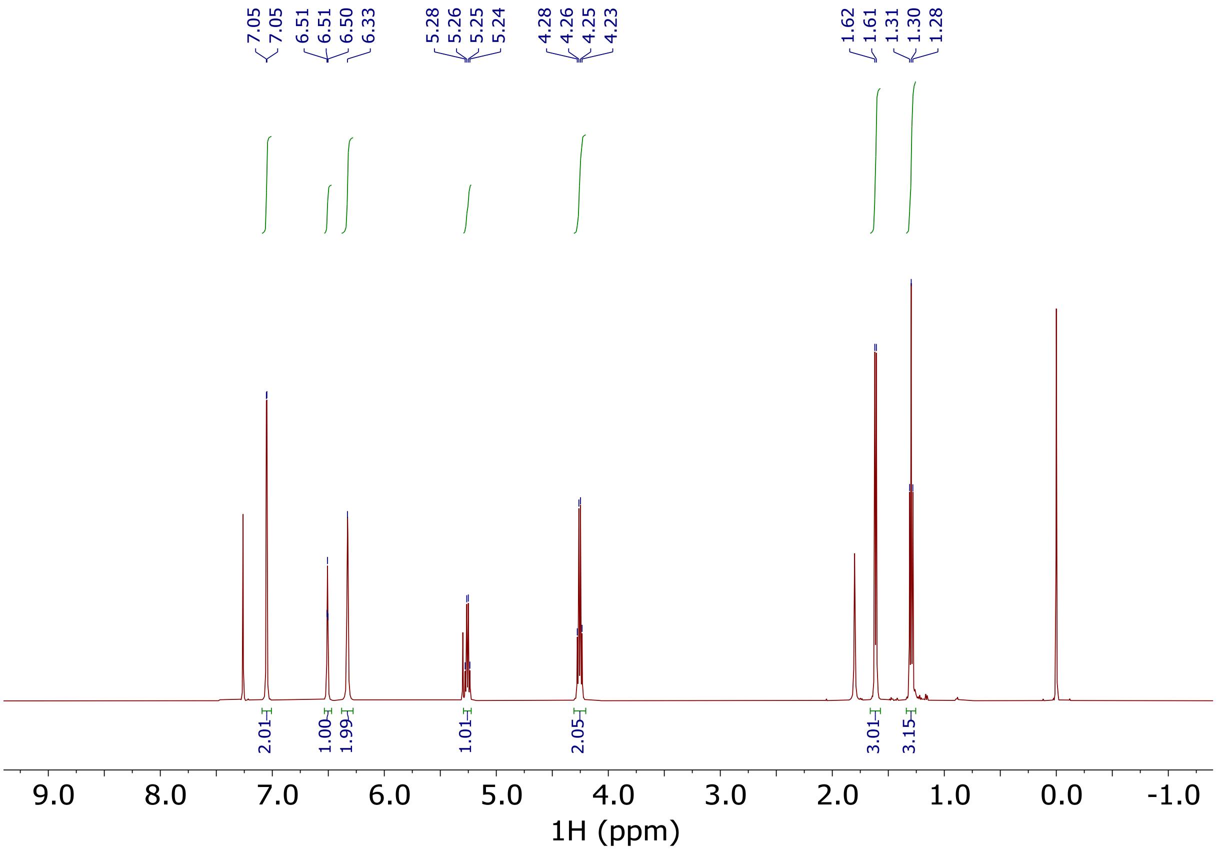

Figure S19. ^{1}H NMR spectrum (500 MHz, $CDCl_3$, 298K) of **G3R**.

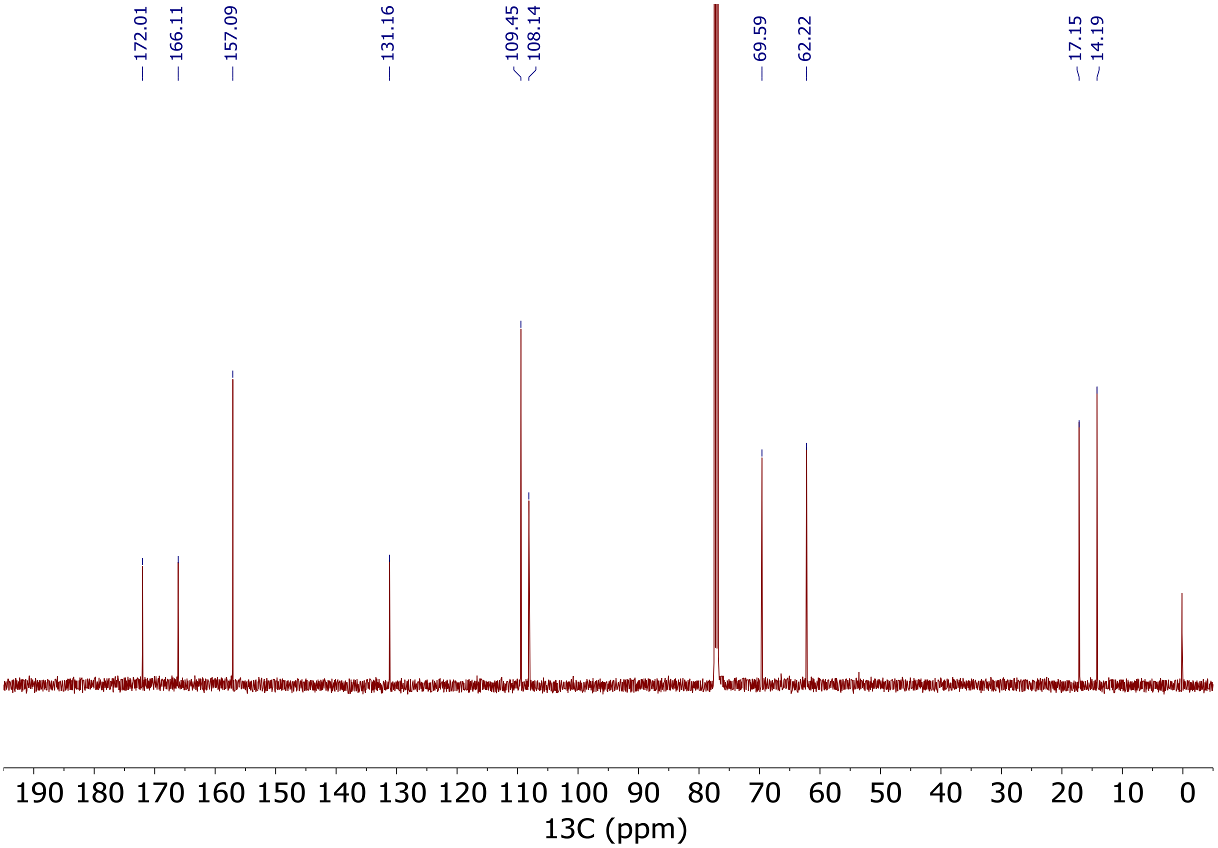

Figure S20. ^{13}C NMR spectrum (126 MHz, $CDCl_3$, 298K) of **G3R**.

G3S

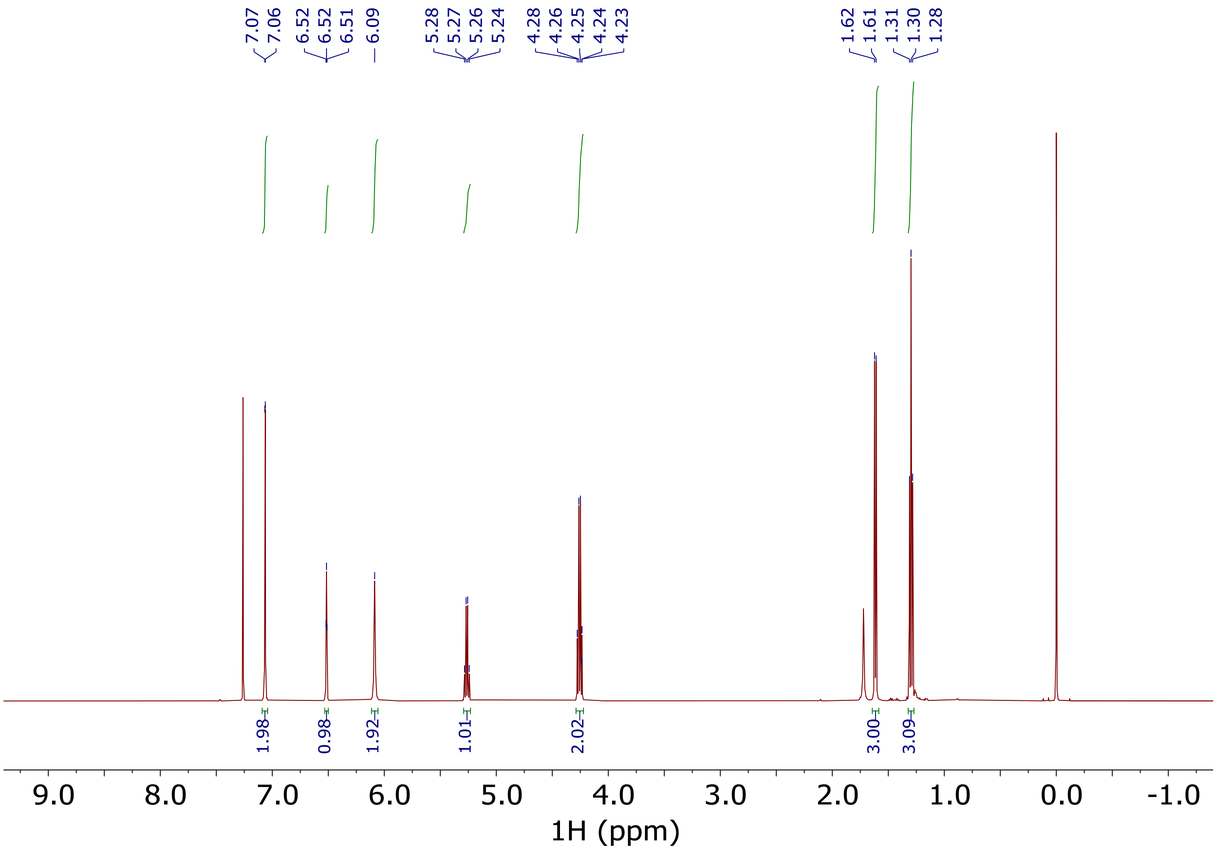

Figure S21. ^{1}H NMR spectrum (500 MHz, $CDCl_3$, 298K) of **G3S**.

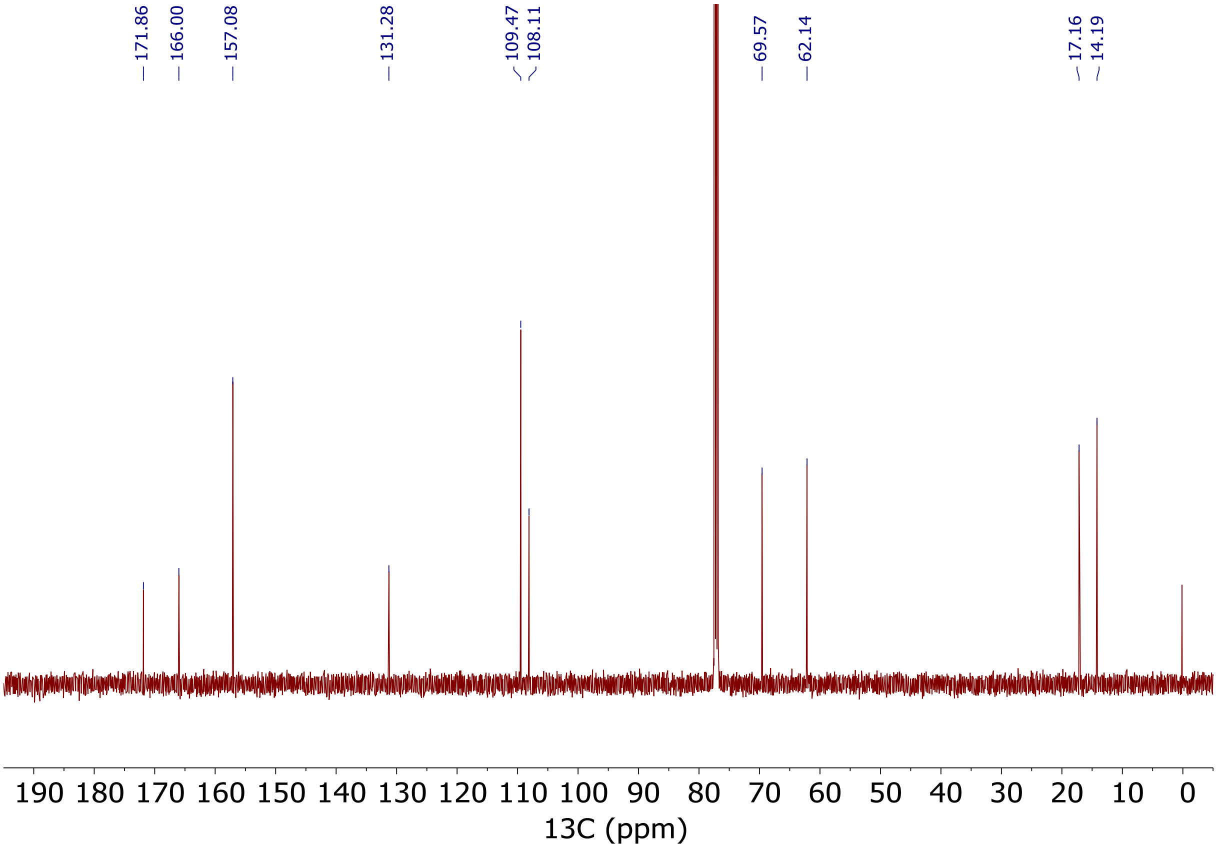

Figure S22. ^{13}C NMR spectrum (126 MHz, $CDCl_3$, 298K) of **G3S**.

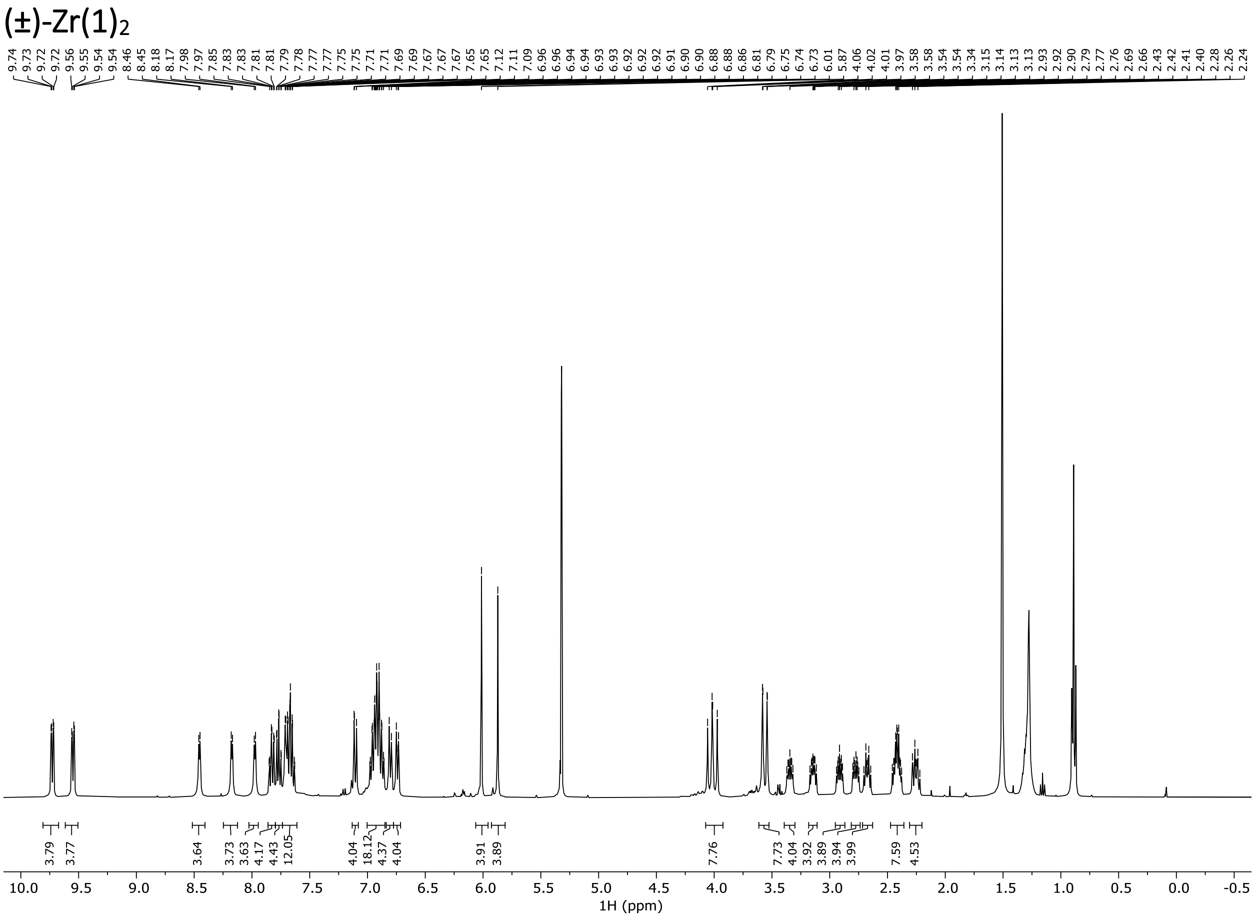

Figure S23. ^{1}H NMR (400 MHz, CD_2Cl_2, 298 K) of **(±)-Zr(1)₂**.

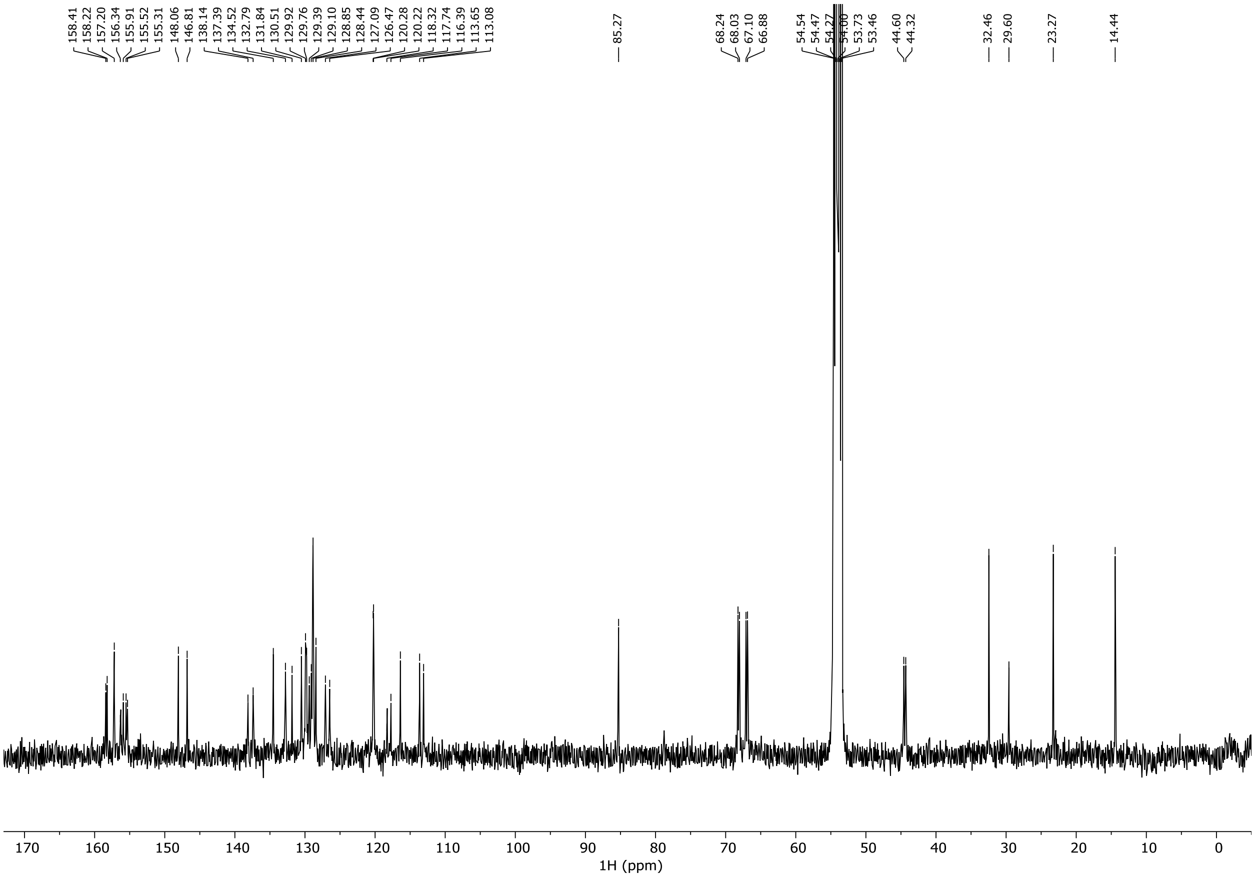

Figure S24. ^{13}C NMR (101 MHz, CD_2Cl_2, 298 K) of **(±)-Zr(1)$_2$**.

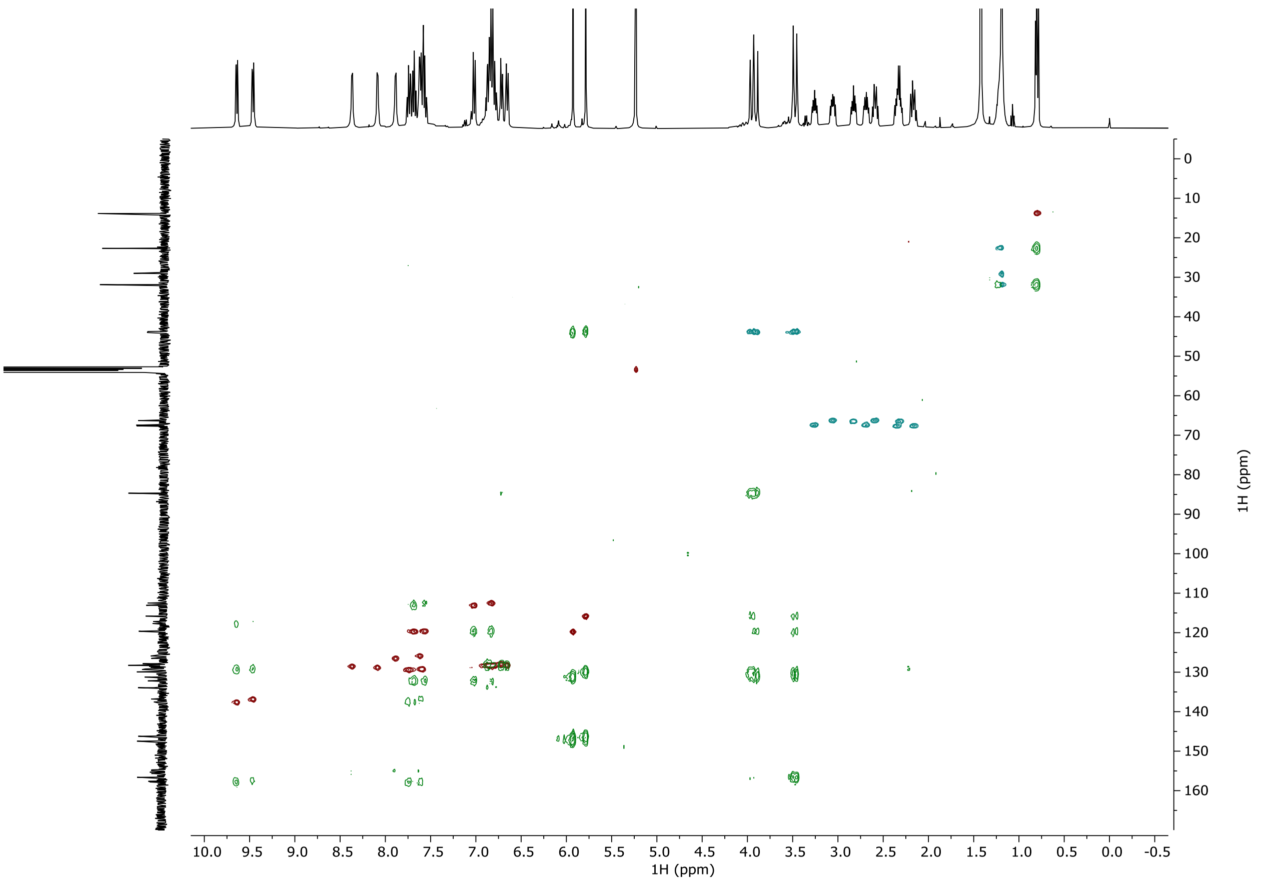

Figure S25. ^{1}H-^{13}C HSQC (400 MHz, CD_2Cl_2, 298 K) (blue for CH_2, red for CH_1/CH_3) HMBC (green) overlay of **(±)-Zr(1)$_2$**.

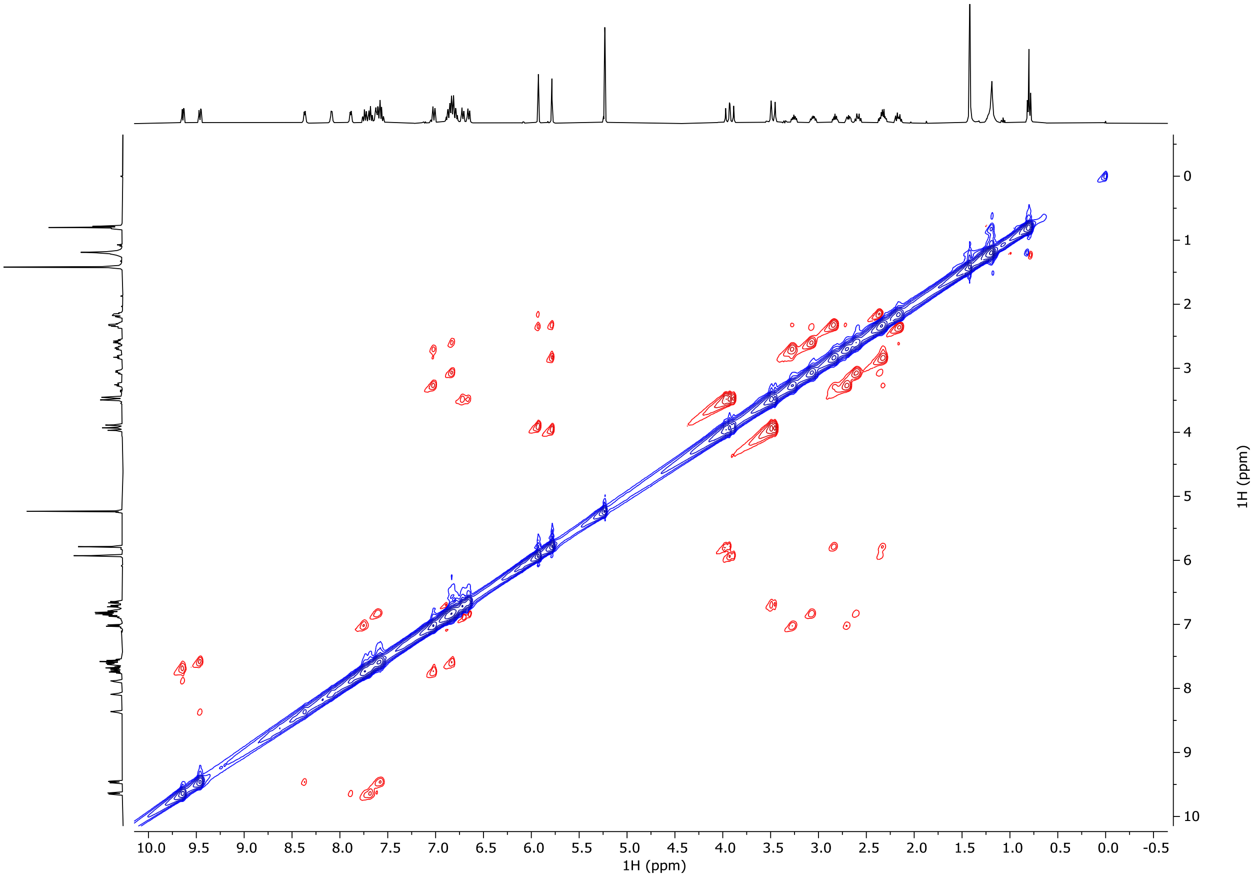

Figure S26. ^{1}H-^{1}H ROESY (400 MHz, CD_2Cl_2, 298 K) spectrum of **(±)-Zr(1)$_2$**.

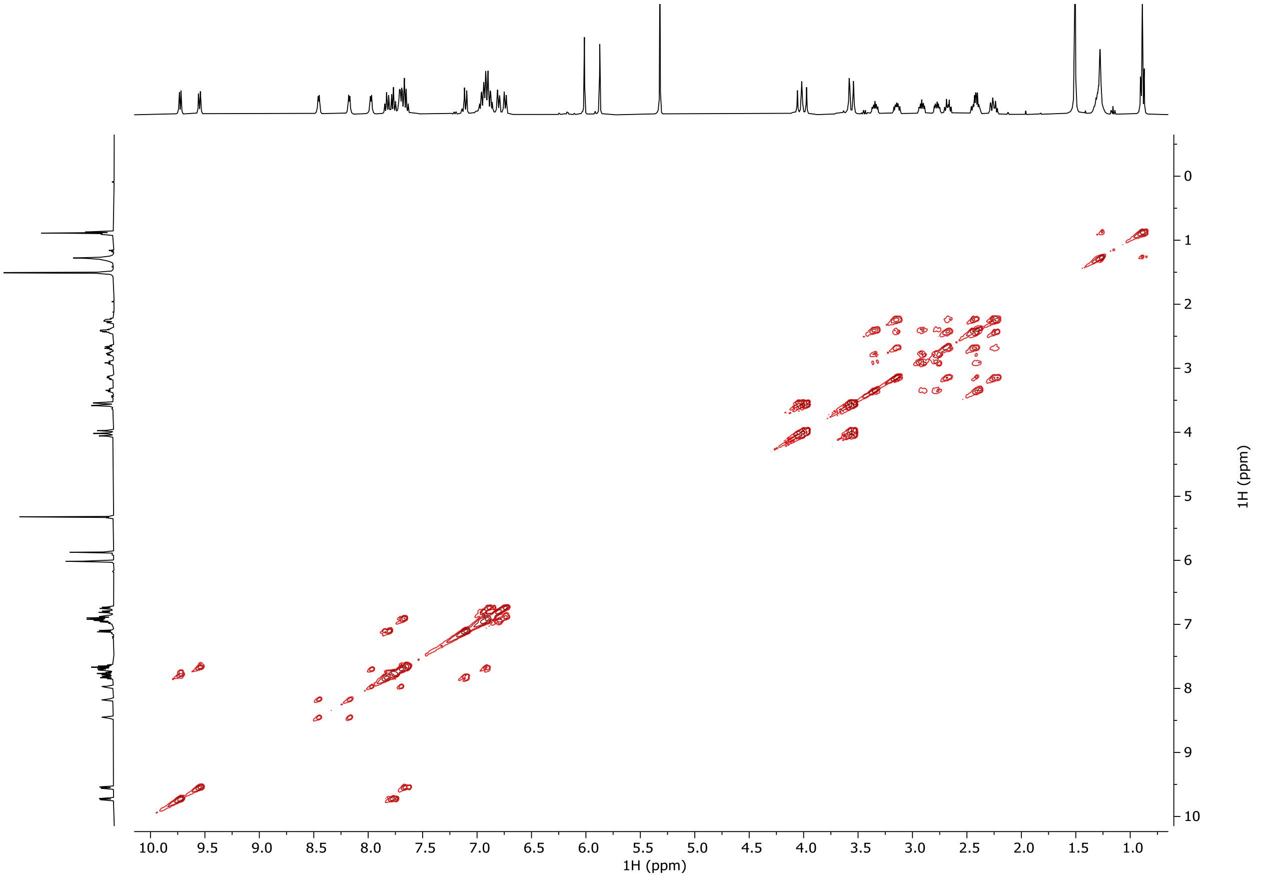

Figure S27. ^{1}H-^{1}H COSY (400 MHz, CD_2Cl_2, 298 K) spectrum of **(±)-Zr(1)$_2$**.

(±)-Zr(1)$_2$ with **G1**

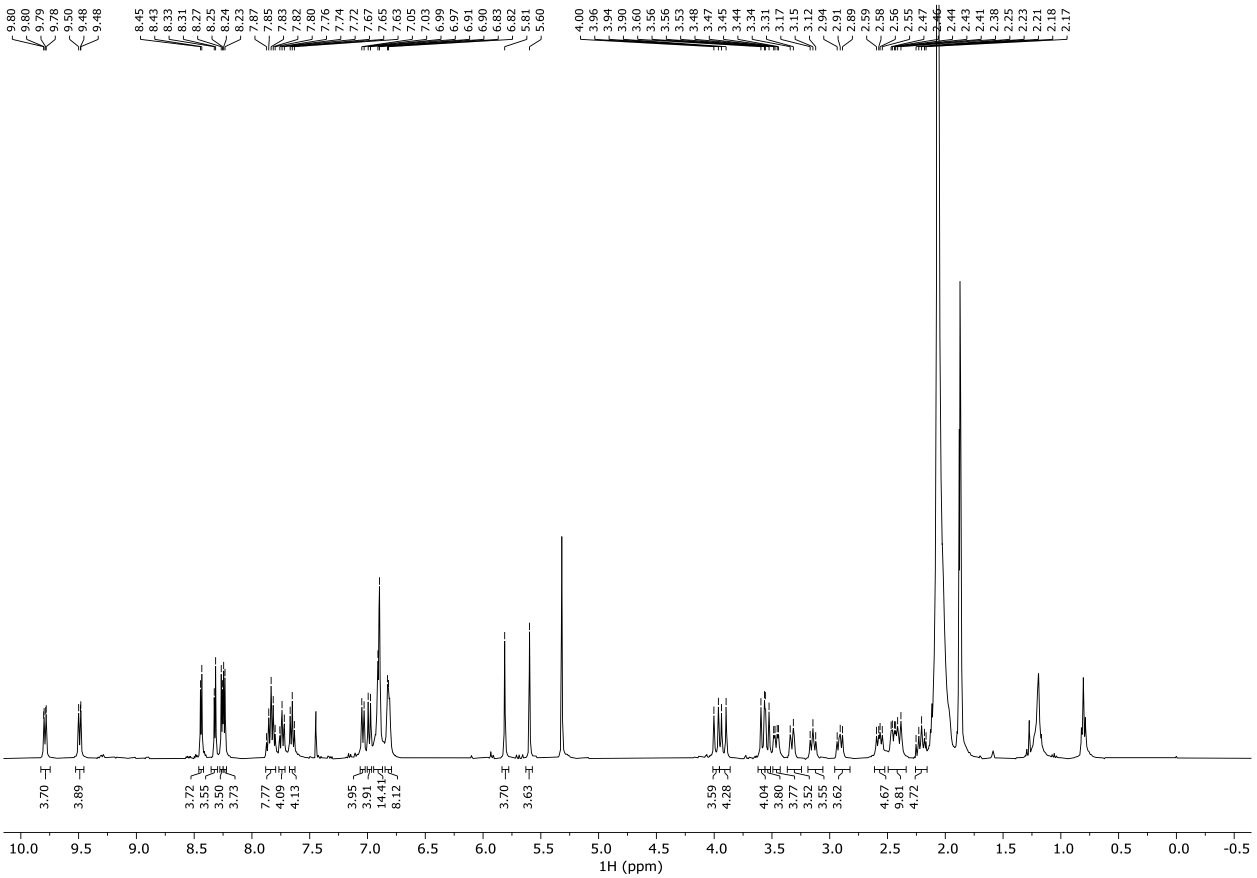

Figure S28. ^{1}H NMR (400 MHz, CD_2Cl_2:CD_3CN, 1:1, v/v, 298 K) of **(±)-Zr(1)$_2$** with **G1** (2 equiv).

(±)-Zr(1)$_2$ with **G1** (0, 1 and 2 equiv) at -28°C

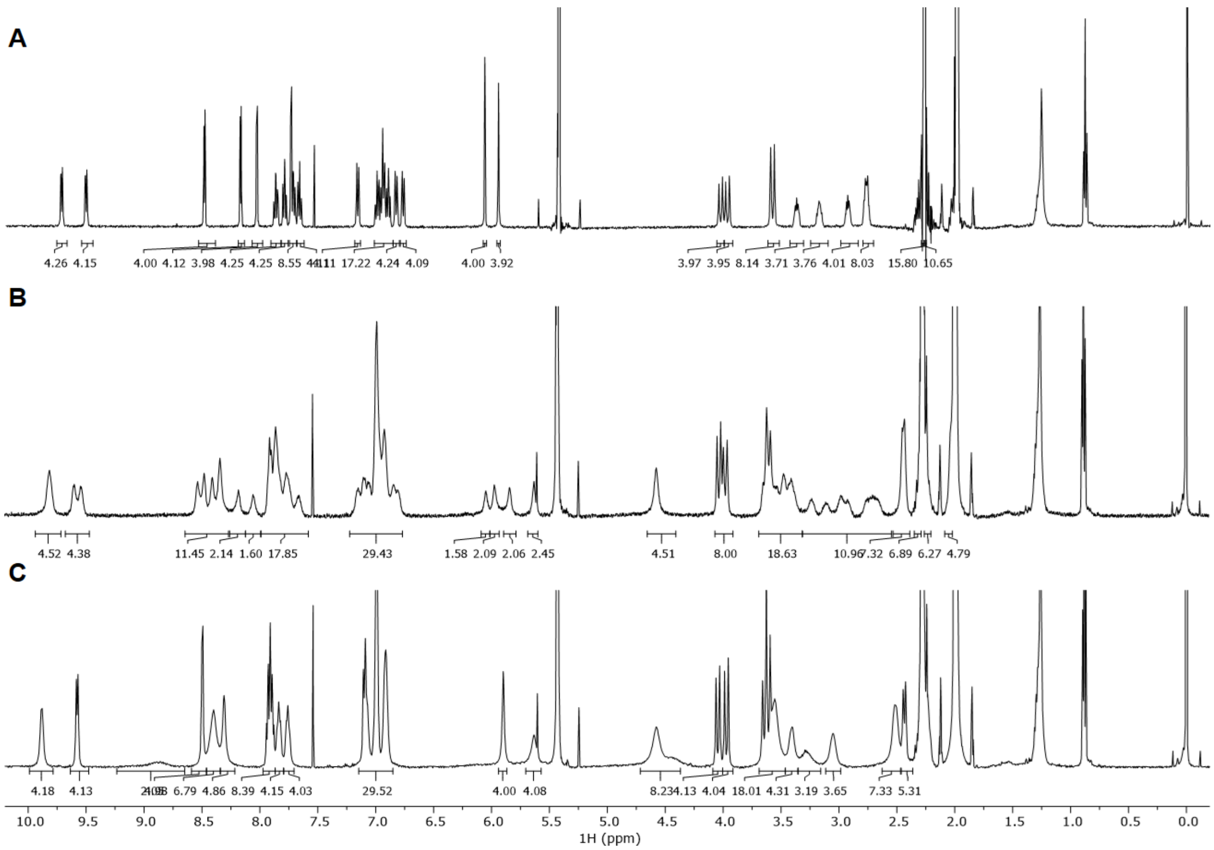

Figure S29. ^{1}H NMR spectra (500 MHz) in CD_2Cl_2/CD_3CN (1:1, v/v) at -28°C of (A) **(±)-Zr(1)$_2$**, (B) its 1:1 host-guest complex with **G1** and (C) its 1:2 host-guest complex.

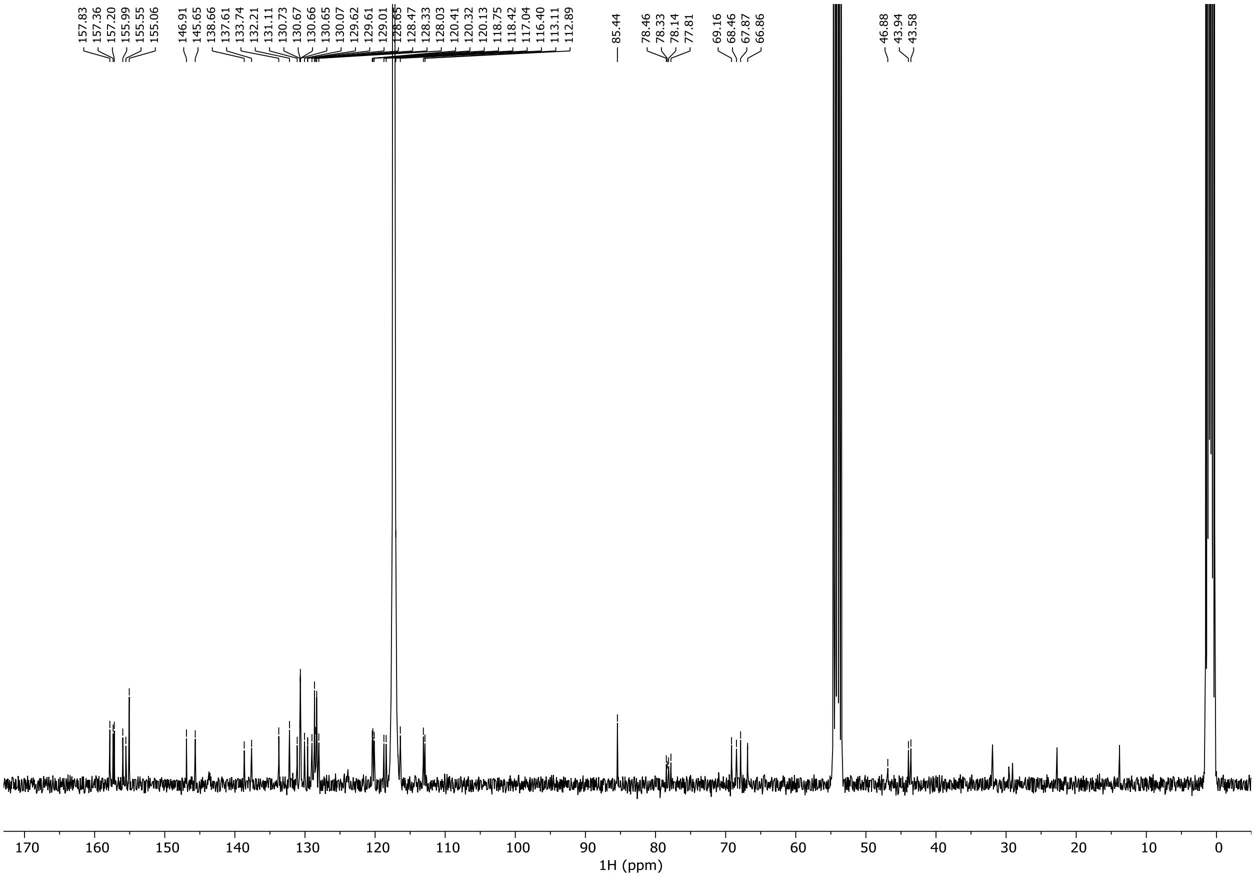

Figure S30. ^{13}C NMR (101 MHz, CD_2Cl_2: CD_3CN, 1:1, v/v, 298 K) of **(±)-Zr(1)$_2$** with **G1** (2 equiv).

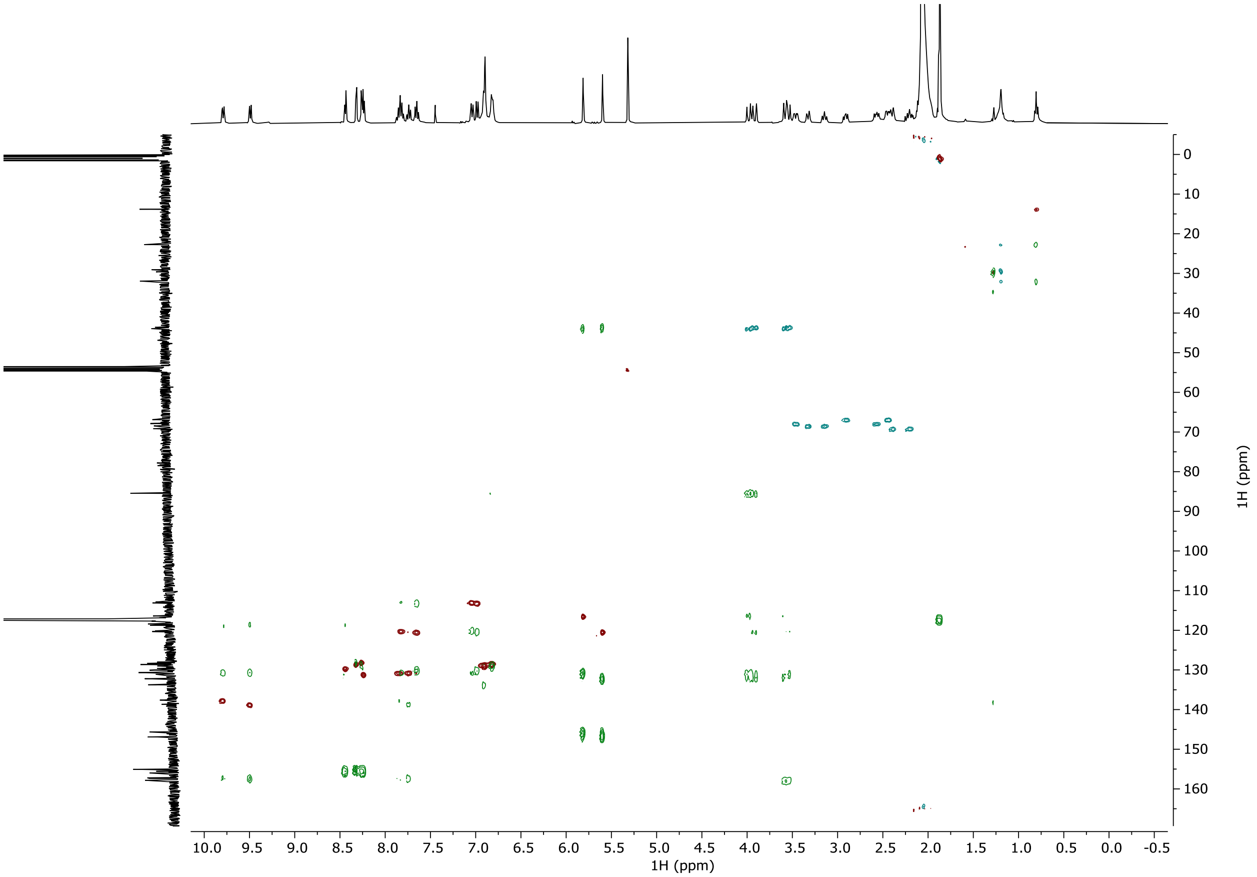

Figure S31. ^{1}H-^{13}C HSQC (400 MHz, CD_2Cl_2:CD_3CN, 1:1, v/v, 298 K) (blue for CH_2, red for CH_1/CH_3) HMBC (green) overlay of **(±)-Zr(1)₂** with **G1** (2 equiv).

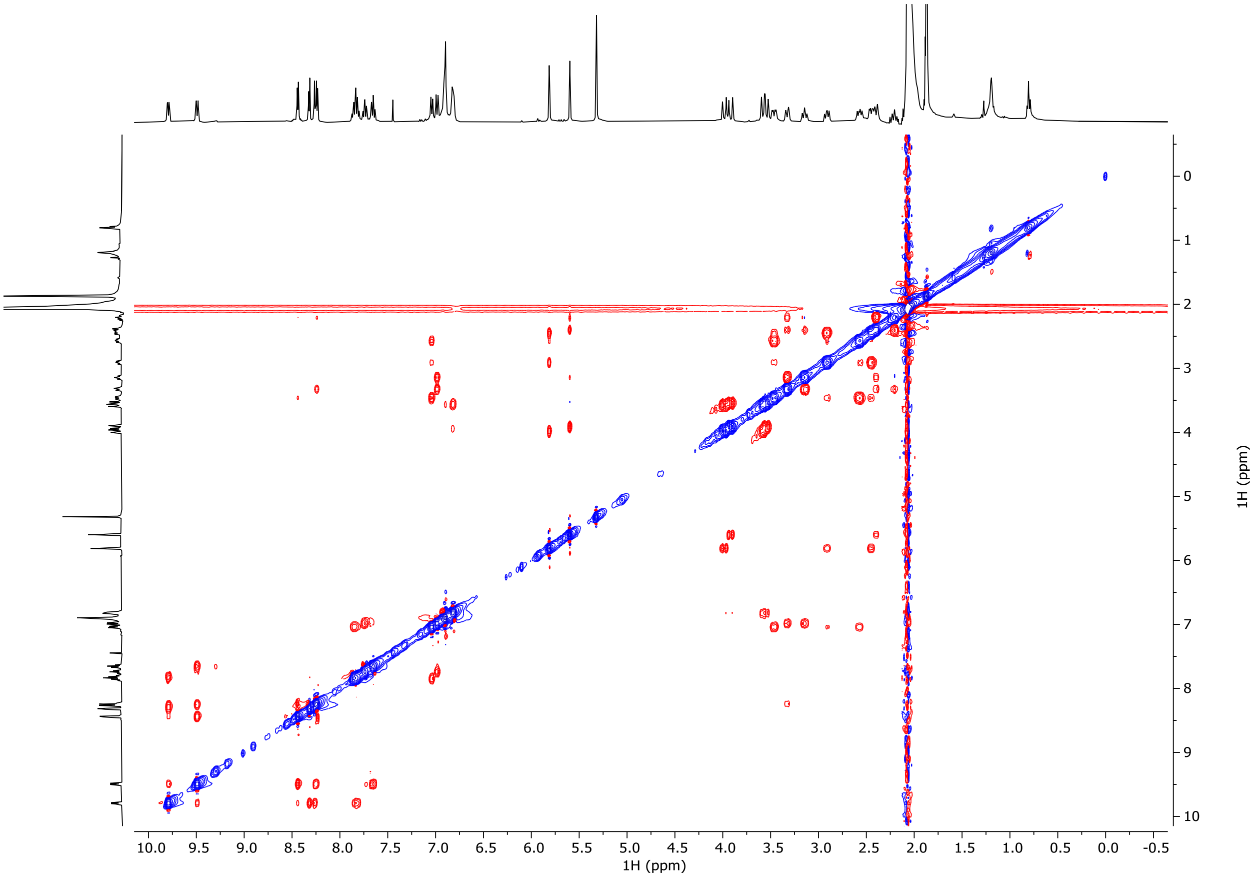

Figure S32. ^{1}H-^{1}H ROESY (400 MHz, CD_2Cl_2:CD_3CN, 1:1, v/v, 298 K) spectrum **(±)-Zr(1)$_2$** with **G1** (2 equiv).

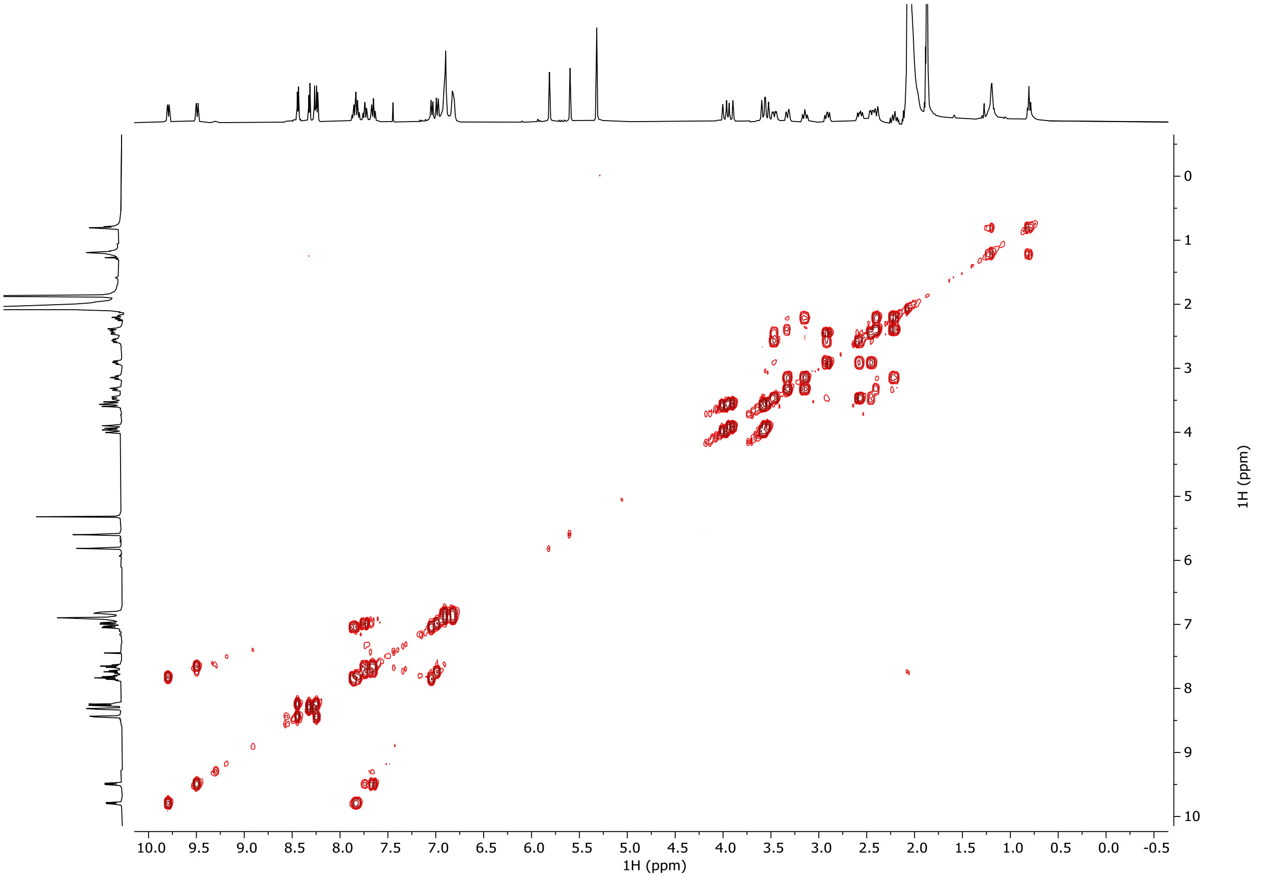

Figure S33. ^1H-^1H COSY (400 MHz, CD_2Cl_2:CD_3CN, 1:1, v/v, 298 K) spectrum of **(±)-Zr(1)$_2$** with **G1** (2 equiv).

Stacks of titration spectra

H_2**1** with **G2**

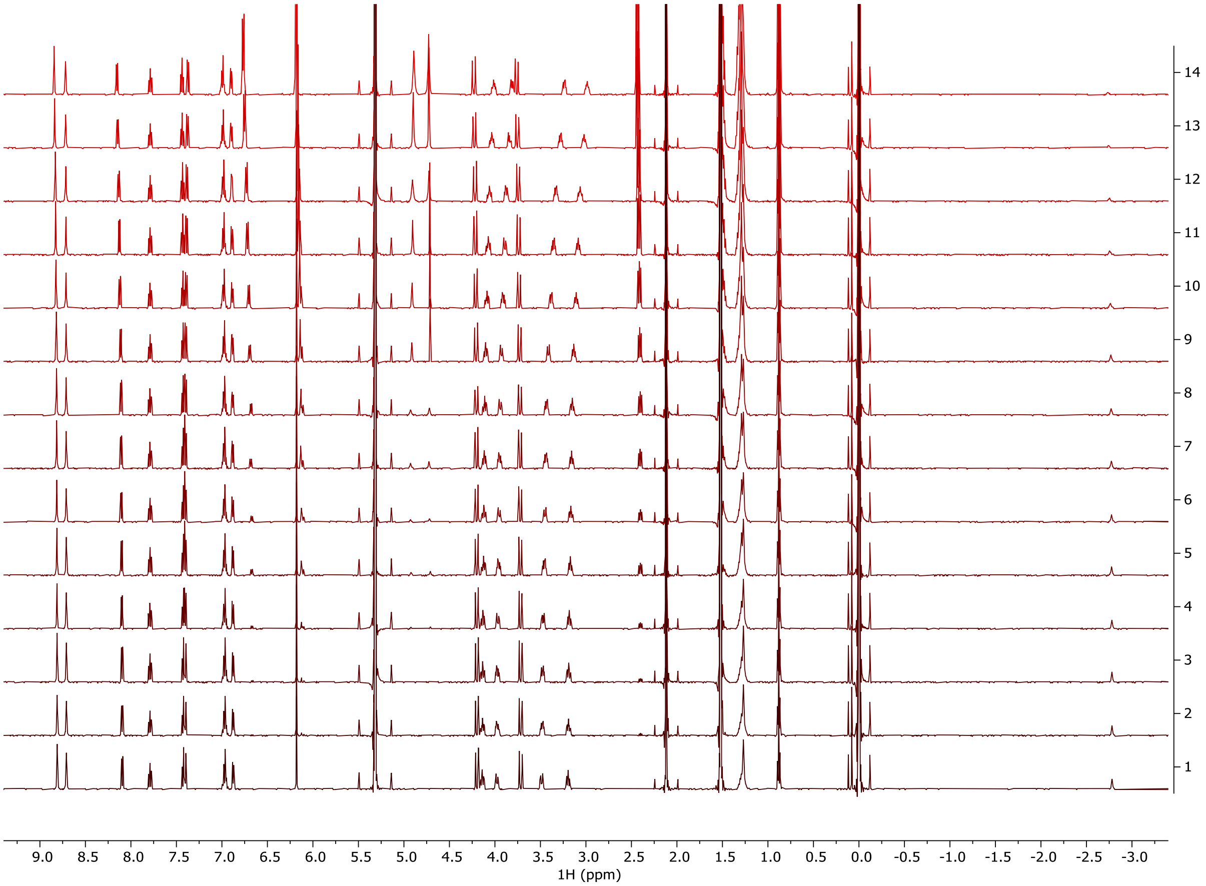

Figure S34. ^{1}H NMR (500 MHz, CD_2Cl_2, 298 K) titration of **$H_2$1** with **G2** with increasing guest concentration (from bottom to top).

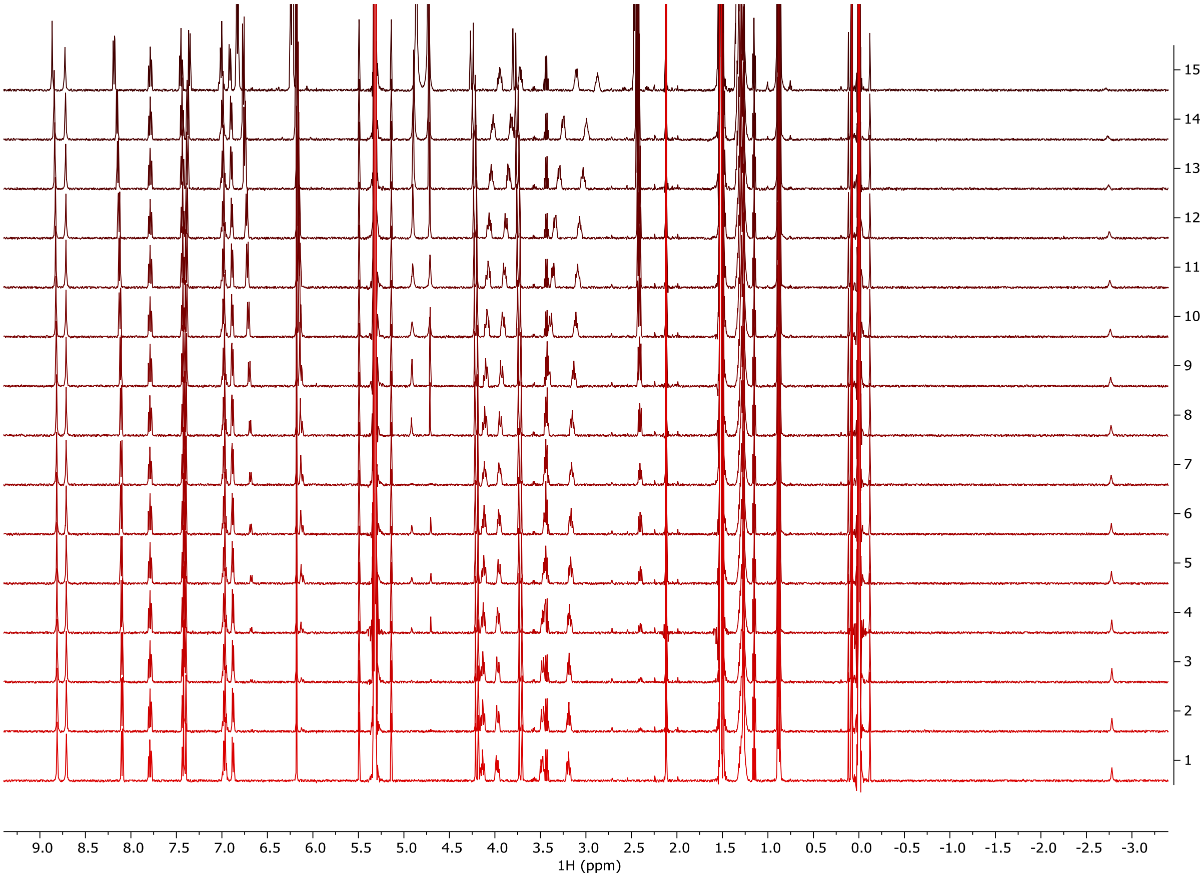

Figure S35. ^{1}H NMR (500 MHz, CD_2Cl_2, 298 K) titration of **$H_2$1** with **G2** (duplo experiment) with increasing guest concentration (from bottom to top).

(±)-Zr(1)$_2$ with **G2**

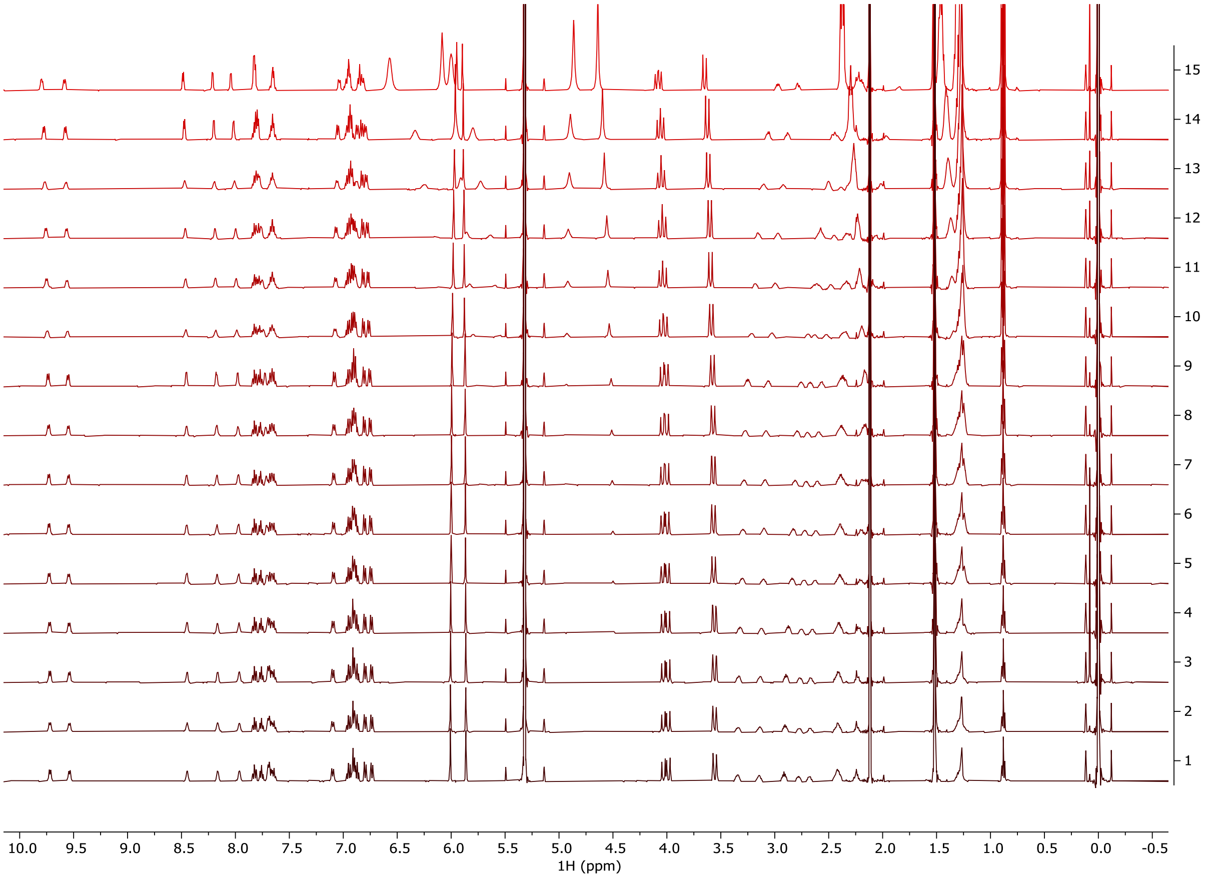

Figure S36. ^{1}H NMR (500 MHz, CD_2Cl_2, 298 K) titration of **(±)-Zr(1)$_2$** with **G2** with increasing guest concentration (from bottom to top).

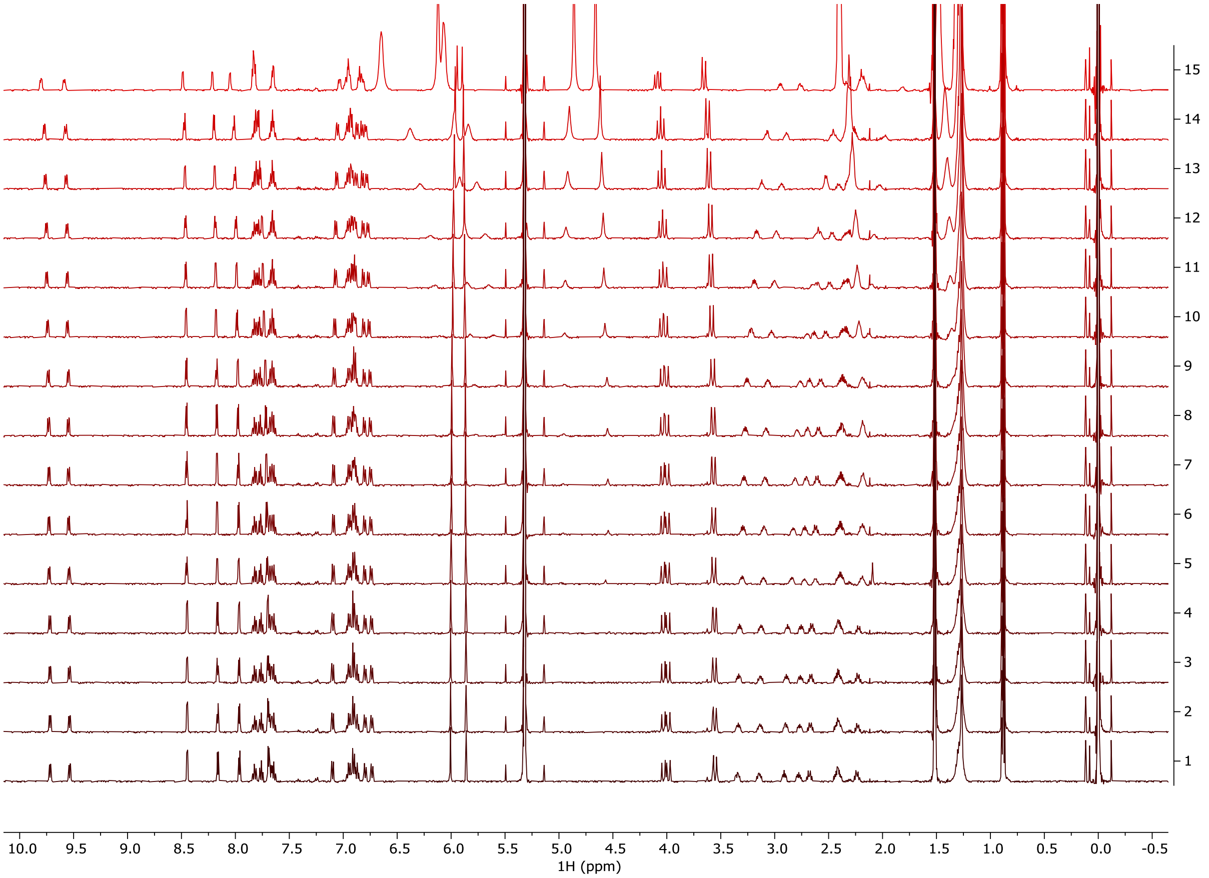

Figure S37. ^{1}H NMR (500 MHz, CD_2Cl_2, 298 K) titration of **(±)-Zr(1)$_2$** with **G2** (duplo experiment) with increasing guest concentration (from bottom to top).

(+)-Zr(1)$_2$ with **G3R**

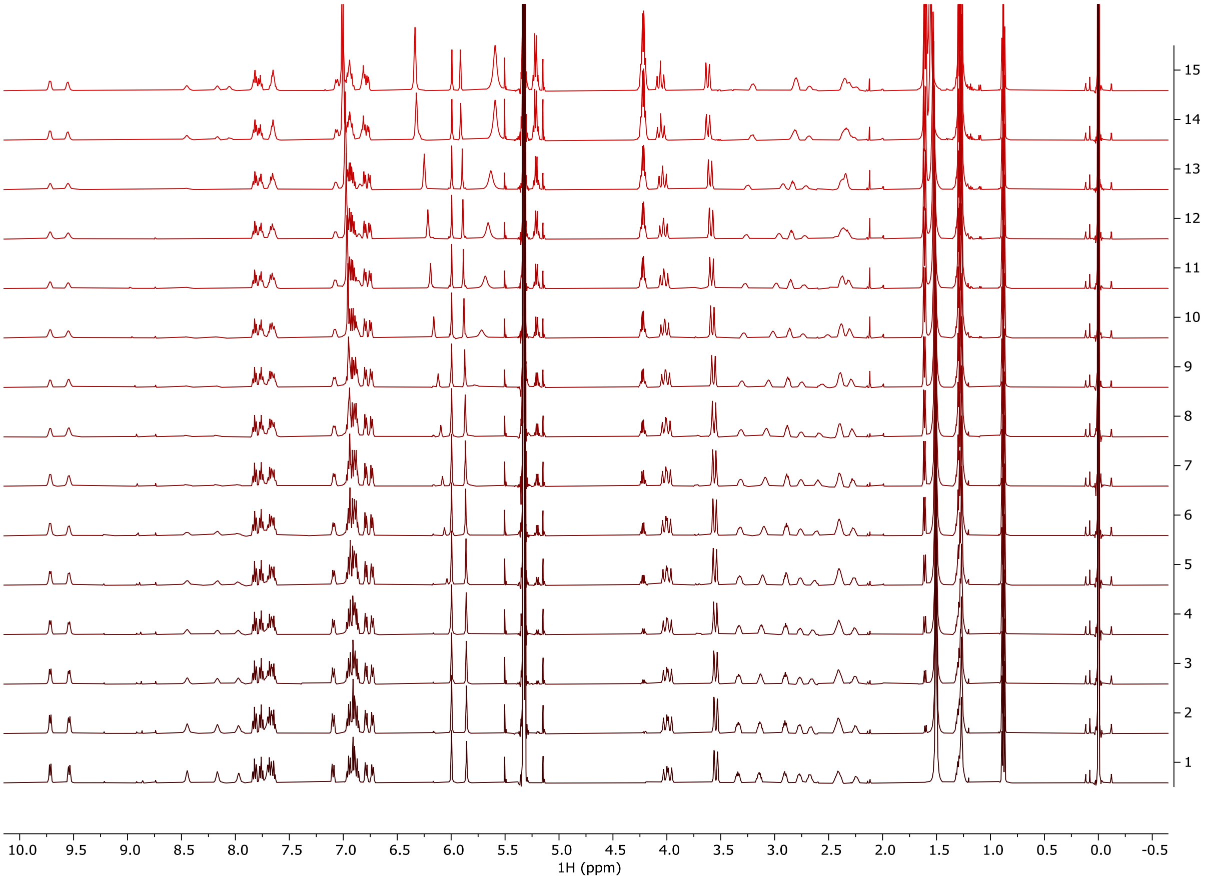

Figure S38. ^{1}H NMR (500 MHz, CD_2Cl_2, 298 K) titration of **(+)-Zr(1)$_2$** with **G3R** with increasing guest concentration from bottom to top

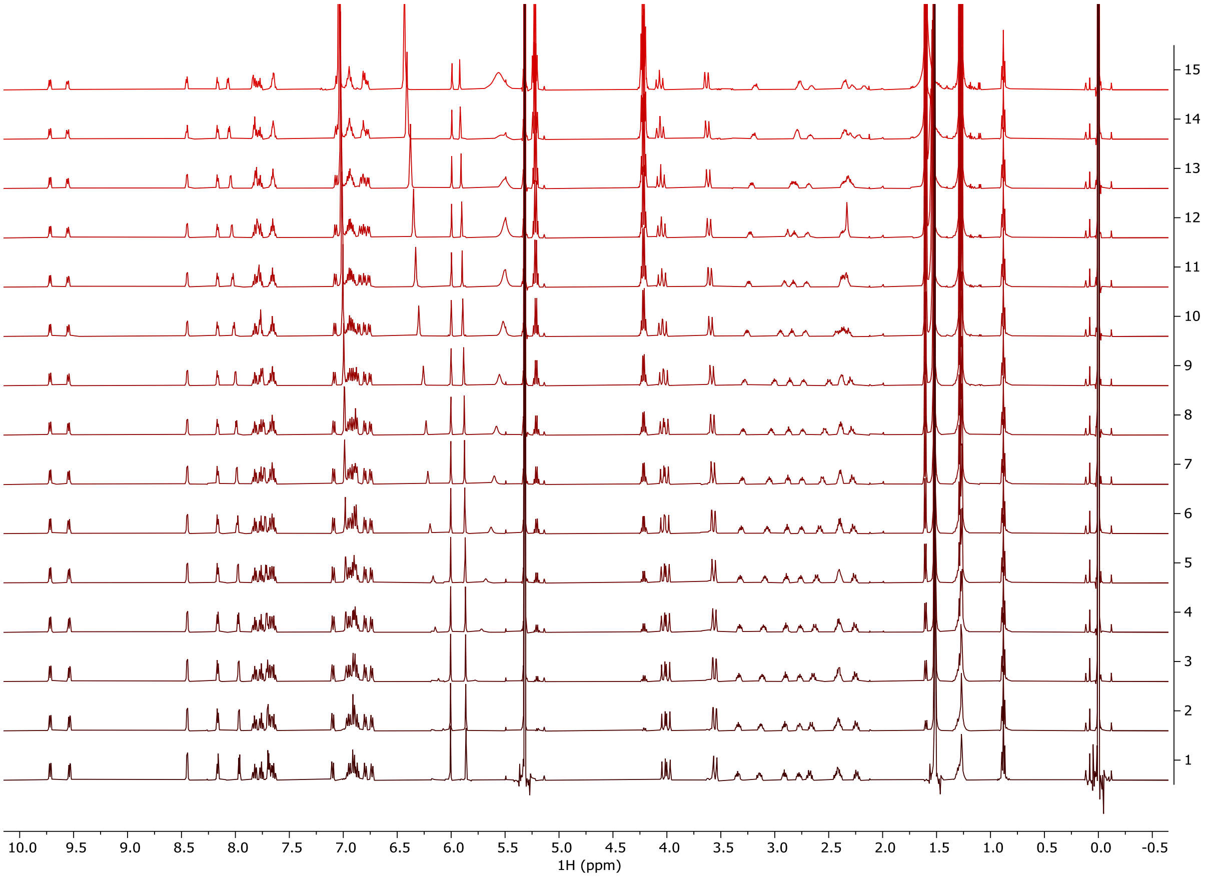

Figure S39. ^{1}H NMR (500 MHz, CD_2Cl_2, 298 K) titration of **(+)-Zr(1)$_2$** with **G3R** (duplo experiment) with increasing guest concentration (from bottom to top).

(+)-Zr(1)$_2$ with **G3S**

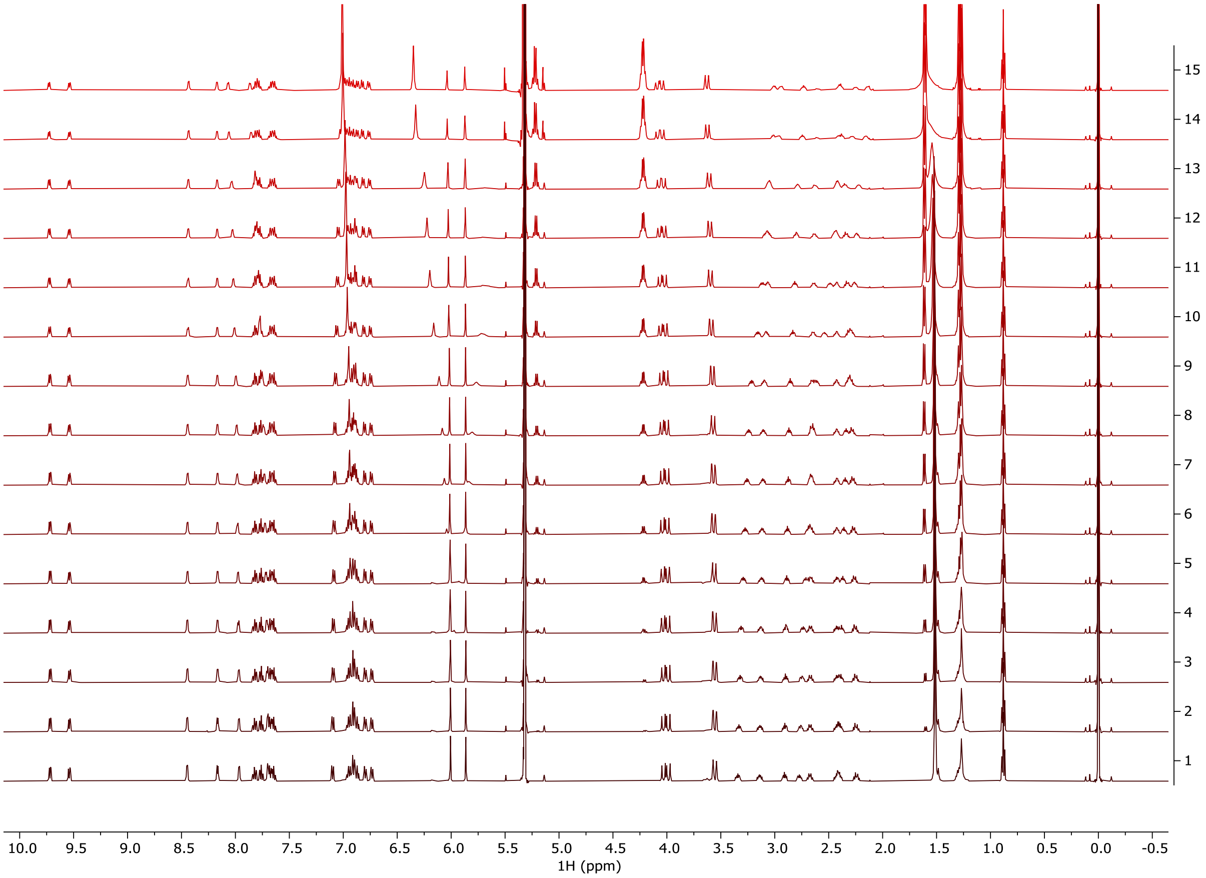

Figure S40. ^{1}H NMR (500 MHz, CD_2Cl_2, 298 K) titration of **(+)-Zr(1)$_2$** with **G3S** with increasing guest concentration (from bottom to top).

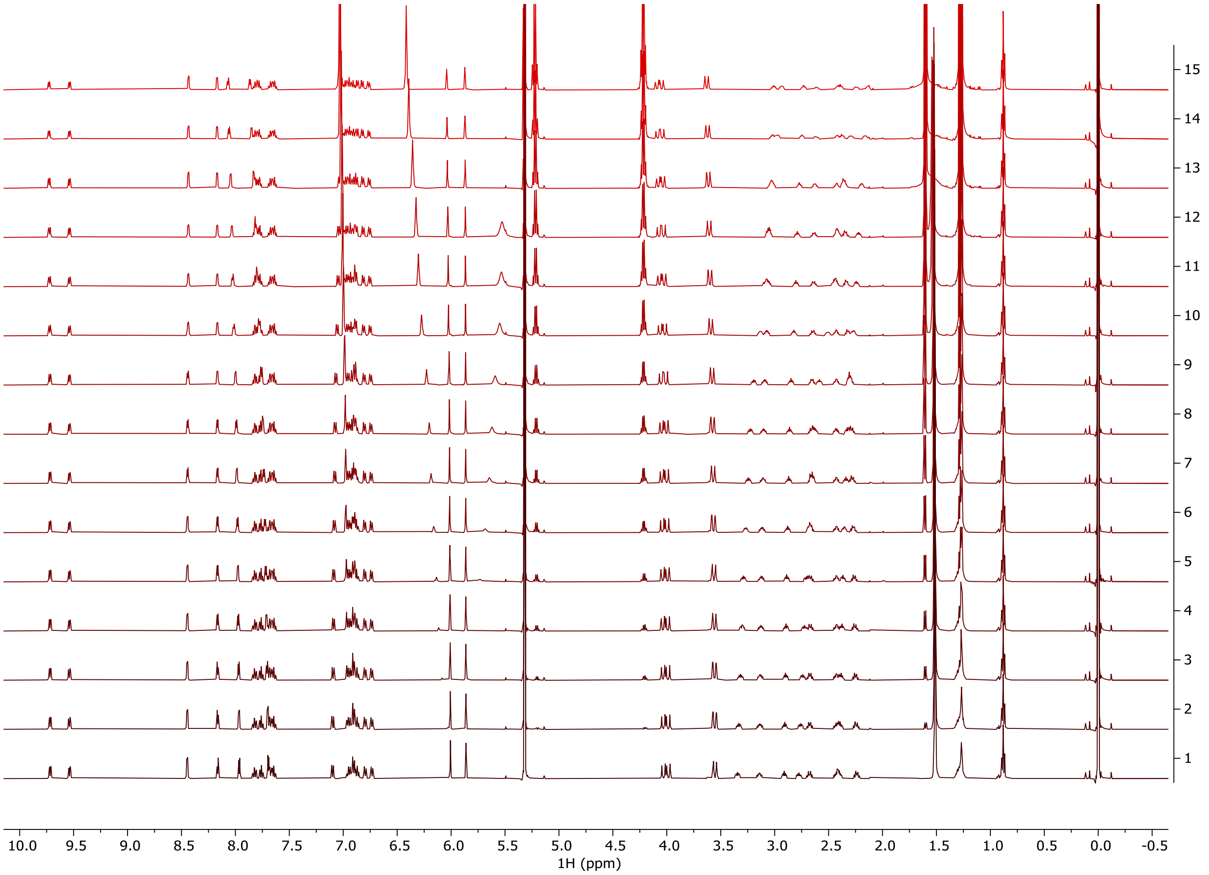

Figure S41. ^{1}H NMR (500 MHz, CD_2Cl_2, 298 K) titration of **(+)-Zr(1)$_2$** with **G3S** (duplo experiment) with increasing guest concentration (from bottom to top).

(–)-Zr(1)$_2$ with **G3R**

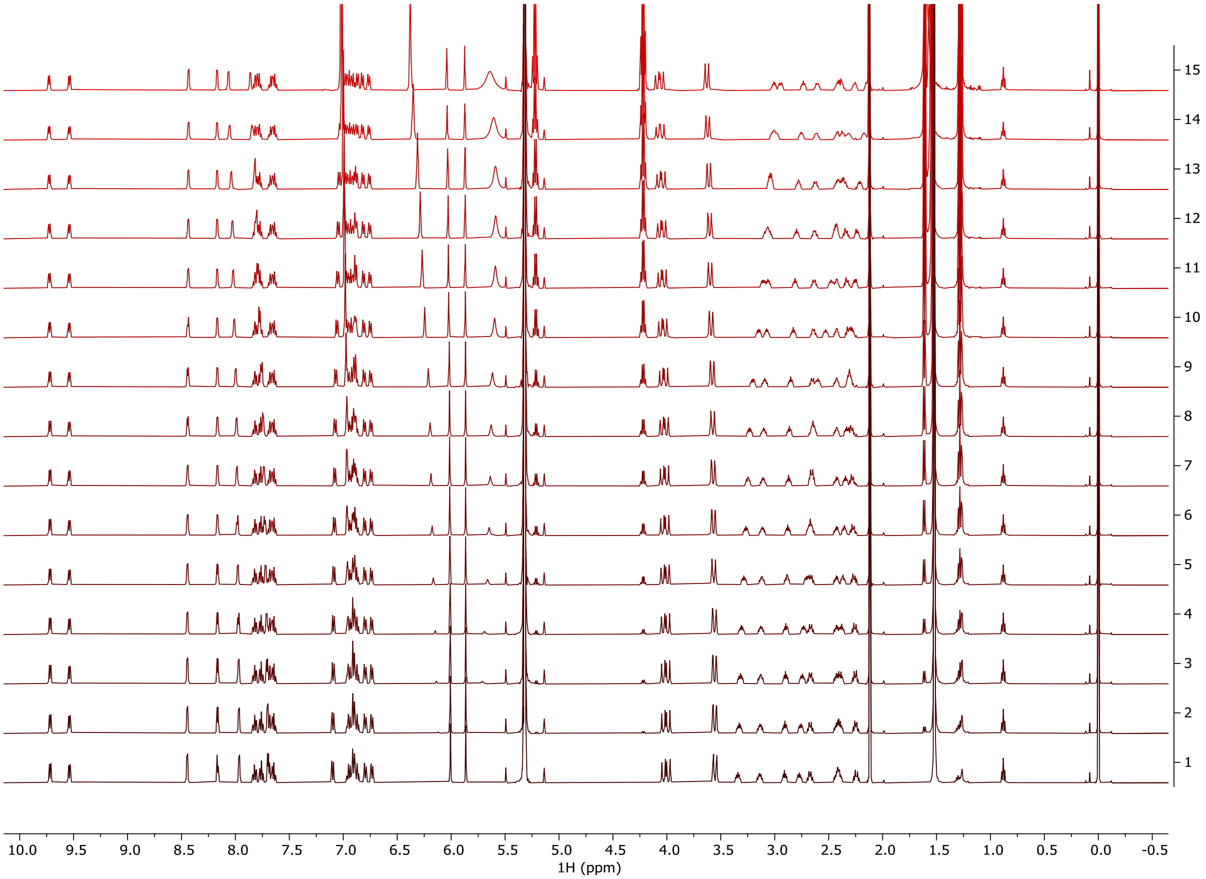

Figure S42. ^{1}H NMR (500 MHz, CD_2Cl_2, 298 K) titration of **(–)-Zr(1)$_2$** with **G3R** with increasing guest concentration (from bottom to top).

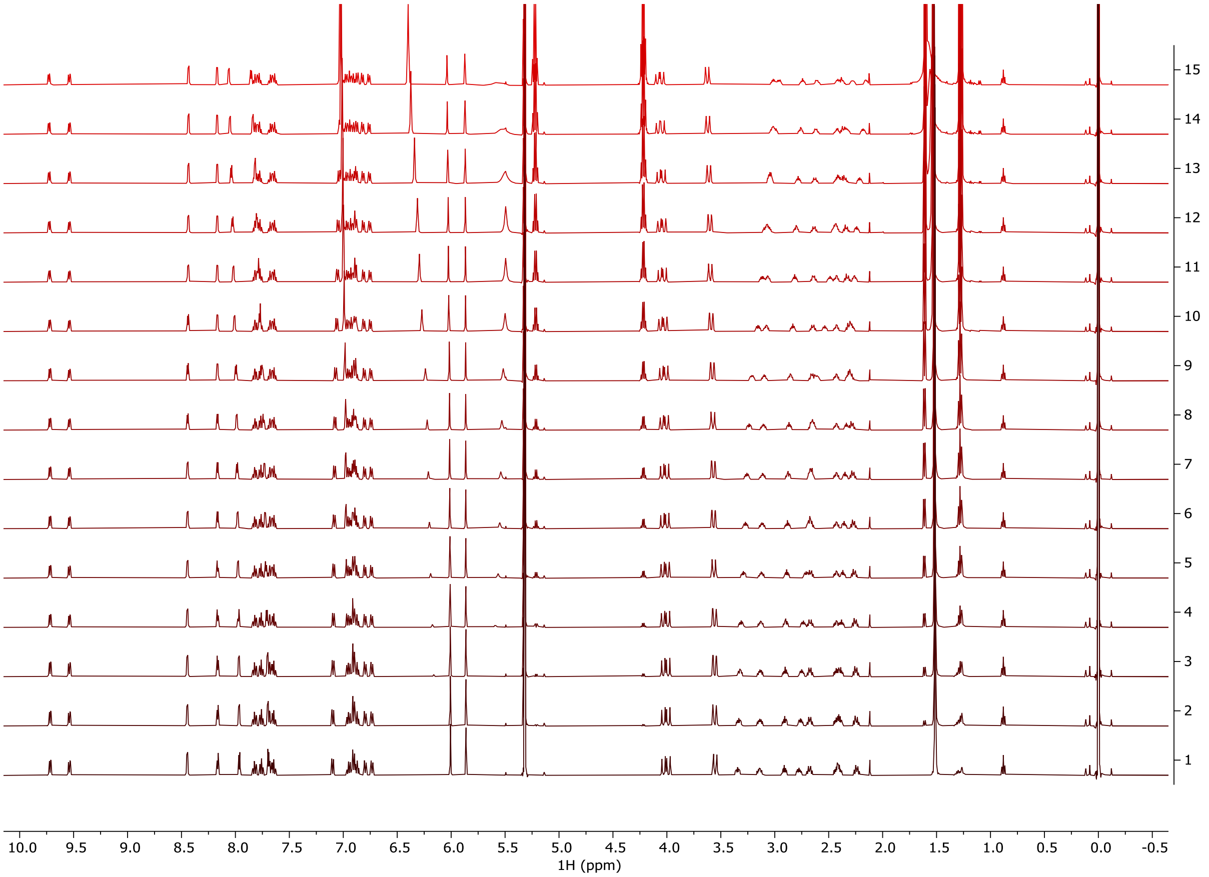

Figure S43. ^{1}H NMR (500 MHz, CD_2Cl_2, 298 K) titration of **(–)-Zr(1)$_2$** with **G3R** (duplo experiment) with increasing guest concentration (from bottom to top).

(–)-Zr(1)$_2$ with **G3S**

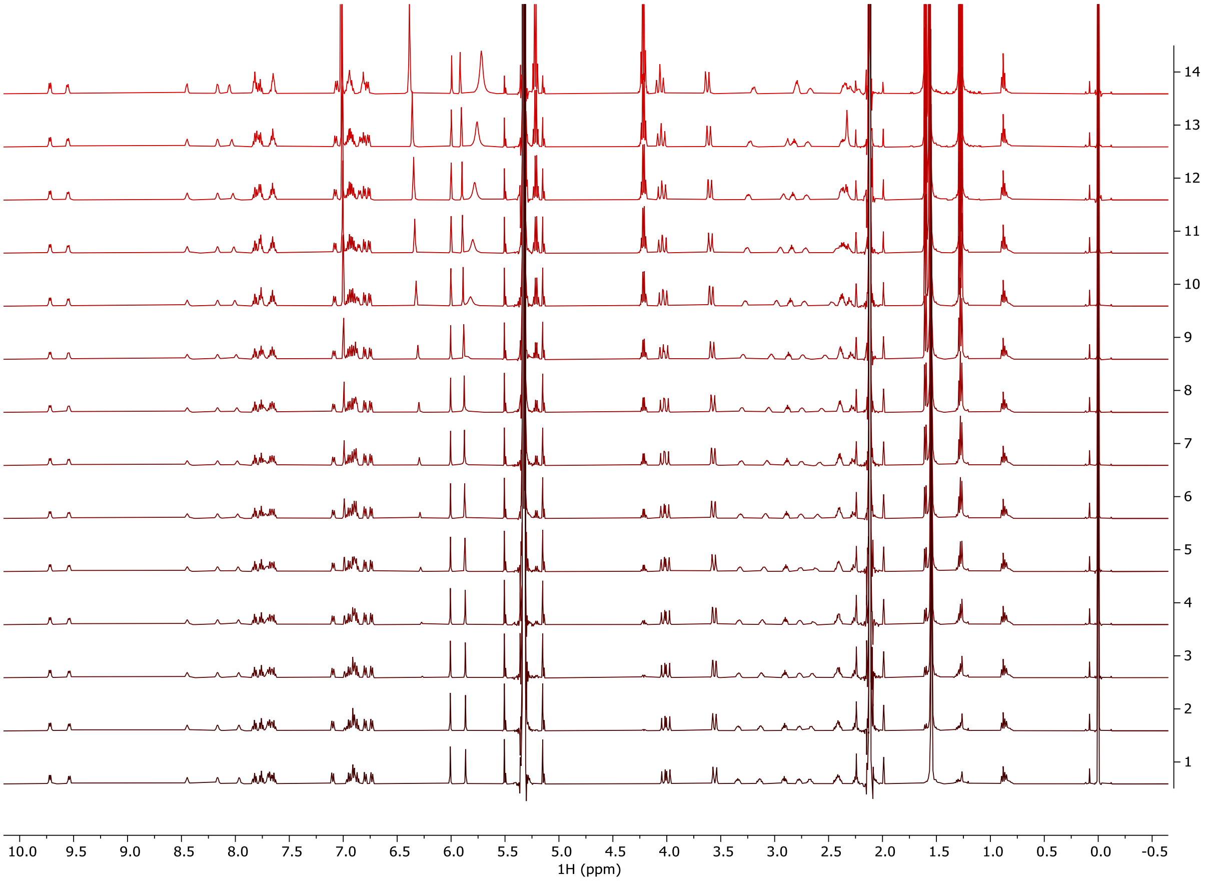

Figure S44. ^{1}H NMR (500 MHz, CD_2Cl_2, 298 K) titration of **(–)-Zr(1)$_2$** with **G3S** with increasing guest concentration (from bottom to top).

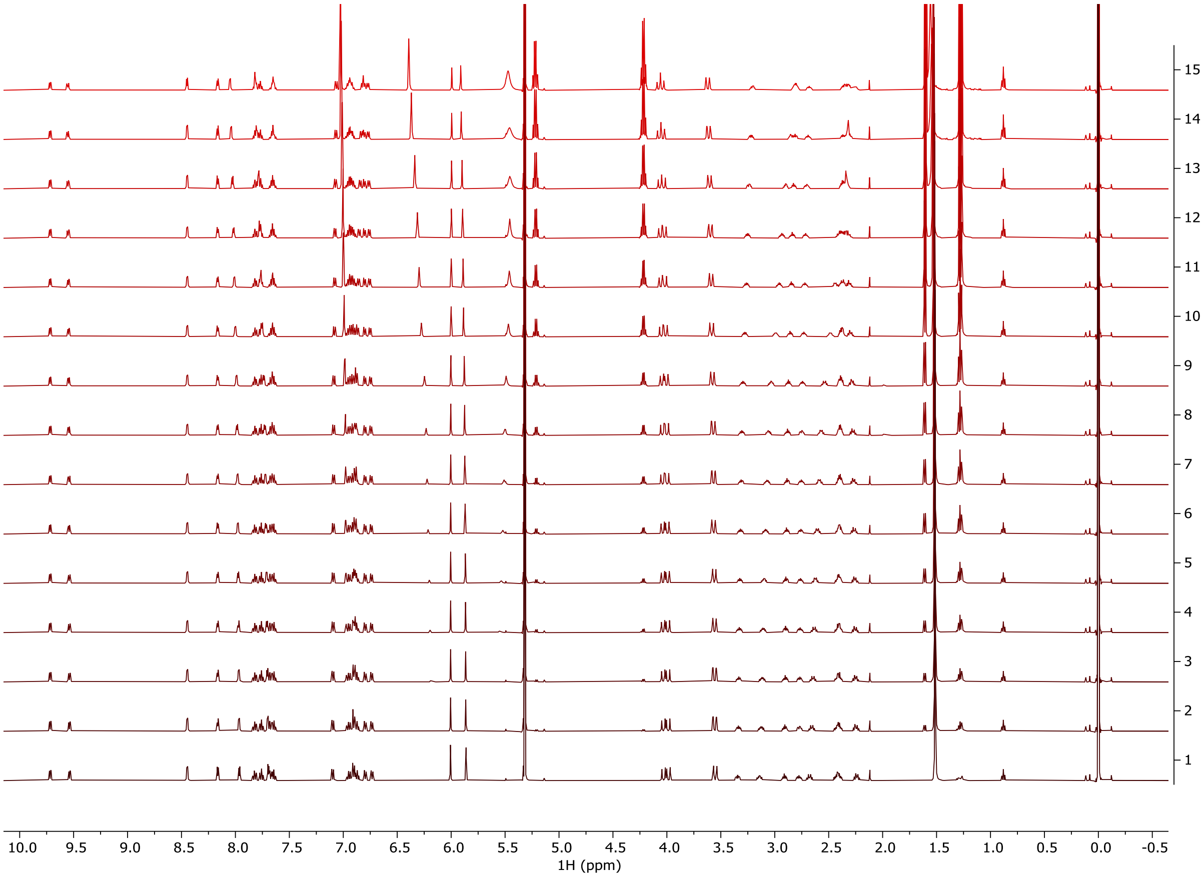

Figure S45. ^{1}H NMR (500 MHz, CD_2Cl_2, 298 K) titration of **(–)-Zr(1)$_2$** with **G3S** (duplo experiment) with increasing guest concentration (from bottom to top).

Molecular Modelling

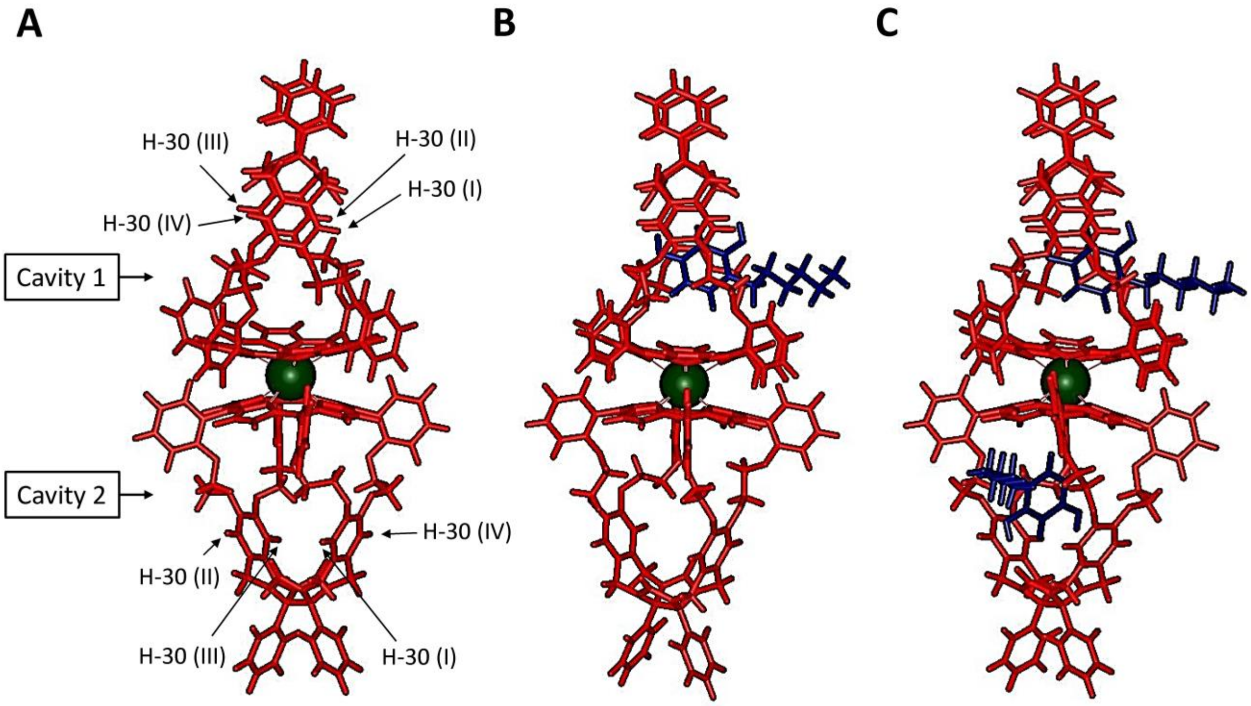

Figure S46. Calculated molecular models of (A) **(–)-Zr(1)$_2$**, (B) its 1:1 complex with **G2** (in blue), and (C) its 1:2 complex with **G2**. The calculated distances between the various side-wall protons H-30 are summarized in Table 4.

References

S1. http://supramolecular.org

S2. D. Brynn Hibbert, Pall Thordarson, *Chem. Commun.*, **2016**, *52*, 12792-12805

S3. K. Kim, W.S. Lee, H.-J. Kim, S.-H. Cho, G.S. Girolami, P.A. Gorlin, K.S. Suslick, *Inorg. Chem.*, **1991**, *30*, 2652 – 2656

S4. P.J. Gilissen, A. Swartjes, B. Spierenburg, J.P.J. Bruekers, P. Tinnemans, P.B. White, F.P.J.T. Rutjes, R.J.M. Nolte, J.A.A.W. Elemans, *Tetrahedron*, **2019**, *75*, 4640 – 4647

S5. P. Rajamalli, E. Prasad, *New J. Chem.,* **2011**, *35*, 1541–1548

S6. C.J. Hawker, R. Lee, J.M.J. Fréchet, *J. Am. Chem. Soc.* **1991**, *113*, 4583–4588.

S7. V.S. Chirvony, A. van Hoek, V.A. Galievsky, I.V. Sazanovich, T.J. Schaafsma, D. Holten, *J. Phys. Chem. B* **2000,** *104,* 9909-9917
